# Shugan Hewei Decoction Alleviates Cecum Mucosal Injury and Improves Depressive- and Anxiety-Like Behaviors in Chronic Stress Model Rats by Regulating Cecal Microbiota and Inhibiting NLRP3 Inflammasome

**DOI:** 10.3389/fphar.2021.766474

**Published:** 2021-12-20

**Authors:** Yingying Yue, Yu Chen, Hao Liu, Lesi Xu, Xian Zhou, Hao Ming, Xin Chen, Miaoqi Chen, Yunya Lin, Lin Liu, Yingqian Zhao, Songlin Liu

**Affiliations:** ^1^ College of Traditional Chinese Medicine, Hubei University of Chinese Medicine, Wuhan, China; ^2^ Institute of Classical Prescription Applications, Hubei University of Chinese Medicine, Wuhan, China; ^3^ School of Medicine, Jianghan University, Wuhan, China

**Keywords:** chronic stress, cecal microbiota, NLRP3 inflammasome, Shugan Hewei Decoction, traditional Chinese medicine

## Abstract

Chronic stress is a significant cause of depression, anxiety, and intestinal mucosal injury. Gut microbiota disturbances are also associated with these disorders. Shugan Hewei Decoction (SHD), which is a traditional Chinese medicine formula developed by our team, has shown superior therapeutic effects in the treatment of depression, anxiety, and functional gastrointestinal diseases caused by chronic stress. In this study, we investigated the modulatory effect of SHD on the cecal microbiota and cecum mucosal NOD-like receptor protein 3 (NLRP3) inflammasome in a chronic unpredictable stress (CUS)/social isolation rat model. After the SHD intervention, the CUS model rats showed improvements in their depressive- and anxiety-like behaviors, as well as sustained body weight growth and improved fecal characteristics. SHD improved the cecal microbiota diversity and changed the abundance of six microbial genera. A Spearman’s correlation analysis showed a strong correlation between the NLRP3 inflammasome and CUS-perturbed cecal biomarker microbiota. SHD regulated the excessive expression of NLRP3, ASC, caspase-1, interleukin-1β (IL-1β), and IL-18 in the serum and cecum mucosa induced by CUS, as well as the activation of the Toll-like receptor 4/nuclear factor-κB signaling cascades. Our results reveal the pharmacological mechanisms of SHD and provide a validated therapeutic method for the treatment of depression, anxiety, and cecum mucosal injury.

## Introduction

Chronic stress due to social pressure or negative emotional stimulation can lead to psychological and somatic manifestations, such as anxiety, depression, and functional gastrointestinal disorders (Qin et al., 2014; Stefanaki et al., 2018), all of which endanger public physical and psychological health (Baxter et al., 2013). Depression and anxiety are the most common psychiatric disorders, and they are currently recognized by WHO as the fourth most prevalent contributors to the global burden of disease (Liang et al., 2016). Depression and anxiety can disturb the homeostasis of the gastrointestinal tract, resulting in loss of appetite, abdominal distension, abdominal pain, abnormal bowel habits, changes in fecal traits, and other symptoms (Bhatia and Tandon, 2005). Chronic stress-induced gastrointestinal symptoms are associated with intestinal mucosal injury, which further exacerbates inflammatory reactions (Wei et al., 2019). Moreover, the intestinal microbiome plays an important role in the incidence of gastrointestinal diseases induced by mental and psychological factors (Karl et al., 2018). Intestinal microbiome disturbances have been reported in both chronic stress primate (Zheng et al., 2020) and rodent models (Ding et al., 2020), and these disturbances are related to NOD-like receptor protein 3 (NLRP3) inflammasome activation in the intestinal mucosa (Inserra et al., 2018; Hao et al., 2021). The NLRP3 inflammasome acts as a bridge between stress and the intestinal immune response, and it plays a dual role in promoting inflammation and maintaining intestinal homeostasis, both of which affect gastrointestinal function (Lamkanfi and Dixit, 2014). The intestinal microbiota and NLRP3 inflammasome are involved in the development of depression, anxiety, and functional gastrointestinal disorders that are induced by chronic stress; thus, they are recognized as potential targets for managing stress-related diseases.

The NLRP3 inflammasome, which is a multimeric protein complex consisting of a cytoplasmic innate receptor (NLRP3), an apoptosis-associated speck-like protein containing a CARD domain (ASC), and a cysteinyl aspartate specific proteinase (caspase-1) (Kim and Jo, 2013), is responsible for activating inflammatory responses upon infection and cellular damage. NLRP3 inflammasome activation correlates with stress responses (Fleshner et al., 2017), depressive- and anxiety-like behaviors, and gut microbiota composition (Wong et al., 2016). It also plays a major role in regulating chronic intestinal inflammation and the maturation of interleukin-1β (IL-1β) and IL-18, both of which are associated with an increased risk of colitis development (Perera et al., 2017). The NLRP3 inflammasome is closely related to the intestinal microbiota, which plays an important role in maintaining gastrointestinal and mental health along the brain–gut axis that connects the central nervous system and the human gastrointestinal tract. There is mounting data indicating that cumulative stress resulting from psychological, environmental, and physical stressors has a consistent and meaningful impact on the intestinal microbiota (Karl et al., 2018), and intestinal microbiota participate in the pathogenesis of gastrointestinal dysfunction mediated by chronic stress.

**FIGURE 1:**
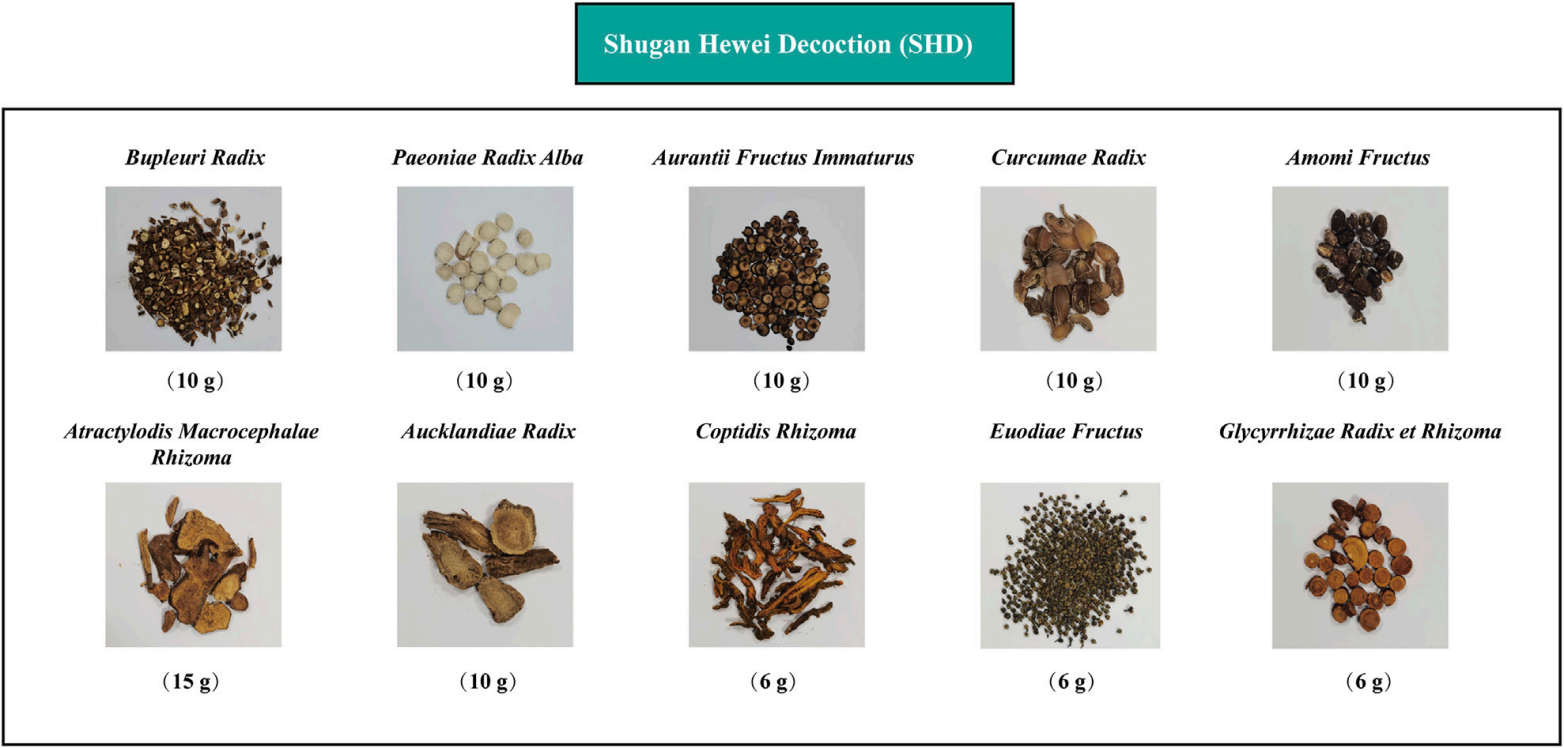
Chinese herbs formula of Shugan Hewei Decoction (SHD).

Shugan Hewei Decoction (SHD), which is a traditional Chinese medicine formula developed by our team, is used clinically to treat various stomach disorders, including functional gastrointestinal diseases induced by chronic stress. It was developed based on the formula for Sini San (SNS) in *Treatise on Febrile Diseases*, which is an ancient Chinese medicine book that was written about 1,800 years ago. SNS is composed of four herbs: *Bupleurum chinense* DC. (Apiaceae; Bupleuri Radix), *Paeonia lactiflora* Pall. (Paeoniaceae; Paeoniae Radix Alba), *Citrus × aurantium* L. (Rutaceae; Aurantii Fructus Immaturus), and *Glycyrrhiza uralensis* Fisch. ex DC. (Fabaceae; Glycyrrhizae Radix et Rhizoma). It has been applied clinically for the treatment of mental and gastrointestinal disorders, including depression and functional gastrointestinal disorders (Shen et al., 2016). SNS can improve depressive behaviors by inhibiting inflammation (Zong et al., 2019). SHD, which is an advanced version of SNS that has six additional Chinese herbs, provides improved therapeutic effects and longer efficacy for managing depression and gastrointestinal disorders. SHD is composed of the following commonly used Chinese herbs: *Bupleurum chinense* DC. (Apiaceae; Bupleuri Radix), *Paeonia lactiflora* Pall. (Paeoniaceae; Paeoniae Radix Alba), *Citrus × aurantium* L. (Rutaceae; Aurantii Fructus Immaturus), *Curcuma aromatica* Salisb. (Zingiberaceae; Curcumae Radix), *Wurfbainia villosa* (Lour.) Skornick. and A.D.Poulsen (Zingiberaceae; Amomi Fructus), *Atractylodes macrocephala* Koidz.(Asteraceae; Atractylodis Macrocephalae Rhizoma), *Aucklandia costus* Falc. (Asteraceae; Aucklandiae Radix), *Coptis chinensis* Franch. (Ranunculaceae, Coptidis Rhizoma), *Tetradium ruticarpum* (A.Juss.) T.G.Hartley (Rutaceae; Euodiae Fructus), and *Glycyrrhiza uralensis* Fisch. ex DC. (Fabaceae; Glycyrrhizae Radix et Rhizoma) (Figure 1). Our previous studies have shown that the SHD formulation and its active substance fractions can improve depressive-like behaviors in a chronic unpredictable stress (CUS)–induced rat model by regulating the release of neurotransmitters (Glu and GABA) in the hypothalamus and inducing the synergistic effects of several neurotransmitters (DA, 5-HT, NA, 5-HIAA) in the PFC-NAc-VTA neural circuit (Mou et al., 2019; Chen et al., 2021). However, the mechanism by which SHD improves gastrointestinal disorders and whether cecal microbiota, intestinal inflammation, and the NLRP3 inflammasome are related to the SHD antidepressant mechanism remain unclear.

In this study, we identified the chemical constituents of SHD and analyzed the active compounds in medicated rat serum using HPLC-TOF-MS/MS. We demonstrated that SHD may alleviate cecum mucosal injury by maintaining intestinal microbiota homeostasis and inhibiting the excessive activation of the NLRP3 inflammasome and Toll-like receptor 4 (TLR4)/nuclear factor (NF)-κB signaling pathway, which also improves depressive-like behaviors and the ability of SHD to regulate neurotransmitter release. Our study provides an advanced therapeutic method for the treatment of chronic stress-related diseases.

## Materials And Methods

### Animal Care and Use

**FIGURE 2:**
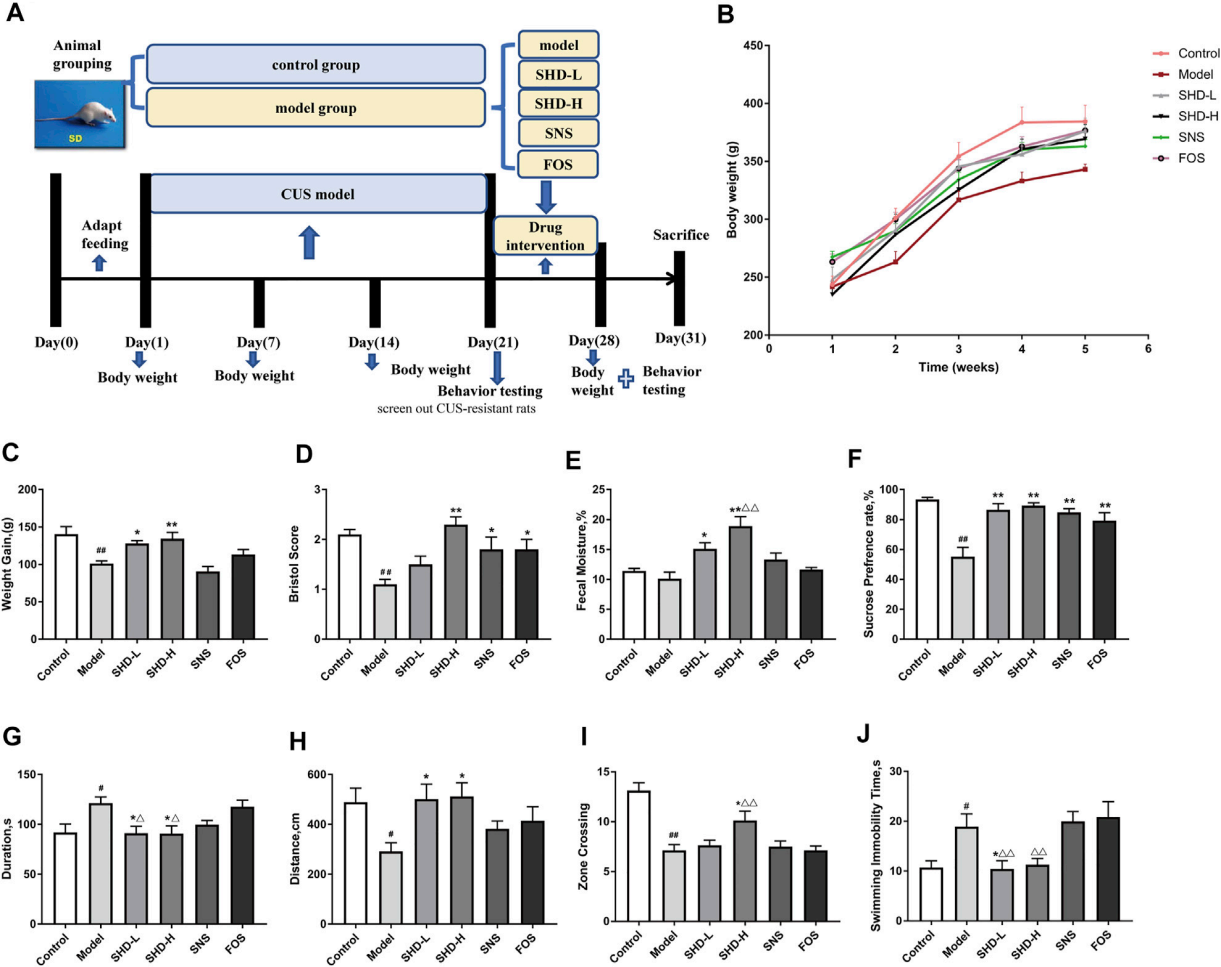
Schematic diagram of the experimental procedures used in this study and the effects of SHD on the weight, fecal traits, and depressive-like behaviors of chronic unpredictable stress (CUS) model rats. **(A)** An experimental flowchart detailing the feeding, grouping, drug interventions, and measuring of the rats. **(B)** Changes in the body weights of the CUS model rats (*n* = 8). **(C)** Effect of SHD on the weight gains experienced by the CUS model rats (*n* = 8–9). **(D)** Effect of SHD on the Bristol evaluation scores for the CUS model rats (*n* = 10). **(E)** Effect of SHD on the fecal moisture content in the CUS model rats (*n* = 5). **(F)** The sucrose preference rate from the sucrose preference test (SPT) (*n* = 8). **(G)** The stationary time observed during the open field test (OFT) (*n* = 8). **(H)** The total movement distance observed during the OFT (*n* = 8). **(I)** The number of central areas crossing the lattice during the OFT (*n* = 8). **(J)** The immobility time observed during the forced swimming test (FST) (*n* = 8). The data are expressed as the mean ± SEM. ^#^ *p* < 0.05, ^##^ *p* < 0.01 *vs.* the control group. *p < 0.05, **p < 0.01 *vs*. the model group. ^**Δ**^ *p*<0.05, ^**ΔΔ**^ *p*<0.01 *vs.* the FOS group. SHD-L, the low dosage of the Shugan Hewei Decoction; SHD-H, the high dosage of the Shugan Hewei Decoction; SNS, Sini San; FOS, fructooligosaccharide.

A total of 84 healthy male Sprague–Dawley SPF rats (SYXK (E) 2017-0067) weighing 160–200 g were purchased from the Chinese Center for Disease Control and Prevention (Wuhan, Hubei, China), and subjected to a 7-day adaptive feeding process prior to starting the formal experiment. The rats were housed in the SPF Animal Lab at the Hubei University of Chinese Medicine under a 12-h light/dark cycle at a constant temperature (25 ± 1°C) and humidity (55–65% relative humidity) with *ad libitum* access to food and water. After 7 days of acclimatization to the environment, 80 rats with similar body weights were selected and randomly divided into a control group (12 rats) and a model group (68 rats), and all the model group rats were housed independently. The model rats were subjected to seven different chronic unpredictable stress (CUS) for 21 days. The 8 CUS-resistant rats were screened out using the sucrose preference test (SPT), open field test (OFT), and forced swimming test (FST) after 3 weeks of exposure to CUS. The remaining 60 rats were randomly divided into the following five groups: a model group, a low SHD dosage (SHD-L) group, a high SHD dosage (SHD-H) group, a SNS group, and a fructooligosaccharide (FOS) group, with 12 rats in each group (Figure 2A). The animal experiments were reviewed and approved by the Animal Ethics Committee (HUCMS 201903009), and all the experiments complied with current animal welfare guidelines.

### Drug Preparation and Intervention

The SHD consists of 10 herbs, containing *Bupleurum chinense* DC. (Apiaceae; Bupleuri Radix), 10 g; *Paeonia lactiflora* Pall. (Paeoniaceae; Paeoniae Radix Alba), 10 g; *Citrus × aurantium* L. (Rutaceae; Aurantii Fructus Immaturus), 10 g; *Curcuma aromatica* Salisb. (Zingiberaceae; Curcumae Radix), 10 g; *Wurfbainia villosa* (Lour.) Skornick. and A.D.Poulsen (Zingiberaceae; Amomi Fructus), 10 g; *Atractylodes macrocephala* Koidz. (Asteraceae; Atractylodis Macrocephalae Rhizoma), 15 g; *Aucklandia costus* Falc. (Asteraceae; Aucklandiae Radix), 10 g; *Coptis chinensis* Franch. (Ranunculaceae, Coptidis Rhizoma), 6 g; *Tetradium ruticarpum* (A.Juss.) T.G.Hartley (Rutaceae; Euodiae Fructus), 6 g; and *Glycyrrhiza uralensis* Fisch. ex DC. (Fabaceae; Glycyrrhizae Radix et Rhizoma), 6 g (Figure 1, Supplementary Table S1). The SNS consists of four herbs, containing *Bupleurum chinense* DC. (Apiaceae; Bupleuri Radix), 10 g; *Paeonia lactiflora* Pall. (Paeoniaceae; Paeoniae Radix Alba), 10 g; *Citrus × aurantium* L. (Rutaceae; Aurantii Fructus Immaturus), 10 g; and *Glycyrrhiza uralensis* Fisch. ex DC. (Fabaceae; Glycyrrhizae Radix et Rhizoma), 10 g. All the raw herbs were provided by Tianji Pharmaceutical Co., Ltd (Hubei, China) and produced based on the procedure described in the Chinese Pharmacopoeia 2015 Edition (National Pharmacopoeia Commission, 2015). The weights of each herb were determined according to the clinical dose. Dry FOS powder (Batch No. 1903303301, purity ≥95.0%) was prepared by the Quantum Hi-Tech Biological Co. Ltd. (Guangzhou City, China) in accordance with the process described in the PRC National Standard GB/T 23528-2009.

Briefly, SHD raw herb weights in total of 930 g (10 doses of raw herbs of SHD) was soaked and then extracted with 10-fold mass of water (9,300 ml). First, 1.38 g of volatile oil (yield of 0.15%) and 23.7 g of aromatic water (yield of 2.55%) were extracted by steam distillation and then stored at −20°C; then by water extraction and alcohol precipitation method, the raw herb was boiled for 3 times, 2 h each, the water exact from each time were retrieved and mixed, then concentrated down to 930 ml (equal to 1 g crude herb/ml) by water bath method. The water extract was filtrated and 74.20 g of polysaccharides was obtained (yield of 7.98%), and the remaining liquid supernatant was concentrated by rotary evaporator and 242.20 g of extractum was obtained (yield of 26.0%); the polysaccharides and extractum were kept in a desiccator before use. The SHD total extract was obtained with a yield of 36.7% (341.48 g: 930 g). Distilled water was used to solve SHD total extract into liquid with the concentration of 0.56 g crude drug/ml (the SHD low dose) and 1.12 g crude drug/ml (the SHD high dose). The SNS preparation method is the same as that of SHD. The SNS total extract was obtained with a yield of 38.4% (153.6 g: 400 g) and SNS liquid with the concentration of 0.48 g crude drug/ml. The FOS solution is prepared according to the relevant literature (Chi et al., 2020), with the concentration of 0.315 g/ml. After completion, all the solution was divided and sterilized, and then stored at 4°C for later. The intragastric administration dosage was calculated based on the clinical equivalent dosage for an adult. The FOS was administered intragastrically (3.15 g/kg/day). The SHD-L dosage was 1.34 g/kg/day (3.67 g crude drug/kg/d), and the SHD-H dosage was 2.68 g/kg/day (7.34 g crude drug/kg/day). The high dose was the clinical equivalent. The SNS dosage was 1.21 g/kg/day (3.15 g crude drug/kg/day) (Zhu et al., 2020). The control group rats were given free access to food and water, and five rats were housed in each cage. The model group rats received an equal volume of distilled water, while the other four groups received the corresponding drug interventions for 7 days, twice a day.

### HPLC-TOF-MS/MS Analysis of SHD

An HPLC-TOF-MS/MS analysis was conducted to identify the main chemical components of the SHD preparation. The SHD concentration was 1 g/ml (a 1-ml solution containing 1 g of the original herbs). The standards for paeoniflorin (Lot X12A8C33672) and palmatine chloride (Lot Z12J7X15968) were provided by Shanghai Yuanye Bio-Technology Co., Ltd. (purity ≥98%, Chengdu, China). Saikosaponin A (Lot 110,777–201,711, purity ≥91.3%), naringin (Lot 110,722–201,815, purity ≥91.3%), neohesperidin (Lot 111,857–201,703, purity ≥99.2%), hesperidin (Lot 10,721–201,818, purity ≥96.2%), liquiritin (Lot 111,610–201,607, purity ≥93.1%), and glycyrrhetinic acid (Lot 110,723–201,715, purity ≥99.6%) were provided by the National Institutes for Food and Drug Control (Beijing, China). The HPLC was performed on an Agilent 1260 Infinity HPLC system (Agilent Technologies, Santa Clara, California, USA) equipped with an Agilent ZorBax Eclipse Plus C18 RRHD (2.1 × 50 mm, 1.8 µm). The mobile phase was eluted with 0.1% formic acid ultra-pure water (A) and acetonitrile (B) in gradient mode. The acetonitrile proportion varied according to the gradient elution program: 0–3 min, 90% A; 3–8 min, 90–65% A; 8–10 min, 65–40% A; 10–16 min, 40–20% A at a flow rate of 0.4 ml/min. An electrospray ion source was used to collect the data under the positive and negative ion modes. The full-mass scanning range was 150–1,500 m/z. The column temperature was 30°C, and the detection wavelength was set at 275 nm.

### Preparation of SHD-Containing Serum

Briefly, to collect the SHD-containing serum and the control serum, 20 Sprague–Dawley rats were randomly divided into two groups: one group was treated with SHD (14.68 g/kg), and the other group was treated with normal saline (10 ml/kg). Blood samples from each of the rats were then centrifuged at 3,500×*g* for 10 min at 4°C, and the serum was filtered through a 0.22-µm microporous membrane.

### Chronic Unpredictable Stress

The CUS procedure was performed as previously described (Willner et al., 1987), although with slight modifications. In brief, the rats were exposed to a randomly selected stressor daily for 21 days. The stressors included food deprivation for 24 h, water deprivation for 24 h, tail squeezing for 1 min, swimming in 4°C water for 5 min, shaking 1 time/s for 5 min, reversal of the light/dark cycle, and shocking the plantar surface (50 mV, stimulation once every 5 s, intermittent 5 s, 10 times in total; Supplementary Table S2). Food and water were freely available to the control group rats, which remained undisturbed on another shelf, except for the 12-h deprivation period prior to each sucrose test.

### Body Weight and Feces Traits

The body weight of each of the rats was recorded 1 day prior to the initiation of the experiments (day 0), as well as on days 7 and 21 of the interventions and 7 days (day 28) after the interventions. The body weight growth was calculated using the formula (Wn − W28)/W28 × 100%, in which W28 is the body weight on the last day of the intervention and W0 is the body weight on the last acclimation day. Changes in the general conditions and fecal characteristics between the groups and at different time points were recorded. The fecal Bristol scores and moisture contents were measured 12 h after the end of the last administration and before the sample collection. Rats in each group were placed in metabolic cages and fed in single cages lined with white paper. Centrifuge tubes (50 ml) were placed at the bottom of the cages to separate urine, avoiding the influence on the feces traits. The fecal traits of the rats were observed for 12 h, after which fecal samples were collected in centrifuge tubes. The average score of Bristol was calculated for each group according to the Bristol stool form scale (Supplementary Table S3) (Lewis and Heaton, 1997). The weight of the wet fecal samples was measured, and the average value was recorded as “A”. The centrifuge tubes were then placed in a drying box for 2 h, after which they were weighed again, with the average value recorded as “B”. The fecal moisture content was calculated using the following formula: fecal moisture content = (A − B)/A × 100%.

### Behavioral Testing

Behavioral testing was performed as previously described (Moretti et al., 2019) in 7 days after the interventions (day 28).

For the SPT, the rats were given two bottles of 1% sucrose solution on the first training day. After 24 h, one of the 1% sucrose bottles was replaced with sterile water. After the adaptation phase, the rats were deprived of water and food for 23 h. The rats were then given free access to both water and 1% sucrose solution for 2 h, after which the remaining amounts of water and sucrose solution were measured. The sucrose preference ratio was determined using the following formula: sucrose preference ratio (%) = sucrose intake (ml) × 100%/[sucrose intake (ml) + water intake (ml)].

The OFT was performed at the end of the SPT. The OFT device included a field reaction box (90 cm × 90 cm×45 cm) and an automatic data acquisition system. The color of the inner wall of the reaction box was black, and the bottom surface was divided into small squares (5 cm × 5 cm). A digital camera was secured 2 m above the device, and its field-of-view covered the entire interior of the box. During the experiment, a rat was placed in the center of the reaction box, which was in a dark and quiet room. A SMART 3.0 animal behavior video acquisition system (Panlab, Barcelona, Spain) was used to record images of the rats for 5 min during the OFT, and the total movement distance, stationary time, and number of times that the rats crossed the central areas were analyzed over a 3-min span. The OFT was scheduled to take place between 8 and 12 a.m. To minimize cross-contamination, the experimental area was cleaned using 75% ethanol after each rat completed the test. To avoid subjectivity errors, both anxiety-like behavior tests were independently evaluated by two trained observers.

The FST was used to detect depressive-like behavior as characterized by immobility during a forced swim, as we described previously (Brenes et al., 2006). A pre-forced swim was conducted for 15 min 1 day before the actual test. During the test, each rat was placed inside a cylinder filled with water (the water temperature was controlled at 25 ± 1°C). During the FST, there is a period of vigorous activity during which the rat tries to escape, followed by a characteristic immobility, in which the rat only moves to maintain its head above water. In the current study, the immobility time was defined as the time during which the rat stopped struggling and floated in the water or only made small body movements to keep its head afloat. In the FST, this physical immobility is used as an indicator of behavioral despair. The immobility time for each rat was recorded and measured independently by two trained observers within a test period of 6 min.

### Sample Collection and Preparation

The rats were anesthetized using an intraperitoneal injection of pentobarbital sodium (50 mg/kg), after which different sample materials were collected from the rats in each group. For the rats numbered 1–6 in each group: first, a 6-ml sample of blood was taken from the abdominal aorta and placed in a coagulation tube at room temperature for 1 h, after which the tubes were placed at 4°C overnight. The blood was then centrifuged at 3,000 rpm at 4°C for 10 min, and the serum was collected from each tube and stored at −20°C for later use. The cecal contents (2 ml) were collected from each rat using a sterile tube under sterile environmental conditions, and approximately 1–2 cm of cecal tissue was also collected. The aforementioned samples were immediately stored at −80°C. The serum samples were analyzed using ELISA to detect the expression of the NLRP3 inflammasome and related inflammatory cytokines. The cecal contents were sequenced using the 16S rRNA high-throughput method to detect the structure of and changes in the cecal microbiota. The cecal tissues were detected using RT-PCR and western blotting to observe the expression of related mRNAs and proteins.

A 4% paraformaldehyde aortic perfusion and fixation was performed for the rats numbered 7–12 in each group. The cecal contents (2 ml) were collected from each of these rats using sterile tubes under sterile environmental conditions, and the cecal samples were immersed in liquid nitrogen and ultimately transferred to −80°C. In addition, cecal tissues (1–2 cm) were separated and fixed in 4% paraformaldehyde at 4°C for 4–6 h. The cecal tissues were then embedded in paraffin and sagittal sections were removed. The pathological morphology of the fixed cecal tissues was observed using H&E staining. An immunofluorescence (IF) co-localization analysis was used to detect the NLRP3 inflammasome in the cecal tissues. The cecal content detection method for these samples was the same as described previously.

### H&E Staining of Cecal Tissue

After the cecal tissues were fixed in 4% paraformaldehyde, they were dehydrated using gradient ethanol and embedded in paraffin. The paraffin sections were then deparaffinized, and the tissues were cut into 4-μm-thick sections and stained with H&E (hematoxylin stained the nucleus and eosin stained, dehydrated, and sealed the cytoplasm). Finally, images of the cecum mucosal tissues were captured using an optical microscope (Nikon Eclipse CI, Tokyo, Japan) and analyzed using an image acquisition system (Nikon DS-U3, Tokyo, Japan) to evaluate their histopathology.

### 16S rRNA Gene Sequencing of Cecal Contents

#### DNA Extraction and PCR Amplification

Microbial community genomic DNA was extracted from the cecal contents samples using the E.Z.N.A.® soil DNA Kit (Omega Bio-tek, Norcross, GA, USA) according to the manufacturer’s instructions. The DNA extract was checked on a 1% agarose gel, and DNA concentration and purity were determined using a NanoDrop 2000 UV–vis spectrophotometer (Thermo Scientific, Wilmington, DE, USA).

The V3–V4 hypervariable region of the bacterial 16S rRNA gene was amplified with primer pairs 338 F (5′-ACT​CCT​ACG​GGA​GGC​AGC​AG-3′) and 806 R (5′-GGACTACHVGGGTWTCTAAT-3′) using an ABI GeneAmp 9700 PCR thermocycler (ABI, Foster City, CA, USA). The PCR amplification was performed as follows: initial denaturation at 95°C for 3 min, followed by 27 cycles of denaturing at 95°C for 30 s, annealing at 55°C for 30 s and extension at 72°C for 45 s, and single extension at 72°C for 10 min, and end at 4°C. The PCR mixtures contained 5× TransStart FastPfu buffer (4 μl), 2.5 mM dNTPs (2 μl), 5 μM forward primer (0.8 μl), 5 μM reverse primer (0.8 μl), TransStart FastPfu DNA polymerase (0.4 μl), template DNA (10 ng), and ddH_2_O (up to 20 μl). The PCR reactions were performed in triplicate, and the PCR product was extracted from a 2% agarose gel and purified using an AxyPrep DNA Gel Extraction Kit (Axygen Biosciences, Union City, CA, USA) according to the manufacturer’s instructions. The purified product was then quantified using a Quantus Fluorometer (Promega, Madison, WI, USA).

#### Illumina MiSeq Sequencing

The purified amplicons were pooled in equimolar amounts and paired-end sequenced on an Illumina MiSeq PE300/NovaSeq PE250 platform (Illumina, San Diego, CA, USA) according to the standard protocols developed by Majorbio Bio-Pharm Technology Co. Ltd. (Shanghai, China). The raw reads were deposited in the NCBI Sequence Read Archive database (accession number: SRP324274).

#### Processing of Sequencing Data

The raw 16S rRNA gene sequencing reads were demultiplexed, quality-filtered by fastp version 0.20.0 (Chen et al., 2018), and merged using FLASH version 1.2.7 (Magoč and Salzberg, 2011) with the following criteria: 1) the 300-bp reads were truncated at any site receiving an average quality score <20 over a 50-bp sliding window, and the truncated reads shorter than 50 bp were discarded, reads containing ambiguous characters were also discarded; 2) only overlapping sequences longer than 10 bp were assembled according to their overlapped sequence. The maximum mismatch ratio of overlap region is 0.2. Reads that could not be assembled were discarded; 3) samples were distinguished according to the barcode and primers, and the sequence direction was adjusted, exact barcode matching, two nucleotide mismatches in primer matching. Operational taxonomic units (OTUs) with a 97% similarity cutoff (Stackebrandt and Goebel, 1994; Edgar, 2013) were clustered using UPARSE version 7.1 (Edgar, 2013), and chimeric sequences were identified and removed. The taxonomy of each OTU representative sequence was analyzed against the 16S rRNA database (e.g., Silva v138) by RDP Classifier version 2.2 (Wang et al., 2007) using confidence threshold of 0.7.

### ELISA of Serum

The NLRP3 expression levels in the serum were measured using ELISA kits (Wuhao Inc., Shanghai, China) according to the manufacturer’s instructions. The expression levels of caspase-1, IL-1β, IL-10, and IL-18 in the serum were also measured using ELISA kits (Cusabio Inc., Wuhan, Hubei, China) according to the manufacturer’s instructions.

### RT-PCR of Cecal Tissues

Total RNA was extracted from the rat tissues using Trizol reagent, and the RNA integrity was confirmed using agarose gel electrophoresis. CDNA was reverse transcribed from the mRNA using a reverse transcription kit, and RT-qPCR was performed using SYBR green assays. The sequences of the target genes were identified using the NCBI database, and primer5 software was used to design the primers. The sequences of the primer pair were as follows: NLRP3: 5ʹ-CAG​AAG​CTG​GGG​TTG​GTG​AA-3ʹ (forward) and 5ʹ-CAG​CAG​GAG​TGT​GAG​GTG​AG-3ʹ (reverse), product length 218 bp; caspase-1: 5ʹ-AGC​TTC​AGT​CAG​GTC​CAT​CAG-3ʹ (forward) and 5ʹ-AAG​ACG​TGT​ACG​AGT​GGG​TG-3ʹ (reverse), product length 227 bp; IL-1β: 5ʹ-TGT​GAT​GTT​CCC​ATT​AGA​C-3ʹ (forward) and 5ʹ-AAT​ACC​ACT​TGT​TGG​CTT​A-3ʹ (reverse), product length 139 bp; ASC: 5ʹ-GGA​CAG​TAC​CAG​GCA​GTT​CG-3ʹ (forward) and 5ʹ-GTC​ACC​AAG​TAG​GGC​TGT​GT-3ʹ (reverse), product length 140 bp; IL-18: 5ʹ-ACA​GCC​AAC​GAA​TCC​CAG​AC-3ʹ (forward) and 5ʹ-TCC​ATT​TTG​TTG​TGT​CCT​GGC-3ʹ (reverse), product length 224 bp; actin: 5ʹ-TCC​TCT​GTG​ACT​CGT​GGG​AT-3ʹ (forward) and 5ʹ-TGG​AGA​ATA​CCA​CTT​GTT​GGC​T-3ʹ (reverse), product length 174 bp. The 2^−ΔΔCt^ method was used to analyze the relative gene expression levels.

### Immunofluorescence of Cecal Tissues

All the sections were analyzed by an investigator blinded to the diagnosis data. Positive staining results for NLRP3, caspase-1, and ASC were counted in each of the three different microscopic fields. The cell densities are expressed as the mean numbers of positively stained cells per field. The results of the double-staining for NLRP3 (green) and caspase-1 (red) and for NLRP3 (green) and ASC (red) were analyzed in a similar manner.

### Western Blot of Cecal Tissues

Proteins were extracted from the cecal tissues by adding RIPA lysis buffer to the samples, followed by repeated grinding, incubation on ice for 10 min, and centrifugation at 16,000×*g* for 15 min at 4°C to obtain the supernatant. For the western blot analysis, 240 μl of the protein supernatant was mixed with 60 μl of 5× loading buffer, after which the mixture was boiled for 10 min then cooled on ice. Tissue lysate samples were resolved using SDS-PAGE and transferred to nitrocellulose membranes. The membranes were then immersed in 5% defatted milk powder (prepared using 1× PBST) at room temperature for 90 min, after which they were incubated at 4°C overnight with the following primary antibodies diluted in 1× PBST: anti-TLR4, anti-NF-κB, anti-p-NF-κB, anti-myeloid differentiation factor 88 (MyD88) (1:1,000 dilution); and β-actin (1:3,000 dilution). The next day, the membranes were incubated at room temperature for 60 min with HRP goat anti-mouse IgG and HRP goat anti-rabbit IgG secondary antibodies, both of which were diluted 1:3,000 in 1× PBST. The membranes were then incubated at room temperature for 90 min, followed by five washes with ECL chemiluminescent solution for color development. The exposed films were scanned and analyzed using Quantity One software.

### Correlation Analysis

To explore the functional relationship between the cecal microbiota, the NLRP3 inflammasome (NLRP3, ASC, caspase-1), and the downstream inflammatory factors (IL-1β and IL-18) in the serum and cecal tissues, we formulated a correlation matrix based on Spearman’s correlation coefficient (*|R|*≥0.5, *p* < 0.05). A Spearman’s correlation was calculated and plotted using R (version 3.5.0, corrplot package).

### Statistical Analysis

The quantitative data were analyzed using GraphPad Prism 6 software (GraphPad Software, Inc., San Diego, CA, USA) and are presented as the mean ± SEM. The differences between the groups were compared using a two-way ANOVA and the Tukey–Kramer test. The significance of the comparisons between the groups for the non-parametric and parametric data with abnormal distributions was determined using the Kruskal–Wallis test, followed by the Mann–Whitney test (SPSS 11.5; IBM Corporation, Armonk, NY, USA). The results of the alpha diversity analysis of the cecal microbiota abundance and composition are expressed as the mean ± SD. A linear discriminant analysis (LDA) coupled with effect size measurement (LEfSe) of the cecal microbiota abundance and composition was based on the Kruskal–Wallis and Wilcoxon tests, and the LDA score threshold was 4.0–5.0. Statistical significance was set at *p* <0.05.

## Results

### HPLC-TOF-MS/MS Analysis of SHD

**FIGURE 3:**
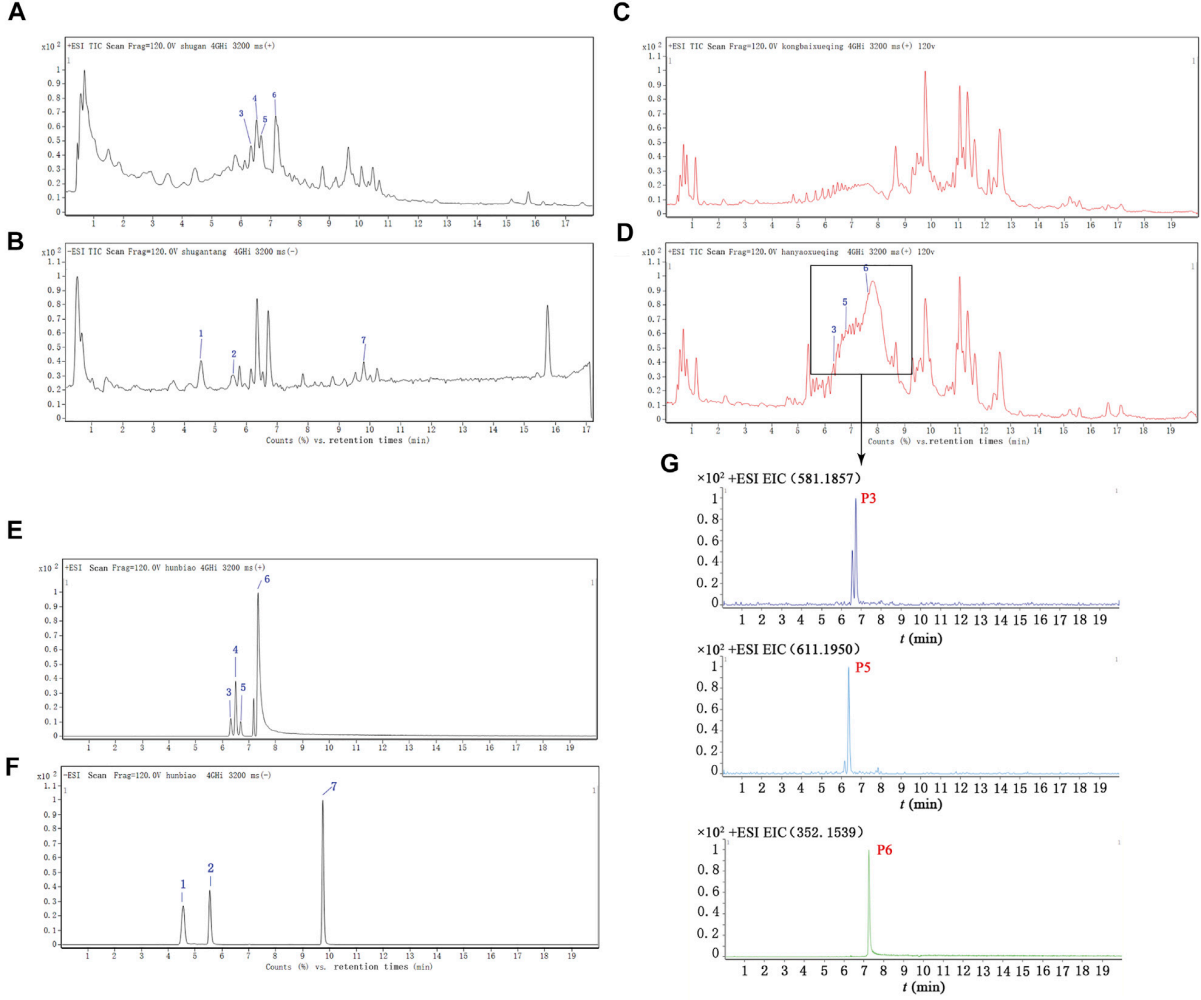
Total ion chromatograms (TICs) for SHD, the SHD-containing serum, and the standard substances. **(A)** The TIC for SHD in the positive mode. **(B)** The TIC for SHD in the negative mode. **(C)** The TIC for the control serum in the positive mode. **(D)** The TIC for the SHD-containing serum in the positive mode. **(E)** The TIC for the standard substances in the positive mode. **(F)** The TIC for the standard substances in the negative mode. **(G)** The ion intensity of the three prototype components identified in the SHD-containing serum. The numbers marked in the figure correspond to (1) paeoniflorin, (2) liquiritin, (3) naringin, (4) hesperidin, (5) neohesperidin, (6) palmatine chloride, and (7) Saikosaponin A.

The main components of SHD, the SHD-containing serum, and the pure standard substances are presented in Figure 3. The following seven major components were identified in the SHD extract: 1) paeoniflorin, 2) liquiritin, 3) naringin, 4) hesperidin, 5) neohesperidin, 6) palmatine chloride, and 7) Saikosaponin A (Table 1). In addition, we identified naringin, neohesperidin, and palmatine chloride in the SHD-containing serum.

**TABLE 1:** Herbal sources and retention times of seven components in SHD

Peak number	Retention times/min	Quasi-molecular ion (m/z)	Formula	Error range (10^−6^)	Fragment ion (m/z)	Ion detection mode	Constituents	Source
1	4.464	525.1624	C_23_H_28_O_11_	1.90	479.15, 449.15	−	Paeoniflorin	*Paeonia lactiflora* Pall.
2	5.530	419.1339	C_21_H_22_O_9_	0.48	257.08, 149.02	+	Liquiritin	*Glycyrrhiza uralensis* Fisch.
3	6.309	581.1854	C_27_H_32_O_14_	−1.89	419.13, 273.07	+	Naringin^a^	*Citrus aurantium* L.
4	6.513	611.1946	C_28_H_34_O_15_	−3.93	303.08, 153.01	+	Hesperidin	*Citrus aurantium* L.
5	6.684	611.1946	C_28_H_34_O_15_	−3.93	303.08, 153.01	+	Neohesperidin^a^	*Citrus aurantium* L.
6	7.139	352.1566	C_21_H_22_ClNO_4_	4.83	337.13, 322.11	+	Palmatine chloride^a^	*Coptis chinensis* Franch.
7	9.814	825.4623	C_42_H_68_O_13_	2.30	779.5	−	Saikosaponin A	*Bupleurum chinense* DC.

a Three prototype components were detected in the SHD-containing serum.

### SHD Maintains Weight Gain and Improves the Fecal Disorder of CUS Model Rats

The body weight changes in the rats mainly reflected the food intake level, metabolic status, gastrointestinal digestion, and absorption function of each of the rats. After inducing chronic stress, the feeding behavior of rats often changes, and this behavioral change was manifested as a reduction in body weight gain (Keser et al., 2020). The body weights of the rats in all the groups showed an increasing trend during the interventions (Figure 2B). The weight gain for the model group decreased from day 1 to day 28 compared with that of the control group (*p* < 0.01, Supplementary Table S4). However, the weight gain for the SHD-L and SHD-H groups increased significantly compared with the model group (*p* < 0.05 or *p* < 0.01), whereas the SNS group exhibited a slight reduction in body weight gain compared with the model group. Although there was not a significant difference between the FOS group and the model group, the FOS group still showed an upward trend in weight gain. On the other hand, both SHD-L and SHD-H alleviated the CUS-induced reduction in body weight gain, and SHD-H had a better effect on maintaining a healthy body weight gain (Figure 2C).

The Bristol stool scale divides stool samples into seven categories: types 1 and 2 indicate constipation (score 1); types 3 and 4 are ideal (score 2); type 4 is the easiest shape to defecate. Types 5–7 indicate possible diarrhea (score 3). The fecal Bristol score and fecal moisture content can reflect the frequency of defecation, fecal pattern, and intestinal function in rats (Li et al., 2012). As shown in Supplementary Table S4, decreased Bristol scores were observed for the CUS model rats compared with those for the control group (*p* < 0.01). Following the SHD-H treatment, the Bristol score and fecal moisture content for the rats were elevated compared with those of the model group (*p* < 0.01) (Figures 2D,E). There was not a significant difference in the fecal moisture content for the SNS and FOS groups compared with the model group; however, there was still an increasing trend. In short, these results indicate that SHD-H can improve fecal disorders and maintain weight gains in CUS model rats.

### SHD Improves Cecum Mucosal Injury in CUS Model Rats

**FIGURE 4:**
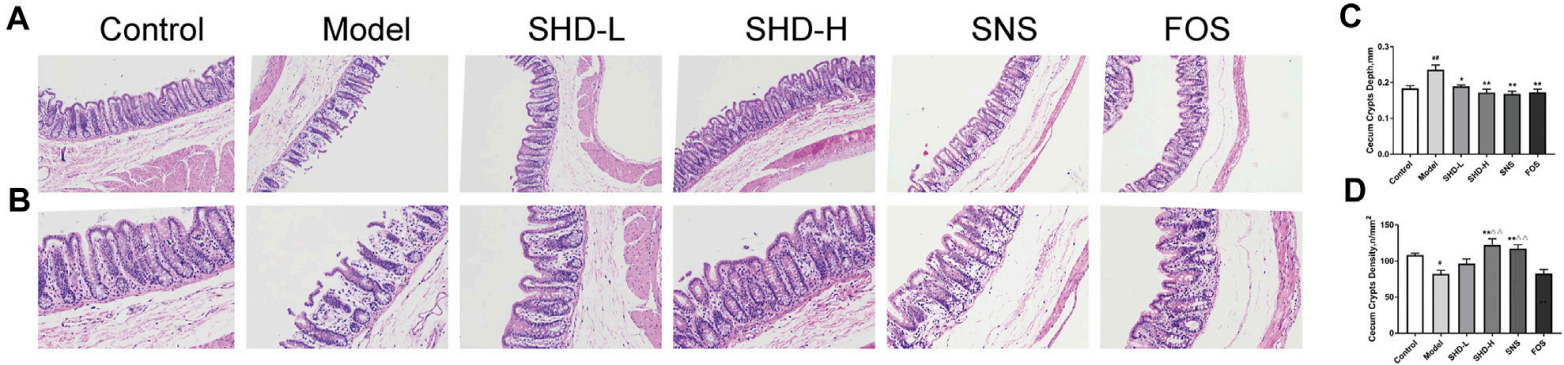
SHD alleviates cecum mucosal histopathology and improves cecum mucosal injury in CUS model rats. **(A)** Sections stained with H&E staining to assess the cecum mucosal injury (×100). Scale bar = 50 μm. **(B)** Sections stained with H&E to assess the cecum mucosal injury (×200). Scale bar = 50 μm. **(C)** Quantification of the mean cecum crypt depths (mm). **(D)** Quantification of the mean cecum crypt densities (n/mm^2^). The data are expressed as the mean ± SEM, *n* = 5. ^#^ *p* < 0.05, ^##^ *p* < 0.01 *vs.* the control group. *p < 0.05, **p < 0.01 *vs.* the model group. ^**Δ**^ *p*<0.05, ^**ΔΔ**^ *p*<0.01 *vs.* the FOS group.

To assess whether SHD can ameliorate cecum mucosal injury, we used H&E staining to determine the changes in the pathophysiological characteristics of the rats (Kolacz et al., 2019). As shown in Figures 4A,B, the cecal structure of the rats in the control group was intact, and the cecum mucosal folds were well arranged. In the CUS group, the epithelial structure was destroyed, the intestinal villi were ruptured, and the cross-sectional area of the cecum crypts was reduced. Moreover, inflammatory cell infiltration was observed in the cecum mucosa and muscular layer. Compared with the CUS group, the inflammatory cell infiltration and degree of cecum mucosa damage were both reduced in the SHD, SNS, and FOS groups to varying degrees. The cross-sectional area of the intestinal crypts in the SHD-H group was enlarged. A small amount of inflammatory cell infiltration can also be seen in the submucosa, and the intestinal villi remain continuous. Partial mucosal epithelial rupture and mucosal and submucosal infiltration of a small number of inflammatory cells were observed in the SNS group. The cecum mucosa folds continuously in the FOS group, and a small number of inflammatory cells are invading the cavity. As shown in Figure 4C, after 3 weeks of exposure to CUS, the cecum crypt depth increased significantly compared with that of the control group (*p* < 0.01), while the SHD-L, SHD-H, SNS, and FOS treatments markedly reversed the cecum crypt depth increase (*p* < 0.05 or *p* < 0.01). In addition, the cecum crypt density decreased significantly (*p* < 0.05) in the CUS model compared with the control group, but the SHD-H treatment completely reversed the reduction (*p* < 0.01) (Figure 4D). These results indicate that SHD can ameliorate cecum mucosal injury in CUS model rats.

### SHD Reverses CUS-Induced Depressive and Anxiety-Like Behaviors in CUS Model Rats

We compared the alterations in the depressive- and anxiety-like behaviors of the six rat groups using SPT, OFT, and FST. The SPT is an effective quantitative index for evaluating anhedonia (Liu et al., 2018). The effects of SHD on the sucrose preference of the CUS rats during the SPF are shown in Figure 2F (Supplementary Table S5). The 21-day stress procedure with separation caused a significant decrease in the sucrose preference of the CUS model group compared with the control group (*p* < 0.01), indicating that the CUS model was successfully created. The sucrose preferences increased (*p* < 0.01) in the SHD-L, SHD-H, FOS, and SNS groups compared with the model group. SHD-H showed the strongest effect on restoring the sucrose preference of the CUS rats.

The OFT is a method that is used to evaluate the autonomous and exploratory behaviors exhibited by experimental animals, as well as the stress they experience, when they are placed in a new environment; the test is based on the frequency and duration of certain behaviors (Roth and Katz, 1979). As shown in Figures 2G,H, and I (Supplementary Table S6), the model group exhibited a shorter total movement distance (*p* < 0.05), longer stationary time (*p* < 0.05), and fewer central areas crossing the lattice (*p* < 0.01) compared with the control group. After the SHD-H intervention, we observed a longer total movement distance, shortened stationary time, and increased number of central areas crossing the lattice (*p* < 0.05) for the rats compared with the model group. There was no significant difference in the OFT results among the SNS, FOS, and model groups.

The FST is a negative stress experiment designed to make use of the inability of animals to escape from harsh environments, which leads to desperation behaviors (Fitzgerald et al., 2019). As shown in Figure 2J (Supplementary Table S5), the model group showed a much longer immobility time (*p* < 0.05) compared with the control group. The SHD-L group (*p* < 0.05) showed a marked reduction in the total immobility time compared with the model group, while the SNS and FOS groups showed no differences. There was not a significant difference in the immobility time for the SHD-H group compared with the model group, although there was still a markedly decreasing trend. These results indicate that SHD can reverse depressive- and anxiety-like behaviors in CUS model rats with a better efficacy compared with SNS.

### SHD Remodels Cecal Microbiota Dysbiosis of CUS Model Rats

**FIGURE 5:**
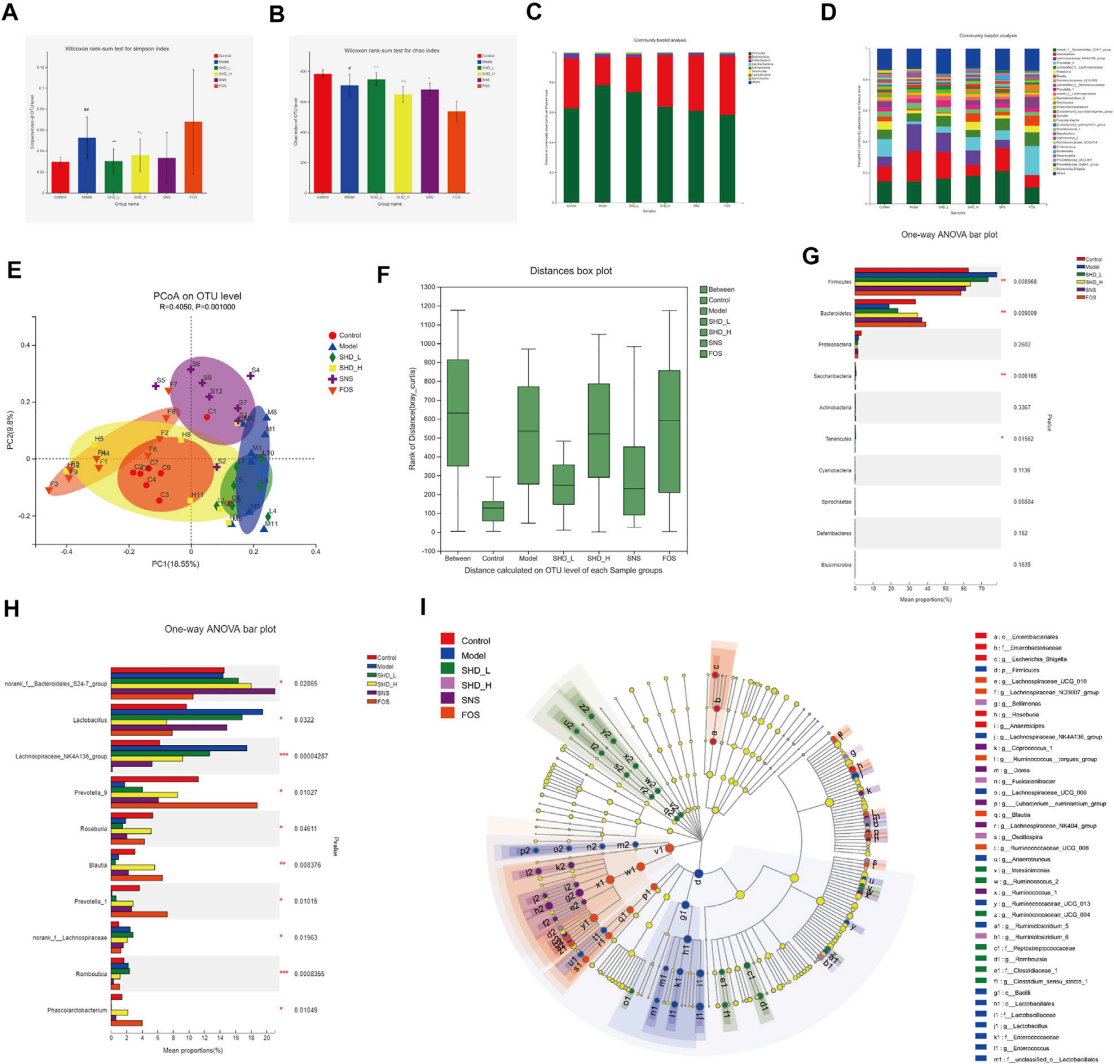
SHD remodels the cecal microbiota dysbiosis of CUS model rats. **(A)** Effect of SHD on the alpha diversity of the cecal contents from the rats in each group (*n* = 7–8), the Simpson index of the operational taxonomic unit (OTU) level. **(B)** Effect of SHD on the alpha diversity of the cecal contents from the rats in each group (*n* = 7–8), the Chao index of the OTU level. **(C)** Effect of SHD on the microbiota abundance (phylum level) in the rats in each group (*n* = 7–8). **(D)** Effect of SHD on the microbiota abundance (genus level) in the rats in each group (*n* = 7–8). **(E)** A principal coordinates analysis (PCoA) map based on the unweighted Unifrac matrix and Bray–Curtis dissimilarity. **(F)** An analysis of similarities of the distance calculated at the OTU level for each group. **(G)** Comparison of the relative microbiota abundance (phylum level) in the cecal contents from the rats in each group (*n* = 7–8). **(H)** Comparison of the relative microbiota abundance (genus level) in the cecal contents from the rats in each group (*n* = 7–8). **(I)** A linear discriminant analysis (LDA) coupled with effect size measurement (LEfSe) of the different microbiota in the cecal contents from the rats in each group, from the phylum level to the genus level (LDA score 3). The data are expressed as the mean ± SD. ^∗^Indicates statistical significance (^∗^0.01 < *p* ≤ 0.05, ^∗∗^0.001 < *p* ≤ 0.01, ^∗∗∗^ *p* ≤ 0.001).

We sequenced the V3–V4 region of the 16S rRNA gene of 49 samples from the six groups of rats, which yielded 2,888,146 high-quality reads for subsequent analysis, as an average of 20,351 sequences per sample (Supplementary Table S7). A total of 1,326 OTUs were generated through direct denoising, and 1,318 OTUs were obtained by drawing out the sample sequence according to the minimum number of sample sequences. A rank–abundance curve showed that the OTU classification level of the cecal contents from the rats in each group was higher in species richness and uniformity, and the proportion of some of the dominant microbiota was higher (Supplementary Figure S1). A pan-analysis indicated that the sample was sufficient, and the total bacterial species richness was high (Supplementary Figure S2). A core analysis indicated that the numbers of core microbiota in the cecal contents from the rats in each group was different (Supplementary Figure S3). The alpha diversity, as calculated using the Simpson and Chao indices, indicated that the CUS model led to greatly reduced microbiota populations and diversity relative to the control group, while the SHD-H and SNS treatments reversed this imbalance to varying degrees (Figures 5A,B and Supplementary Table S8). FOS promotes the proliferation and activity of beneficial bacteria in the intestine, although excessive FOS intake will disturb the original balance of the intestinal microbiota and reduce diversity. This may explain the poor results for the FOS group in this part of the analysis (Ten et al., 2003).

To understand the impact of SHD on the cecal microbiota, we further analyzed two different cecal microbiota taxonomic levels. At the phylum level, the CUS model rats showed a reduction in the relative abundance of *Bacteroidetes* and *Proteobacteria* and an increase in the abundance of *Firmicutes*. In contrast, SHD-H, SNS, and FOS significantly increased the levels of *Bacteroidetes* and decreased the levels of *Firmicutes* in the CUS model rats (Table 2 and Figure 5C). At the genus level, the distribution of microbiota among the different samples showed both similarity and distinctiveness. The CUS model rats showed an increase in the relative abundance of *Lactobacillus* and *Lachnospiraceae_NK4A136_group* and a reduction in the abundance *of Prevotellae_9*, *Roseburia*, *Blautia,* and *Prevotella_1.* In contrast, SHD-H and FOS significantly decreased the relative abundance of *Lactobacillus* and *Lachnospiraceae_NK4A136_group* and increased the abundance of *Prevotellae_9*, *Roseburia*, *Blautia*, and *Prevotella*_1 (Table 3 and Figure 5D).

**TABLE 2:** Effect of SHD on bacterial abundance (by phylum) in each group of rats (%, *n* = 7–8)

Phylum level	Control	Model	SHD-L	SHD-H	SNS	FOS
Firmicutes	62.71	78.22	73.56	63.73	61.03	58.46
Bacteroidetes	33.30	18.84	23.60	34.52	36.97	39.05
Proteobacteria	3.44	1.93	1.52	1.13	1.63	1.52
Saccharibacteria	0.17	0.46	0.61	0.20	0.03	0.31
Actinobacteria	0.17	0.23	0.25	0.27	0.13	0.40
Tenericutes	0.18	0.27	0.38	0.08	0.04	0.10
Cyanobacteria	0.01	0.03	0.02	0.01	0.11	0.03
Spirochaetae	0.01	0.00	0.02	0.03	0.00	0.12
Others	0.02	0.01	0.03	0.01	0.05	0.01

**TABLE 3:** Effect of SHD on bacterial abundance (by genus) in each group of rats (%, *n* = 7–8)

Genus level	Control	Model	SHD-L	SHD-H	SNS	FOS
*norank_f_Bacteroidales_S24-7_group*	14.47	14.44	16.29	17.99	21.09	10.55
*Lactobacillus*	9.67	19.50	16.88	7.13	14.87	7.86
*Lachnospiraceae_NK4A136_group*	6.27	17.41	12.66	9.15	5.22	0.15
*Prevotella_9*	11.21	1.72	3.95	8.59	6.08	18.67
*unclassified_f_Lachnospiraceae*	6.18	5.20	6.89	4.73	7.68	8.81
*Roseburia*	5.34	1.88	1.49	5.14	2.03	4.24
*Blautia*	3.06	0.96	0.57	5.56	2.23	6.61
*Ruminococcaceae_UCG-005*	3.26	3.91	3.64	2.60	2.77	2.31
*unclassified_f_Ruminococcaceae*	2.75	2.54	3.64	2.92	2.83	2.45
*Prevotella_1*	3.63	0.10	0.63	2.86	2.62	7.22
Others	34.15	32.33	33.36	33.32	32.59	31.12

A principal coordinates analysis revealed a distinct shift in the cecal microbiota composition in the CUS model rats treated with SHD-H, SNS, and FOS compared with the CUS rats (Figure 5E). The analysis of similarities (*R* = 0.4050, *p* = 0.0010) shows that the differences between the groups are more significant than the differences within the groups (Figure 5F). A LEfSe analysis was applied at the phylum and genus levels to determine the key microbial taxa that were differentially represented in the CUS model rats and the rats exposed to the various treatments. Ten key phyla and ten key genera were identified in the six groups (Figures 5G,H). As shown in Figure 5I, the cecal microbiota, such as *Firmicutes*, *Lactobacillaceae*, and *Bacteroides*, are taking part in distinguishing control from CUS model rats. Notably, *Roseburia*, *Tenericutes*, and *Fusicatenibacter* play vital roles in the SHD-treated groups, while the specific bacterial taxa in the SNS- and FOS-treatment groups were *Bacteroides_S24_7_group* and *Prevotella_1.*

To investigate the cecal microbiota functions associated with the SHD treatment, we used the PICRUSt software package to analyze the 16S rRNA gene profiles. When the data were analyzed using the COG database (Supplementary Figure S4), the microbiota functions were found to be mainly related to carbohydrate transport and metabolism, transcription, and amino acid transport and metabolism. When the Tax4Fun software package was used (Supplementary Figure S5), a KEGG pathway analysis showed that the predicted function of the microbiota was associated with carbohydrate metabolism, membrane transport, and the immune system. Collectively, these results indicate that CUS is associated with cecal microbiota disturbances, while SHD, SNS, and FOS remodel cecal microbiota dysbiosis *in vivo*. Notably, the abundance and proportion of the cecal microbiota in the SHD-H group were the closest to those of the control group.

### SHD Regulates NLRP3 Inflammasome Expression in CUS Model Rats

**FIGURE 6:**
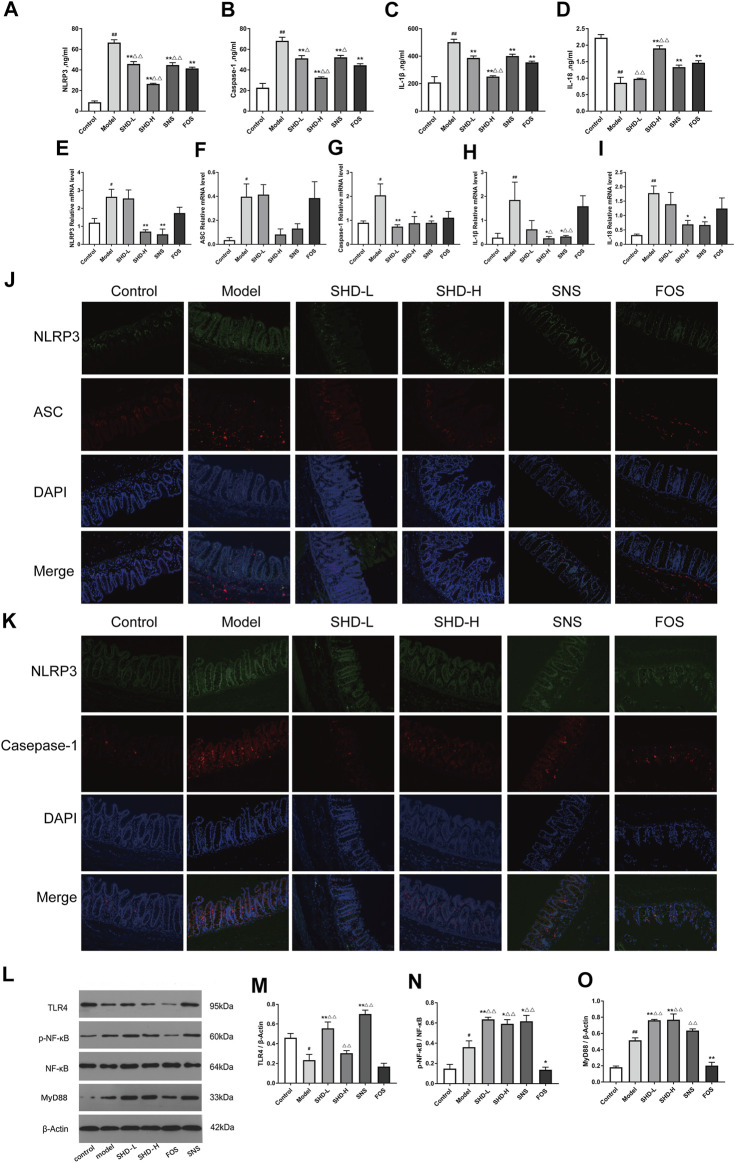
SHD reduces the CUS-induced expression of NOD-like receptor protein 3 (NLRP3), apoptosis-associated speck-like protein containing a CARD domain (ASC), caspase-1, interleukin-1β (IL-1β), and IL-18 in the serum and cecum mucosa and the activation of the Toll-like receptor 4 (TLR4)/nuclear factor (NF)-κB signaling cascades. **(A)** Serum levels of NLRP3 in each group. **(B)** Serum levels of caspase-1 in each group. **(C)** Serum levels of IL-1β in each group. **(D)** Serum levels of IL-18 in each group (*n* = 5). **(E)** Relative mRNA expression levels of NLRP3 in the cecal tissue. **(F)** Relative mRNA expression levels of ASC in the cecal tissue. **(G)** Relative mRNA expression levels of caspase-1 in the cecal tissue. **(H)** Relative mRNA expression levels of IL-1β in the cecal tissue. **(I)** Relative mRNA expression levels of IL-18 in the cecal tissue. The 2^−ΔΔCt^ method was used to analyze the relative gene expression (*n* = 4–5). **(J)** Positive area expression levels of NLRP3 and ASC in the cecal tissue. **(K)** Positive area expression levels of NLRP3 and caspase-1 in the cecal tissue (×200) using immunofluorescence. Scale bar: 50 μm (*n* = 4–6). **(L)** Western blot assays were performed to evaluate the expression levels of TLR4, p-NF-κB, NF-κB, and myeloid differentiation factor 88 (MyD88) in the cecal tissue of the control, model, SHD-L, SHD-H, SNS, and FOS group rats. β-Actin was used as the loading control. **(M)** Quantitative analysis of the relative expression levels of TLR4 in the cecal tissue. **(N)** Quantitative analysis of the relative expression levels of p-NF-κB/NF-κB in the cecal tissue. **(O)** Quantitative analysis of the relative expression levels of MyD88 in the cecal tissue (*n* = 3). The data are expressed as the mean ± SEM. ^*^ *p* < 0.05, ^**^ *p* < 0.01 *vs*. the control group; ^#^ *p* < 0.05, ^##^ *p* < 0.05, *vs*. the CUS model group; ^△^ *p* < 0.05, ^△△^ *p* < 0.05, *vs*. the FOS group.

Disturbances in the intestinal microbiota and immune function of the intestinal mucosa are associated with the development of gastrointestinal dysfunction (Weiss and Hennet, 2017). When the intestinal microbiota is disturbed, the intestinal mucosal barrier can be damaged. This causes the bacteria to come into contact with the submucosal immune cells, which leads to intestinal mucosal NLRP3 inflammation activation (Inserra et al., 2018). We investigated the expression of the NLRP3 inflammasome in the serum using ELISA to determine whether SHD could inhibit NLRP3 inflammation in the rats. As shown in Figures 6A,B (Supplementary Table S9), the serum NLRP3 and caspase-1 levels in the CUS model group were significantly higher than those in the control group (*p* < 0.01). However, after treatment with SHD-L, SHD-H, or SNS, the serum NLRP3 and caspase-1 levels were significantly decreased compared with the model group (*p* < 0.01 and *p* < 0.05, respectively). As shown in Figure 6C (Supplementary Table S9), the serum IL-1β level in the model group was also higher than that of the control group (*p* < 0.01). As shown in Figure 6D, the serum IL-18 level in the model group was lower than that of the control group (*p* < 0.01). After the SHD-H, SNS, and FOS treatments, we observed lower IL-1β levels and higher IL-18 levels in the rats (*p* < 0.01 and *p* < 0.05, respectively). Compared with the FOS group, the SHD-H group showed significantly better efficacy for regulating the release of the NLRP3 inflammasome (*p* < 0.01). Our results are consistent with the findings that cecal microbiota disturbances caused by CUS are related to the NLRP3 inflammasome (Hao et al., 2021), and SHD can regulate the expression of the serum NLRP3 inflammasome, as well as that of the downstream IL-1β and IL-18 in serum.

To evaluate whether SHD influences the NLRP3 inflammasome and exerts its therapeutic effects in cecal tissue, we tested the mRNA levels of the NLRP3 inflammasome and its associated inflammatory cytokines (IL-1β and IL-18) in the cecal tissues using RT-PCR. Figures 6E,G–I (Supplementary Table S10) illustrate that the NLRP3, caspase-1, IL-1β, and IL-18 mRNA levels were significantly increased in the CUS model rats (*p* < 0.01 or *p* < 0.05), but they were downregulated after treatment with SHD-H and SNS. There was not a significant difference in the ASC mRNA levels between either the SHD-H group or the SNS group and the model group (Figure 6F). Furthermore, we determined the protein expression levels of the NLRP3 inflammasome using an immunofluorescence co-localization analysis of the cecal mucosa. As shown in Figures 6J,K (Supplementary Tables S11 and S12), the NLRP3, ASC, and caspase-1 positive areas in the cecal mucosa of the model group were increased compared with the control group (*p* < 0.05). After the SHD-H treatment, the NLRP3 and caspase-1 positive areas were decreased compared with those detected in the model group (*p* < 0.05). However, there was not a significant difference in the NLRP3 and ASC positive areas between the FOS and model groups. These results indicate that SHD-H can inhibit cecal tissue inflammation by regulating the abnormal expression of the NLRP3 inflammasome, as well as that of the downstream inflammatory factors IL-1β and IL-18.

NLRP3 inflammasome activation is closely related to TLR signaling (Jo et al., 2016), which is altered by the host intestinal microbiota during chronic intestinal inflammation (Burgueño and Abreu, 2020). TLR4 activates the transcription factor NF-κB through MyD88, which is critical for the expression of cytokines, chemokines, and costimulatory molecules, such as TNF-α, IL-1β, and IL-18 (Kawai and Akira, 2011; Kawasaki and Kawai, 2014). We examined the expression of proteins associated with the NLRP3 inflammasome in the TLR4 signaling pathway, including TLR4, p-NF-κB, NF-κB, and MyD88, using western blotting. As shown in Figure 6M, the TLR4 expression level in the cecal tissue of the model group was significantly decreased (*p* < 0.05) compared with that of the control group and increased in the SHD-L and SNS groups (*p* < 0.01). Notably, there was not a significant increase in the TLR4 expression after the SHD-H intervention compared with that of the model group. The p-NF-κB/NF-κB and MyD88 protein expression levels were increased in the model group in comparison with the control group (*p* < 0.01 and *p* < 0.05, respectively), and they were further upregulated by the SHD-L and SHD-H interventions (*p* < 0.01 and *p* < 0.05, respectively), while they were downregulated by FOS (*p* < 0.01 and *p* < 0.05, respectively) in comparison with the model group (Figures 6N,O). These results indicate that SHD can modulate the TLR4/NF-κB signaling pathway, which is involved in NLRP3 inflammasome expression and the downstream production of IL-1β and IL-18 in serum and cecal tissue.

### Relationship Between the Cecal Microbiota and NLRP3 Inflammasomes in Cecal Tissue and Serum

**FIGURE 7:**
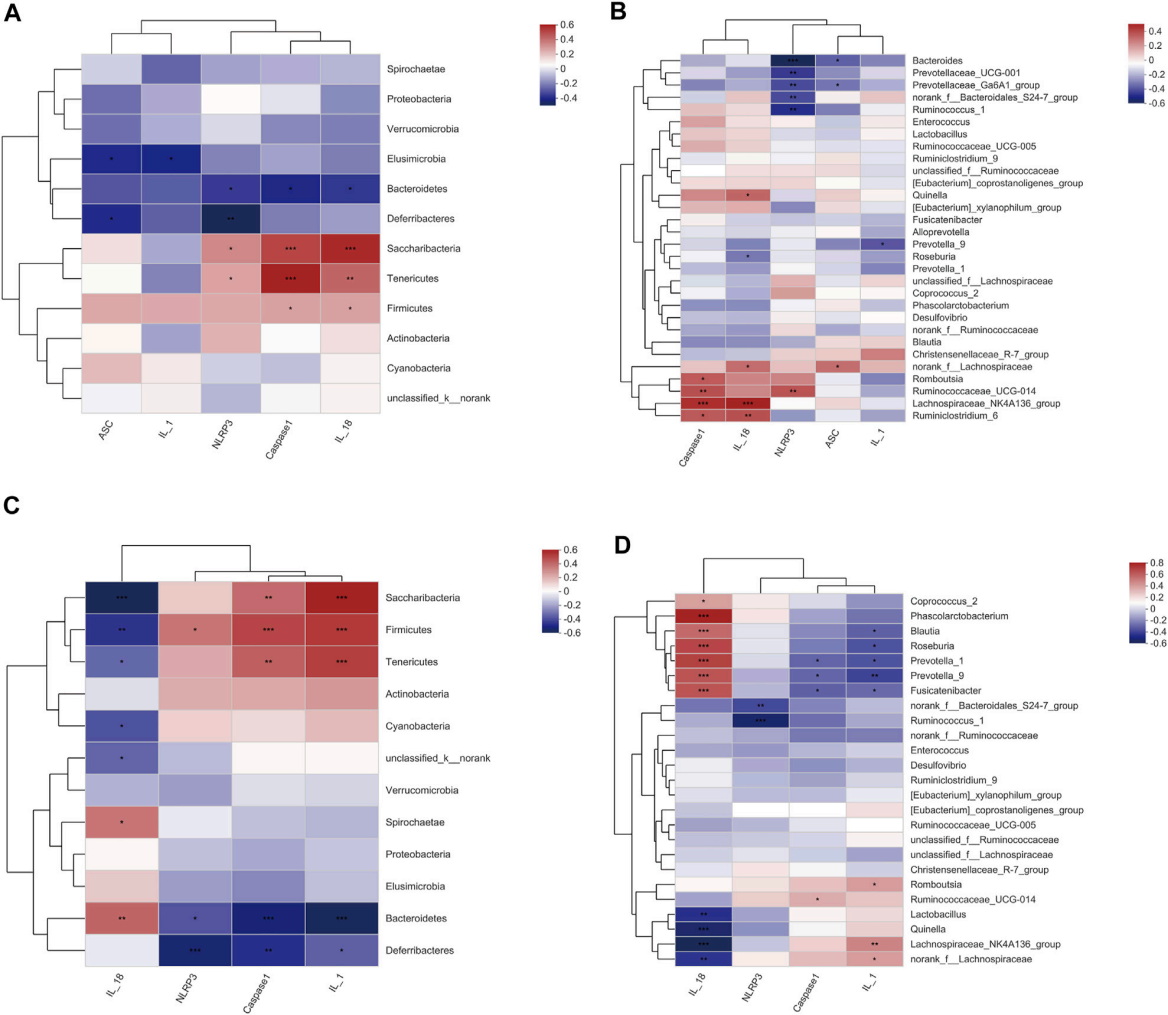
A Spearman’s correlation between the cecal microbiota and NLRP3 inflammasome in the cecum tissue and serum. **(A)** Correlations between the cecal microbiota (phylum level) and NLRP3 inflammasome mRNA in the cecum tissue. **(B)** Correlations between the cecal microbiota (genus level) and NLRP3 inflammasome mRNA in the cecum tissue. **(C)** Correlations between the cecal microbiota (phylum level) and NLRP3 inflammasome in the serum. **(D)** Correlations between the cecal microbiota (genus level) and NLRP3 inflammasome in the serum. *R* value, the closer this value is to 1, the more positive the correlation, while the closer it is to −1, the more negative the correlation. (*p* ≤ 0.001) indicates an extremely significant correlation, (0.001 < *p* ≤ 0.01) indicates a very significant correlation, (0.01 < *p* ≤ 0.05) indicates a significant correlation. The data are expressed as the mean ± SEM, (*n* = 6).

To understand whether SHD affects the NLRP3 inflammasome and the release of downstream IL-1β and IL-18 through regulation of the cecal microbiota, we analyzed the correlations between the biomarker microbiota and the NLRP3 inflammasome in the cecal tissue and serum of the CUS rats. A Spearman’s correlation test was performed on the pooled data set, and the results are shown in Supplementary Table S13. As shown in Figure 7A, at the phylum level, the abundance of *Firmicutes* was positively correlated with the expression of both caspase-1 and IL-18 mRNA (*R* = 0.289, 0.288, *p* = 0.044, 0.045, respectively). The *Bacteroidetes* abundance was negatively correlated with the expression of NLRP3, caspase-1, and IL-18 mRNA (*R* = −0.287, −0.333, −0.289, *p* = 0.045, 0.019, 0.037, respectively). Other phyla, such as *Saccharobacter*, were positively correlated with the expression of NLRP3, caspase-1, and IL-1β mRNA (*R* = 0.346, 0.496, 0.562, *p* = 0.047, 0.00001, 0.0003, respectively); *Tenericutes* was positively correlated with the expression of NLRP3, caspase-1, and IL-1β mRNA (*R* = 0.285, 0.587, 0.424, *p* = 0.015, 0.0003, 0.002, respectively). At genus level (Supplementary Table S14, and Figure 7B), *norank_f_Bacteroidales_S24-7_group* was negatively correlated with the expression of NLRP3 mRNA (*R* = -0.384, *p* = 0.00648), while *Prevotella_9* was negatively correlated with IL-1β mRNA expression (*R* = −0.355, *p* = 0.012). *Lachnospiraceae_NK4A136_group* was positively correlated with the expression of both caspase-1 and IL-18 mRNA (*R* = 0.456, 0.494, respectively, *p* < 0.001 for both), and *Roseburia* was negatively correlated with IL-18 mRNA expression (*R* = −0.289, *p* = 0.0436).

Interestingly, we observed different correlations between the NLRP3 inflammasome expression and the microbiota in the serum; this was particularly true for *Firmicutes*, which was positively associated with NLRP3, caspase-1, and IL-1β (*R* = 0.353, 0.471, 0.505, *p* = 0.01275, 0.00064, 0.00021, respectively), but inversely related to the IL-18 expression (*R* = −0.408, *p* = 0.00362) (Supplementary Tables S15 and S16 and Figures 7C,D). This analysis revealed that the inflammasomes and the inflammation markers IL-1β and IL-18 seem to be strongly influenced by the cecal microbiota, as we observed significant correlations with most of the bacterial groups (i.e., *Firmicutes*, *Bacteroidetes*, *Saccharobacter*, and *Tenericutes*) investigated. However, part of the cecal microbiota was not correlated with the inflammasome, suggesting that some of the bacterial groups in the cecum have different functions that are unlikely to influence inflammation. In summary, these results suggest that SHD regulates cecal microbiota disturbances and inhibits NLRP3 inflammasomes in CUS model rats to ameliorate cecum mucosal injury.

## Discussion

The adaptive stress response can improve an individual’s adaptability and survivability when they are exposed to an onerous situation; however, excessive or chronic stress can markedly disrupt body homeostasis, leading to varying degrees of physiological, psychological, and gastrointestinal dysfunction (Molina-Torres et al., 2019). In this study, we found that the weight gains experienced by the chronic stress model rats were significantly decreased, and they were accompanied by a fecal trait disorder, which is consistent with previous reports (González-Torres and Dos, 2019; Li et al., 2019). In addition, we found that the higher cecum crypt depths and lower cecum crypt densities that we observed under light microscopy might explain the fecal trait disorders. However, physical and psychological comorbidities are often characteristic of chronic stress-related diseases (Averous et al., 2020). In our investigation, after 21 days, the CUS model rats showed various depressive- and anxiety-like behavior changes, as assessed through the SPT, FST, and OFT. We determined that these behavioral changes, which are similar to those that accompany the liver-stomach disharmony syndrome described in traditional Chinese medicine, are characteristics that are inherent to the CUS model rats, suggesting that the model development was successful. We observed that the weight gains, fecal disorders, cecum mucosal injuries, and depressive- and anxiety-like behaviors in the CUS model rats improved significantly with the SHD-H and SNS treatments. Our pharmacodynamic observations revealed that SHD-H could maintain weight gains, improve fecal disorders, and reverse depressive-like behaviors, and these benefits may be associated with the effects of relieving cecum mucosal injury.

Differences in anxiety-related behaviors are commonly reported in mice with altered gut microbiomes, which implicate the gut microbiota in stress (Park et al., 2013). The gut microbiota is mainly dominated by *Firmicutes* and *Bacteroidetes*, although *Proteobacteria*, *Actinobacteria*, *Verrucomicrobia*, and *Fusobacteria* are also present in the colon and cecum (Averous et al., 2020). Subtle alterations in the *Firmicutes*/*Bacteroidetes* ratio have been described in studies of patients with irritable bowel syndrome (correlated with clinical depression and anxiety) (Jeffery et al., 2012); irritable bowel syndrome is also associated with chronic low-grade inflammation (Akiho et al., 2010). Furthermore, in a previous study, a noteworthy decrease in the abundance of *Bacteroidetes* and increase in *Firmicutes* were detected in a chronic unpredictable mild stress model group, and these results were reversed with FOS treatment (Li et al., 2019). Such gut microbiome alterations may be indicators of drug therapy effectiveness (Zhu et al., 2019). The main function of *Firmicutes* in the intestine is to hydrolyze carbohydrates and proteins (Bäckhed et al., 2005). *Bacteroides* mainly act on steroids, polysaccharides, and bile acids, which contribute to protein synthesis and the absorption of polysaccharides (Xu et al., 2003). Commensal *Lactobacillus* species can restore homeostasis in intestinal disorders; thus, they play a protective role against inflammatory diseases. A recent study showed that *L. acidophilus* suppressed proinflammatory cytokines, such as IL-6, TNFα, and IL-1β in colon tissues (Park et al., 2018). The *Lachnospiraceae_NK4A136_group*, *unclassified_f_Lachnospiraceae* belongs to the *Lachnospiraceae* family. *Lachnospiraceae* is one of the most abundant families within the *Firmicutes* phylum, and it is most often associated with the beneficial production of short-chain fatty acids (SCFAs) from complex polysaccharides (Vacca et al., 2020). *Prevotella_9* and *Prevotella_1* both belong to the *Prevotellaceae* family, which is also known to produce SCFAs. SCFA receptors regulate gut primarily through gene expression (e.g., inhibition of the nuclear transcription factor NF-κB, downregulation of pro-inflammatory factors, and inhibition of intestinal inflammation) (Duncan et al., 2009). *Blautia* and *Roseburia* represent the genera most involved in the control of gut inflammatory processes and immune system maturation (La Rosa et al., 2019; Liu et al., 2021). In the present investigation, our findings concerning the gut microbiota are consistent with these reports. We found that the species diversity and richness of the cecal microbiota in the CUS model rats were significantly decreased, while SHD-H increased this diversity and richness, and it did so with better efficacy compared with SNS. We also found that the *Firmicutes*, *Bacteroidetes*, and *Proteobacteria* abundance ratio balance at the phylum level was restored in the CUS model rats after the SHD-H treatment. Specifically, *Lactobacillus* and *Lachnospiraceae_NK4A136_group* in the *Firmicutes* phylum were decreased, while *Roseburia* and *Blautia* were increased; in addition, *Prevotella_9* and *Prevotella_1* were increased in *the Bacteroidetes* phylum. Furthermore, SHD-H had a better effect on regulating the abundance ratio balance of the cecal microbiota than did FOS. We concluded that the CUS-induced cecal microbiota imbalance can be reversed by treatment with SHD-H. This effect may be related to the reduction of intestinal inflammation and release of inflammatory factors.

Dysbiosis of the gut microbiota increases intestinal permeability, leading bacterial metabolites, such as endotoxin (lipopolysaccharide, LPS), to stimulate intestinal mucosal TLRs (Larabi et al., 2020). LPS activates the NLRP3 inflammasome *via* recognition by TLRs, resulting in the production of proinflammatory factors, such as IL-1β and IL-18 (Kelley et al., 2019), and acceleration of the gastrointestinal dysfunction process (Hirota et al., 2011). Caspase-1 is a cysteine protease that cleaves pro-IL-1β and pro-IL-18 into their mature isoforms in response to stressful stimuli such as psychosocial and microbial stress (Lamkanfi et al., 2007). IL-1β and IL-18 are involved in maintaining intestinal integrity and epithelial repair, while aberrant IL-1β and IL-18 signaling can be detrimental to gut function (Nowarski et al., 2015). However, deregulation of pro-inflammatory cytokine production can lead to immunopathology and immune responses. In our investigation, we found that the serum levels of NLRP3, caspase-1, and IL-1β were significantly decreased in the CUS model rats after the SHD, SNS, or FOS treatments, while the IL-18 levels were significantly increased. IL-18, which is a member of the IL-1 family of cytokines, has pro-inflammatory and immune defense enhancing properties (Kaplanski, 2018). The IL-18 level in the model group was lower than that of the control group, suggesting that chronic stress can reduce IL-18 levels, which may be related to declines in immune defense properties. However, the IL-18 levels of the SHD-H, SNS, and FOS groups increased, indicating that these drug treatments can enhance the anti-stress and immune responses. Activation of hypothalamic–pituitary–adrenal axis (HPA axis) and stress-induced changes in the levels of corticosteroids and corticosterone may regulate the inflammatory response (Shuei and Bruno, 2008), making the IL-1β and IL-18 serum level change trends inconsistent. SHD-H and FOS had a significant effect on reversing these changes. However, in the cecal tissue, the relative expression of the NLRP3, ASC, caspase-1, IL-1β, and IL-18 mRNAs was significantly decreased by treatment with either SHD-H or SNS, and both treatment groups showed consistent results in the expression of the positive area. Therefore, we determined that SHD-H may exert its effects on gastrointestinal function regulation by adjusting the intestinal inflammation imbalance, inhibiting the activation of the NLRP3 inflammasome-driven pathways (including ASC and caspase-1), and regulating the abnormal expression of IL-1β and IL-18 in the serum and cecum.

The TLR4 signaling pathway is a crucial upstream regulator of NLRP3 inflammasomes (Silk et al., 2017). The NLRP3 inflammasome activation pathway includes two stages: initiation and activation. The pathogen-associated molecular patterns and damage-associated molecular patterns are mainly identified by TLR4 during the initiation phase. NF-κB is activated by the downstream signaling molecule MyD88. Finally, the expression of NLRP3 and pro-IL-1β is induced. During the activation stage, multiple stimuli promote the assembly of the NLRP3 inflammasome complex and activate the TLR4 signaling pathway (Wang and Hauenstein, 2020). Indeed, our results showed that the TLR4 protein expression level in the cecal tissue of the CUS model rats was markedly downregulated, while the p-NF-κB/NF-κB and MyD88 protein expression levels were upregulated. In addition, the TLR4 protein expression levels in the cecal tissue were increased after treatment with SHD-L, SHD-H, and SNS, and the p-NF-κB/NF-κB and MyD88 proteins were still increased. However, an opposite trend was observed for the FOS. The reason for these results may be that the CUS model is different from the acute inflammatory injury model, and the SHD mechanism may be partially different from that of FOS. In addition, TLR4-related mechanisms can mediate stress-induced adaptations through multi-directional communication between the immune, neural, and endocrine systems during stress (Liu et al., 2014). We can assume that SHD and SNS can increase the protein expression of TLR4 in the cecal tissue to enhance the adaptations to chronic stress. At the chronic stress stage, the sustained activation of the HPA axis demonstrates cortisol resistance, which upregulates NF-κB signaling. NF-κB increases the proinflammation cytokines, which in turn reduce the inflammatory responses (Tian et al., 2014). There is the bidirectional communication between the gut microbiota and the HPA axis, which controls various body processes in response to stress (Farzi et al., 2018). This may explain the continued increases in the p-NF-κB/NF-κB and MyD88 protein levels after the SHD and SNS treatments, both of which may modulate the network regulation of the cecal microbiota and HPA axis to affect the TLR4/NF-κB signaling pathway. Therefore, we determined that NLRP3 inflammasome can be adjusted by treatment with SHD or SNS through multiple pathways and targets, both in the serum and cecal tissue, and the TLR4/NF-κB signaling pathway may be one of those pathways.

Moreover, the extent to which the NLRP3 inflammasome is activated appears to be greatly influenced by the composition and function of the gut microbiome (Wong et al., 2016). Interestingly, upregulation of the NLRP3 inflammasome pathway disrupts the gut microbiota by influencing pro-inflammatory signaling, which modulates the gut microbiota composition by increasing the representation of bacterial clades conducive to pro-inflammatory signaling (e.g., *Proteobacteria*, *Allistipes*, *Prevotella*, *Oscillibacter*, *Actinobacteria*) and decreasing the representation of bacterial clades conducive to anti-inflammatory signaling (e.g., *Firmicutes*, *Faecalibacterium*, *Lachnospiraceae*, and *Bacteroidetes*) (Inserra et al., 2018). In addition, *Proteus mirabilis* (a *Proteobacteria* phylum member) triggers NLRP3 activation and IL-1β production (Seo et al., 2015). Conversely, the abundance of *Lactobacillus*, a genus involved in inflammasome activation via caspase-1-mediated IL-1β production, was increased (Zheng et al., 2016).

FOS can regulate intestinal microbiota disturbances, inhibit the expression of the NLRP3 inflammasome and the intestinal mucosal pre-inflammatory cytokines IL-1α and IL-1β to affect gastrointestinal function (Ferenczi et al., 2016), and improve mental and emotional abnormalities (Savignac et al., 2016). This is why FOS was used in the positive control group in this experiment, as these FOS characteristics aided in clarifying the SHD mechanism. Li et al. reported that moxibustion and probiotic treatments elicited similar effects by regulating intestinal host-microbial homeostasis and the expression of NLRP3 inflammasome-related factors (Li et al., 2021). However, little is known about how SHD regulates the intestinal microbiota and NLRP3 inflammasome signaling. In this study, our correlation analysis showed that *Firmicutes*, *Bacteroidetes*, *Saccharobacter*, *Tenericutes* (phylum level), *norank_f_Bacteroidales_S24-7_group*, *Prevotella_9*, *Lachnospiraceae_NK4A136_group*, and *Roseburia* (genus level) are biomarker microbiota, which are highly correlated with the NLRP3 inflammasome and downstream inflammatory factors (IL-1β, IL-18) in CUS model rats. We considered that SHD could regulate cecal microbiota and that they are highly correlated with the NLRP3 inflammasome and downstream inflammatory factors (IL-1β and IL-18) in CUS model rats. Therefore, we determined that SHD could regulate cecal microbiota to inhibit CUS-induced NLRP3 inflammasome activation.

Notably, compared with SNS, SHD-H provided superior efficacy in terms of maintaining weight gain, improving fecal traits, alleviating cecum mucosal injury, inhibiting NLRP3 inflammasome expression, and regulating the diversity, abundance, and proportion of the cecal microbiota. Although both SHD-H and FOS can remodel cecal microbiota dysbiosis, SHD-H has more obvious advantages in maintaining weight gain, improving cecum mucosal injury, reversing depressive- and anxiety-like behaviors, and regulating NLRP3 inflammasome expression in CUS model rats. Therefore, SHD-H provides an advanced therapeutic effect for the treatment of depression and gastrointestinal dysfunction caused by chronic stress.

## Conclusion

The results of our study suggest that SHD can alleviate cecum mucosal injury and improve depressive- and anxiety-like behaviors by regulating the cecal microbiota and inhibiting the excessive activation of the NLRP3 inflammasome in the cecum and serum. Our work reveals the pharmacological mechanisms of SHD and provides a new therapeutic method for the prevention and treatment of psychosomatic comorbidities related to intestinal function disorders and depressive- and anxiety-like behaviors. SHD contains 10 traditional Chinese herbs, each of which contains complex chemical compounds. Therefore, it will be necessary to clarify which compounds in the SHD formulation have beneficial effects on stress-related diseases and what their precise targets are. In addition, because SHD metabolism *in vivo* is complicated, further studies are needed to identify the gut-derived metabolites and other metabolomic products that are involved in the relationship between gut microbiota dysbiosis and inflammasomes. Nevertheless, this study is far from sufficient, more investigations are needed, and a series of follow-up studies are underway in our laboratory.

## Data Availability

The data presented in the study are deposited in the (online) repository, accession number NCBI SRP324274.
