# Supplementary material for: Shugan Hewei Decoction Alleviates Cecum Mucosal Injury and Improves Depressive- and Anxiety-Like Behaviors in Chronic Stress Model Rats by Regulating Cecal Microbiota and Inhibiting NLRP3 Inflammasome
Source: Front Pharmacol. 2021 Dec 20;12:766474. doi: 10.3389/fphar.2021.766474 (PMC8721152; doi:10.3389/fphar.2021.766474)
Supplement: Supplementary file 1 [file DataSheet3.ZIP › Supplementary_Material-original data1/FIGURE1/Figure 1.pptx]

## Slide 1
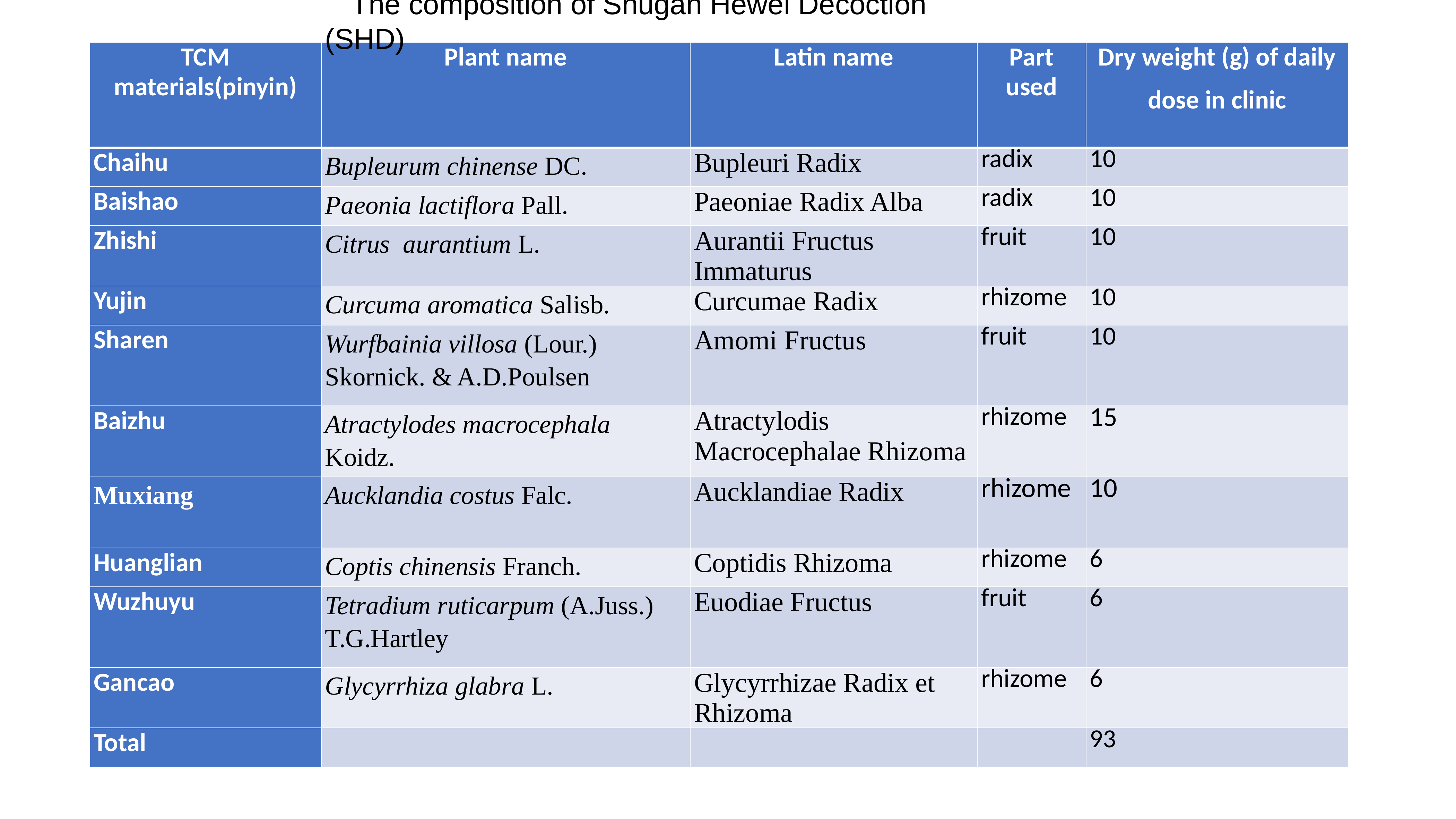

The composition of Shugan Hewei Decoction (SHD)
| TCM materials(pinyin) | Plant name | Latin name | Part used | Dry weight (g) of daily dose in clinic |
| --- | --- | --- | --- | --- |
| Chaihu | Bupleurum chinense DC. | Bupleuri Radix | radix | 10 |
| Baishao | Paeonia lactiflora Pall. | Paeoniae Radix Alba | radix | 10 |
| Zhishi | Citrus aurantium L. | Aurantii Fructus Immaturus | fruit | 10 |
| Yujin | Curcuma aromatica Salisb. | Curcumae Radix | rhizome | 10 |
| Sharen | Wurfbainia villosa (Lour.) Skornick. & A.D.Poulsen | Amomi Fructus | fruit | 10 |
| Baizhu | Atractylodes macrocephala Koidz. | Atractylodis Macrocephalae Rhizoma | rhizome | 15 |
| Muxiang | Aucklandia costus Falc. | Aucklandiae Radix | rhizome | 10 |
| Huanglian | Coptis chinensis Franch. | Coptidis Rhizoma | rhizome | 6 |
| Wuzhuyu | Tetradium ruticarpum (A.Juss.) T.G.Hartley | Euodiae Fructus | fruit | 6 |
| Gancao | Glycyrrhiza glabra L. | Glycyrrhizae Radix et Rhizoma | rhizome | 6 |
| Total | | | | 93 |
