# Supplementary figures and images for: Shugan Hewei Decoction Alleviates Cecum Mucosal Injury and Improves Depressive- and Anxiety-Like Behaviors in Chronic Stress Model Rats by Regulating Cecal Microbiota and Inhibiting NLRP3 Inflammasome (part 1 of 2)
Source: Front Pharmacol. 2021 Dec 20;12:766474. doi: 10.3389/fphar.2021.766474 (PMC8721152; doi:10.3389/fphar.2021.766474)

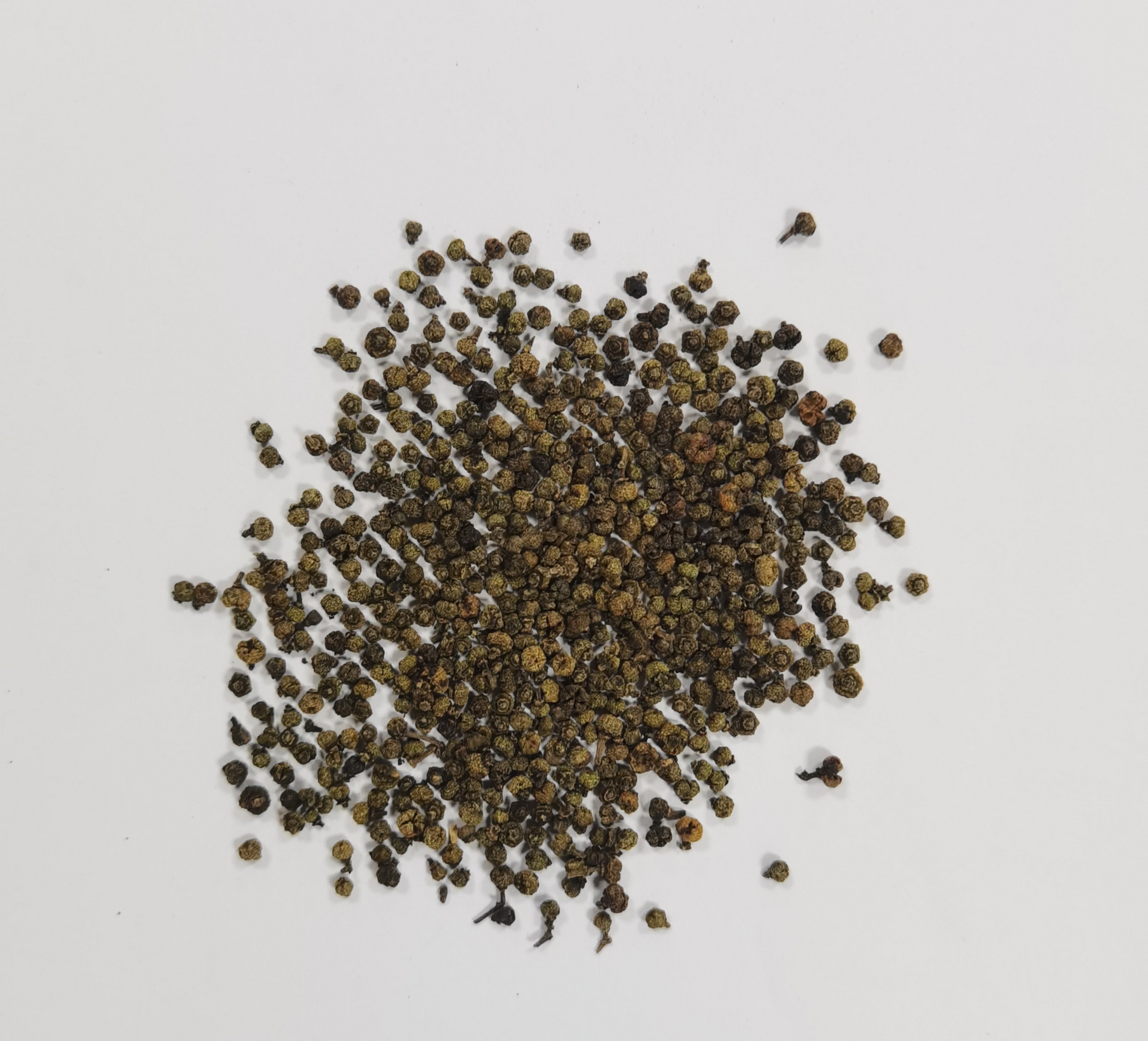

Supplement: Supplementary file 1 [file DataSheet3.ZIP › Supplementary_Material-original data1/FIGURE1/SHD/吴茱萸.jpg]

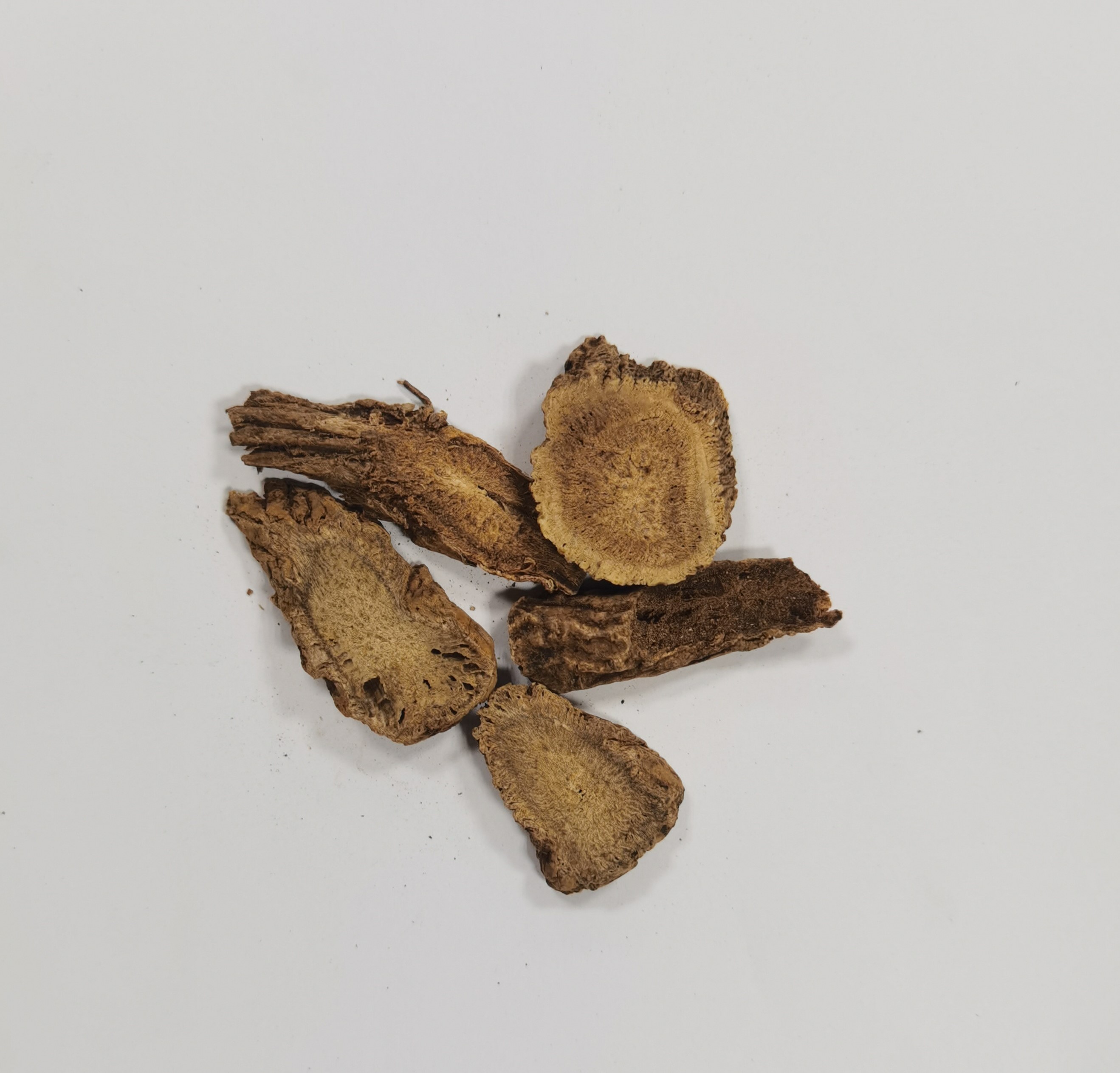

Supplement: Supplementary file 1 [file DataSheet3.ZIP › Supplementary_Material-original data1/FIGURE1/SHD/木香.jpg]

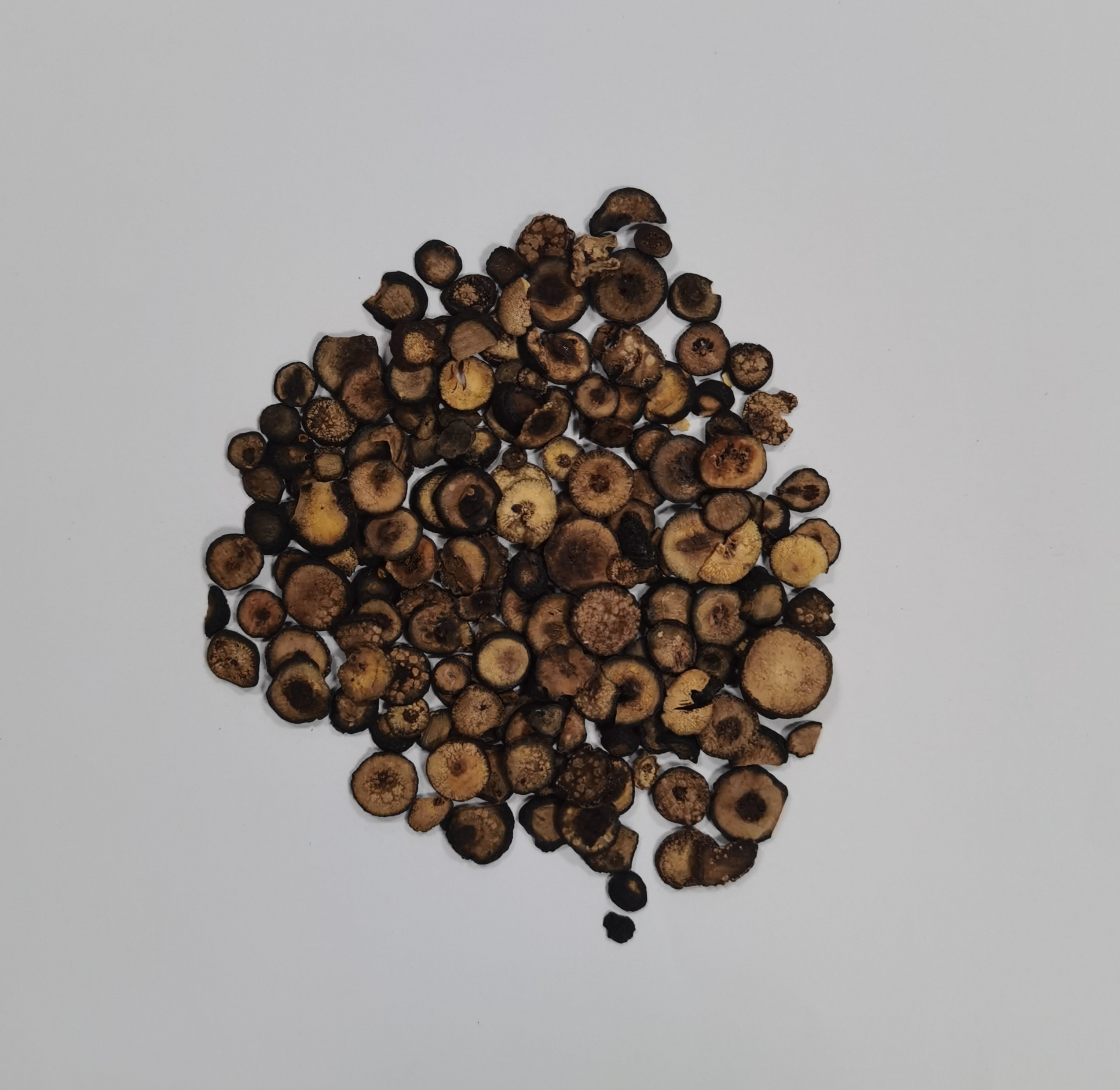

Supplement: Supplementary file 1 [file DataSheet3.ZIP › Supplementary_Material-original data1/FIGURE1/SHD/枳实.jpg]

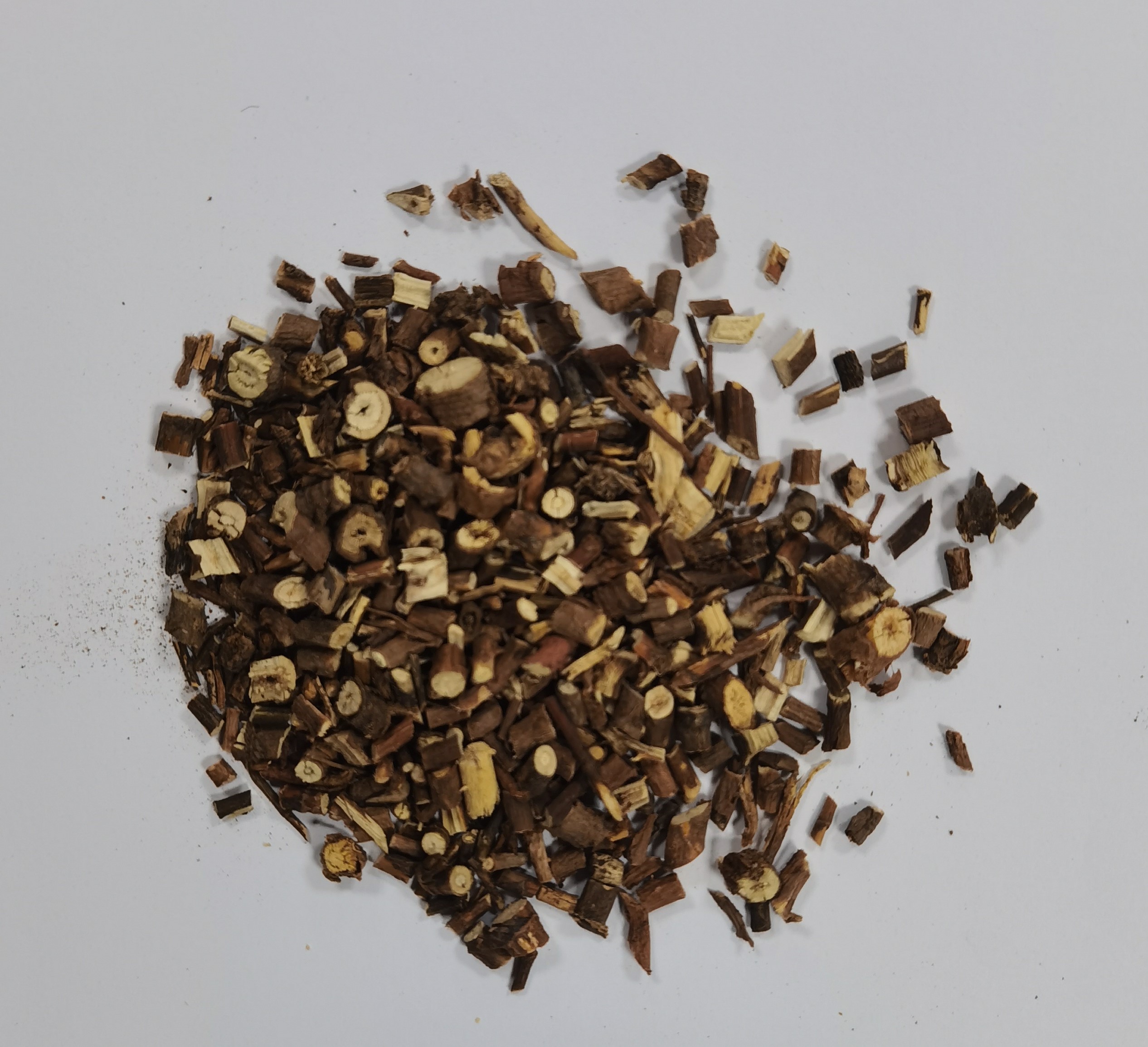

Supplement: Supplementary file 1 [file DataSheet3.ZIP › Supplementary_Material-original data1/FIGURE1/SHD/柴胡.jpg]

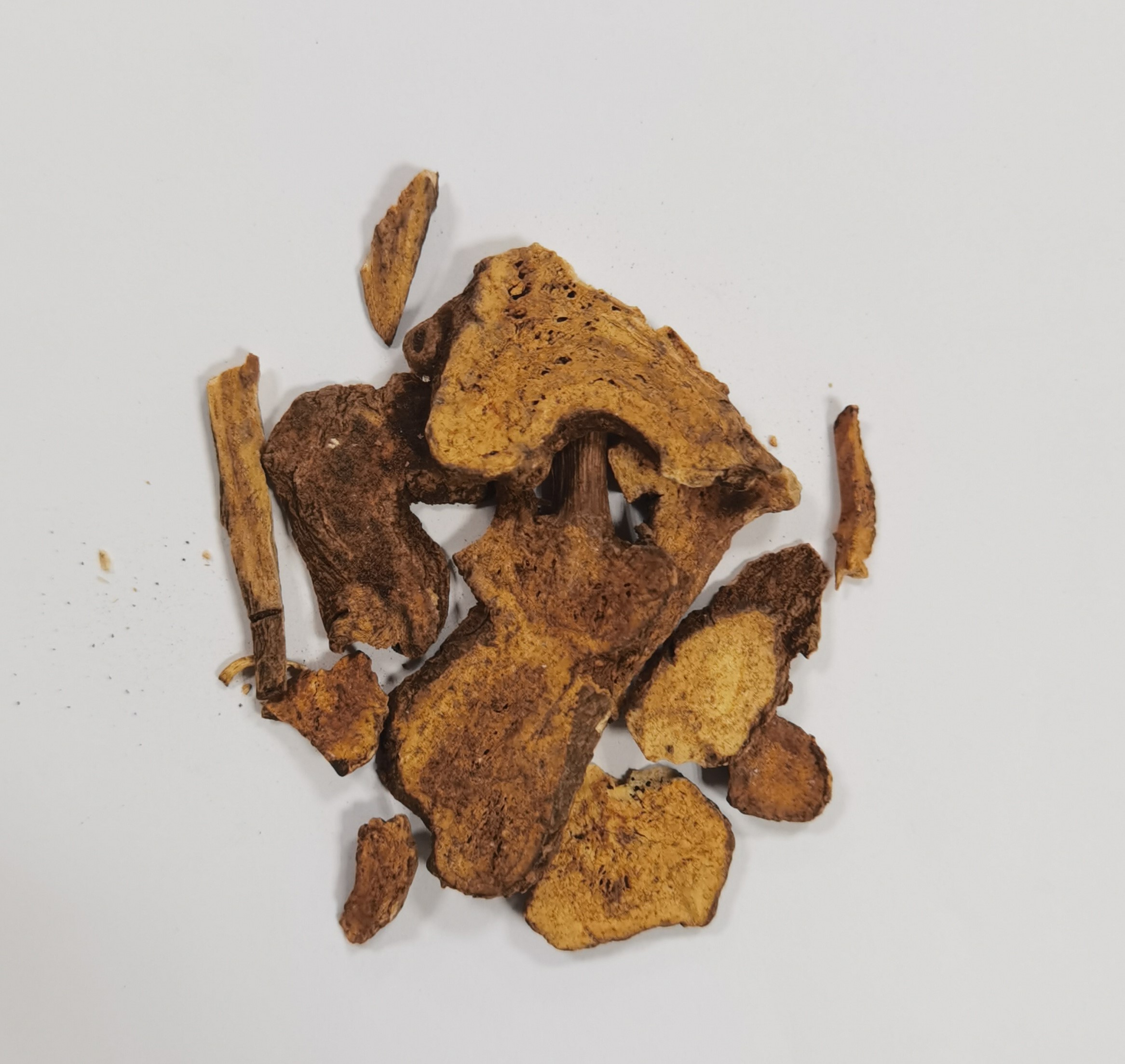

Supplement: Supplementary file 1 [file DataSheet3.ZIP › Supplementary_Material-original data1/FIGURE1/SHD/炒白术.jpg]

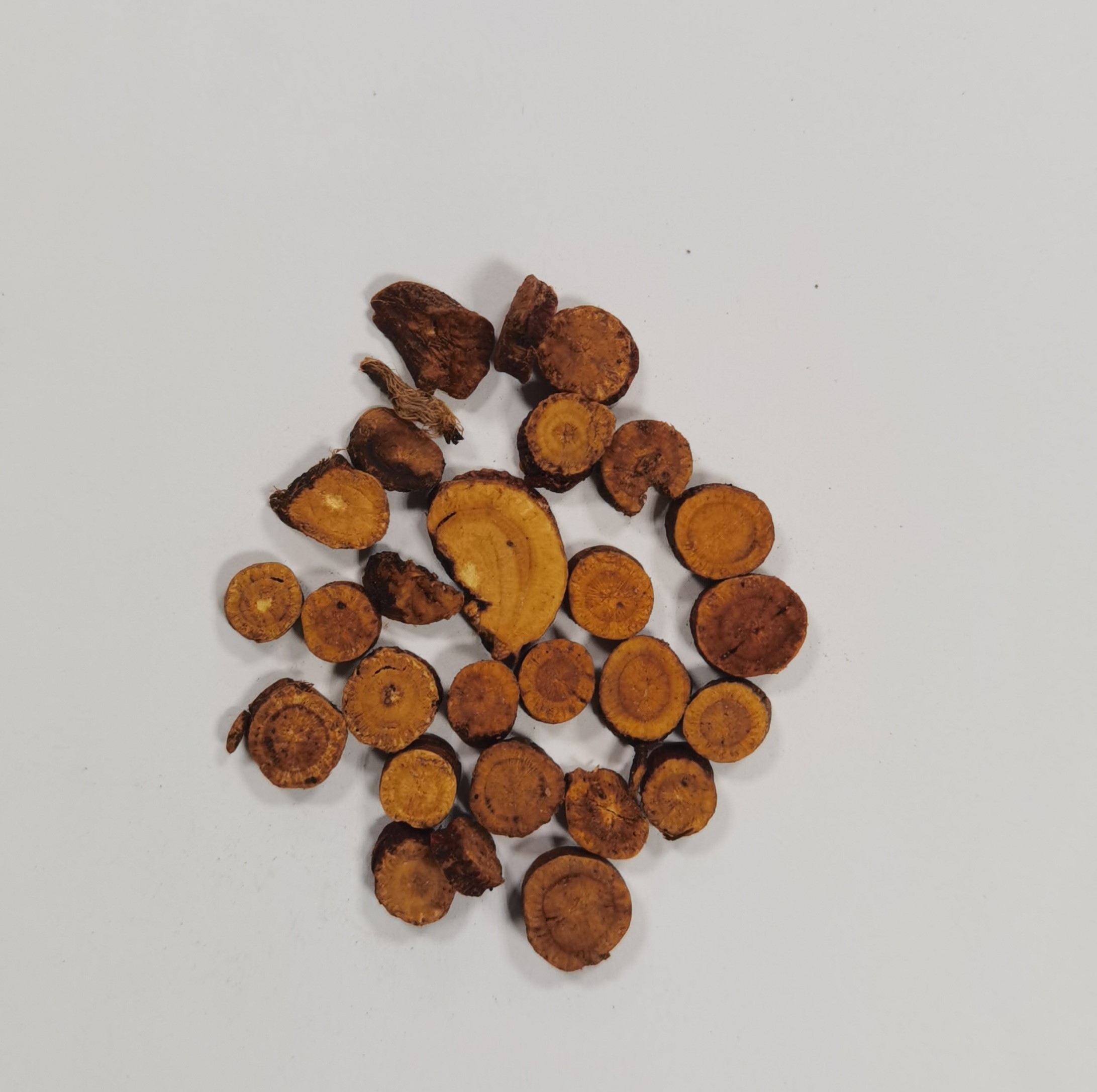

Supplement: Supplementary file 1 [file DataSheet3.ZIP › Supplementary_Material-original data1/FIGURE1/SHD/炙甘草.jpg]

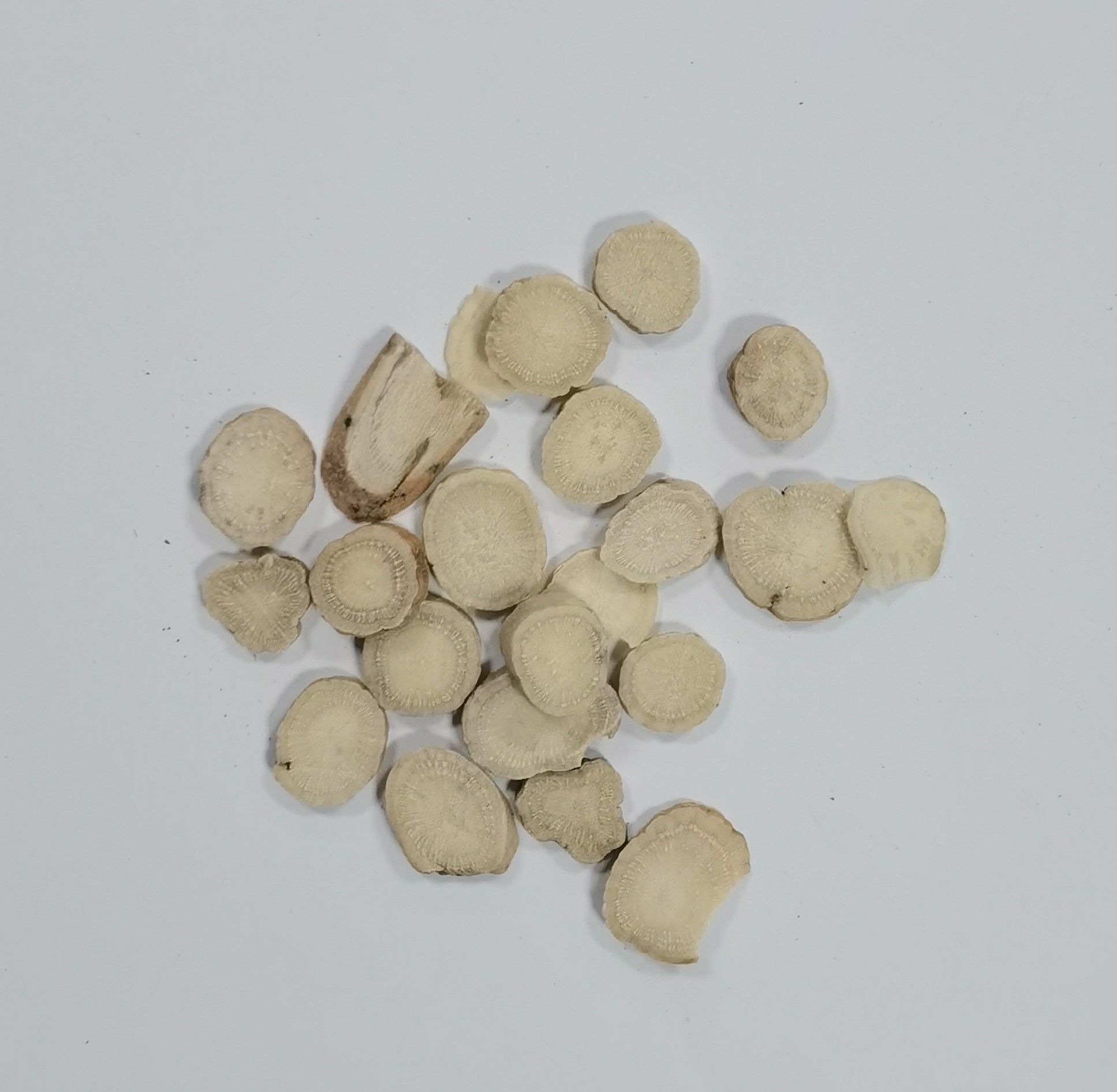

Supplement: Supplementary file 1 [file DataSheet3.ZIP › Supplementary_Material-original data1/FIGURE1/SHD/白芍.jpg]

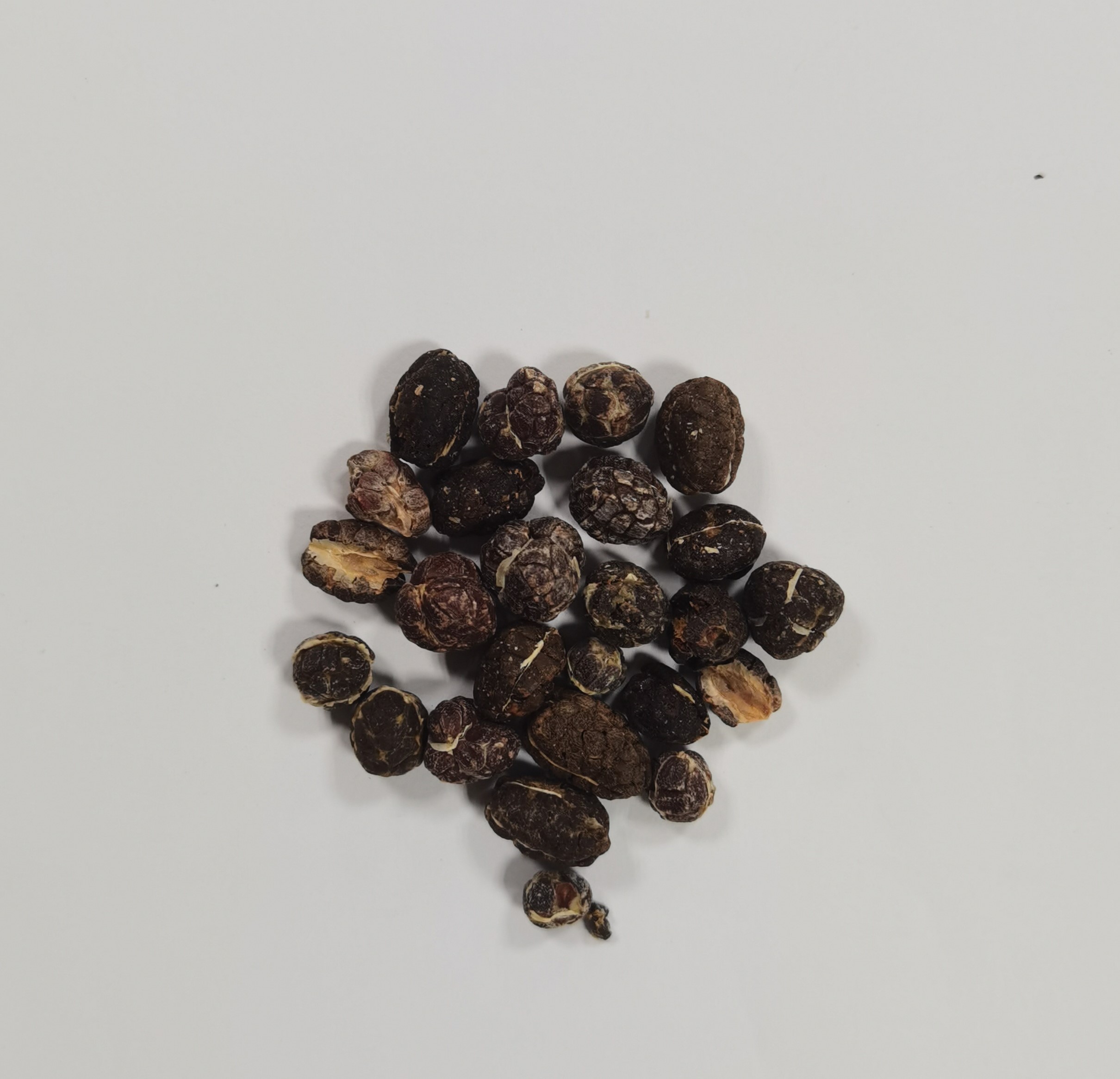

Supplement: Supplementary file 1 [file DataSheet3.ZIP › Supplementary_Material-original data1/FIGURE1/SHD/砂仁.jpg]

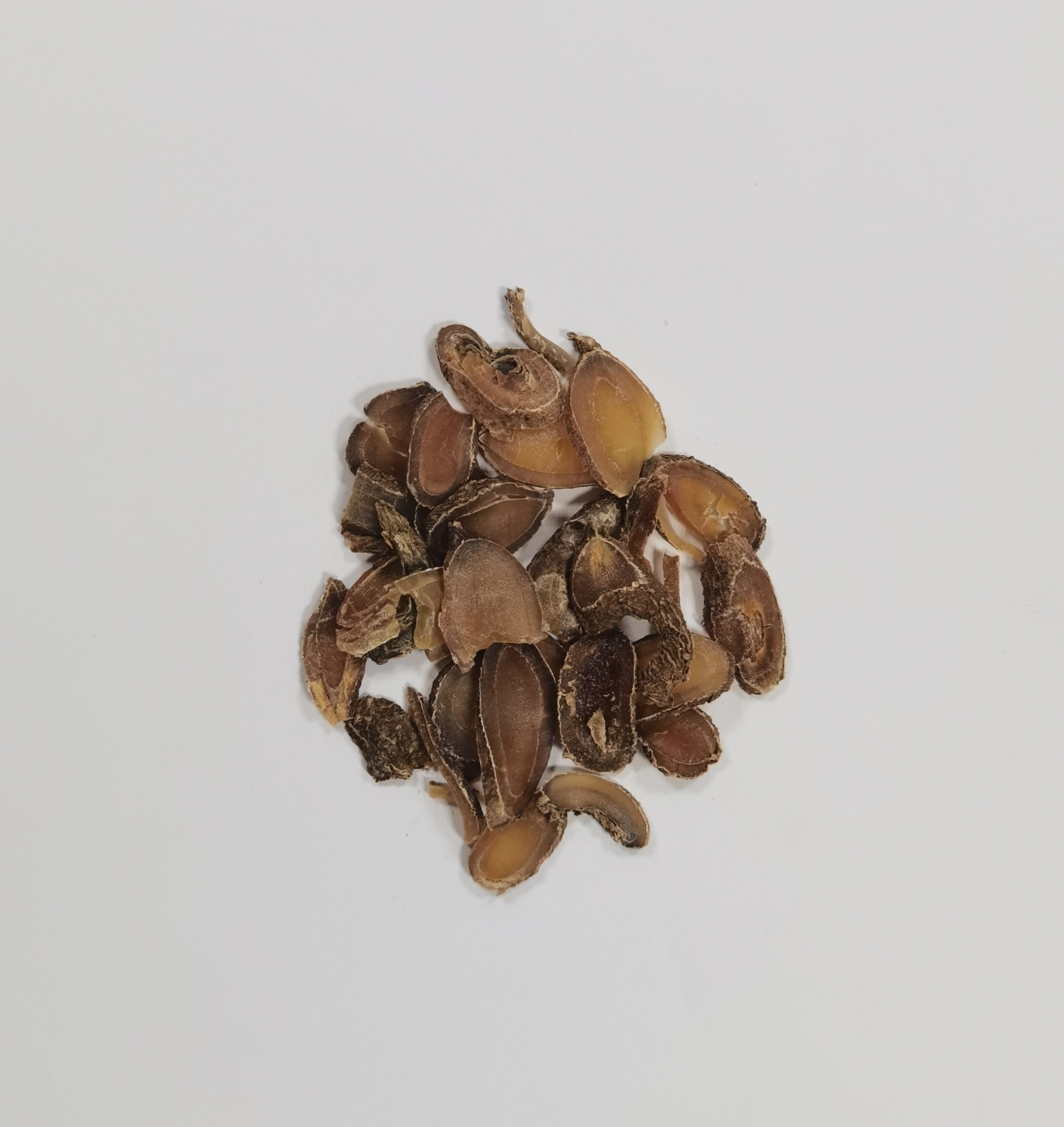

Supplement: Supplementary file 1 [file DataSheet3.ZIP › Supplementary_Material-original data1/FIGURE1/SHD/郁金.jpg]

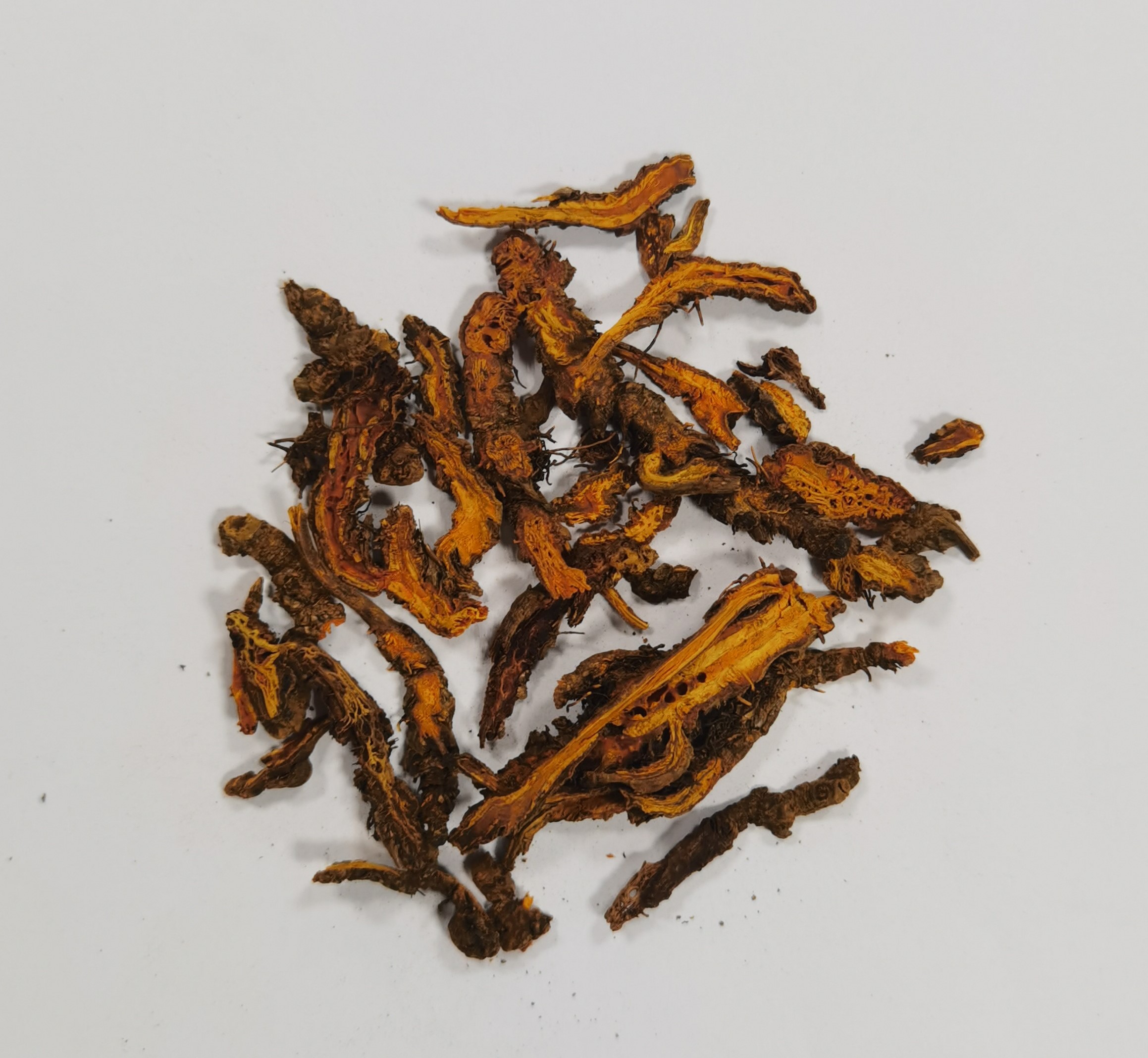

Supplement: Supplementary file 1 [file DataSheet3.ZIP › Supplementary_Material-original data1/FIGURE1/SHD/黄连.jpg]

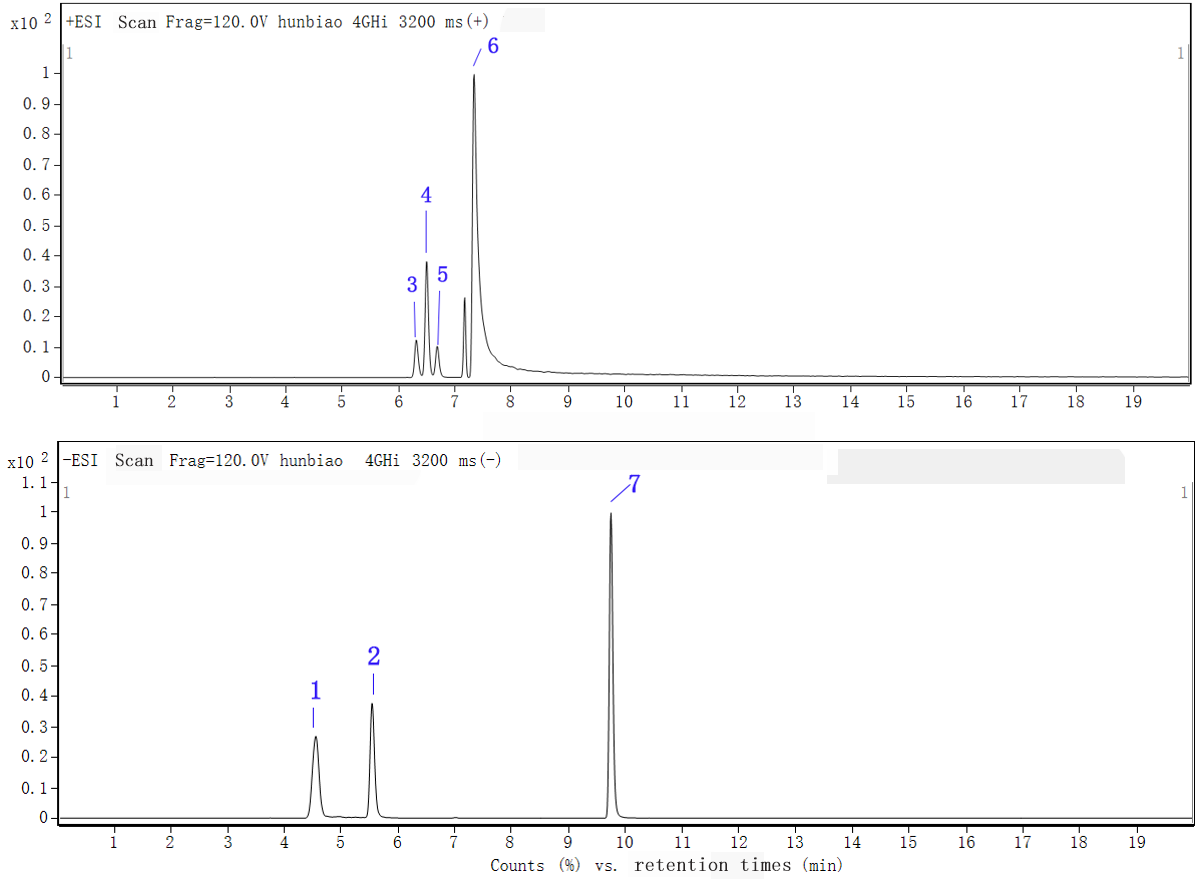

Supplement: Supplementary file 1 [file DataSheet3.ZIP › Supplementary_Material-original data1/FIGURE3/Figure 3(E) TIC of standard substances in positive mode. (F) TIC of standard substances in negative mode.tif]

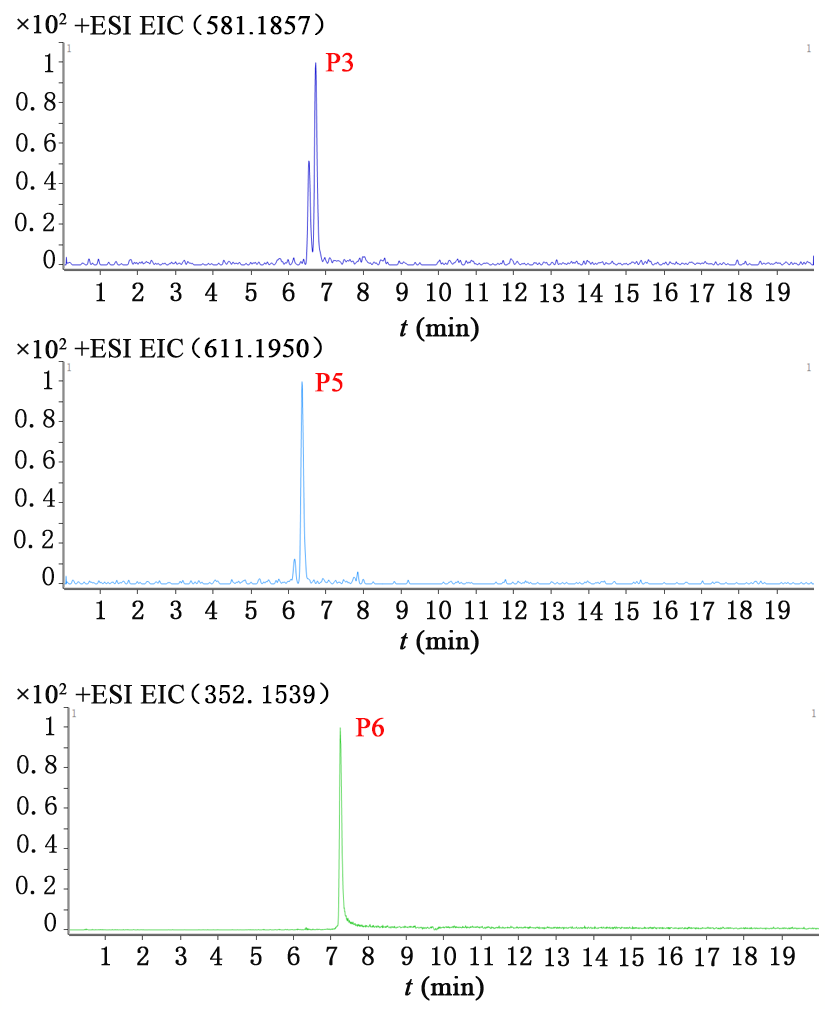

Supplement: Supplementary file 1 [file DataSheet3.ZIP › Supplementary_Material-original data1/FIGURE3/Figure 3(G) The ion intensity of three prototype components identified from SHD-Containing serum.tif]

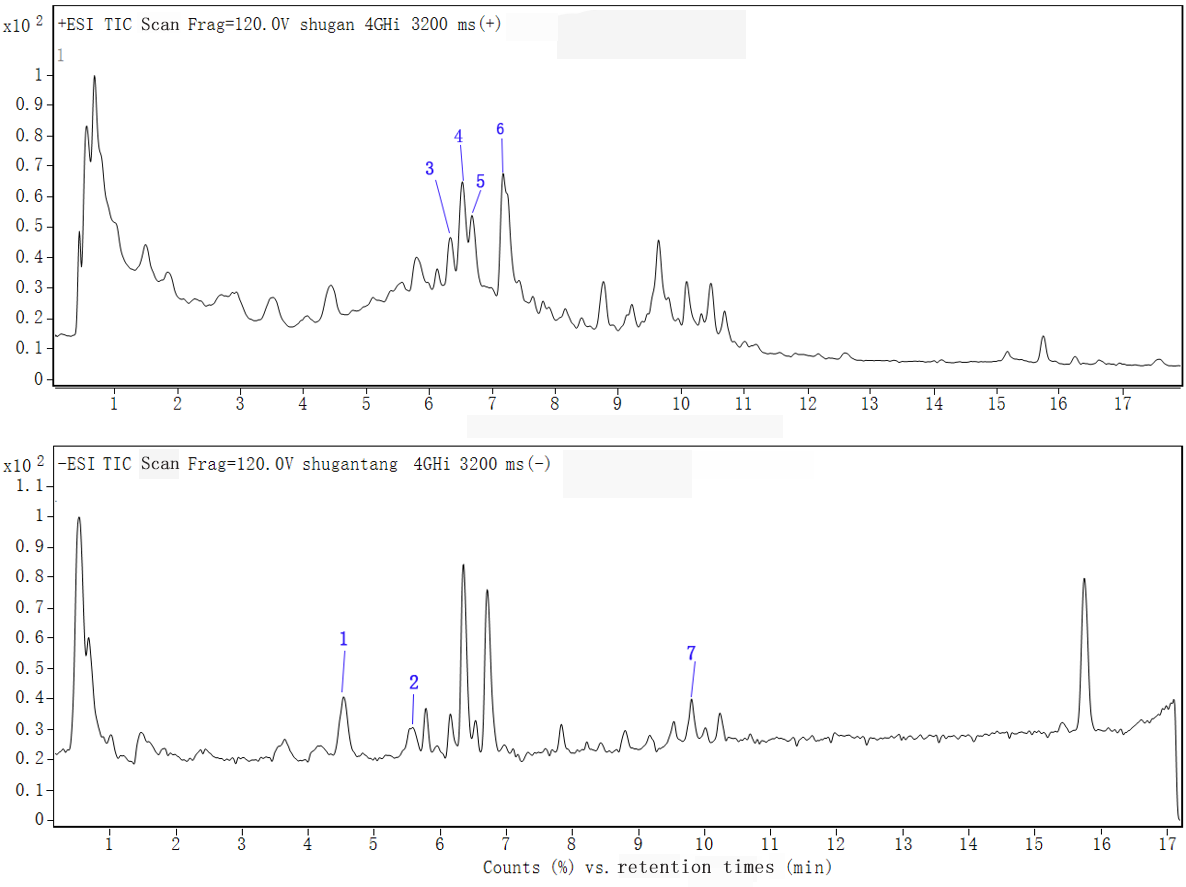

Supplement: Supplementary file 1 [file DataSheet3.ZIP › Supplementary_Material-original data1/FIGURE3/Figures 3(A) TIC of SHD in positive mode. (B) TIC of SHD in negative mode.tif]

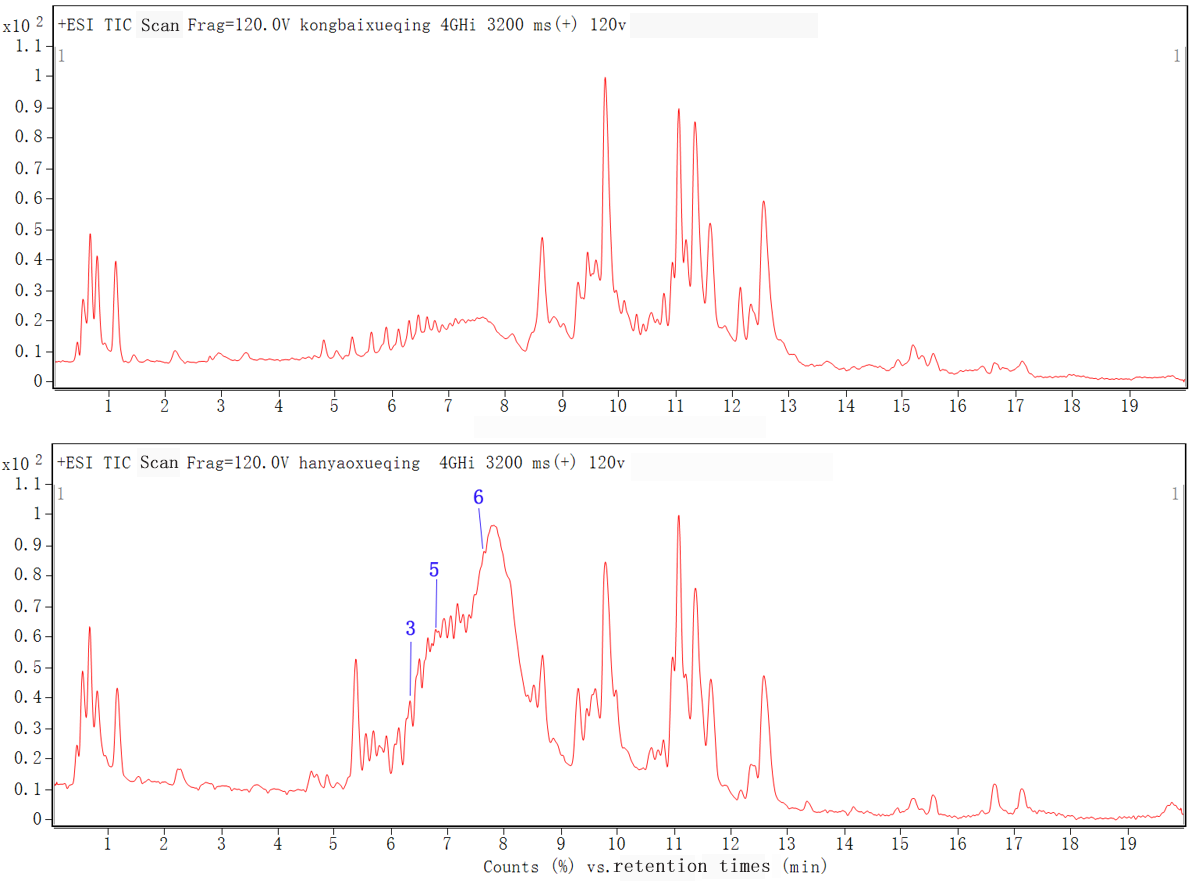

Supplement: Supplementary file 1 [file DataSheet3.ZIP › Supplementary_Material-original data1/FIGURE3/Figures 3(C) TIC of Control serum in positive mode. (D)TIC of SHD-Containing serum in positive mode.tif]

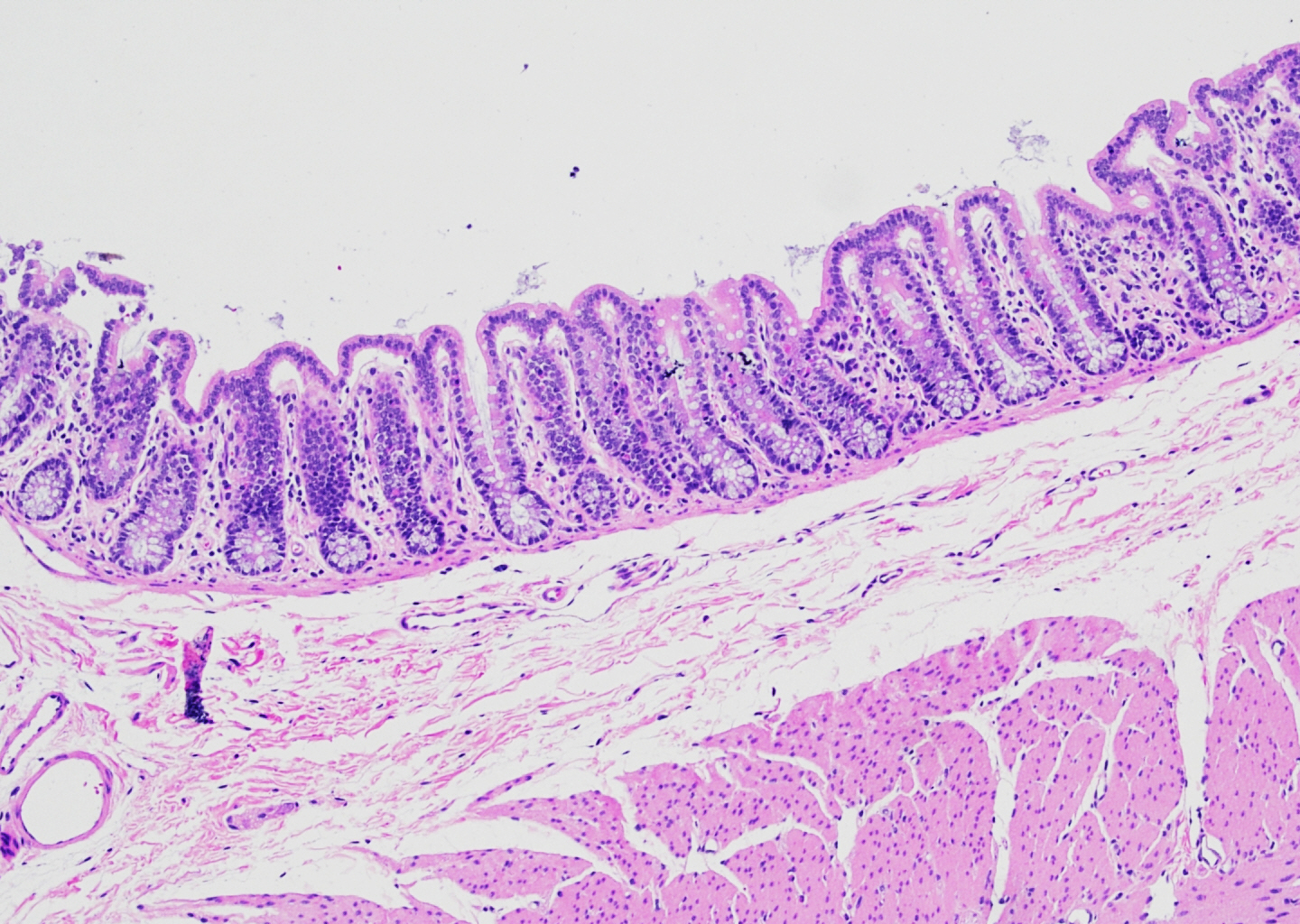

Supplement: Supplementary file 1 [file DataSheet3.ZIP › Supplementary_Material-original data1/FIGURE4/Figure4 A-control×100/A-control-CM1-9 100-3.jpg]

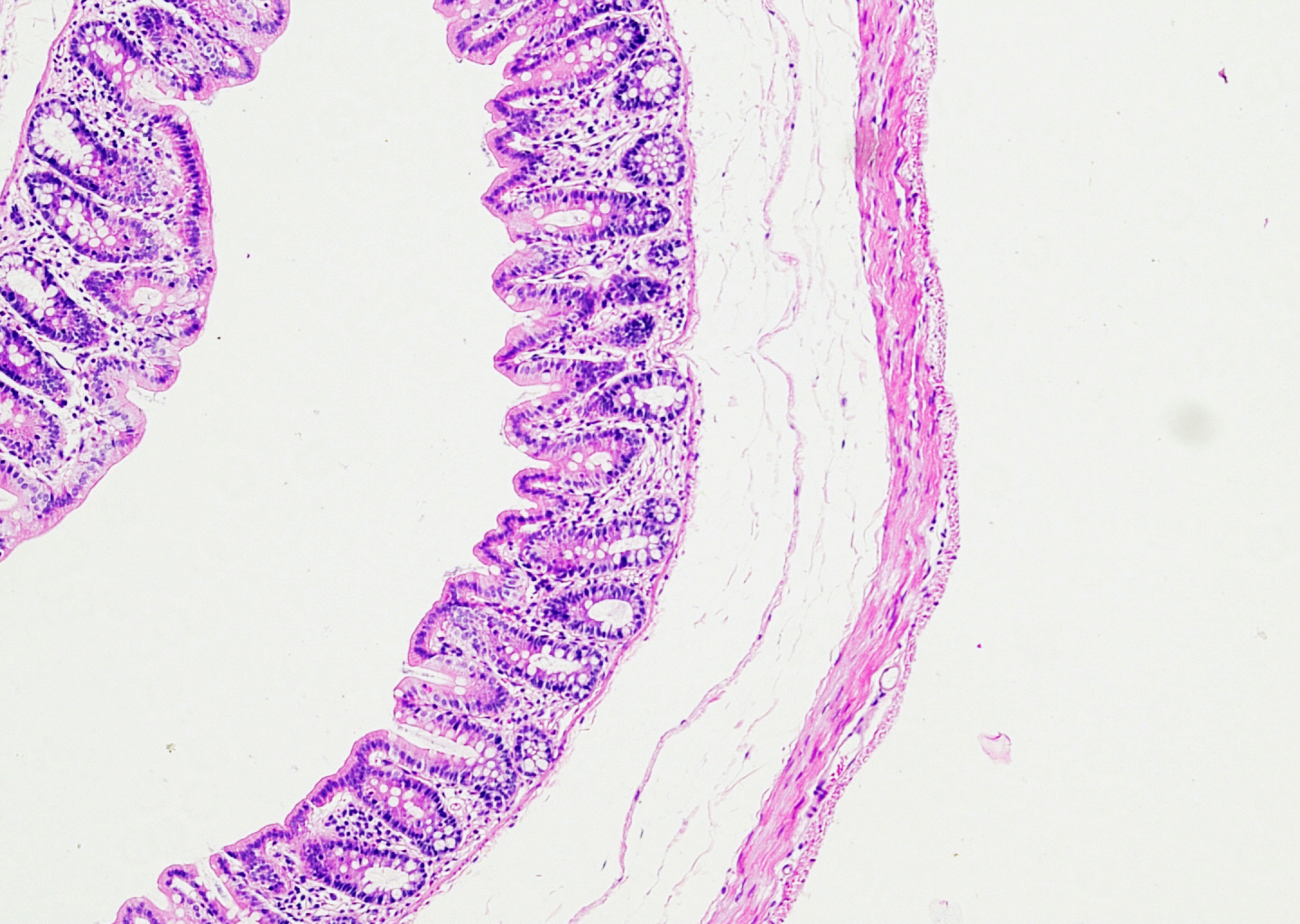

Supplement: Supplementary file 1 [file DataSheet3.ZIP › Supplementary_Material-original data1/FIGURE4/Figure4 A-FOS×100/FOS-Y1-1 100-2.jpg]

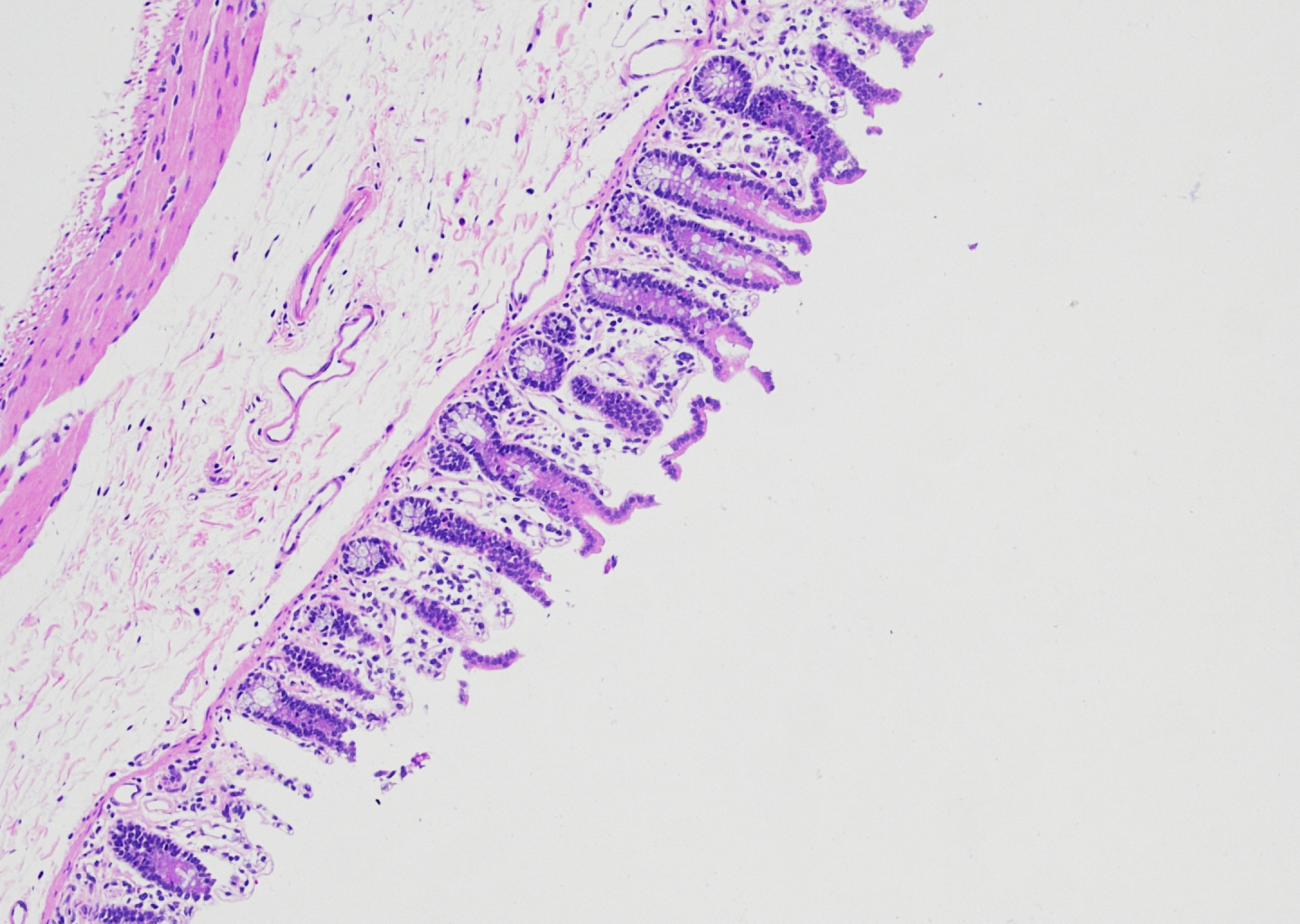

Supplement: Supplementary file 1 [file DataSheet3.ZIP › Supplementary_Material-original data1/FIGURE4/Figure4 A-model×100/B-model-CM2-13 100-3.jpg]

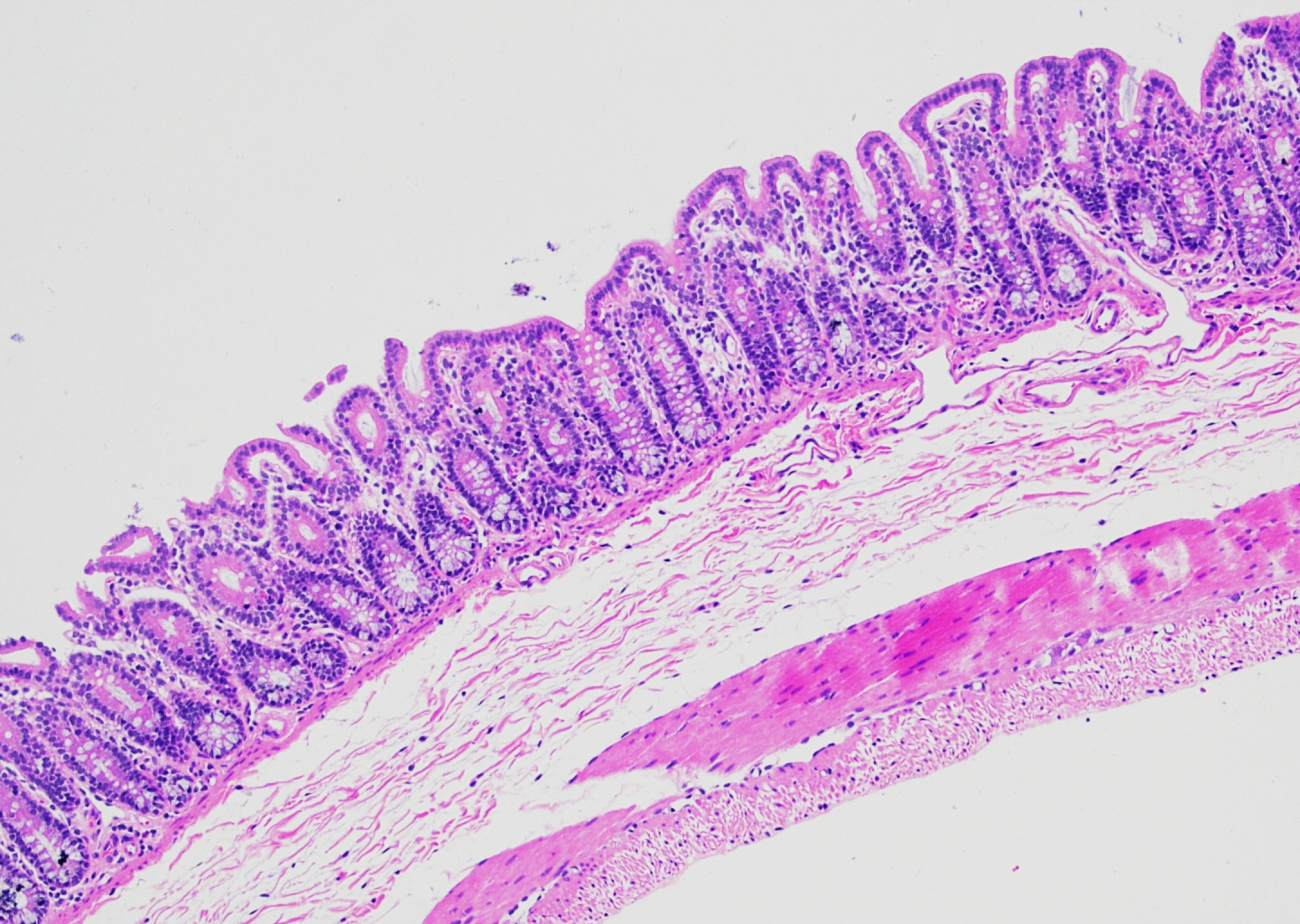

Supplement: Supplementary file 1 [file DataSheet3.ZIP › Supplementary_Material-original data1/FIGURE4/Figure4 A-SHD-H×100/SHD-H-200%-CM4-10 100-3.jpg]

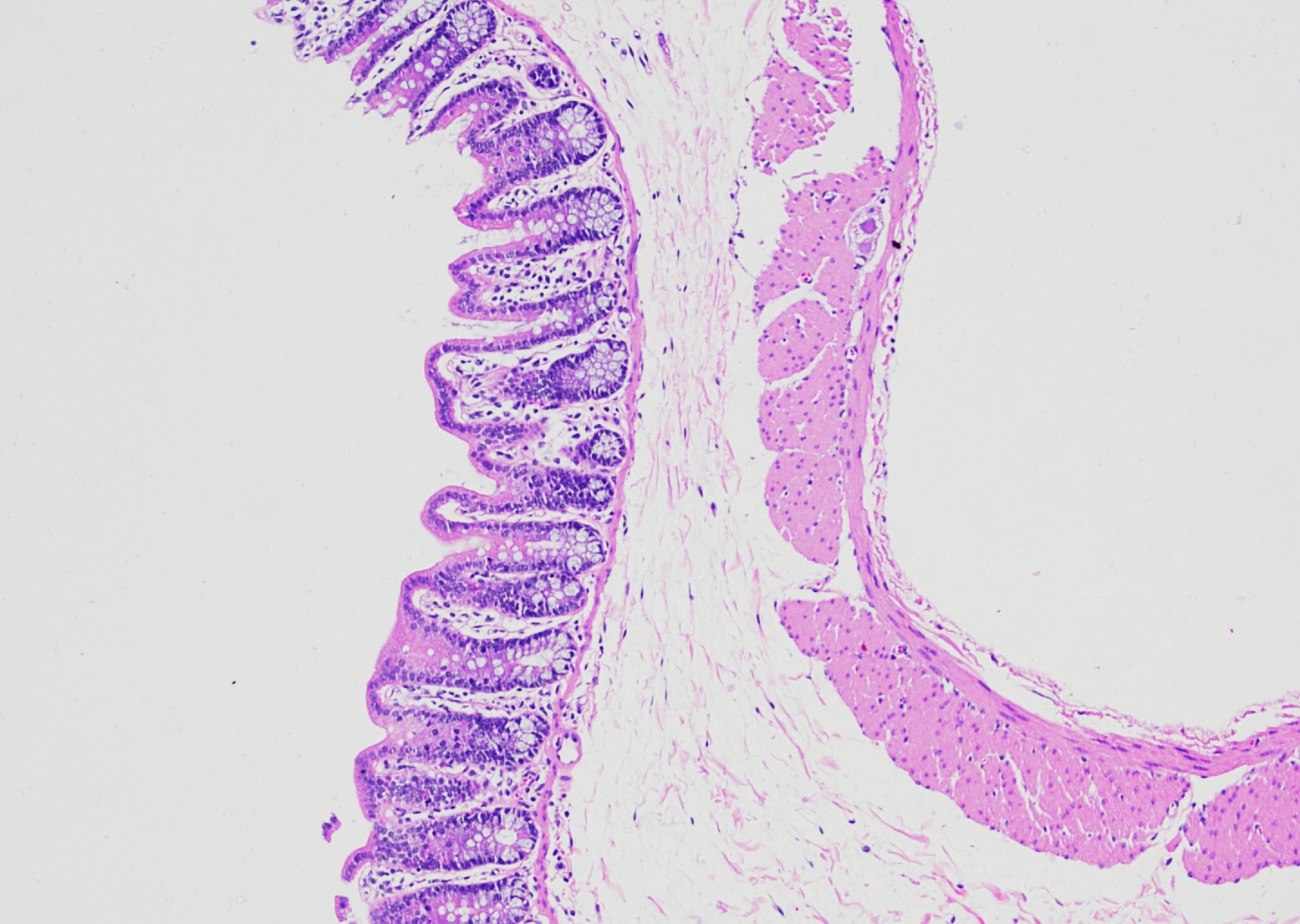

Supplement: Supplementary file 1 [file DataSheet3.ZIP › Supplementary_Material-original data1/FIGURE4/Figure4 A-SHD-L×100/SHD-L-100%-CM3-11 100-3.jpg]

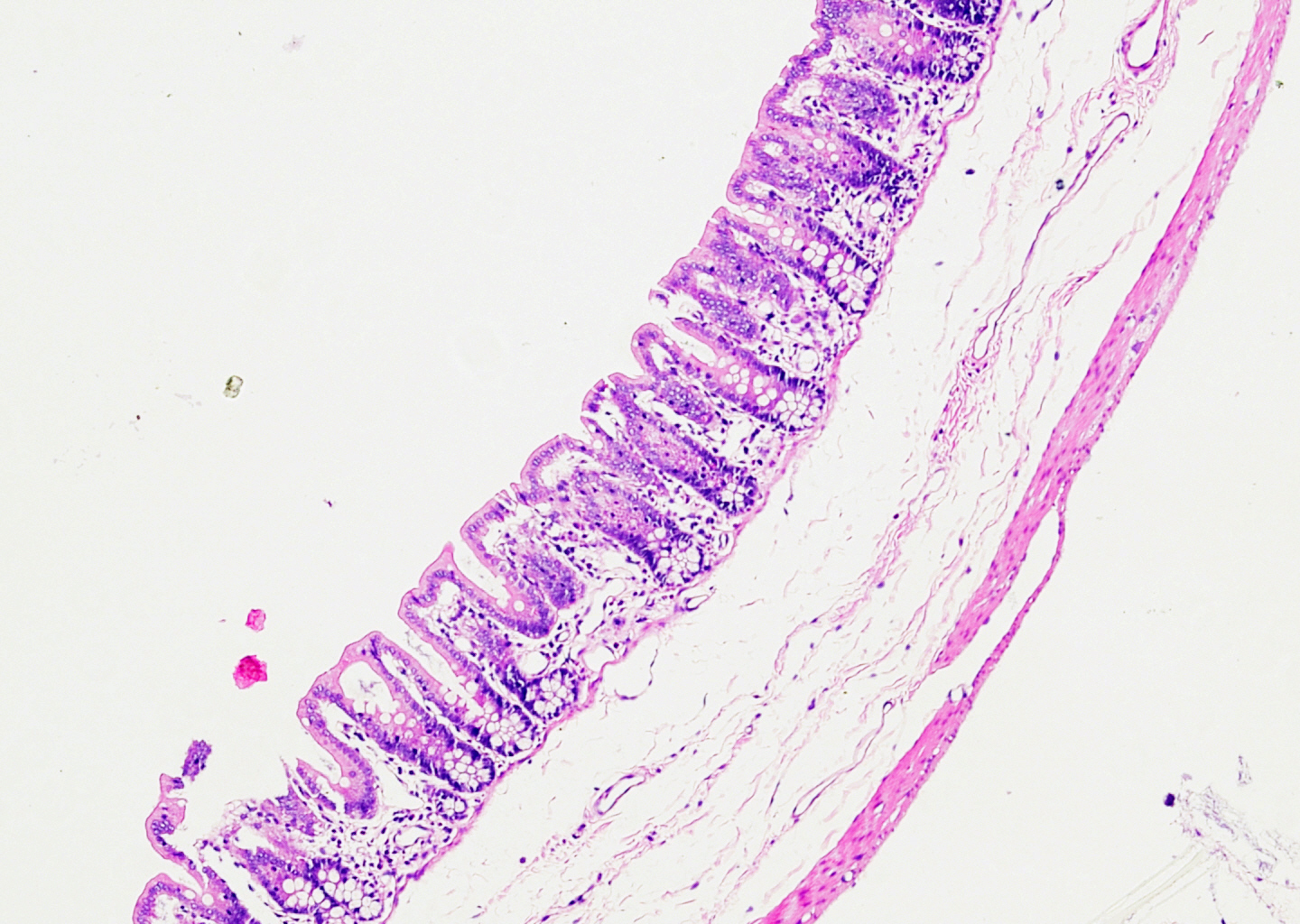

Supplement: Supplementary file 1 [file DataSheet3.ZIP › Supplementary_Material-original data1/FIGURE4/Figure4 A-SNS×100/SNS-Y1-3 100-3.jpg]

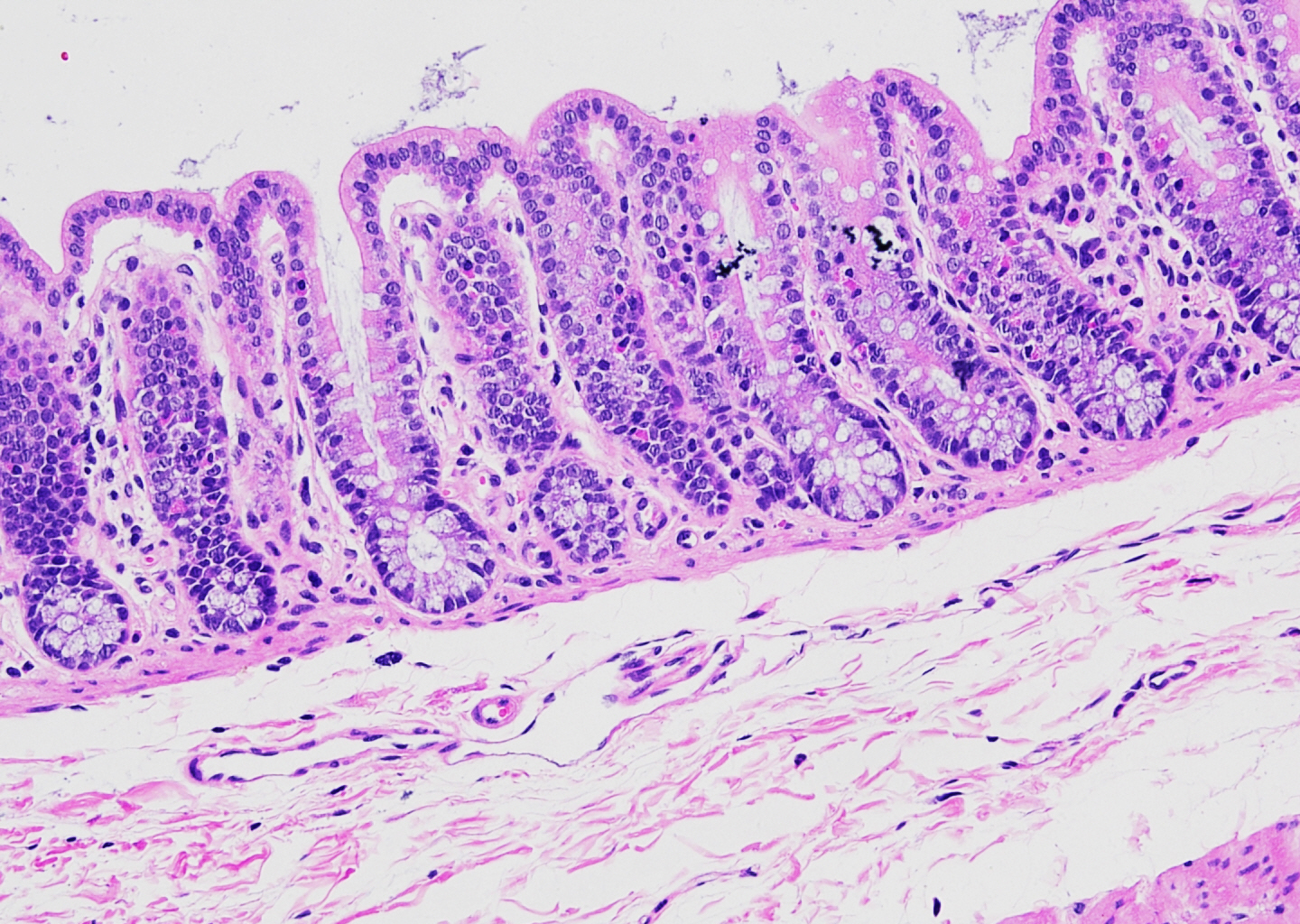

Supplement: Supplementary file 1 [file DataSheet3.ZIP › Supplementary_Material-original data1/FIGURE4/Figure4 B-control×200/A-control-CM1-9 200-3.jpg]

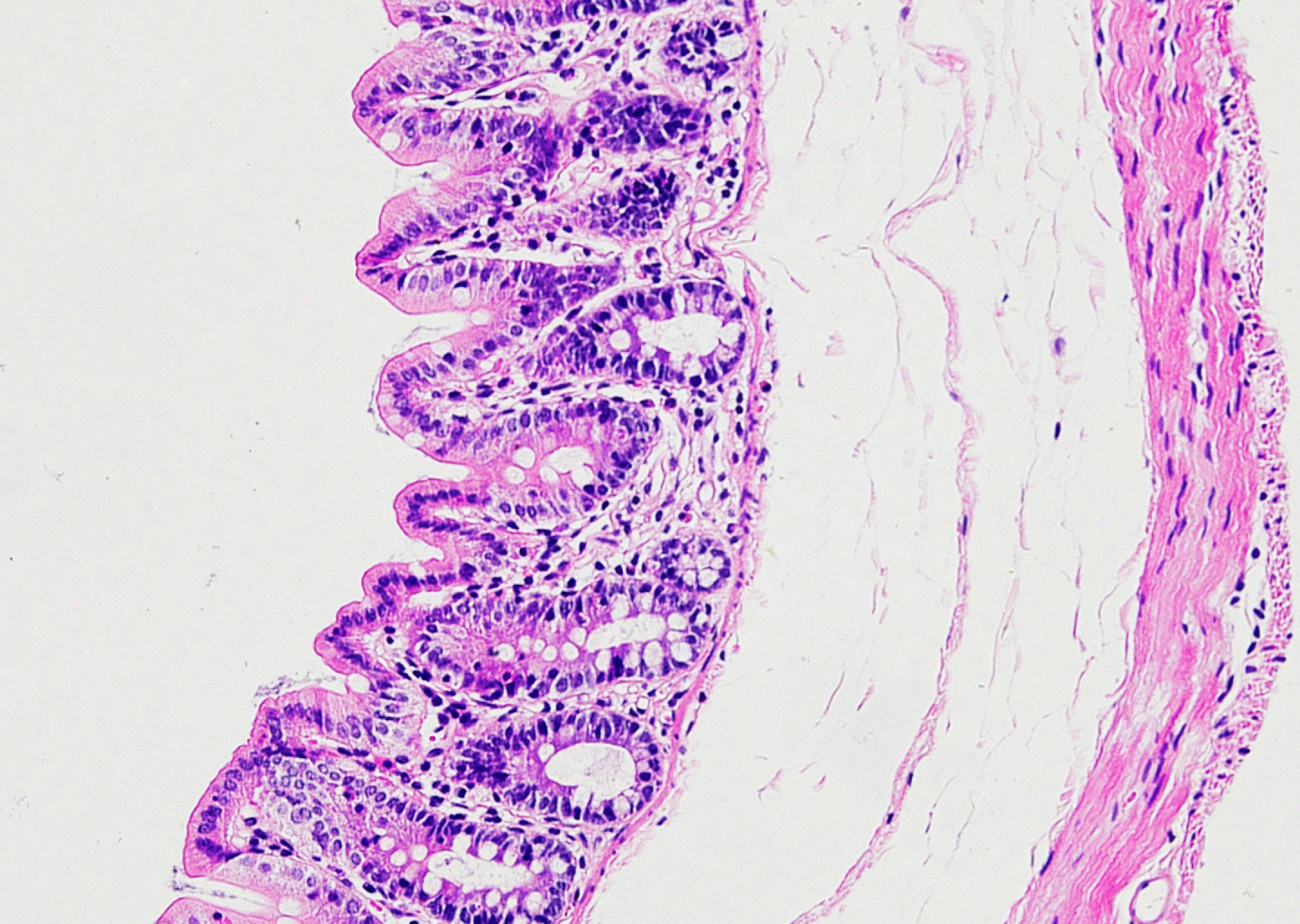

Supplement: Supplementary file 1 [file DataSheet3.ZIP › Supplementary_Material-original data1/FIGURE4/Figure4 B-FOS×200/FOS-Y1-1 200-2.jpg]

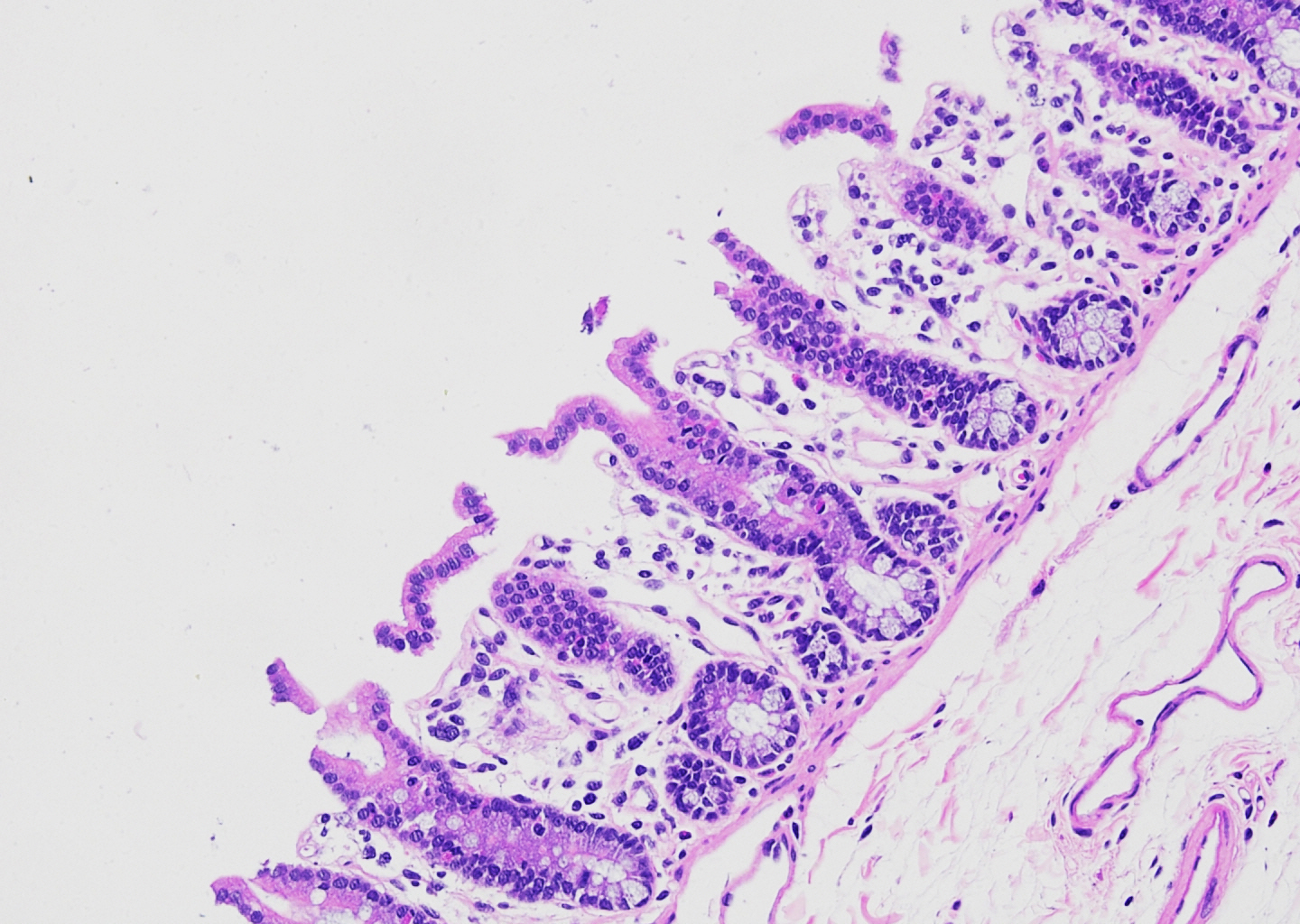

Supplement: Supplementary file 1 [file DataSheet3.ZIP › Supplementary_Material-original data1/FIGURE4/Figure4 B-model×200/B-model-CM2-13 200-3.jpg]

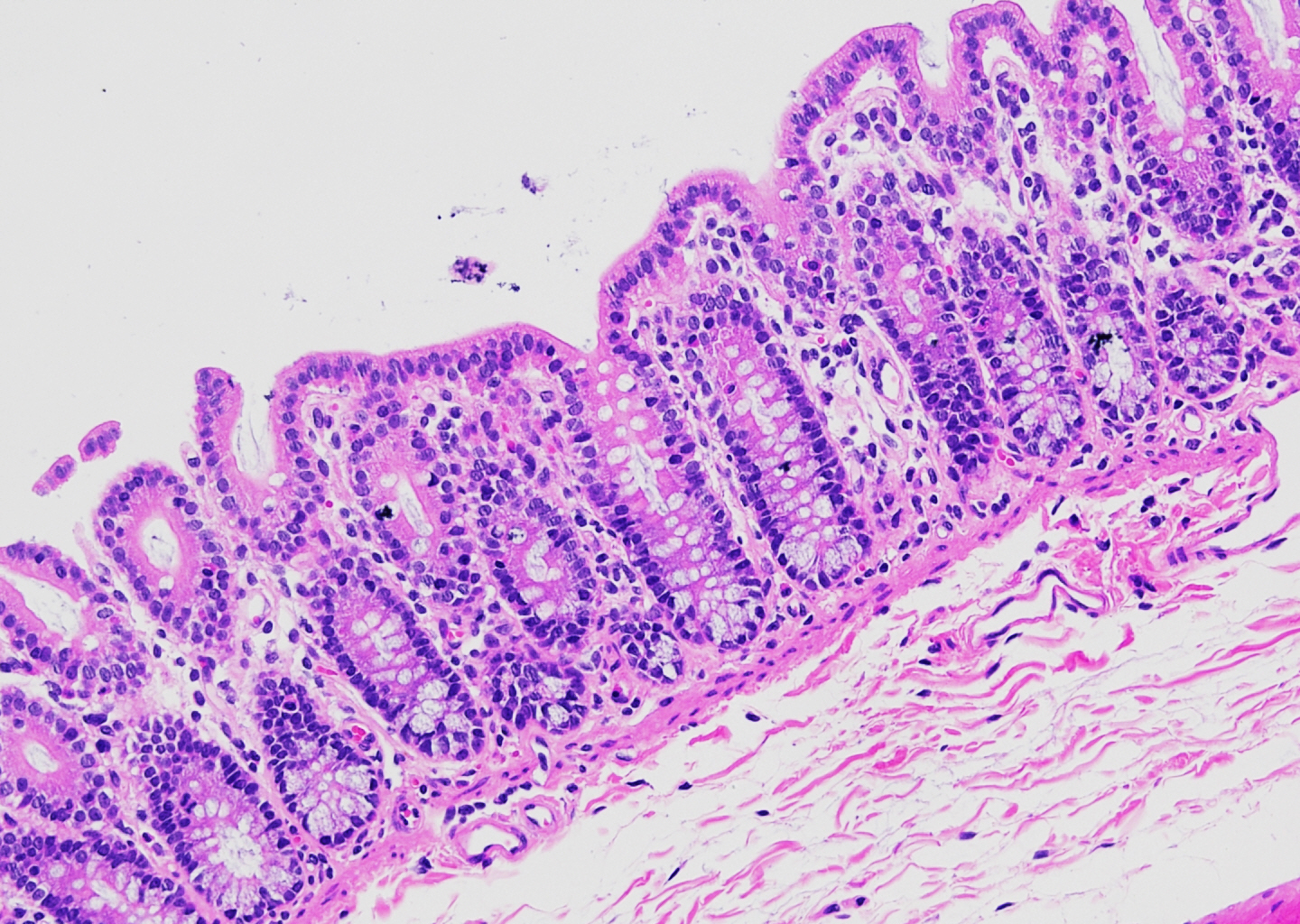

Supplement: Supplementary file 1 [file DataSheet3.ZIP › Supplementary_Material-original data1/FIGURE4/Figure4 B-SHD-H×200/SHD-H-CM4-10 200-3.jpg]

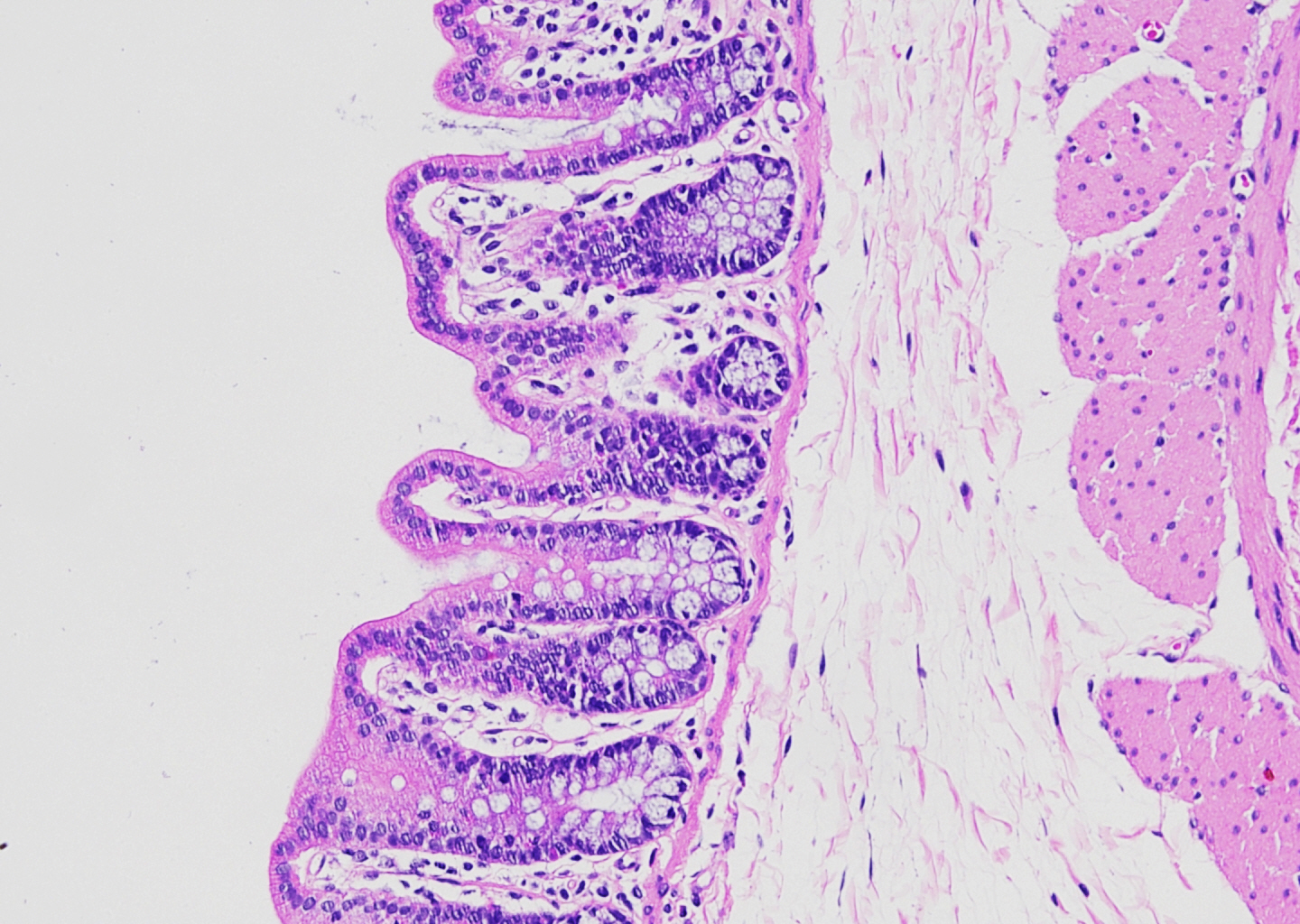

Supplement: Supplementary file 1 [file DataSheet3.ZIP › Supplementary_Material-original data1/FIGURE4/Figure4 B-SHD-L×200/SHD-L-CM3-11 200-3.jpg]

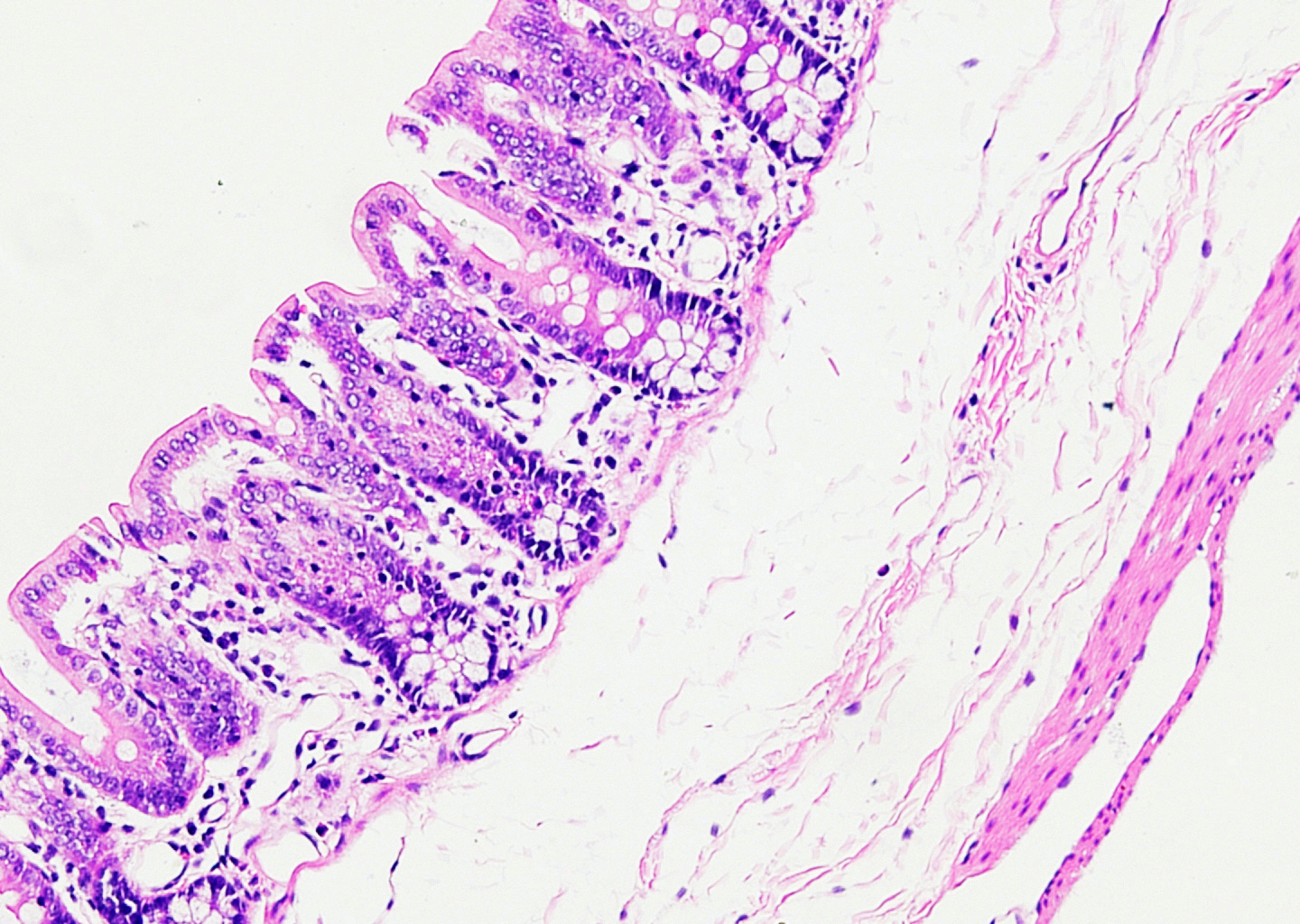

Supplement: Supplementary file 1 [file DataSheet3.ZIP › Supplementary_Material-original data1/FIGURE4/Figure4 B-SNS×200/SNS-Y1-3 200-3.jpg]

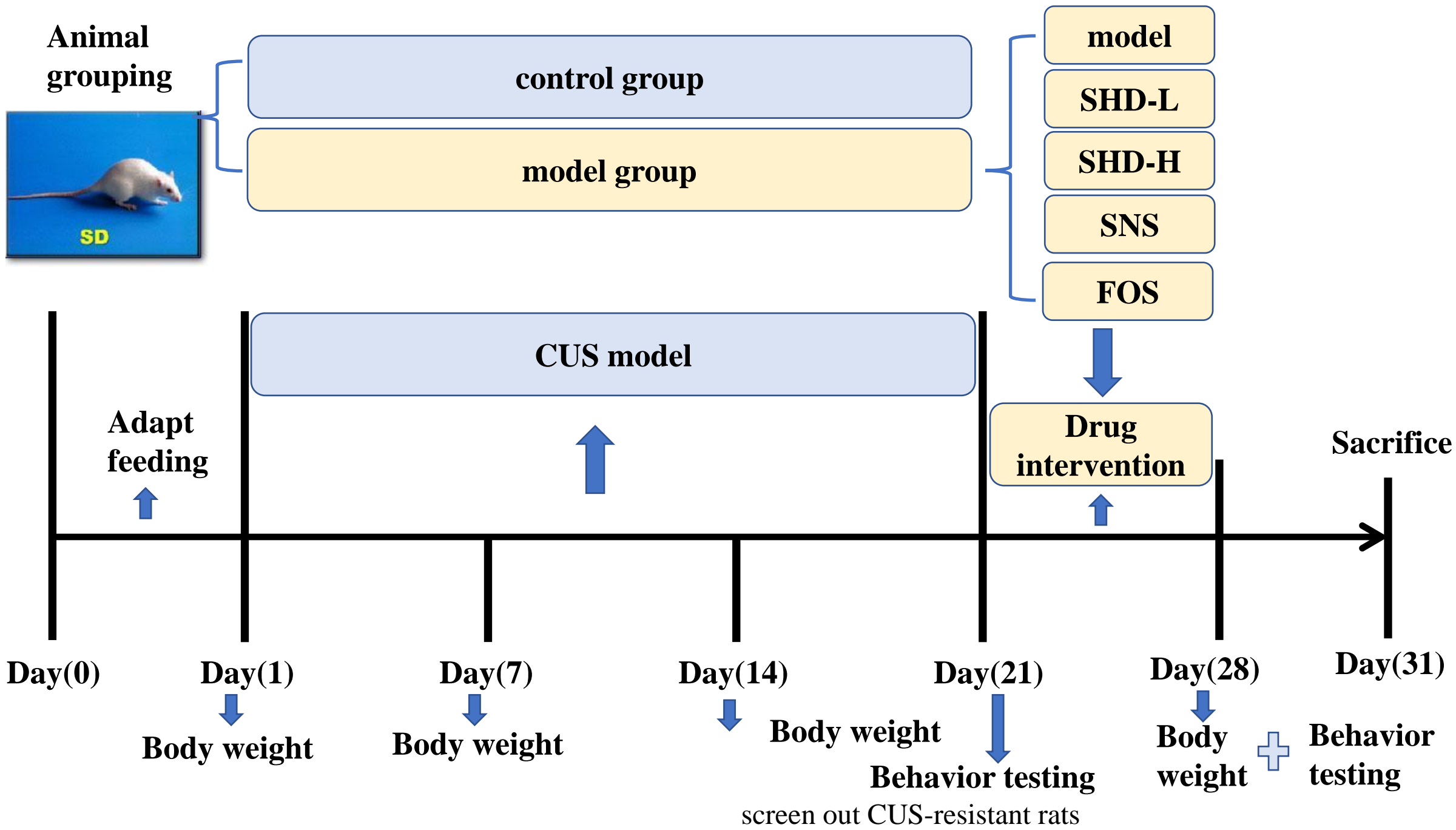

Figure 1A | Schematic diagram of time points of experimental rats feeding and grouping

Supplement: Supplementary file 2 [file DataSheet4.ZIP › Supplementary_Material-original data2/FIGURE2/Figure2A/Figure 2A.pdf]

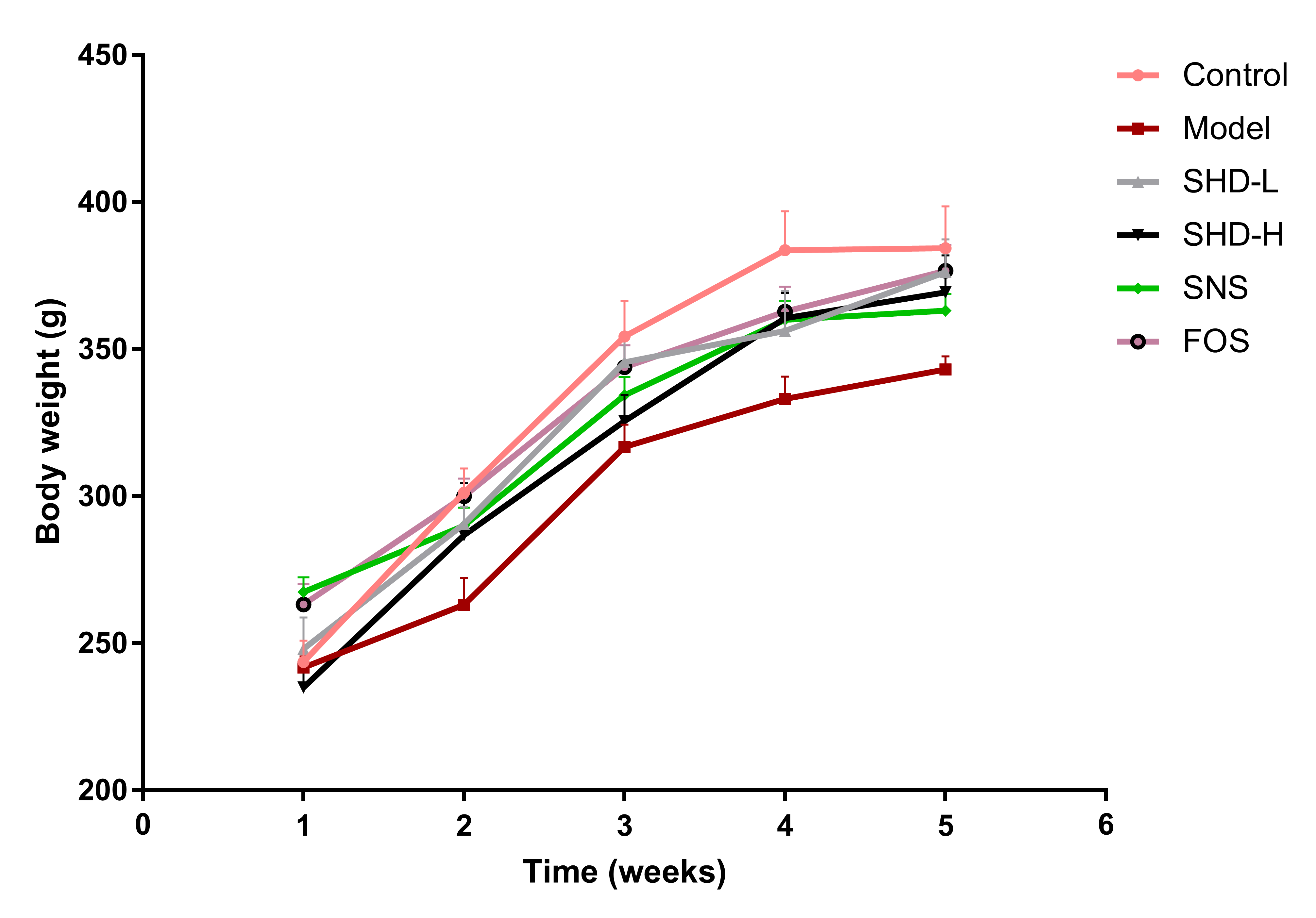

Supplement: Supplementary file 2 [file DataSheet4.ZIP › Supplementary_Material-original data2/FIGURE2/Figure2B/Figure2B Body weight.tif]

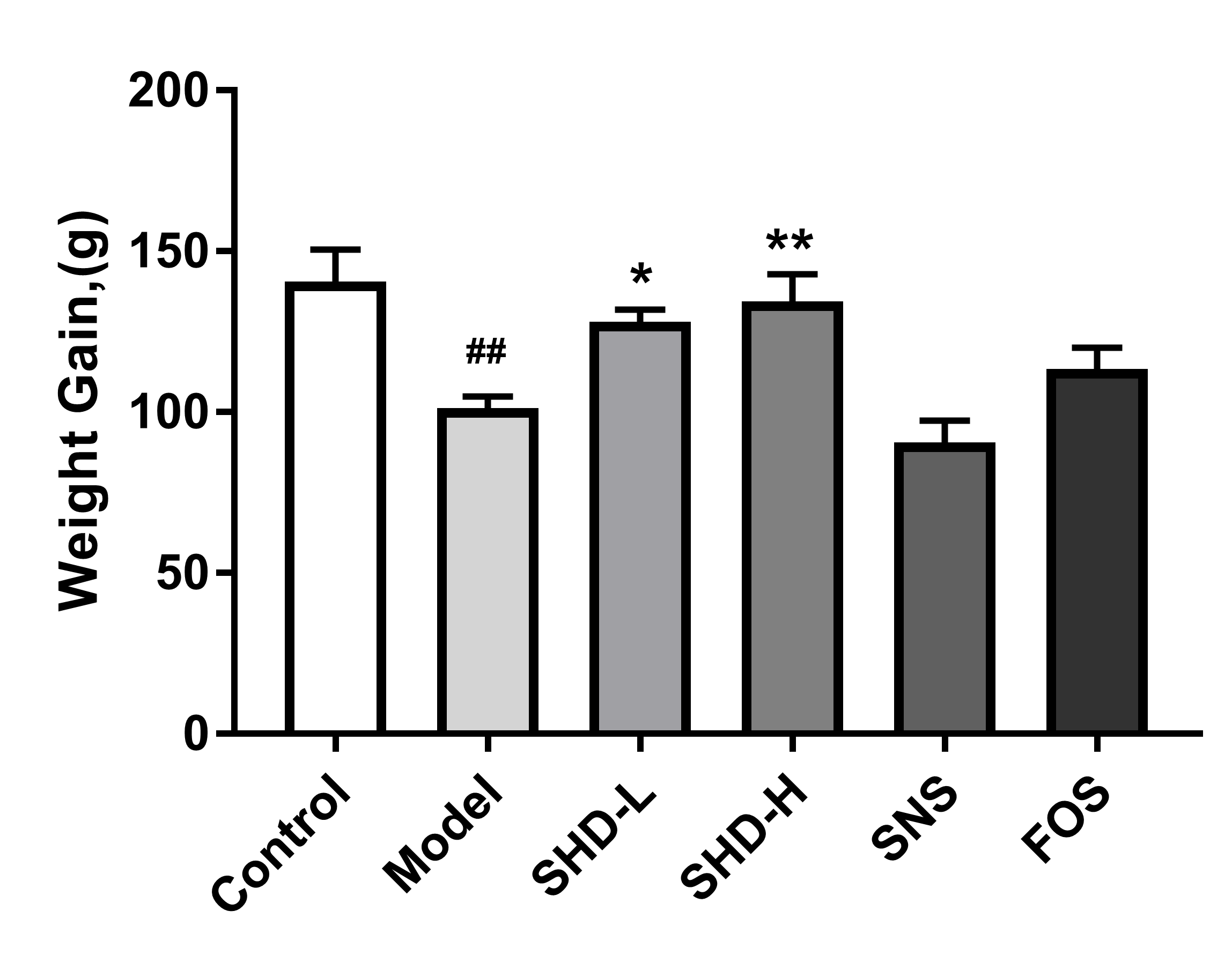

Supplement: Supplementary file 2 [file DataSheet4.ZIP › Supplementary_Material-original data2/FIGURE2/Figure2C/Figure2C Weight gain.tif]

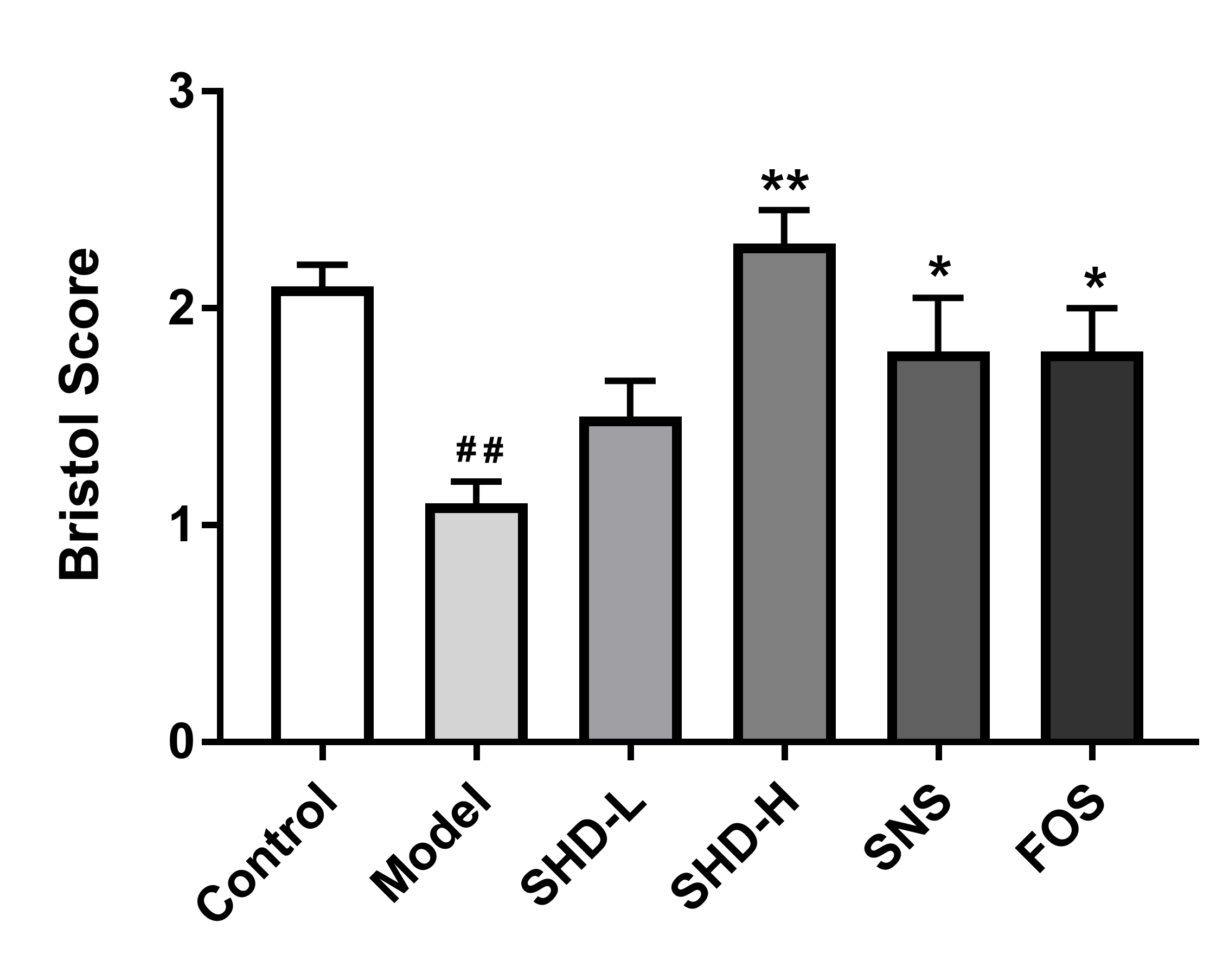

Supplement: Supplementary file 2 [file DataSheet4.ZIP › Supplementary_Material-original data2/FIGURE2/Figure2D/Figure2D Bristol Score.tif]

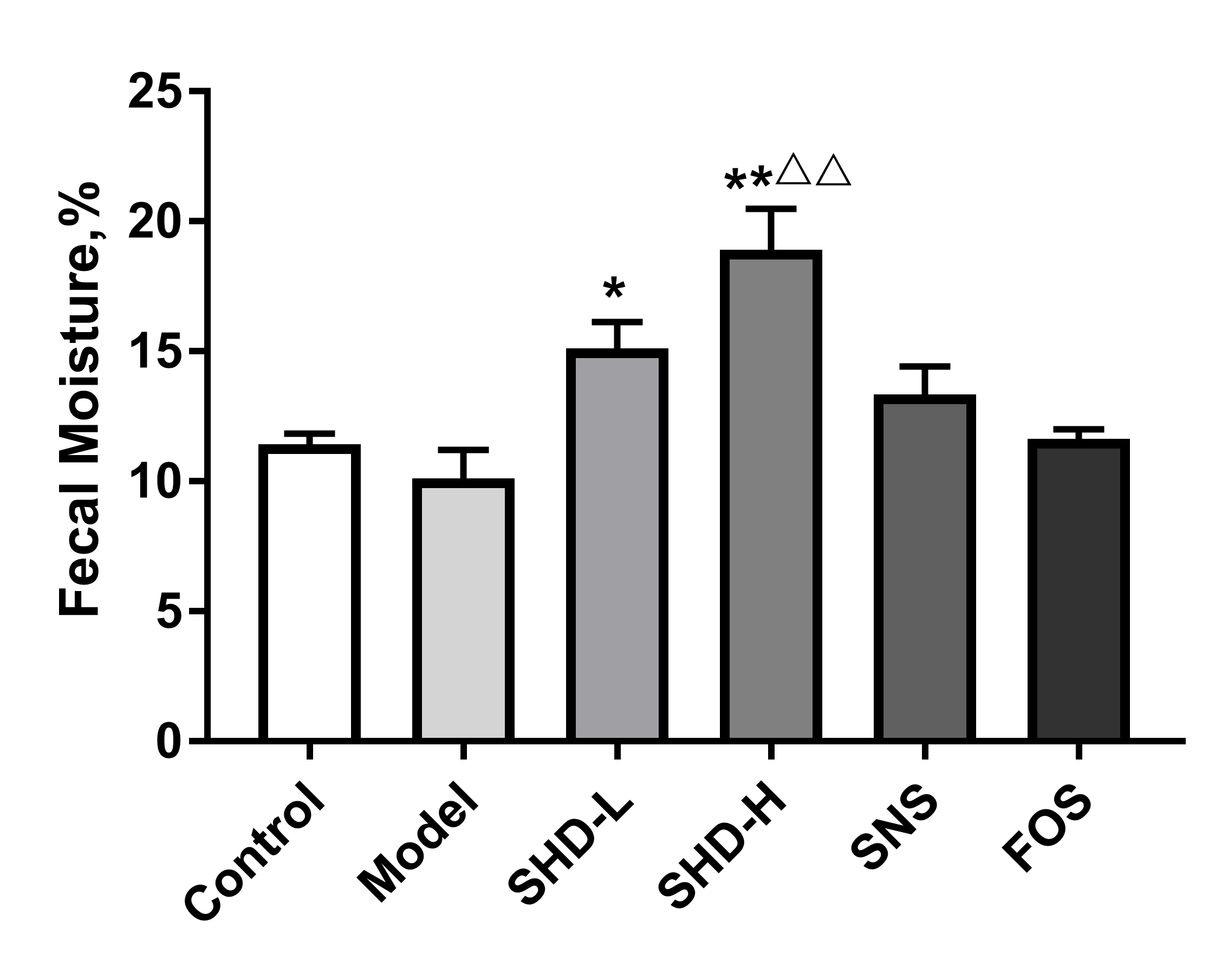

Supplement: Supplementary file 2 [file DataSheet4.ZIP › Supplementary_Material-original data2/FIGURE2/Figure2E/Figure2E Fecal Moisture.tif]

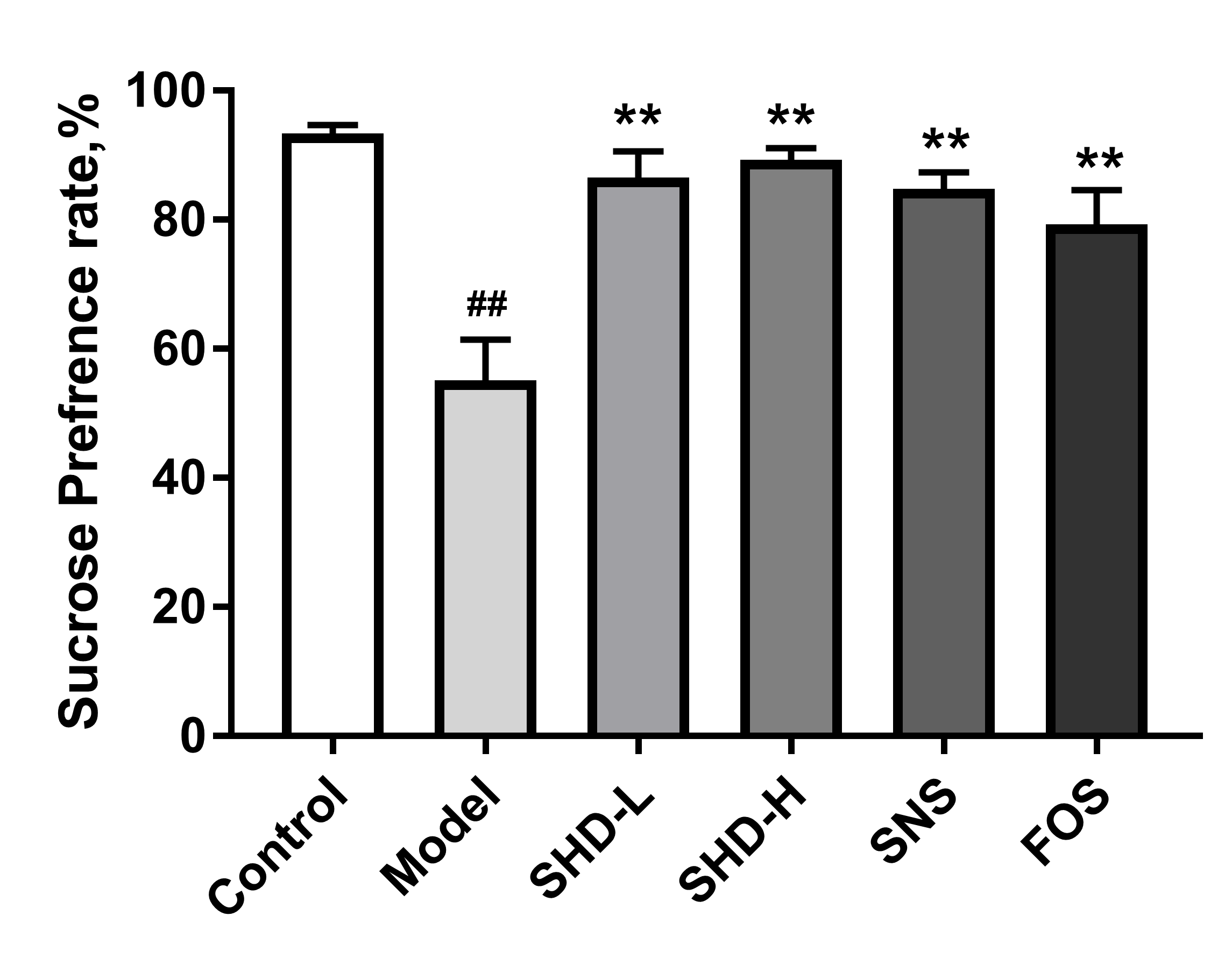

Supplement: Supplementary file 2 [file DataSheet4.ZIP › Supplementary_Material-original data2/FIGURE2/Figure2F/Figures2F-Sucrose Prefrence Test.tif]

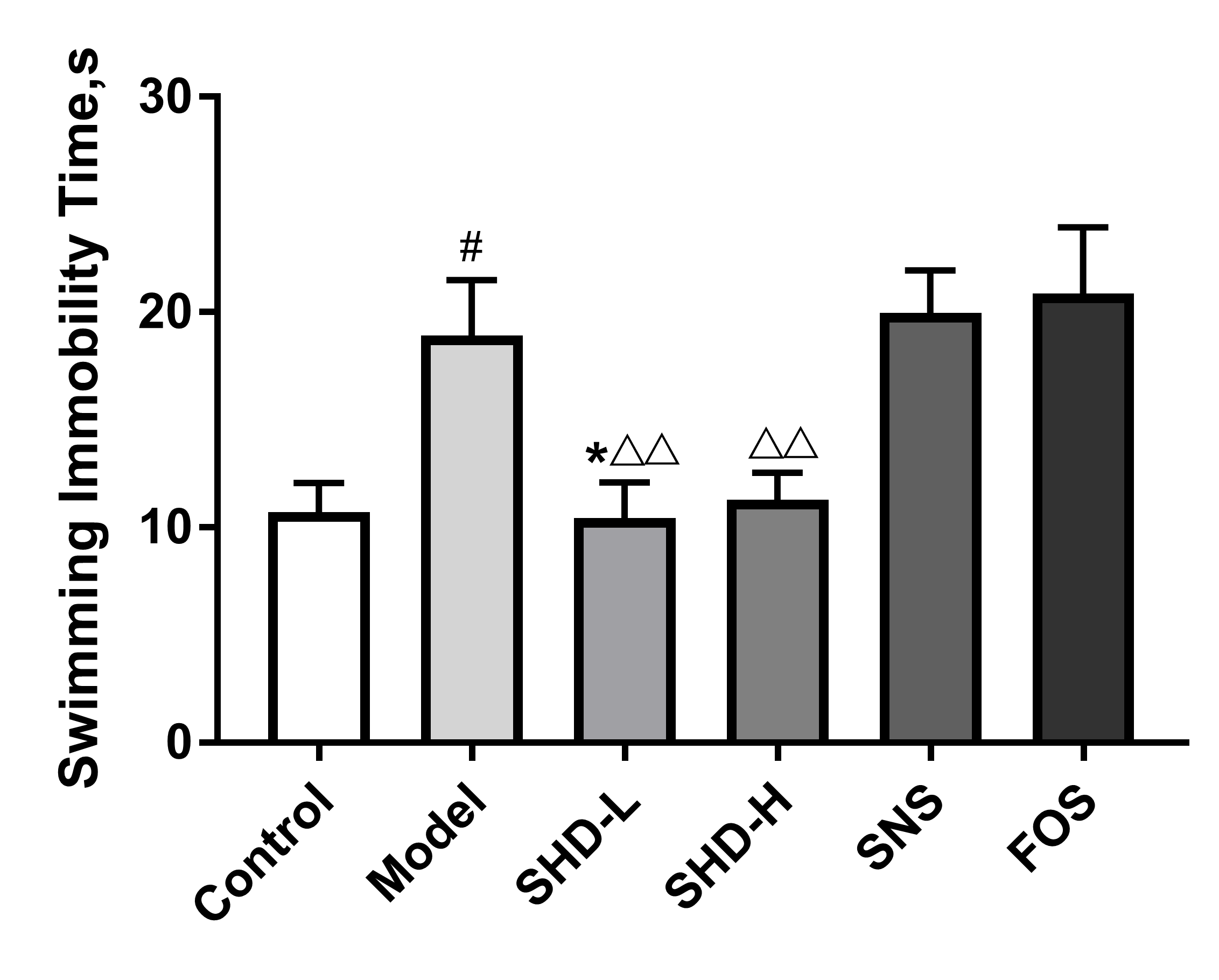

Supplement: Supplementary file 2 [file DataSheet4.ZIP › Supplementary_Material-original data2/FIGURE2/Figure2J/Figure2J-FST.tif]

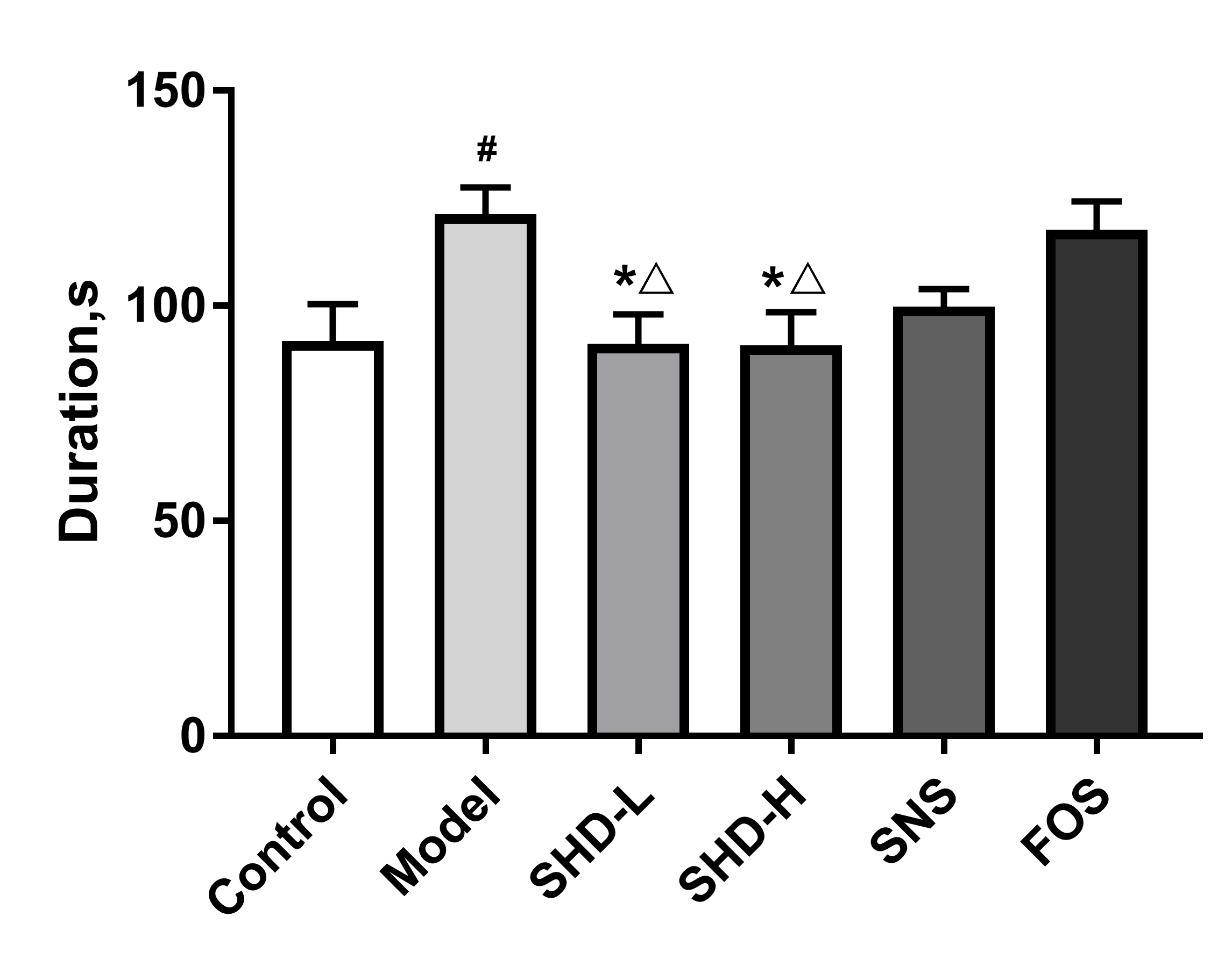

Supplement: Supplementary file 2 [file DataSheet4.ZIP › Supplementary_Material-original data2/FIGURE2/Figures 2G,2H,2I/Figure 2G.OFT-Duration.tif]

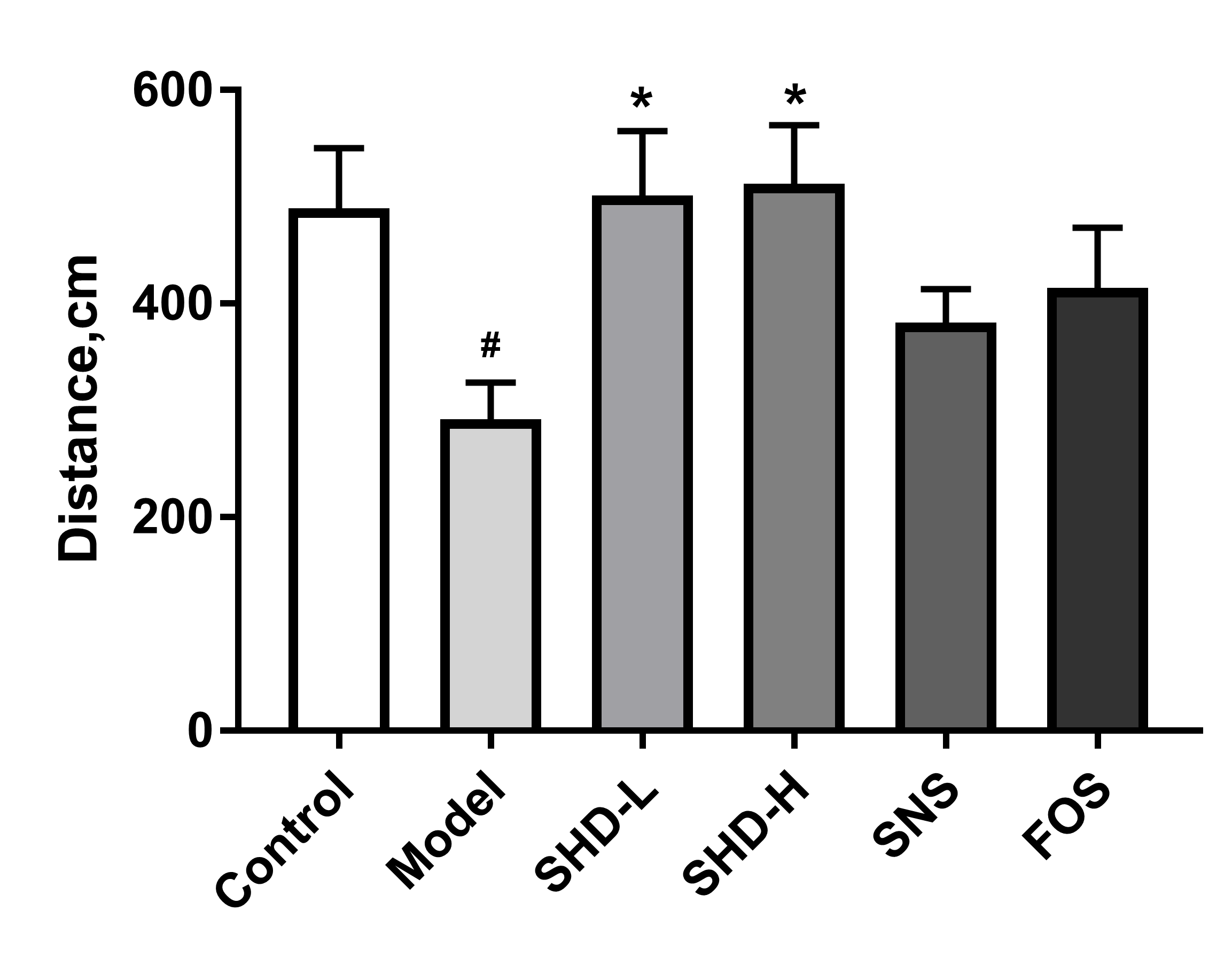

Supplement: Supplementary file 2 [file DataSheet4.ZIP › Supplementary_Material-original data2/FIGURE2/Figures 2G,2H,2I/Figure 2H.OFT-Distance.tif]

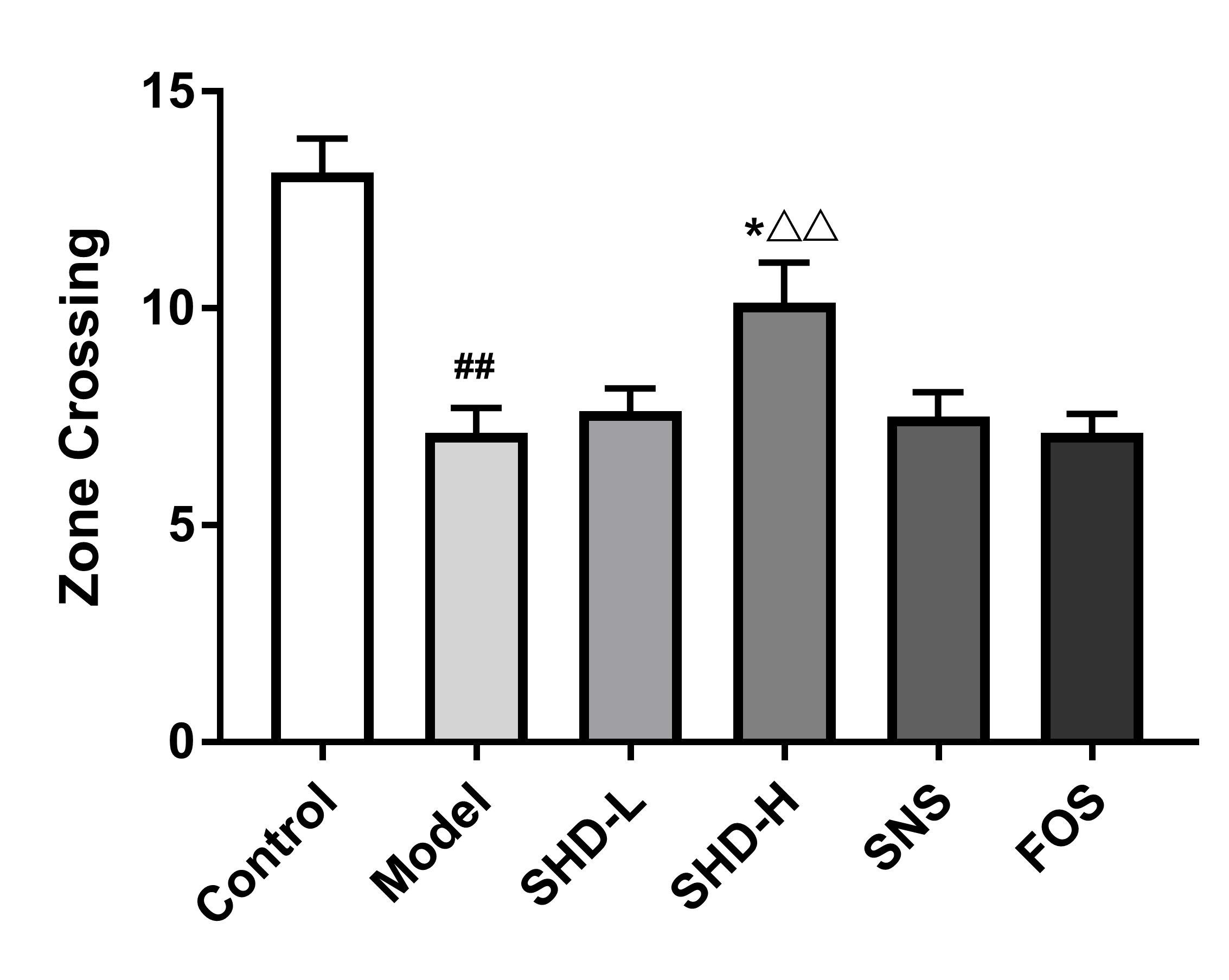

Supplement: Supplementary file 2 [file DataSheet4.ZIP › Supplementary_Material-original data2/FIGURE2/Figures 2G,2H,2I/Figure 2I.OFT-Zone Crossing.tif]

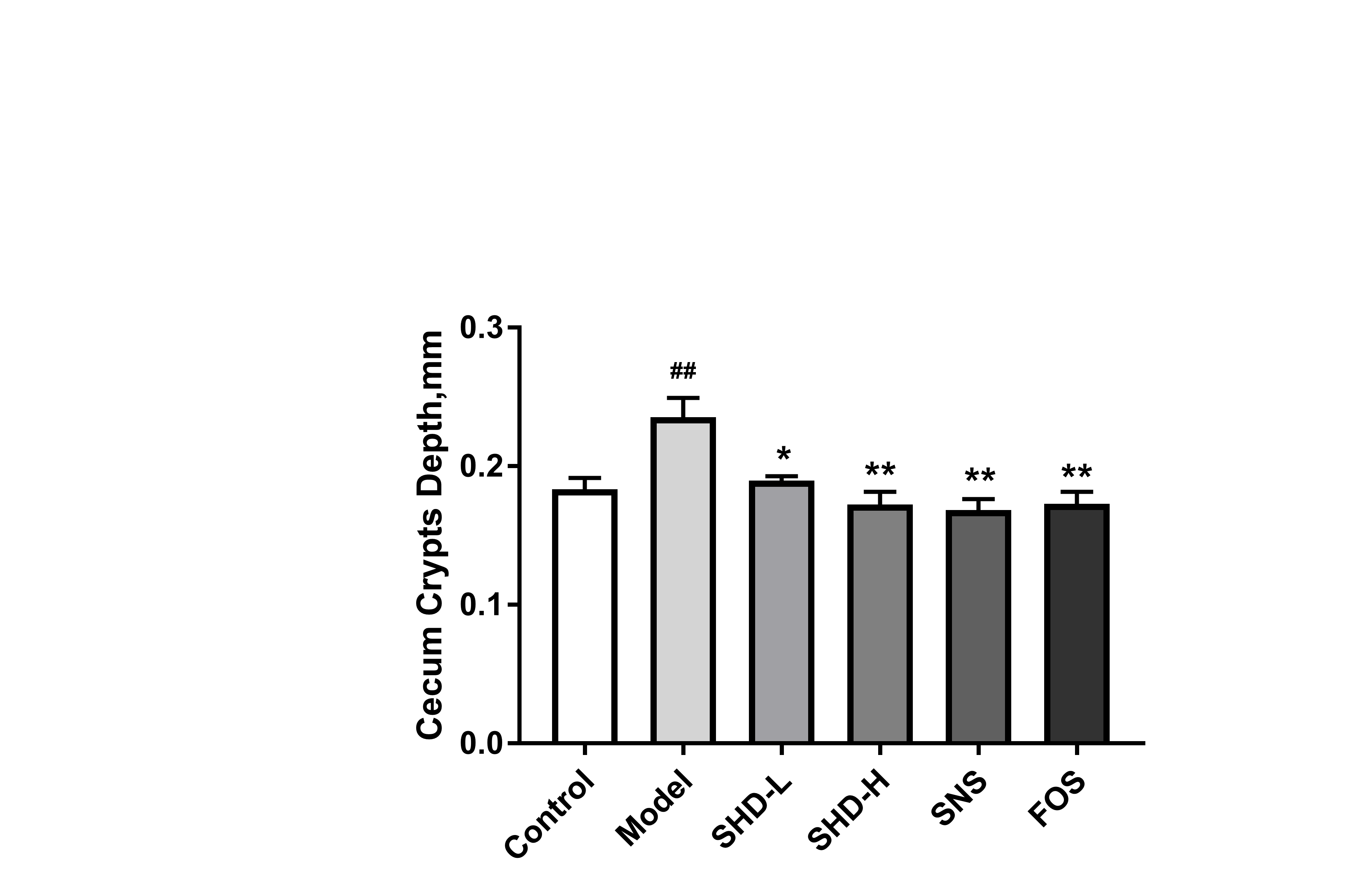

Supplement: Supplementary file 2 [file DataSheet4.ZIP › Supplementary_Material-original data2/FIGURE4/Figures 4C,4D/Figure4C-Cecum Crypts Depth.tif]

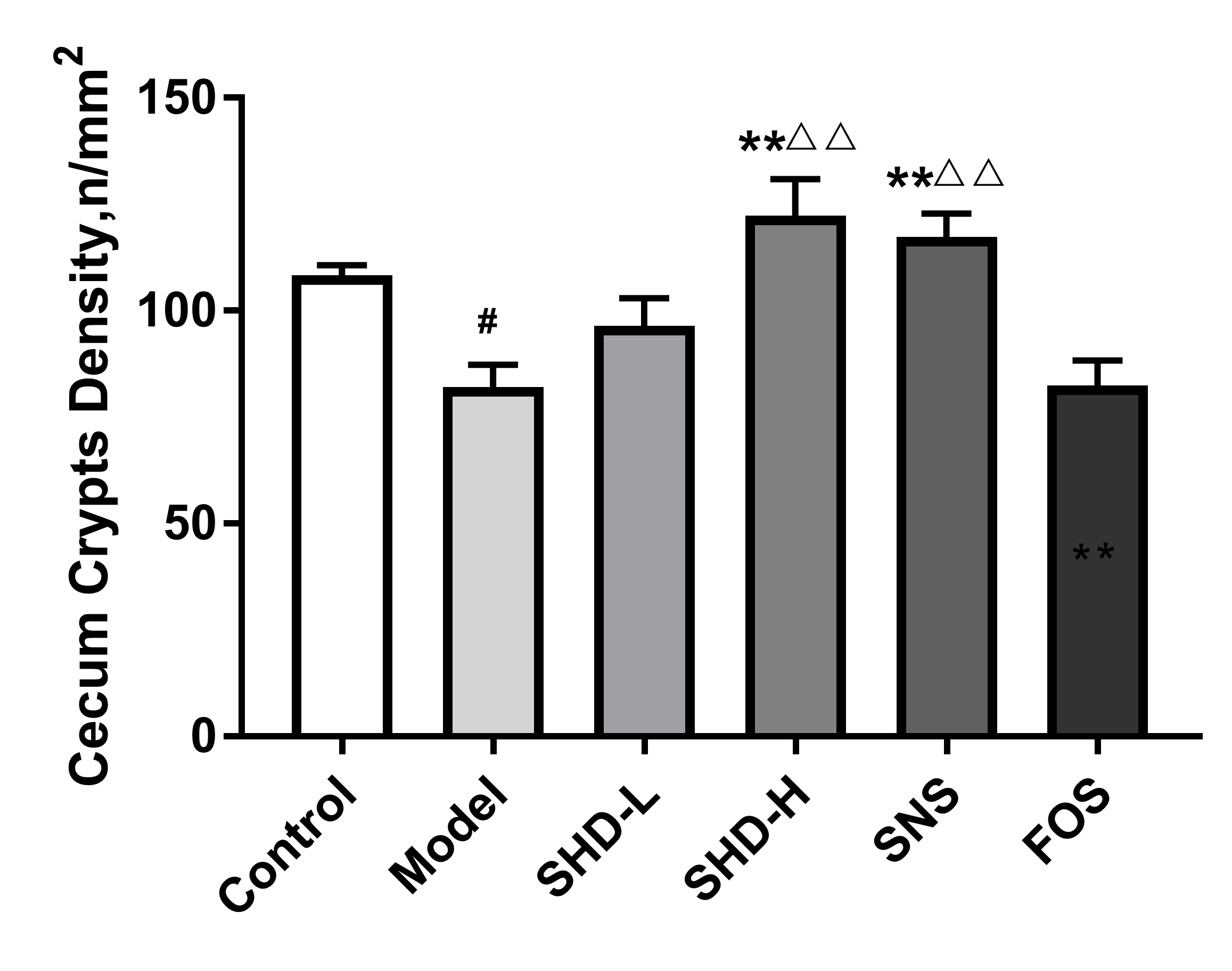

Supplement: Supplementary file 2 [file DataSheet4.ZIP › Supplementary_Material-original data2/FIGURE4/Figures 4C,4D/Figure4D-Cecum Crypts Density.tif]

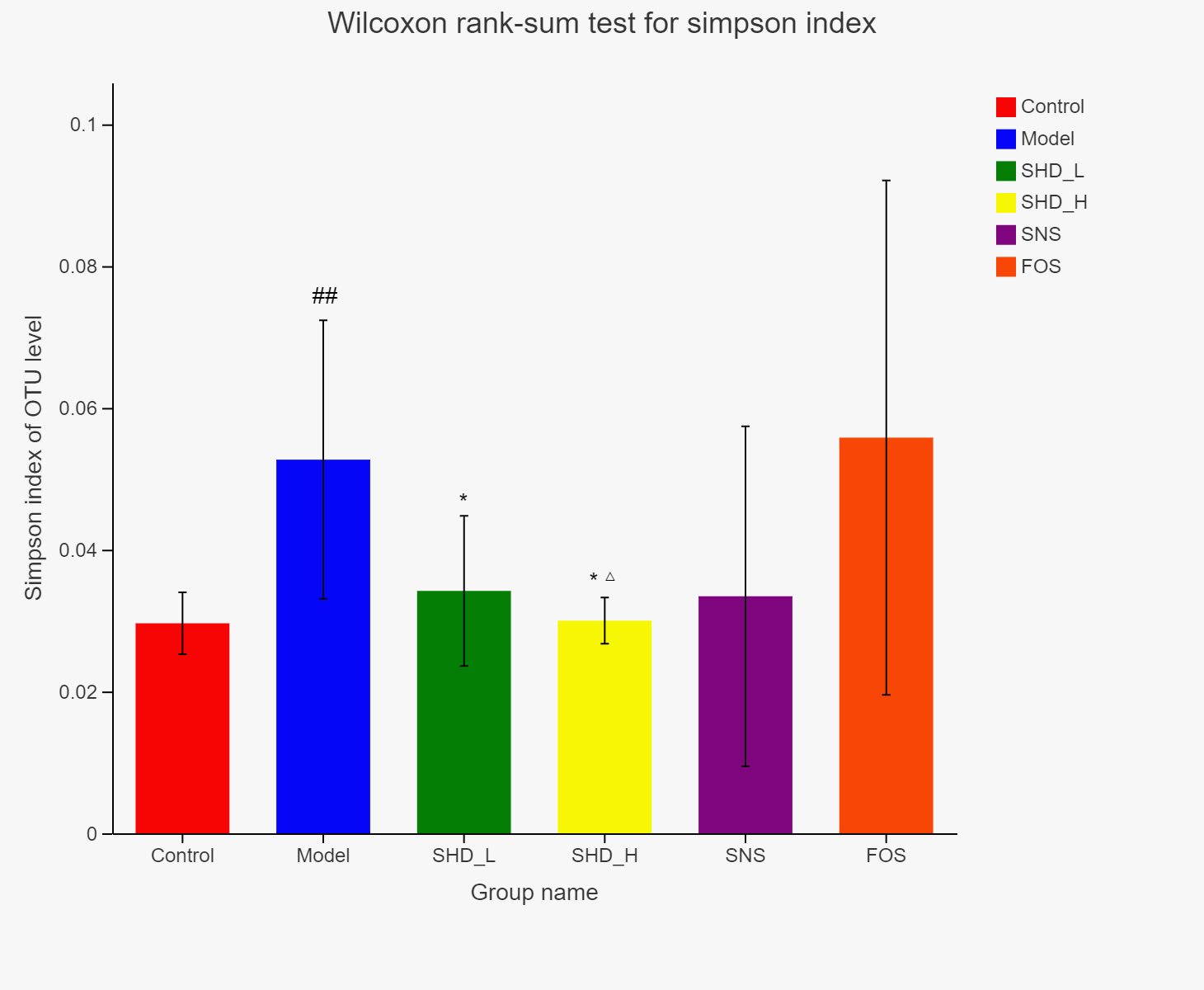

Supplement: Supplementary file 2 [file DataSheet4.ZIP › Supplementary_Material-original data2/FIGURE5/Figure 5A.png]

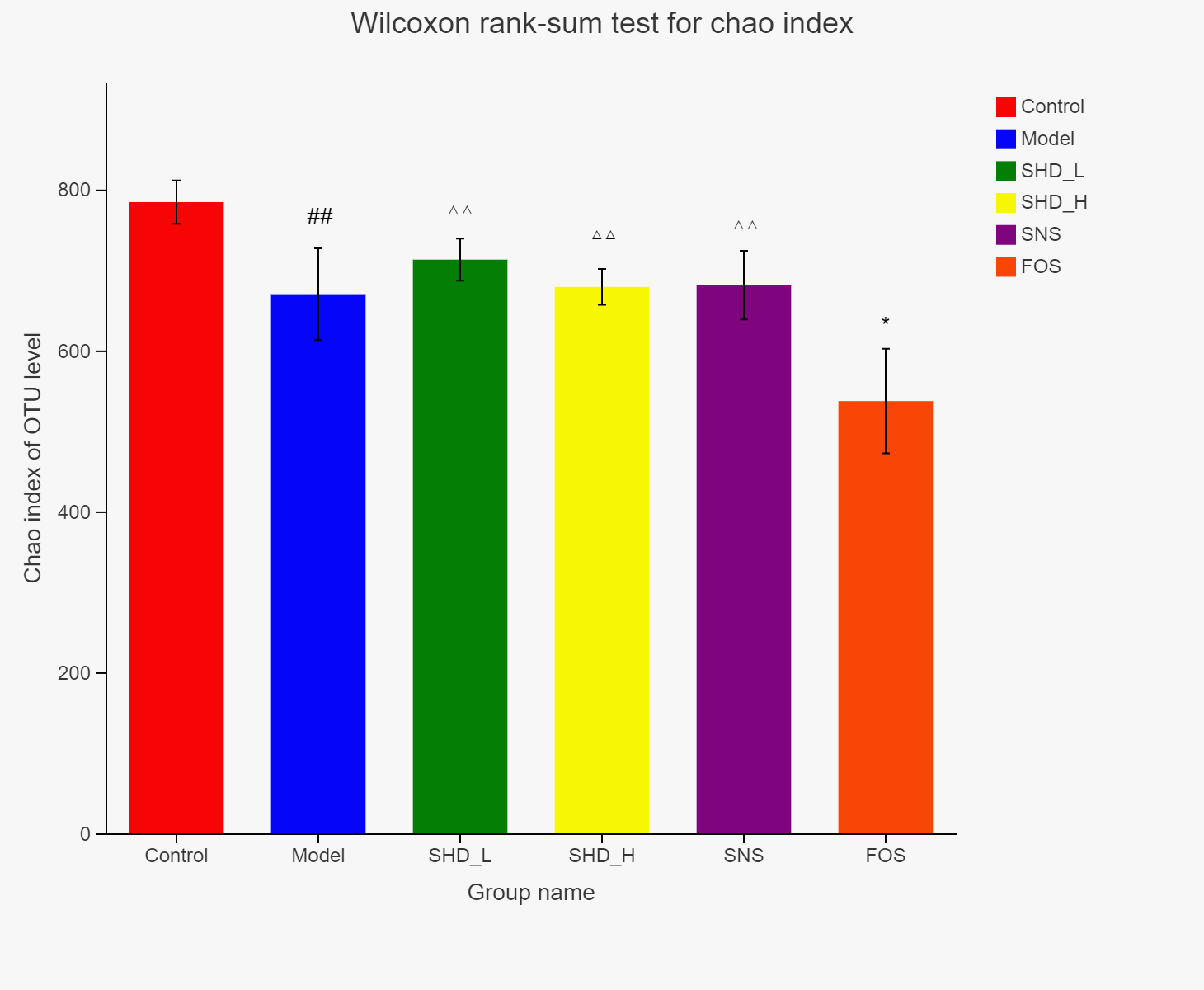

Supplement: Supplementary file 2 [file DataSheet4.ZIP › Supplementary_Material-original data2/FIGURE5/Figure 5B.png]

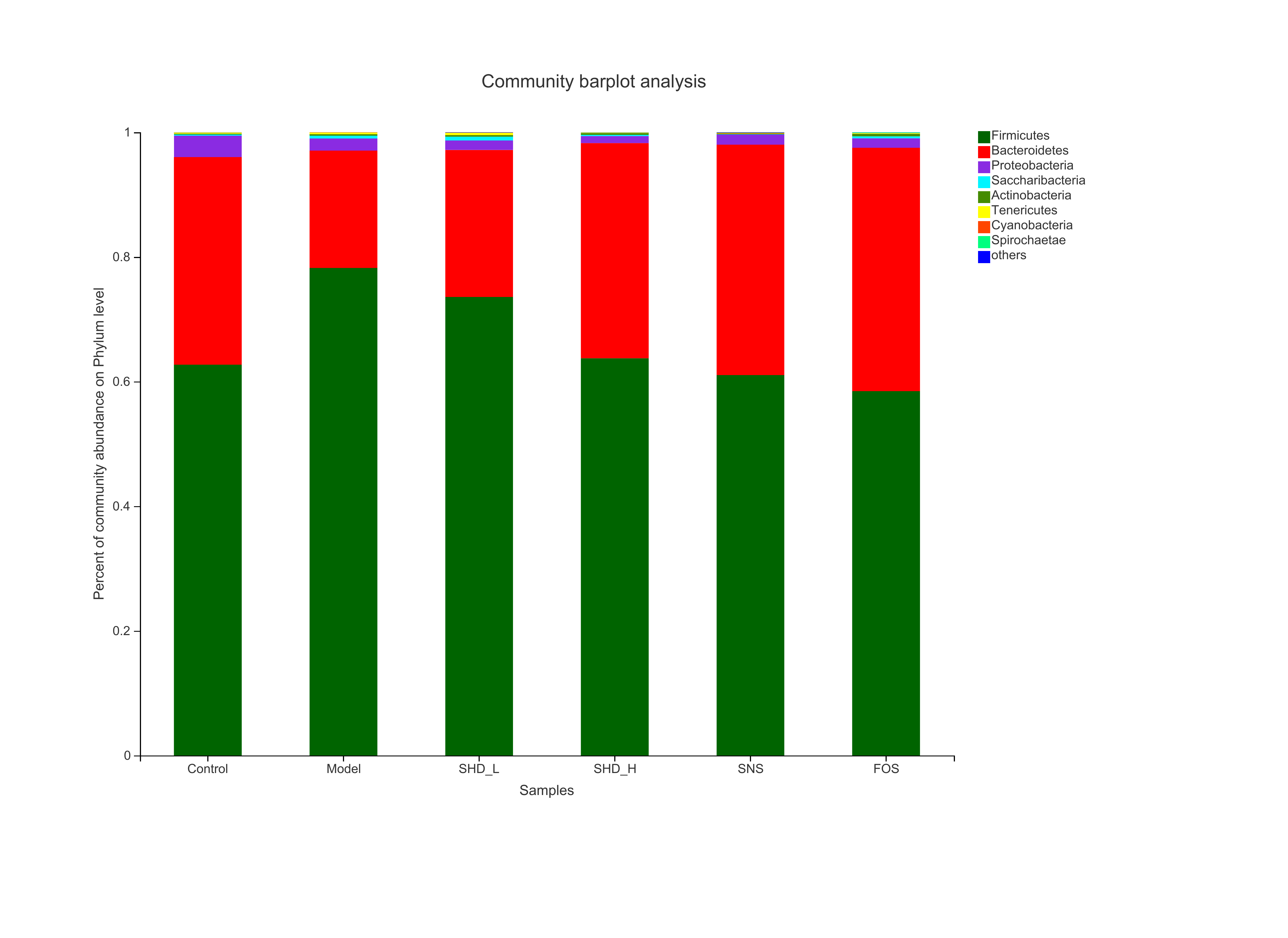

Supplement: Supplementary file 2 [file DataSheet4.ZIP › Supplementary_Material-original data2/FIGURE5/Figure 5C.png]

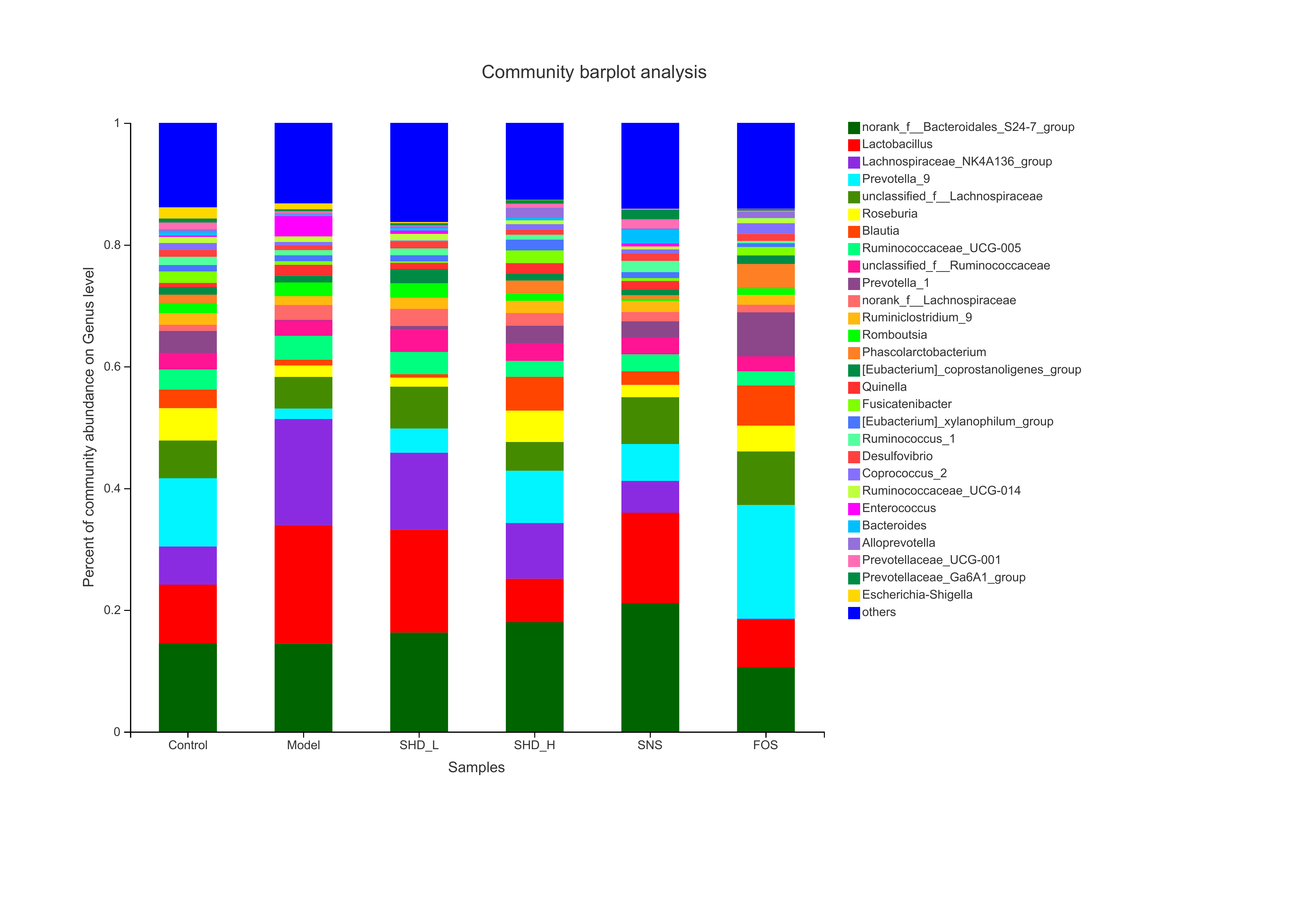

Supplement: Supplementary file 2 [file DataSheet4.ZIP › Supplementary_Material-original data2/FIGURE5/Figure 5D.png]

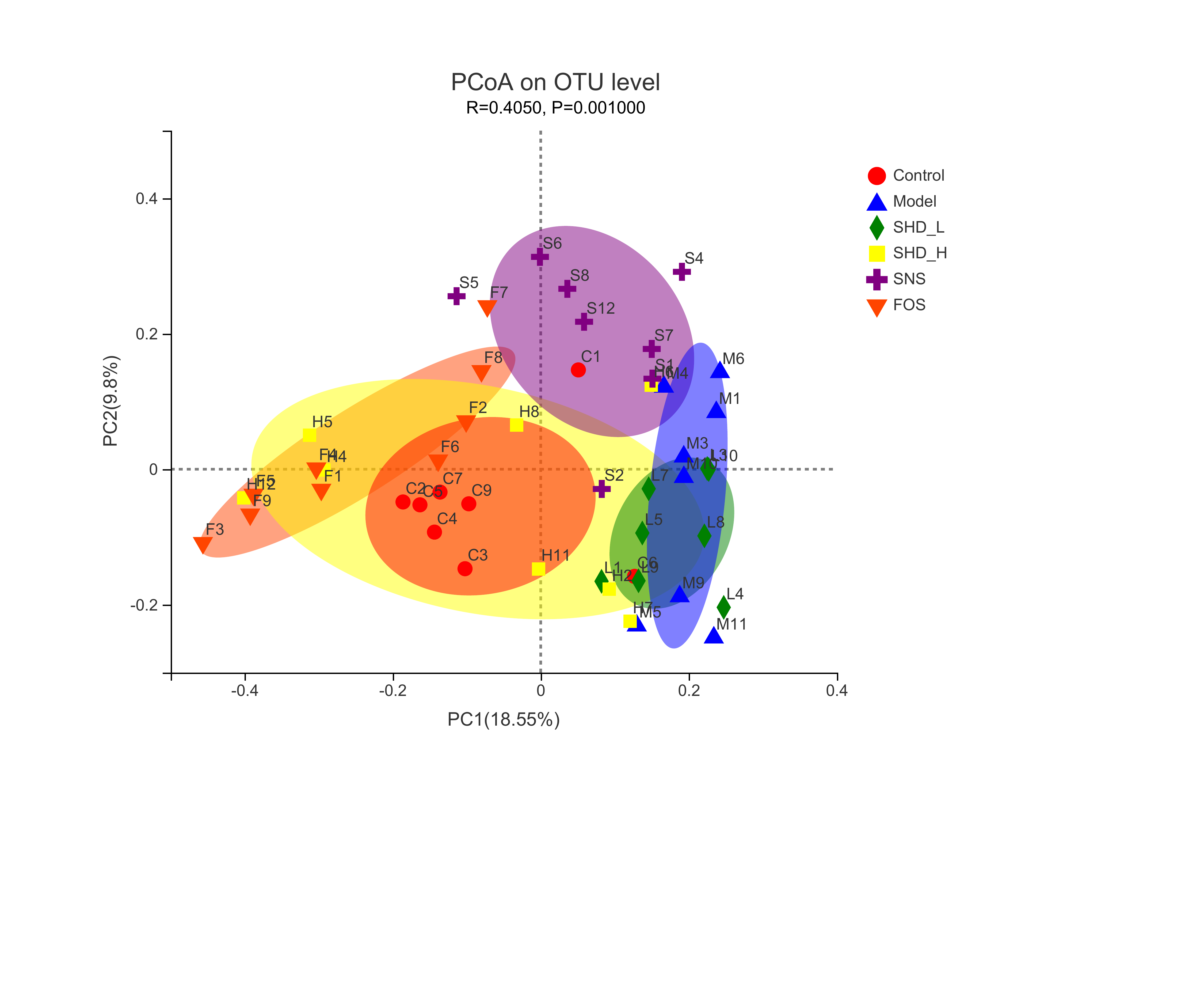

Supplement: Supplementary file 2 [file DataSheet4.ZIP › Supplementary_Material-original data2/FIGURE5/Figure 5E.png]

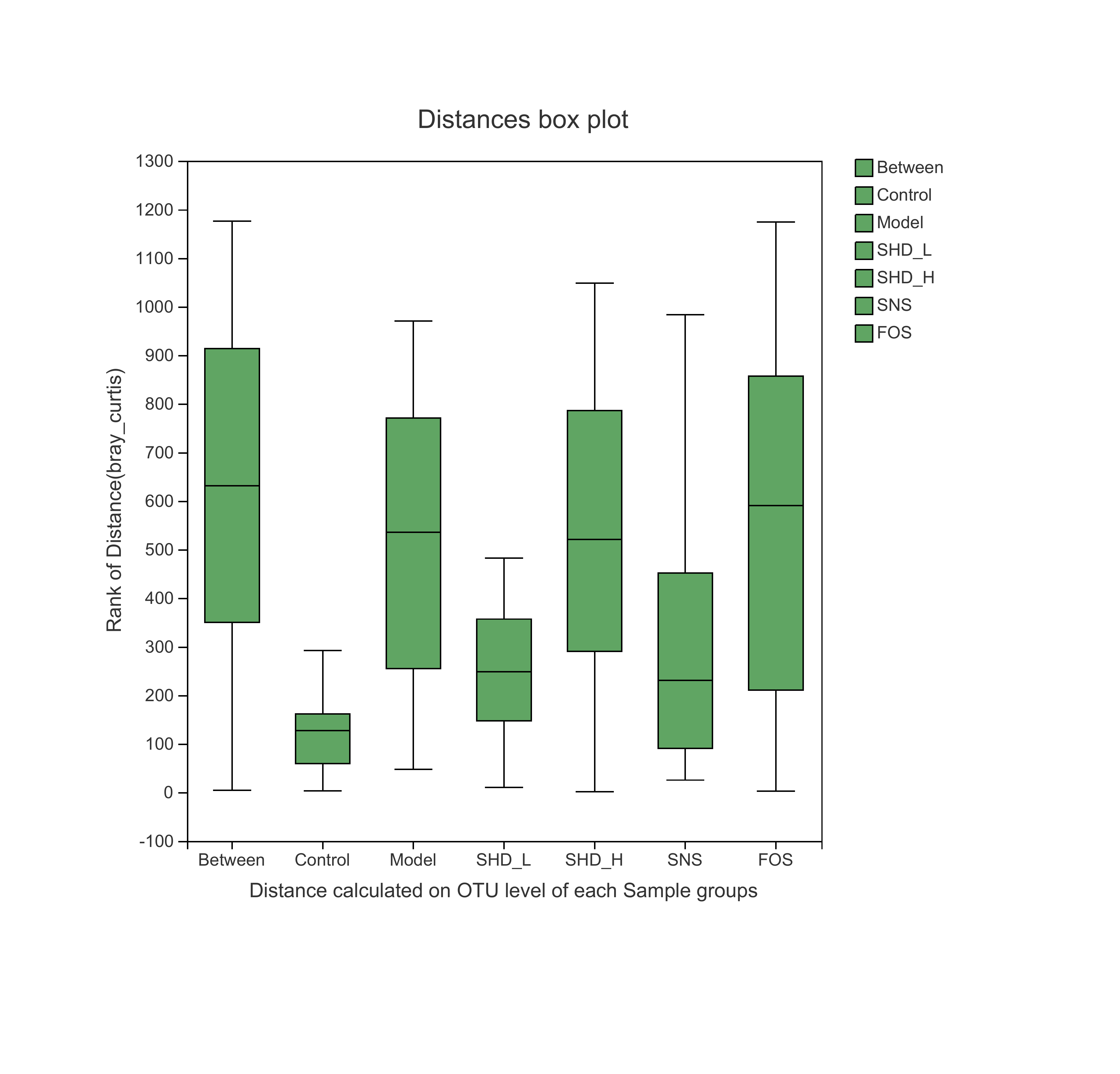

Supplement: Supplementary file 2 [file DataSheet4.ZIP › Supplementary_Material-original data2/FIGURE5/Figure 5F.png]

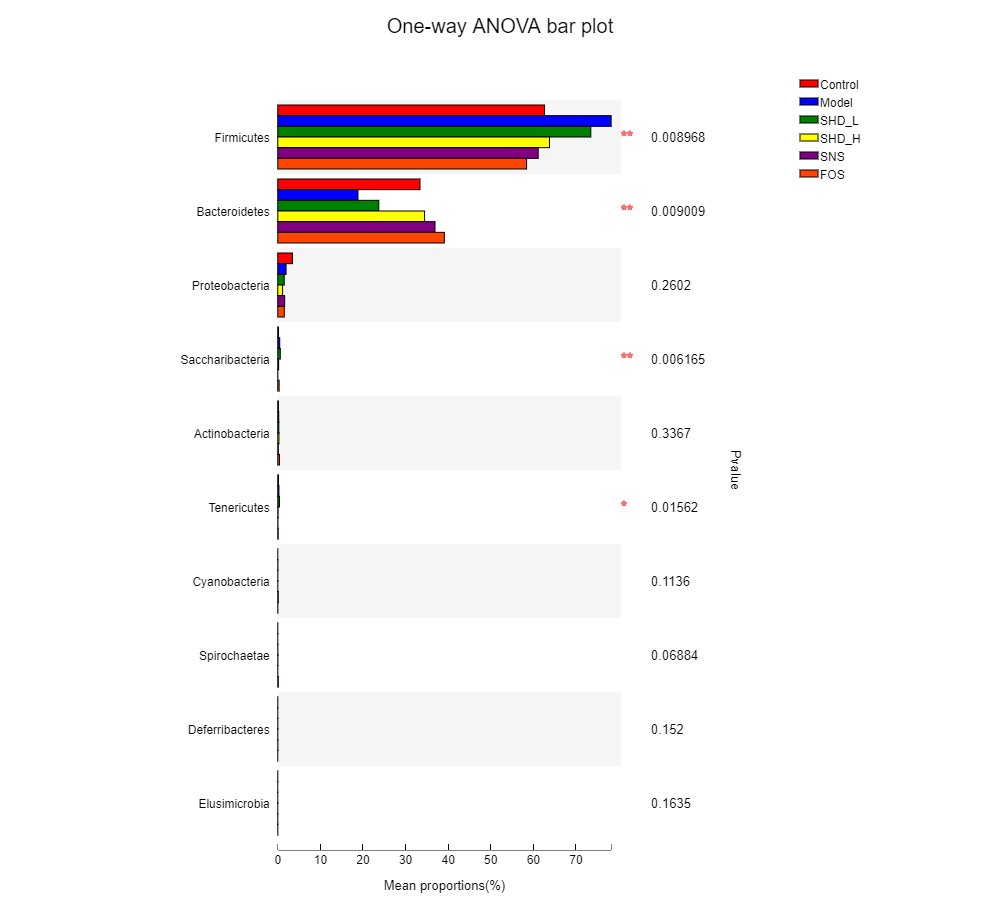

Supplement: Supplementary file 2 [file DataSheet4.ZIP › Supplementary_Material-original data2/FIGURE5/Figure 5G.png]

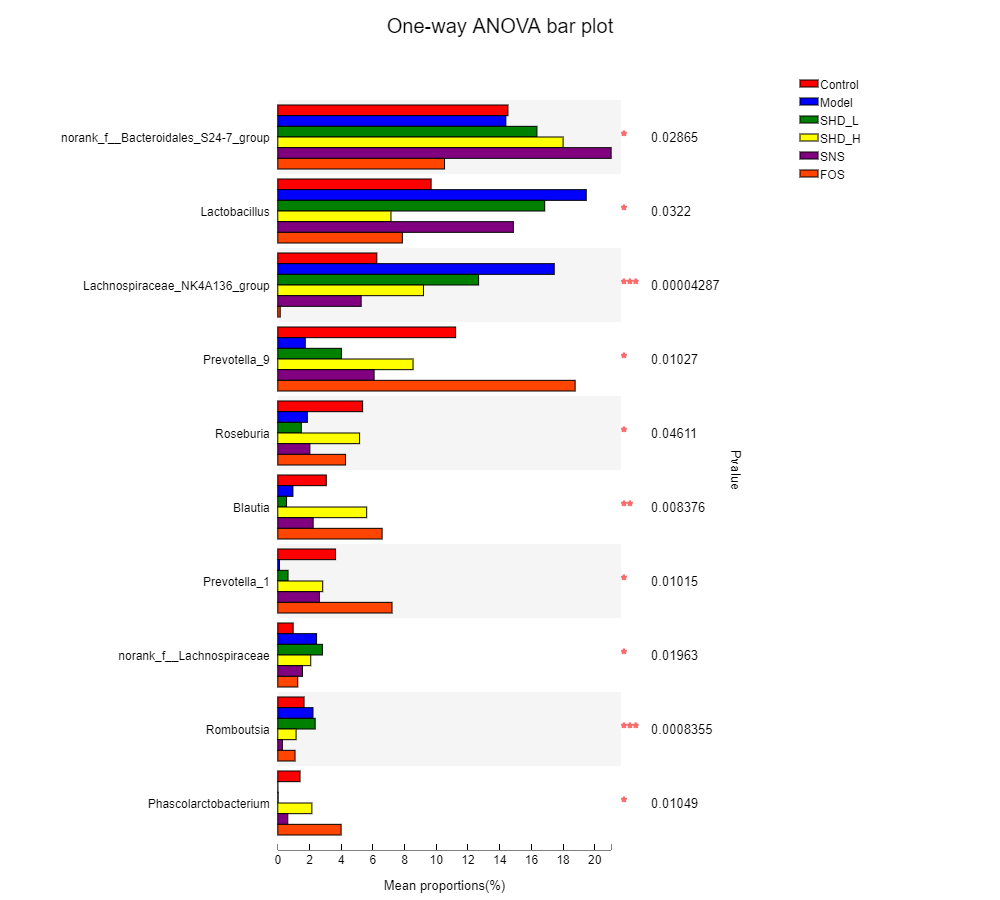

Supplement: Supplementary file 2 [file DataSheet4.ZIP › Supplementary_Material-original data2/FIGURE5/Figure 5H.png]

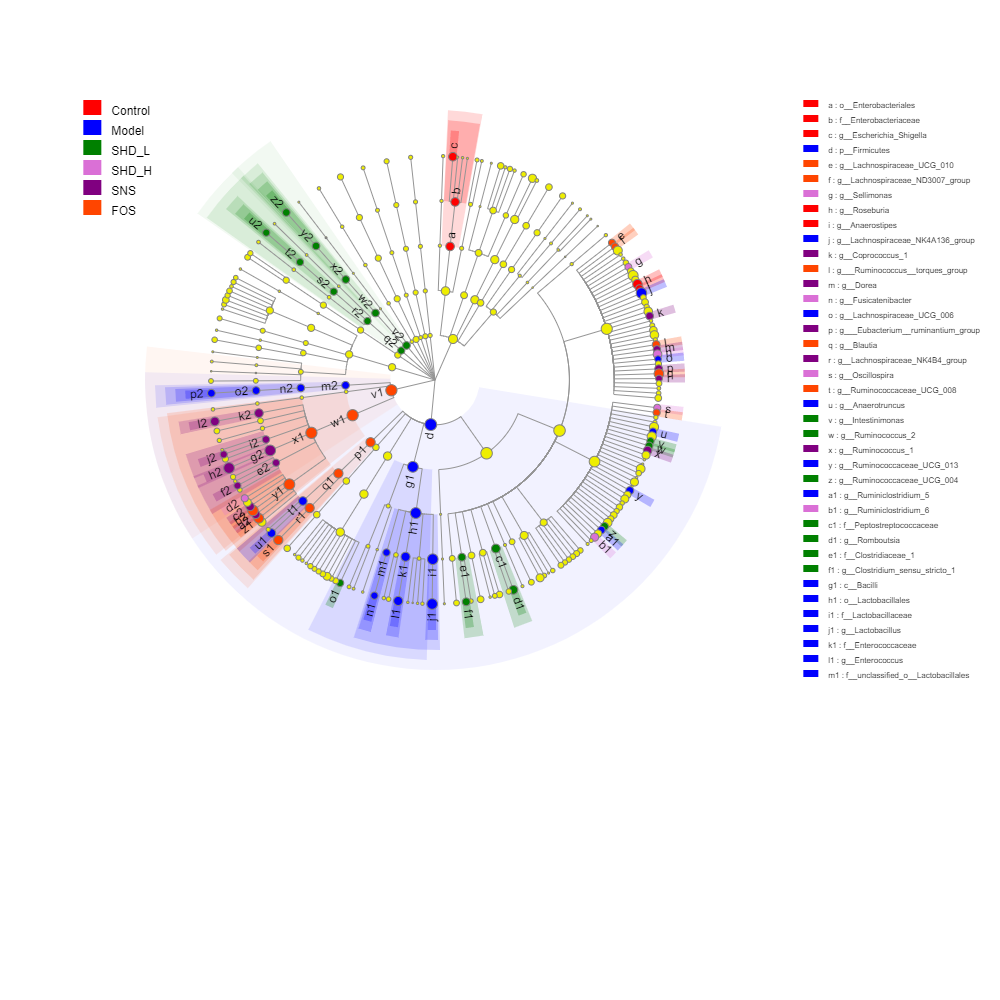

Supplement: Supplementary file 2 [file DataSheet4.ZIP › Supplementary_Material-original data2/FIGURE5/Figure 5I.png]

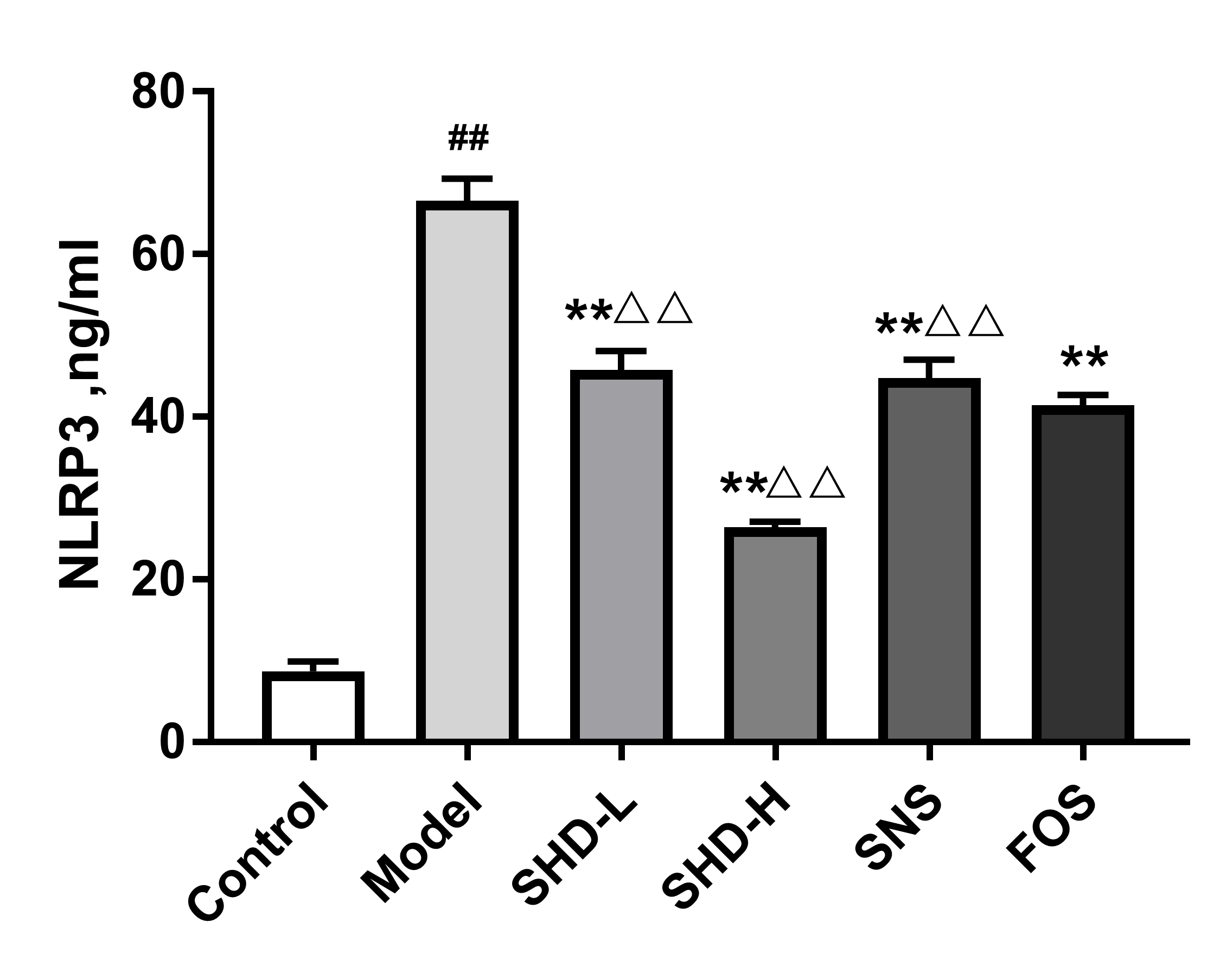

Supplement: Supplementary file 2 [file DataSheet4.ZIP › Supplementary_Material-original data2/FIGURE6/Figures 6A-D(Serum-Elisa)/Figure6A-Serum-NLRP3-Elisa.tif]

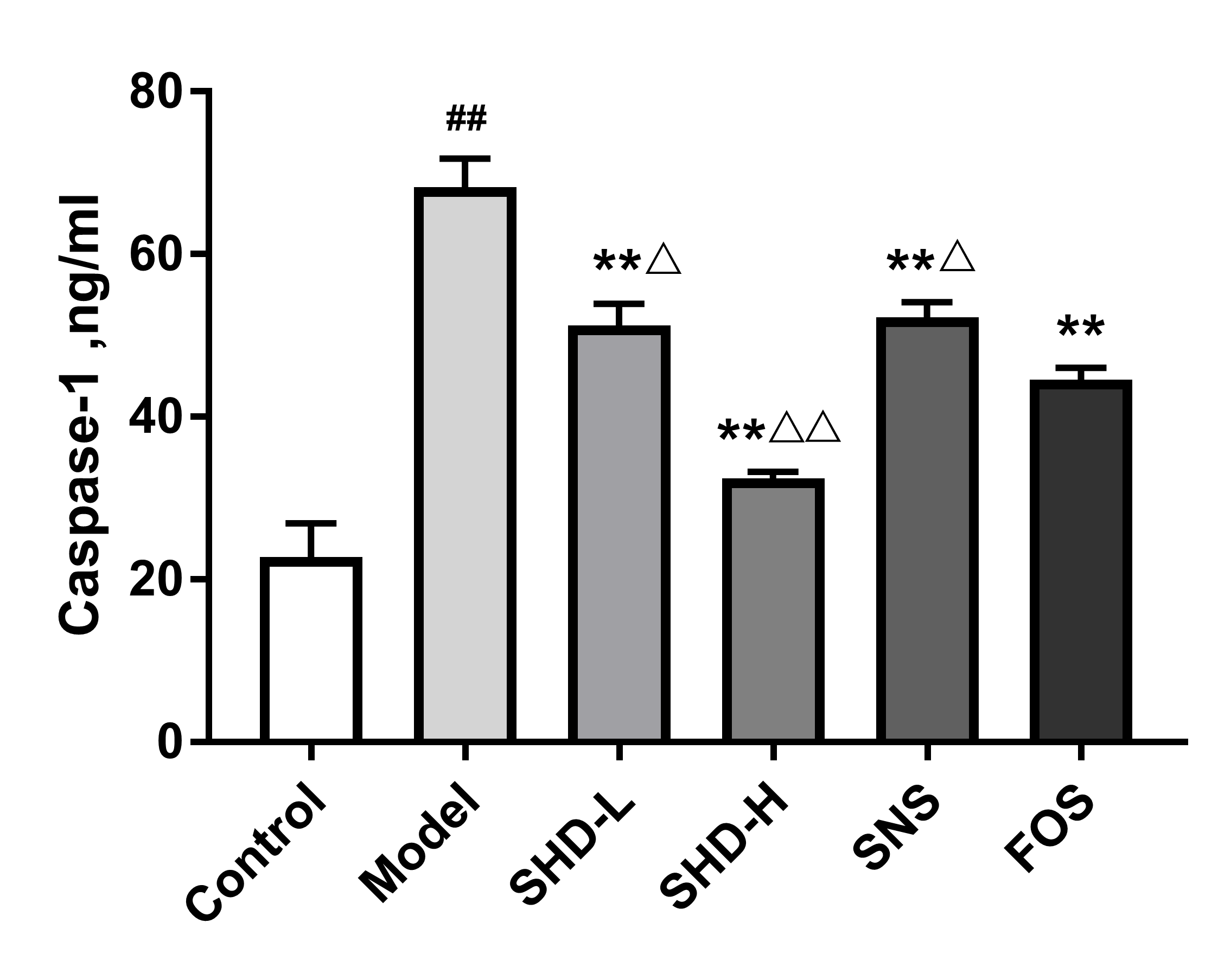

Supplement: Supplementary file 2 [file DataSheet4.ZIP › Supplementary_Material-original data2/FIGURE6/Figures 6A-D(Serum-Elisa)/Figure6B-Serum-Caspase1-Elisa.tif]

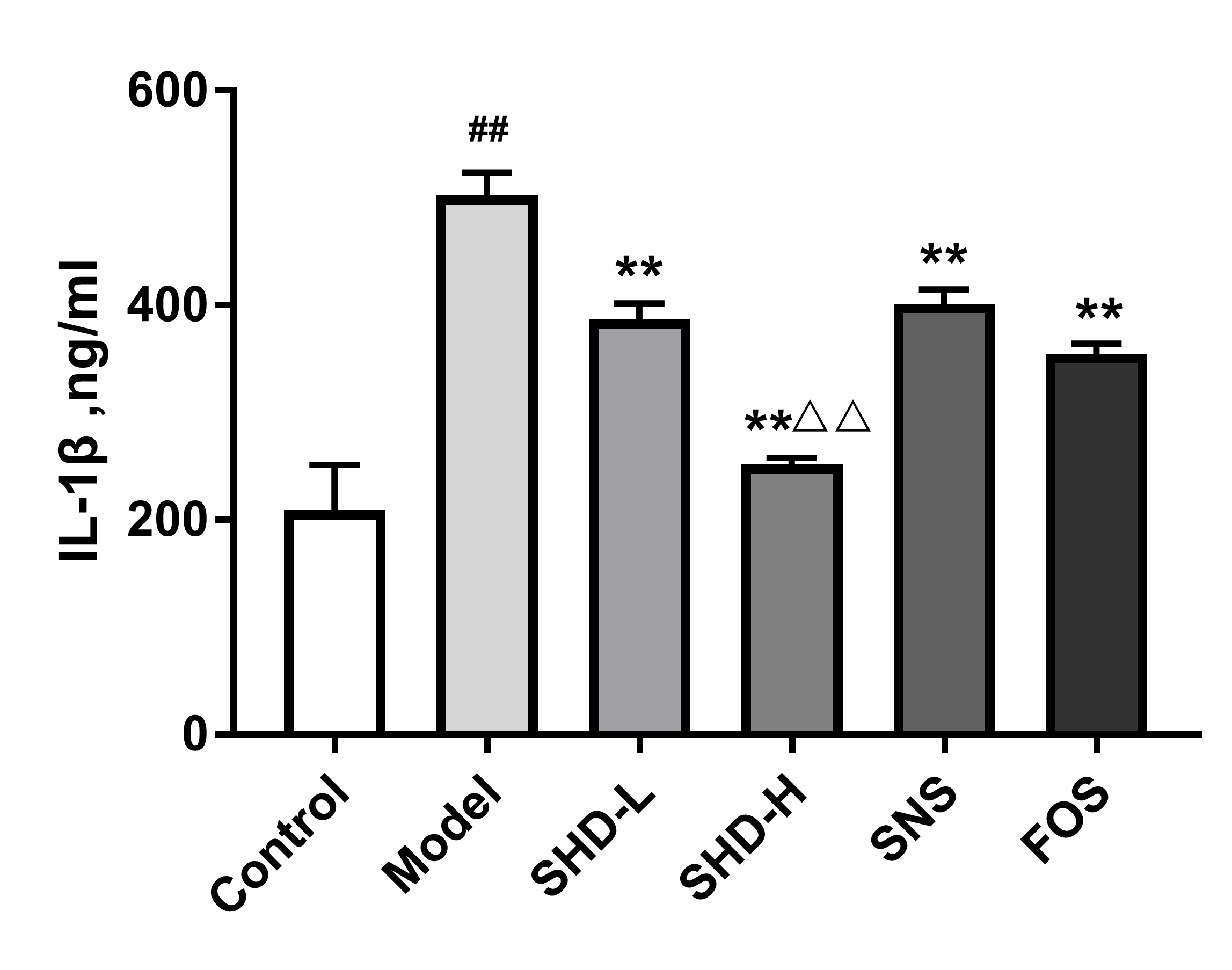

Supplement: Supplementary file 2 [file DataSheet4.ZIP › Supplementary_Material-original data2/FIGURE6/Figures 6A-D(Serum-Elisa)/Figure6C-Serum-IL-1β-Elisa.tif]

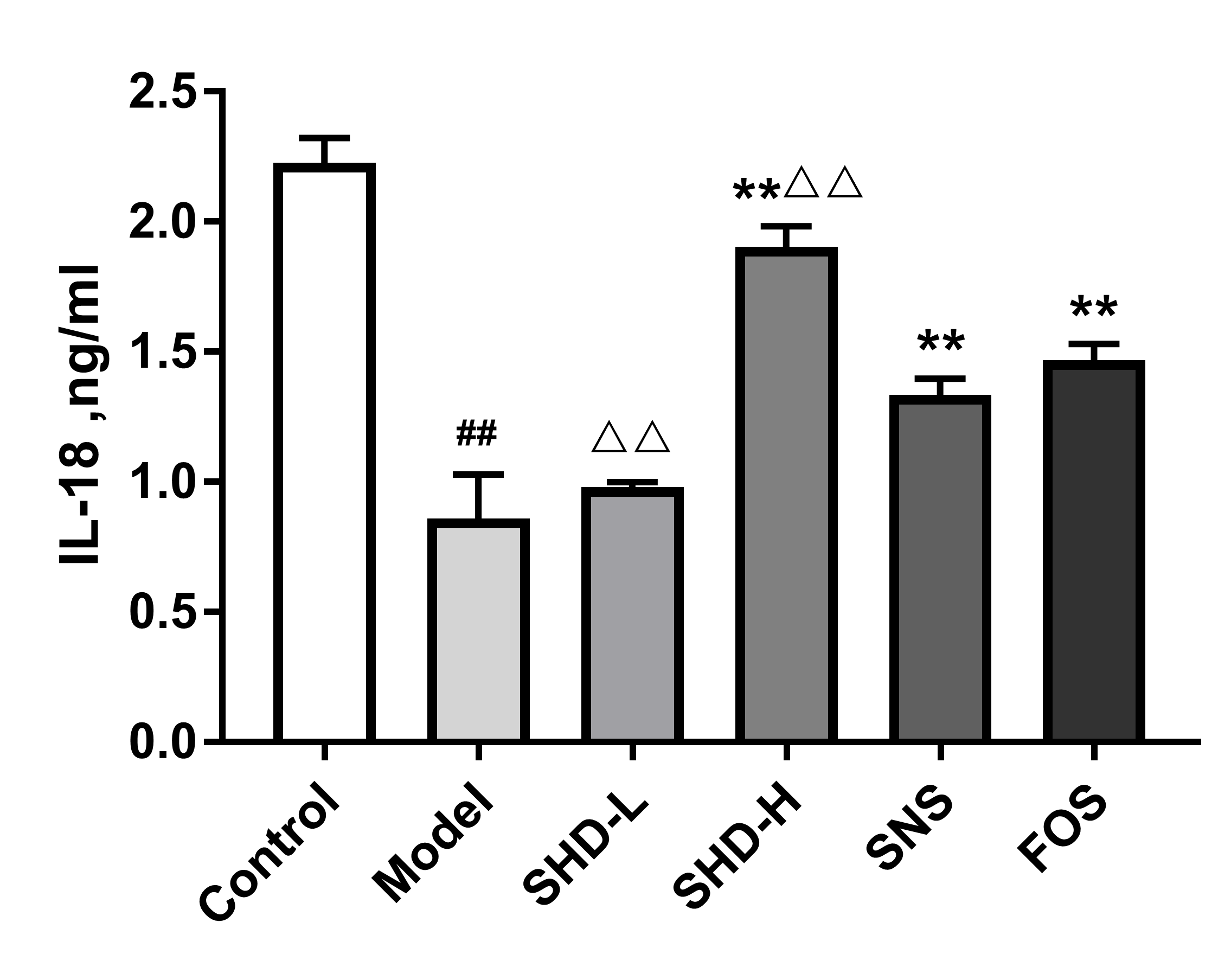

Supplement: Supplementary file 2 [file DataSheet4.ZIP › Supplementary_Material-original data2/FIGURE6/Figures 6A-D(Serum-Elisa)/Figure6D-Serum-IL-18-Elisa.tif]

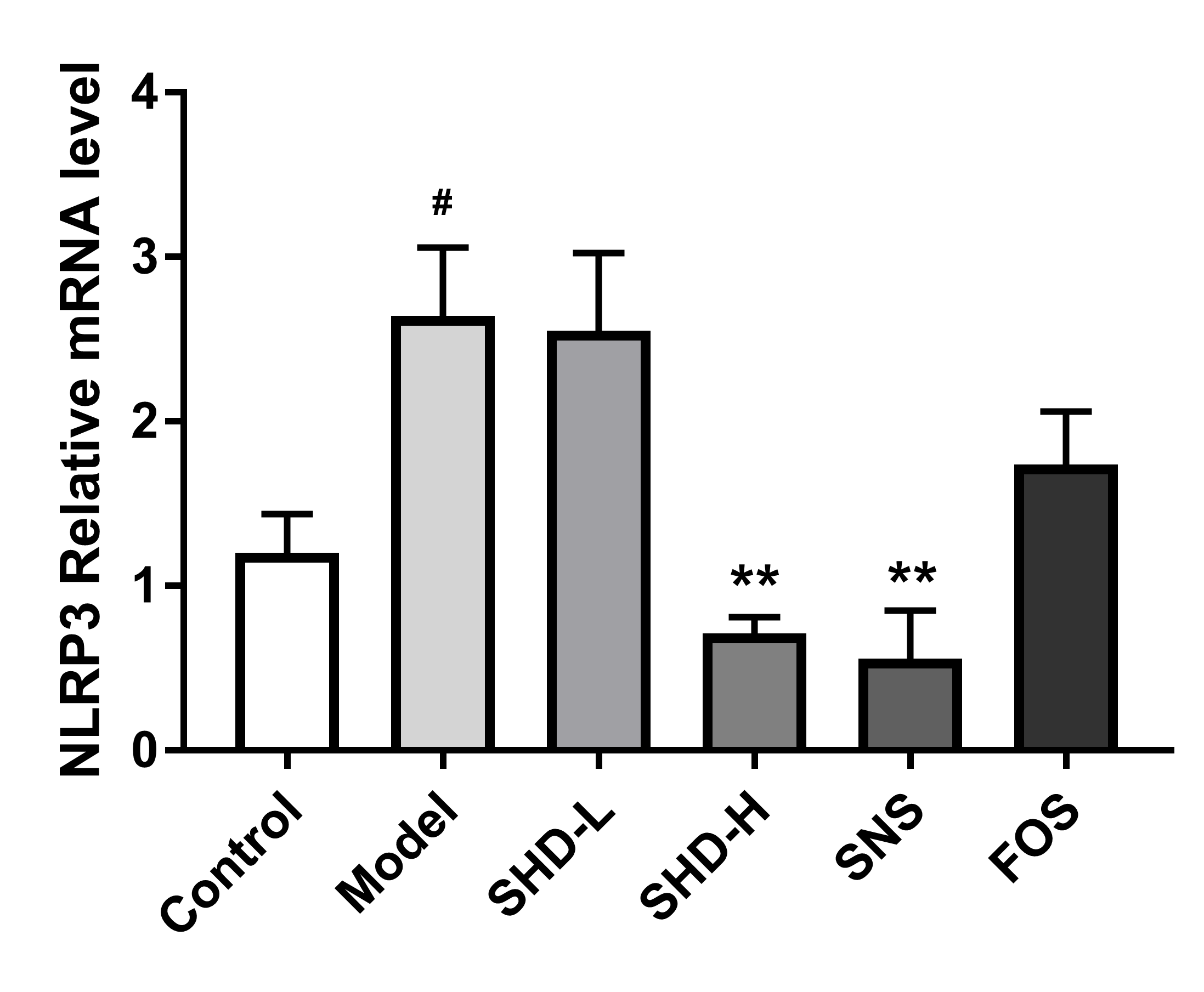

Supplement: Supplementary file 2 [file DataSheet4.ZIP › Supplementary_Material-original data2/FIGURE6/Figures 6E-I(Cecal-PCR )/Figure6E-Cecal-NLRP3-PCR.tif]

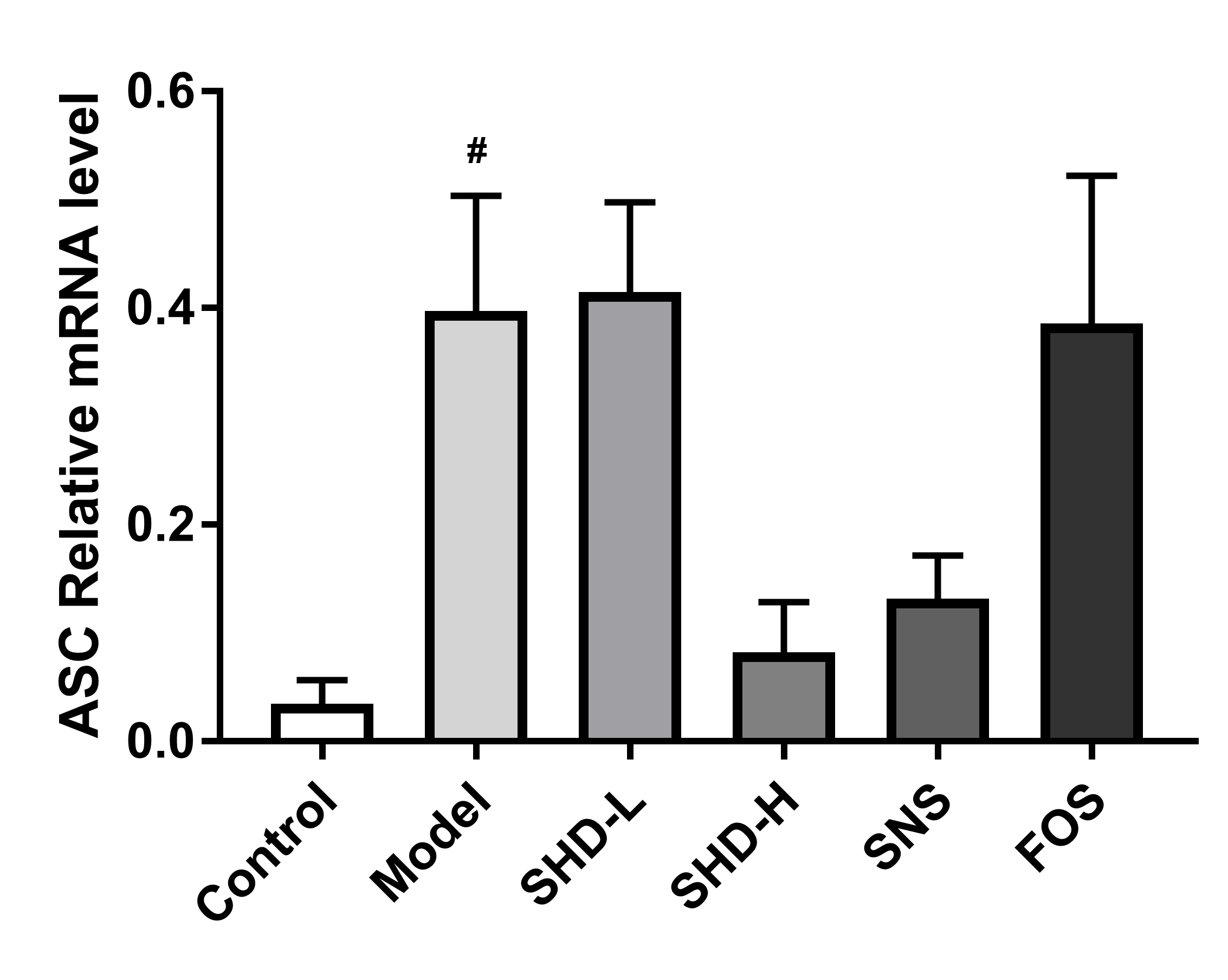

Supplement: Supplementary file 2 [file DataSheet4.ZIP › Supplementary_Material-original data2/FIGURE6/Figures 6E-I(Cecal-PCR )/Figure6F-Cecal-ASC-PCR.tif]

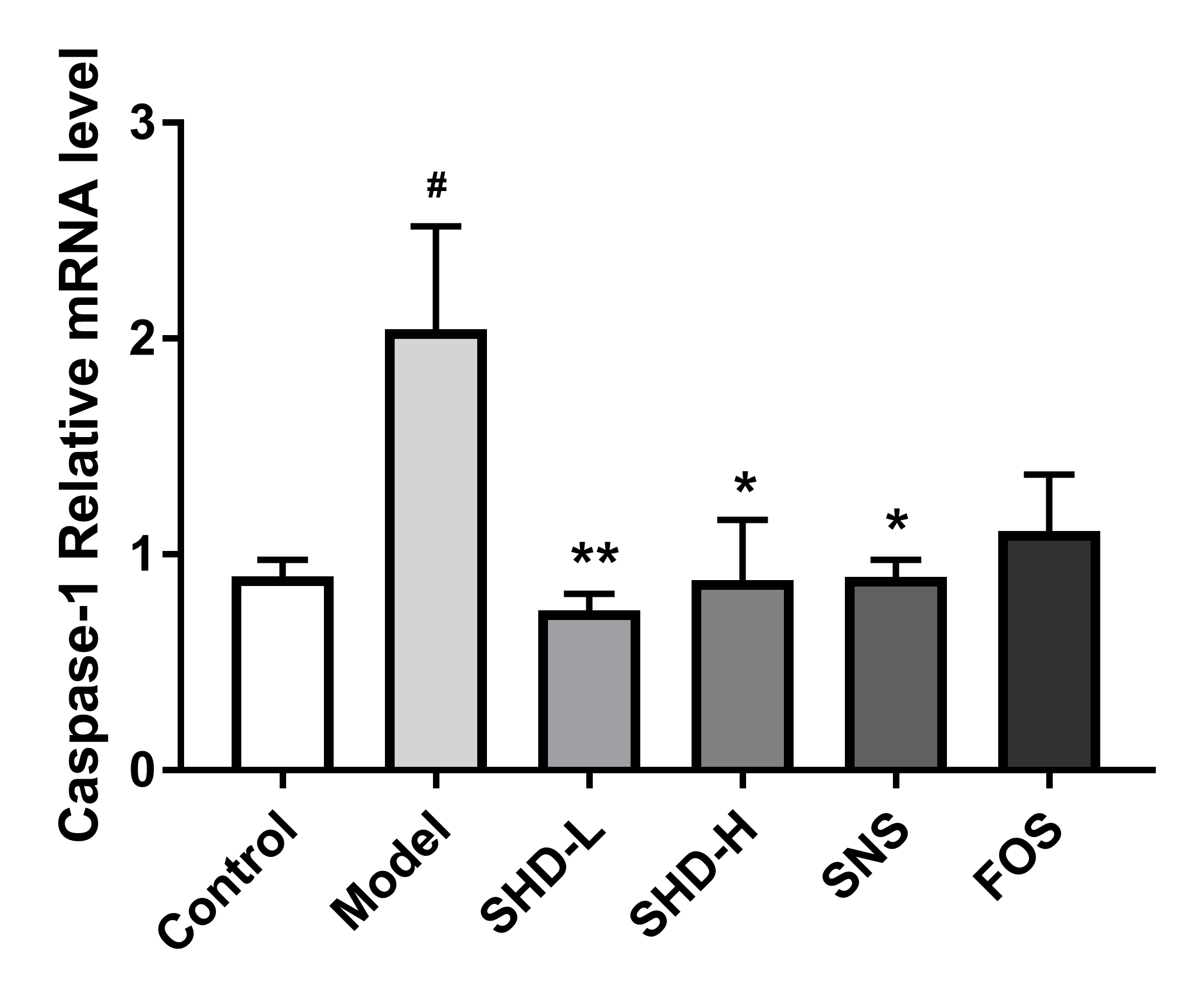

Supplement: Supplementary file 2 [file DataSheet4.ZIP › Supplementary_Material-original data2/FIGURE6/Figures 6E-I(Cecal-PCR )/Figure6G-Cecal-Caspase1-PCR.tif]

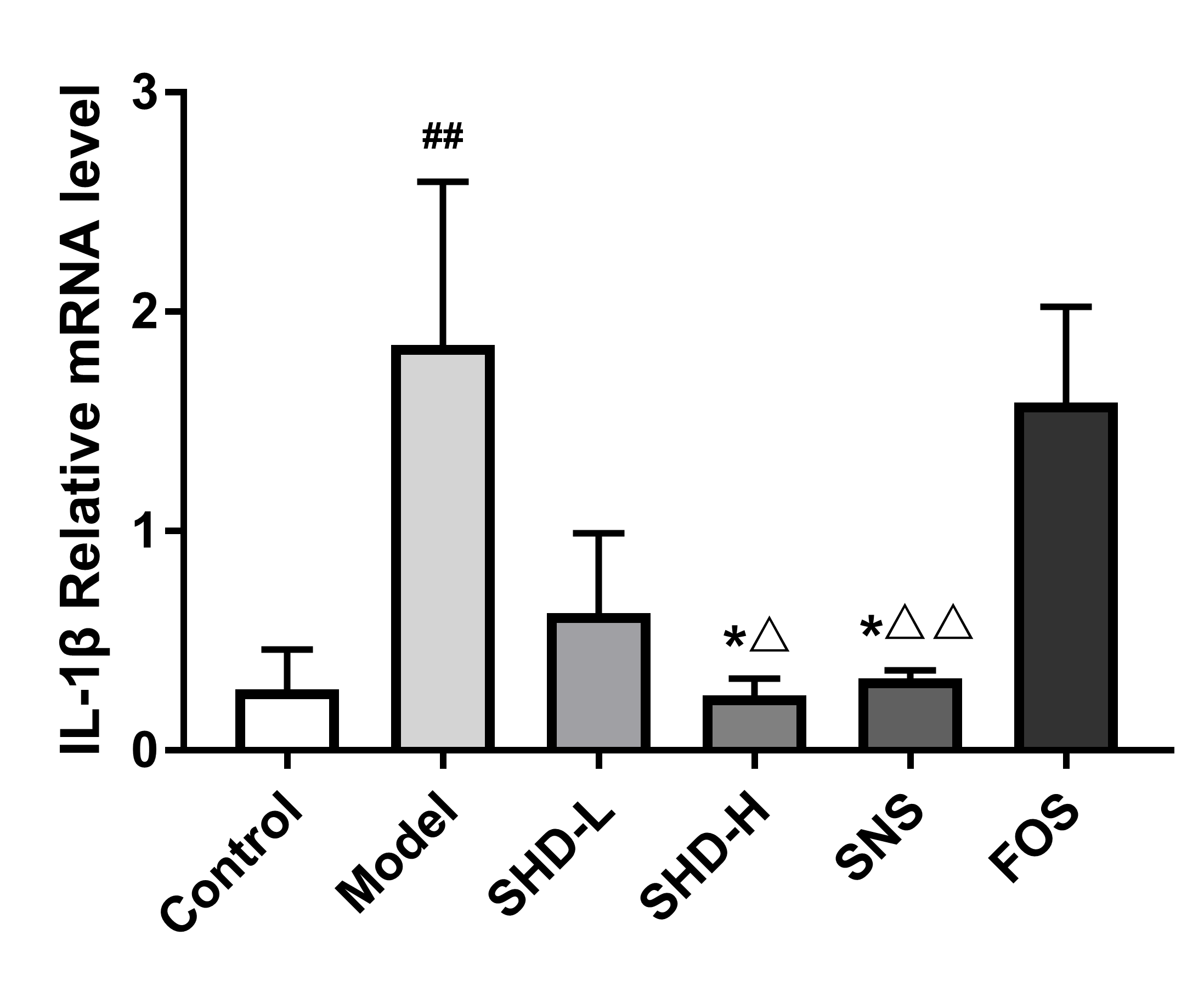

Supplement: Supplementary file 2 [file DataSheet4.ZIP › Supplementary_Material-original data2/FIGURE6/Figures 6E-I(Cecal-PCR )/Figure6H-Cecal-IL-1β-PCR.tif]

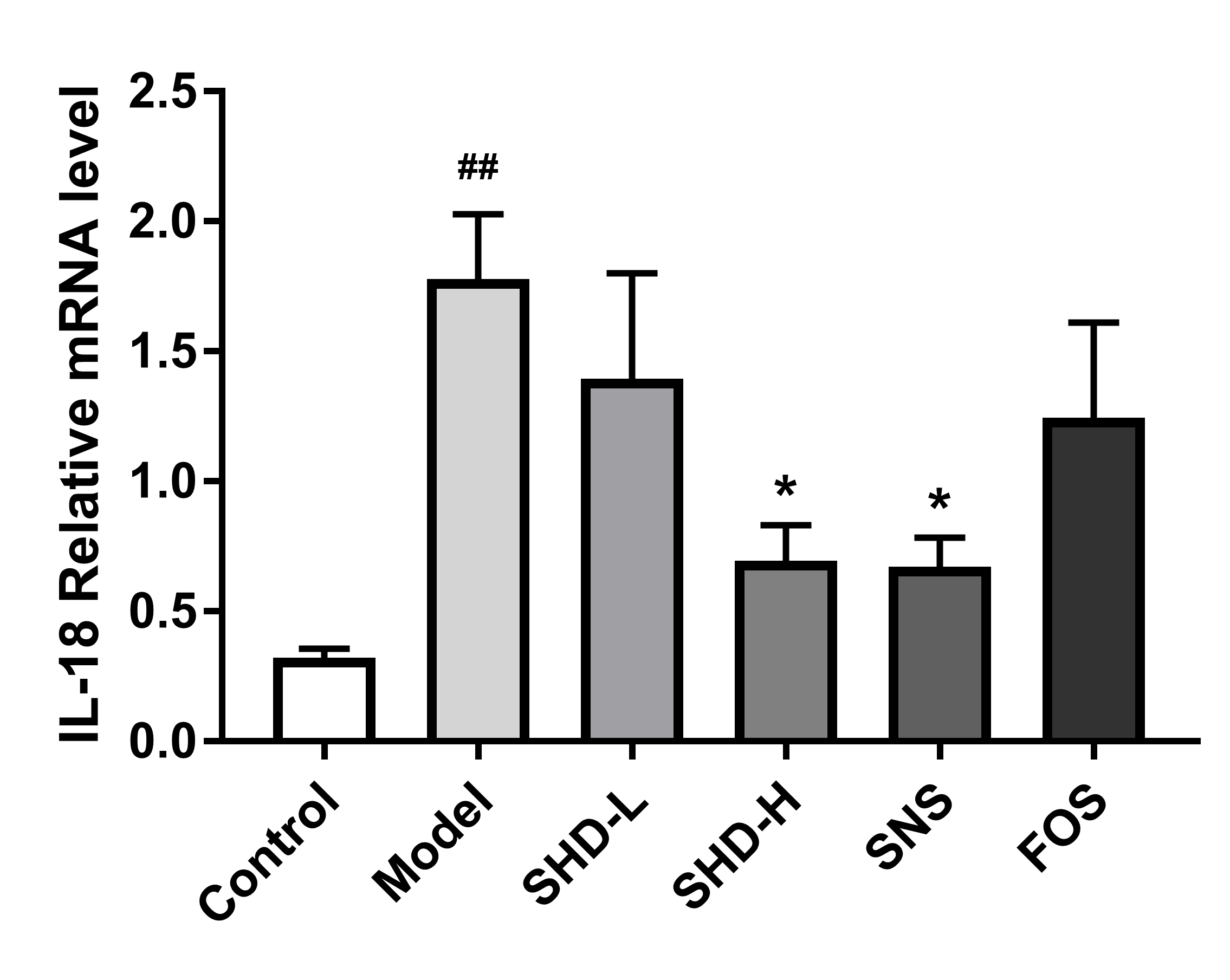

Supplement: Supplementary file 2 [file DataSheet4.ZIP › Supplementary_Material-original data2/FIGURE6/Figures 6E-I(Cecal-PCR )/Figure6I-Cecal-IL-18-PCR.tif]

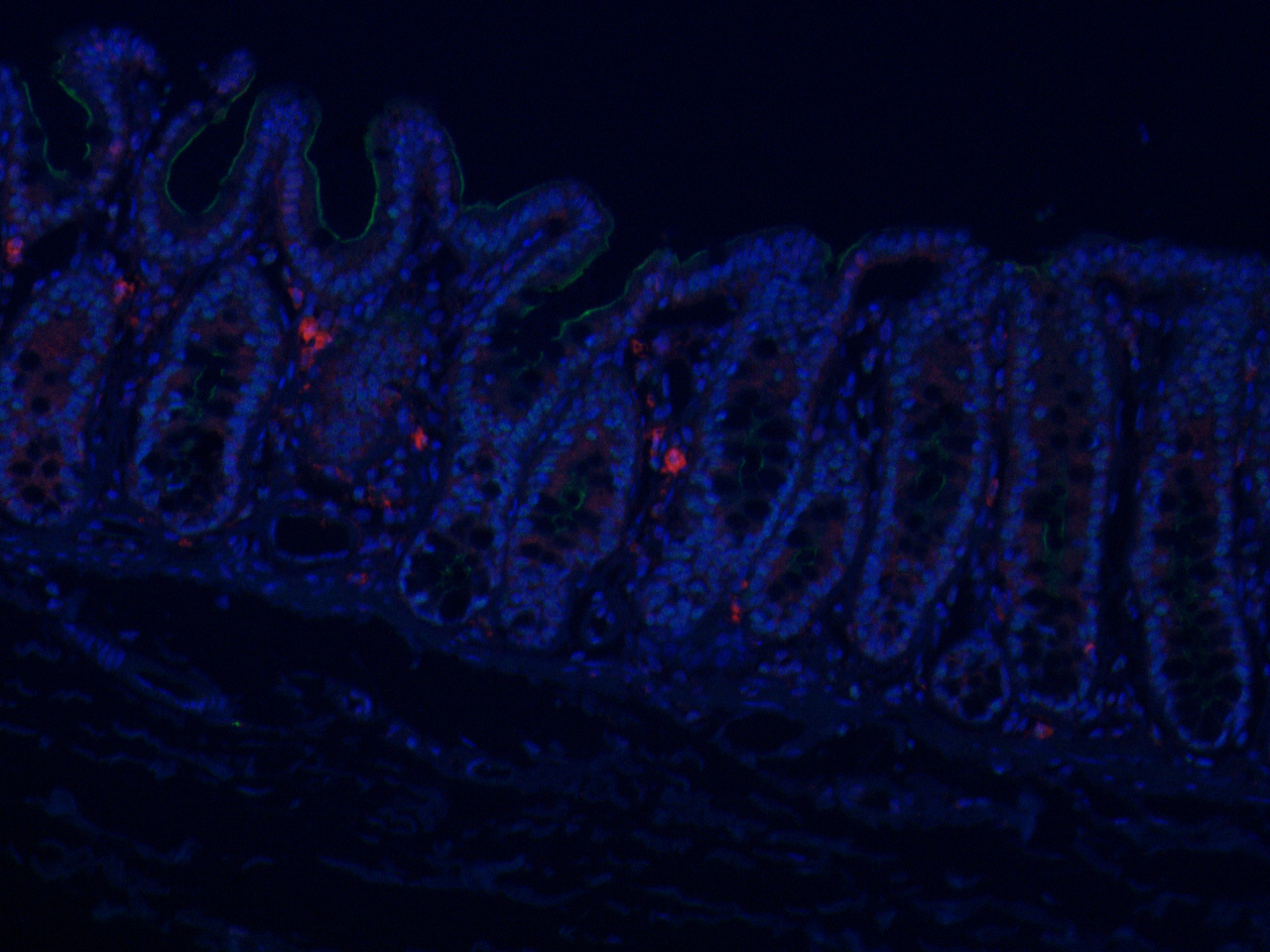

Supplement: Supplementary file 2 [file DataSheet4.ZIP › Supplementary_Material-original data2/FIGURE6/Figures 6J-K(Cecal-IF×200 )/Figure6-K-NLRP3-Caspase-1/Control/CM1-9 NLRP3(绿)+caspase1(红) 200-1 2 3.jpg]

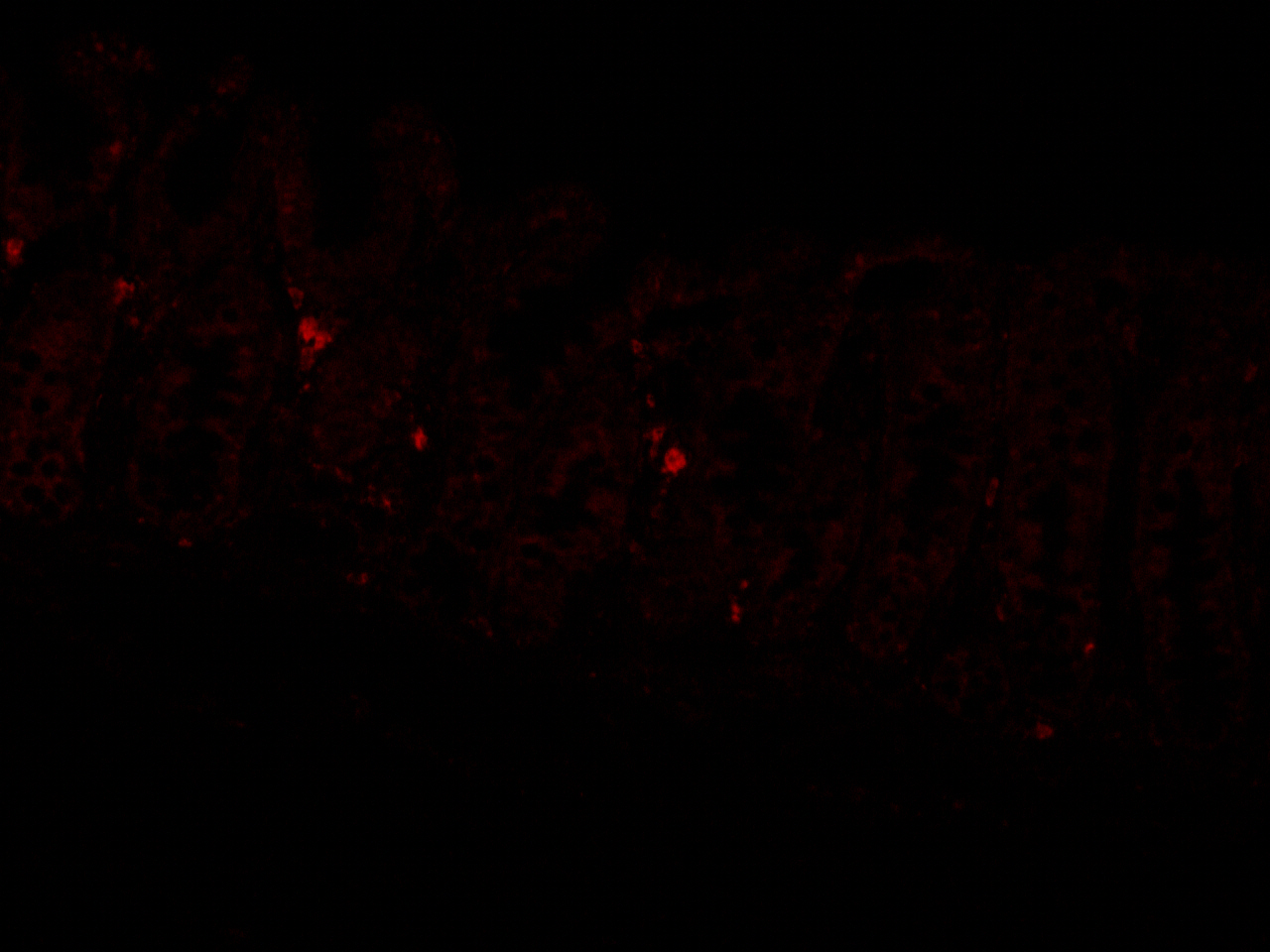

Supplement: Supplementary file 2 [file DataSheet4.ZIP › Supplementary_Material-original data2/FIGURE6/Figures 6J-K(Cecal-IF×200 )/Figure6-K-NLRP3-Caspase-1/Control/CM1-9 NLRP3(绿)+caspase1(红) 200-1.jpg]

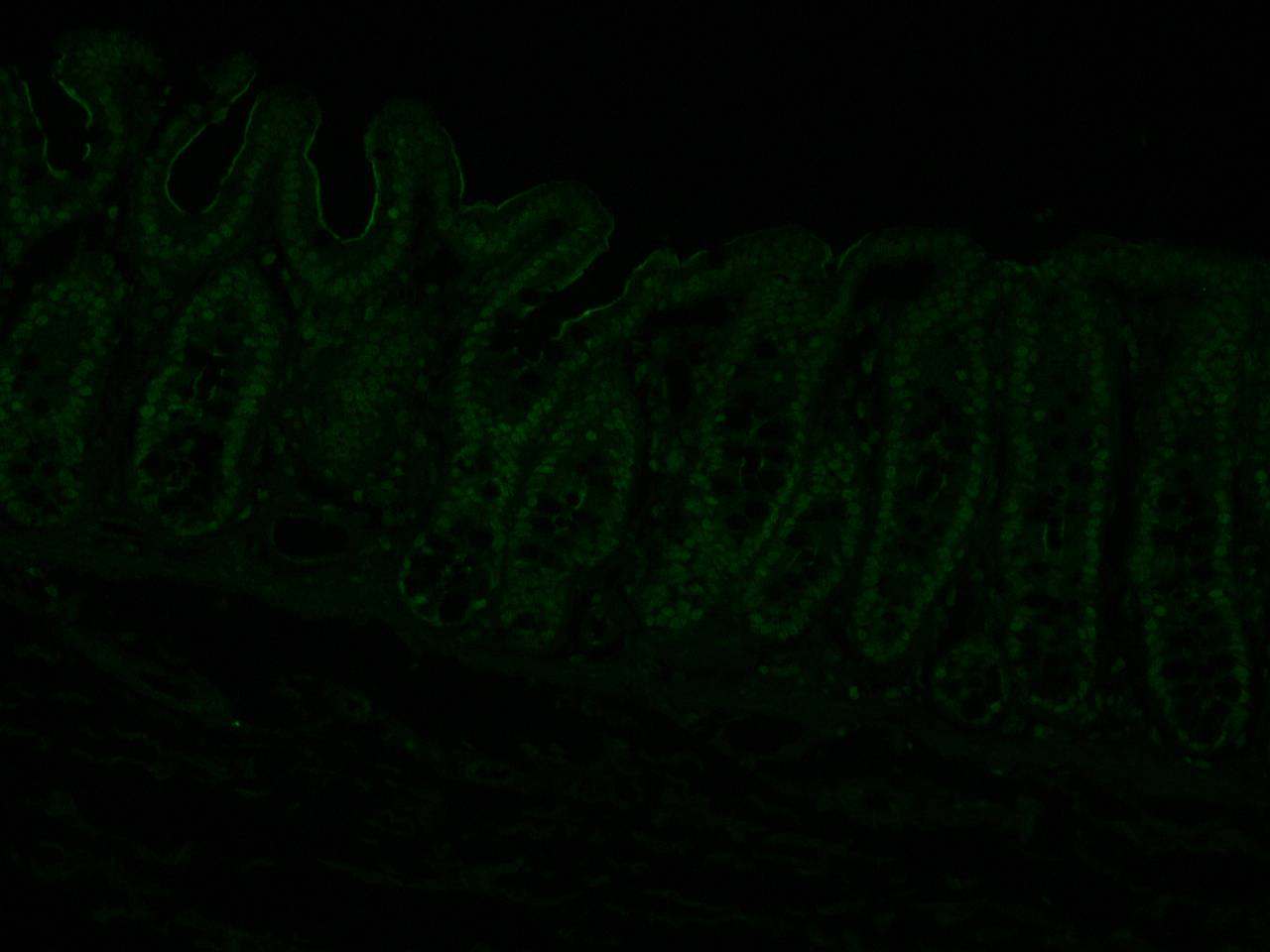

Supplement: Supplementary file 2 [file DataSheet4.ZIP › Supplementary_Material-original data2/FIGURE6/Figures 6J-K(Cecal-IF×200 )/Figure6-K-NLRP3-Caspase-1/Control/CM1-9 NLRP3(绿)+caspase1(红) 200-2.jpg]

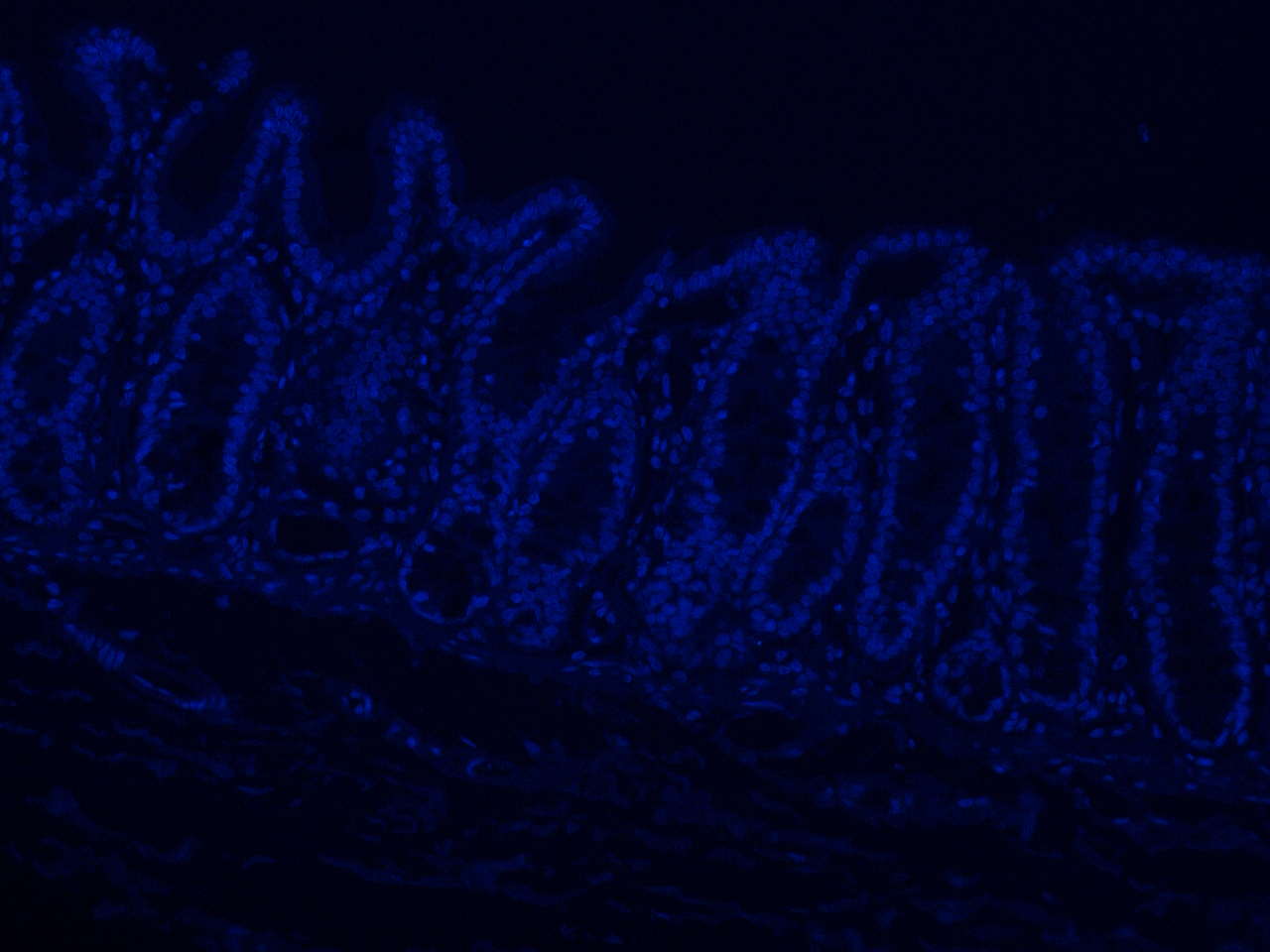

Supplement: Supplementary file 2 [file DataSheet4.ZIP › Supplementary_Material-original data2/FIGURE6/Figures 6J-K(Cecal-IF×200 )/Figure6-K-NLRP3-Caspase-1/Control/CM1-9 NLRP3(绿)+caspase1(红) 200-3.jpg]

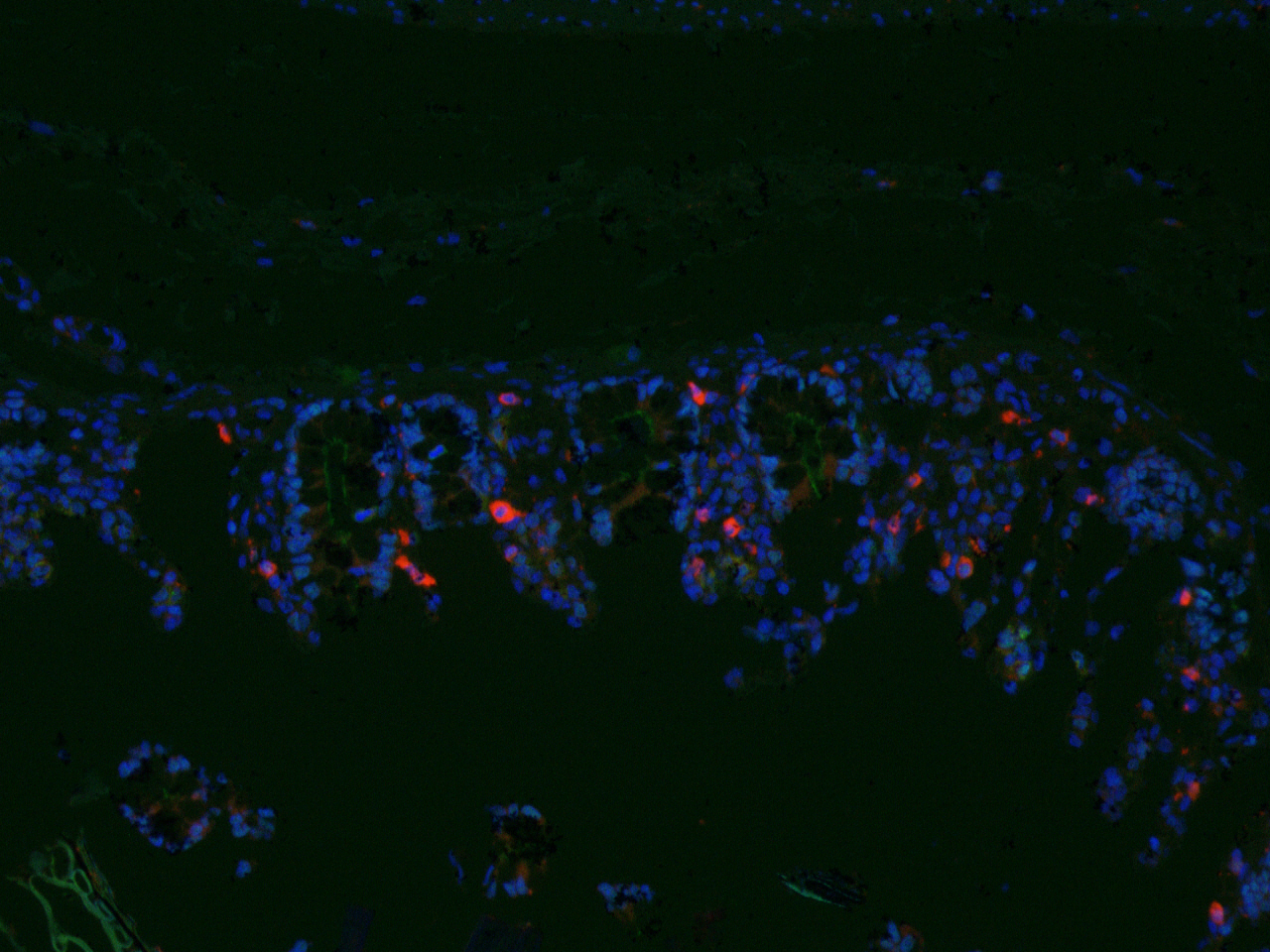

Supplement: Supplementary file 2 [file DataSheet4.ZIP › Supplementary_Material-original data2/FIGURE6/Figures 6J-K(Cecal-IF×200 )/Figure6-K-NLRP3-Caspase-1/FOS/盲 Y 2-1 caspase1(红)+NLRP3(绿) 200-7 8 9.jpg]

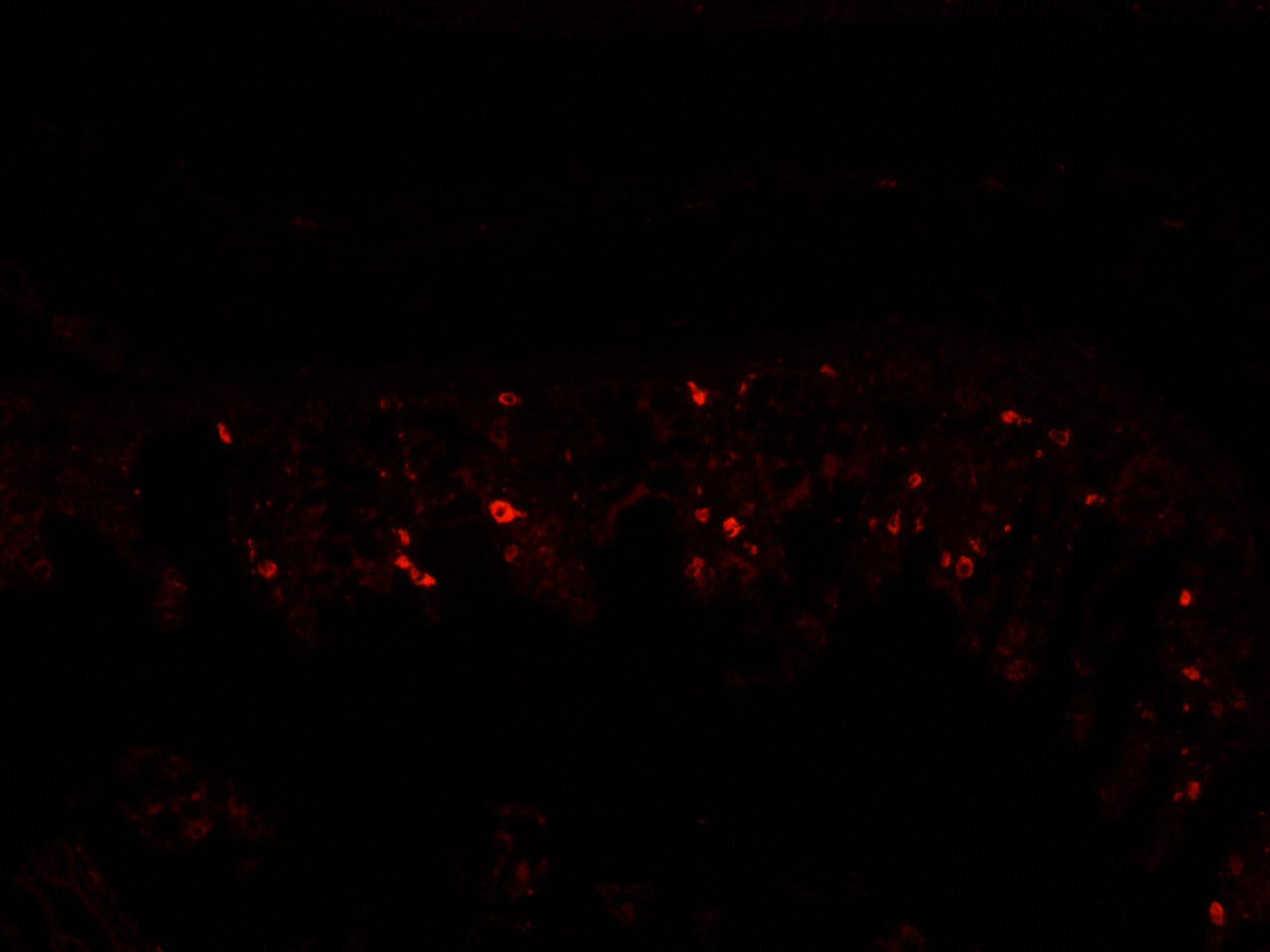

Supplement: Supplementary file 2 [file DataSheet4.ZIP › Supplementary_Material-original data2/FIGURE6/Figures 6J-K(Cecal-IF×200 )/Figure6-K-NLRP3-Caspase-1/FOS/盲 Y 2-1 caspase1(红)+NLRP3(绿) 200-7.jpg]

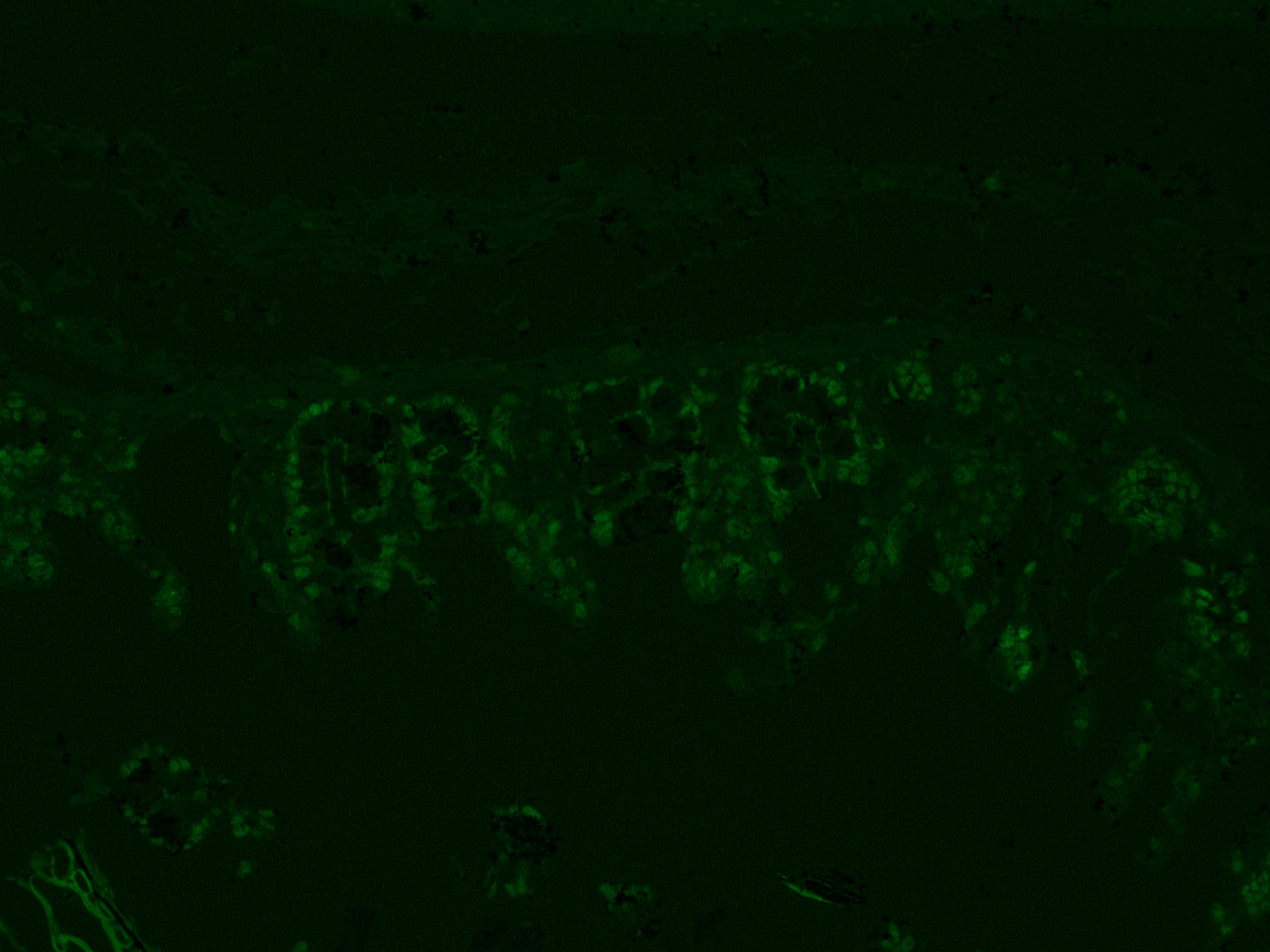

Supplement: Supplementary file 2 [file DataSheet4.ZIP › Supplementary_Material-original data2/FIGURE6/Figures 6J-K(Cecal-IF×200 )/Figure6-K-NLRP3-Caspase-1/FOS/盲 Y 2-1 caspase1(红)+NLRP3(绿) 200-8.jpg]

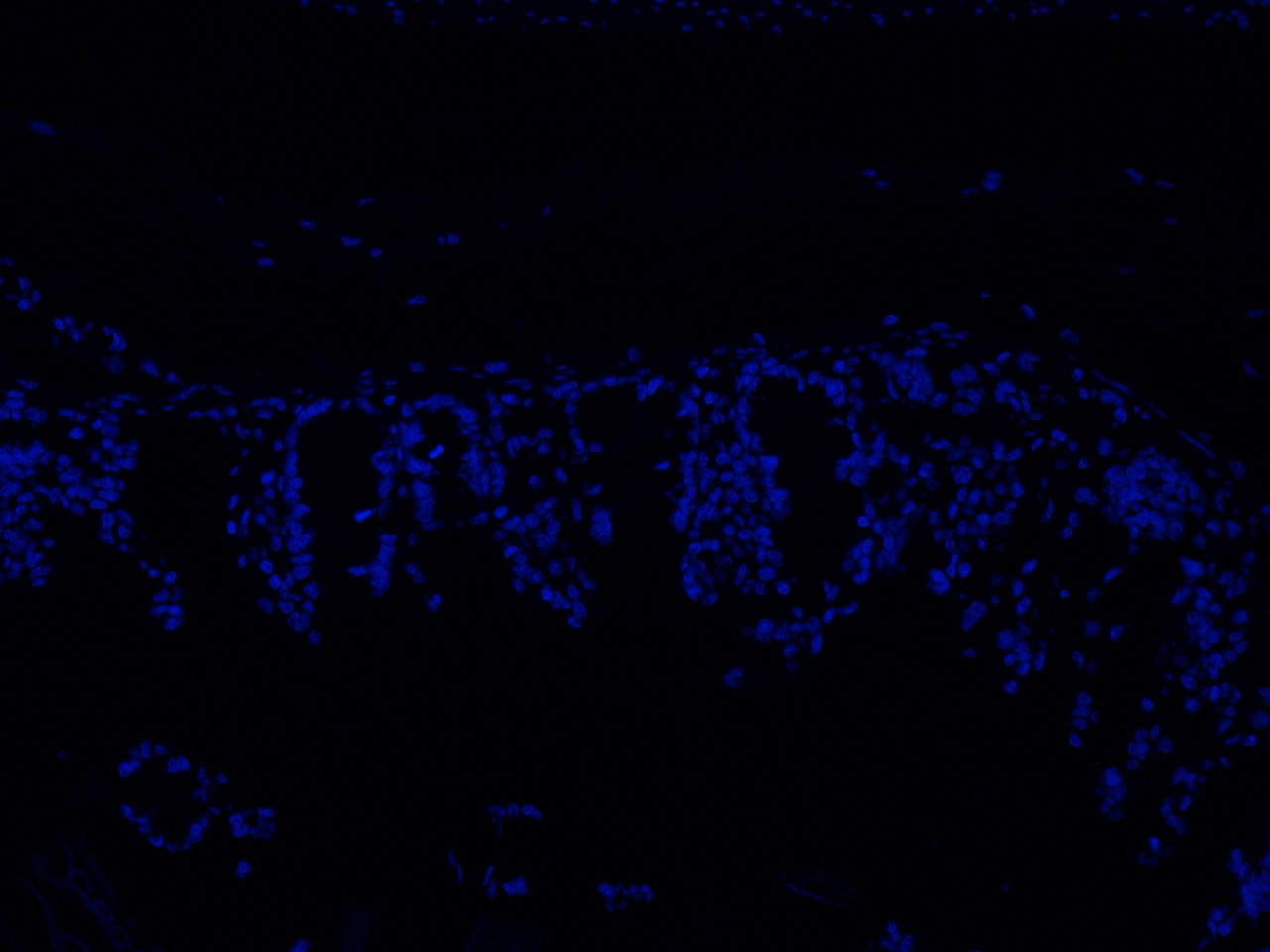

Supplement: Supplementary file 2 [file DataSheet4.ZIP › Supplementary_Material-original data2/FIGURE6/Figures 6J-K(Cecal-IF×200 )/Figure6-K-NLRP3-Caspase-1/FOS/盲 Y 2-1 caspase1(红)+NLRP3(绿) 200-9.jpg]

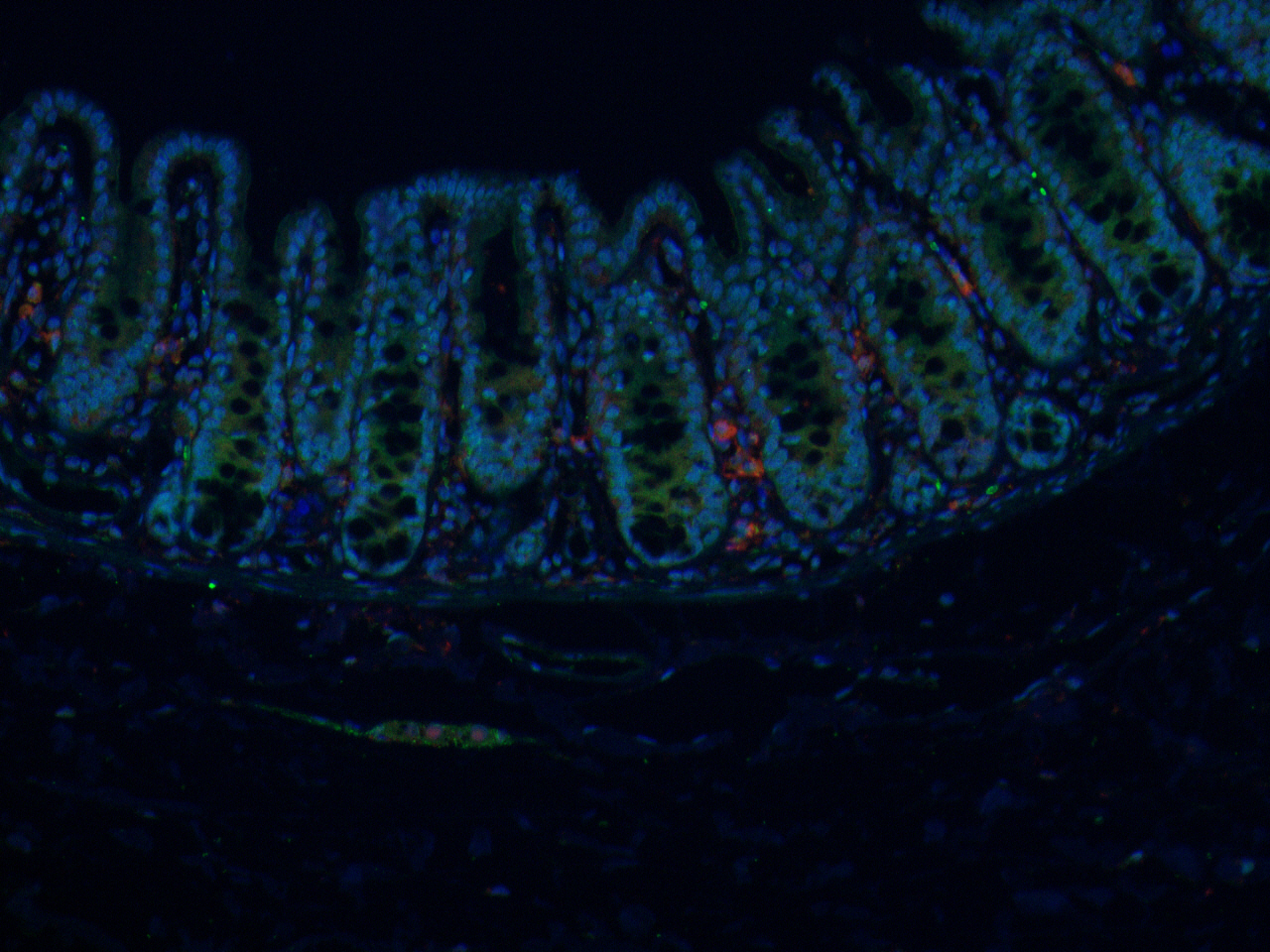

Supplement: Supplementary file 2 [file DataSheet4.ZIP › Supplementary_Material-original data2/FIGURE6/Figures 6J-K(Cecal-IF×200 )/Figure6-K-NLRP3-Caspase-1/Model/CM2-12 NLRP3(绿)+caspase1(红) 200-4 5 6.jpg]

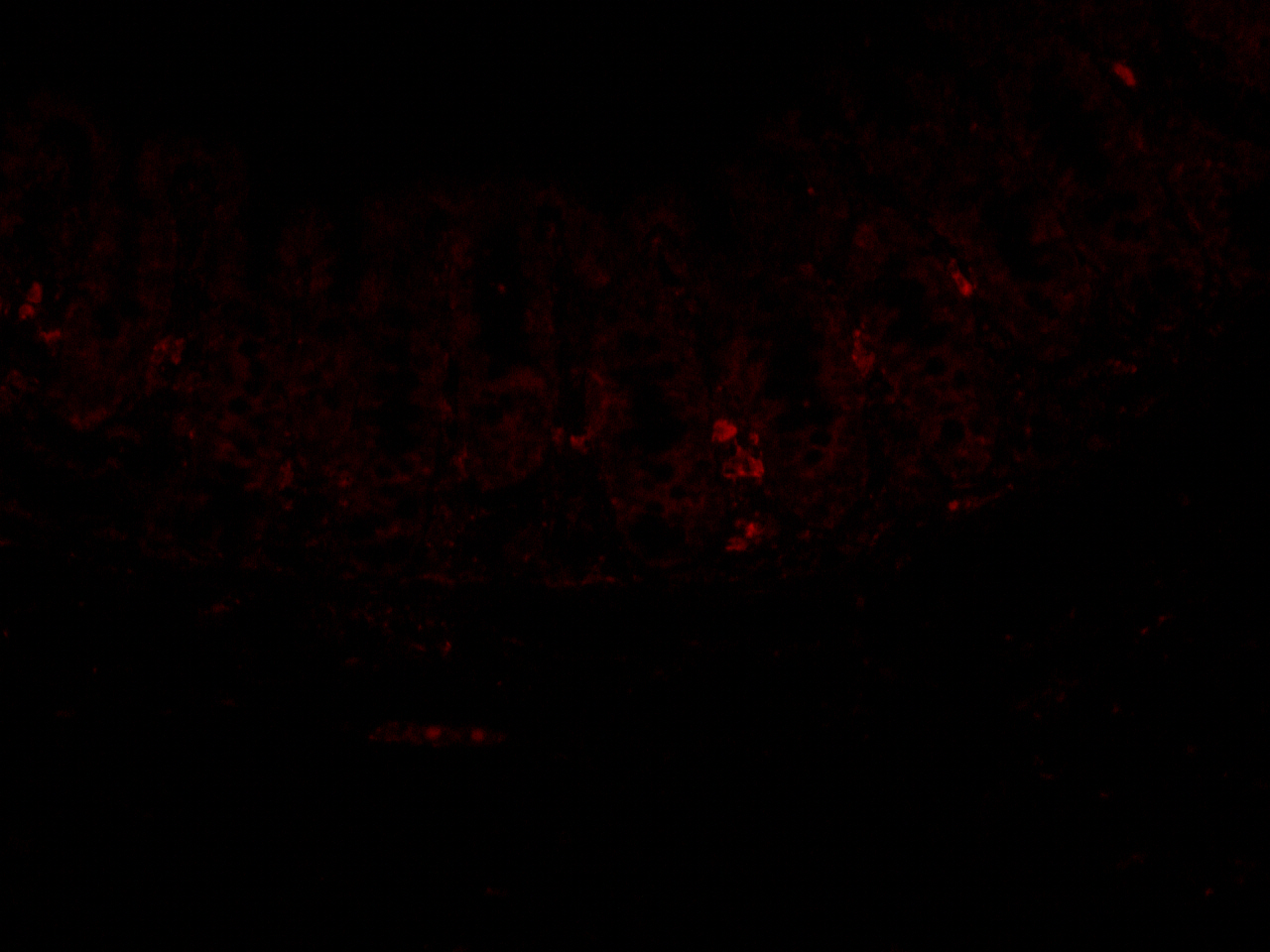

Supplement: Supplementary file 2 [file DataSheet4.ZIP › Supplementary_Material-original data2/FIGURE6/Figures 6J-K(Cecal-IF×200 )/Figure6-K-NLRP3-Caspase-1/Model/CM2-12 NLRP3(绿)+caspase1(红) 200-4.jpg]

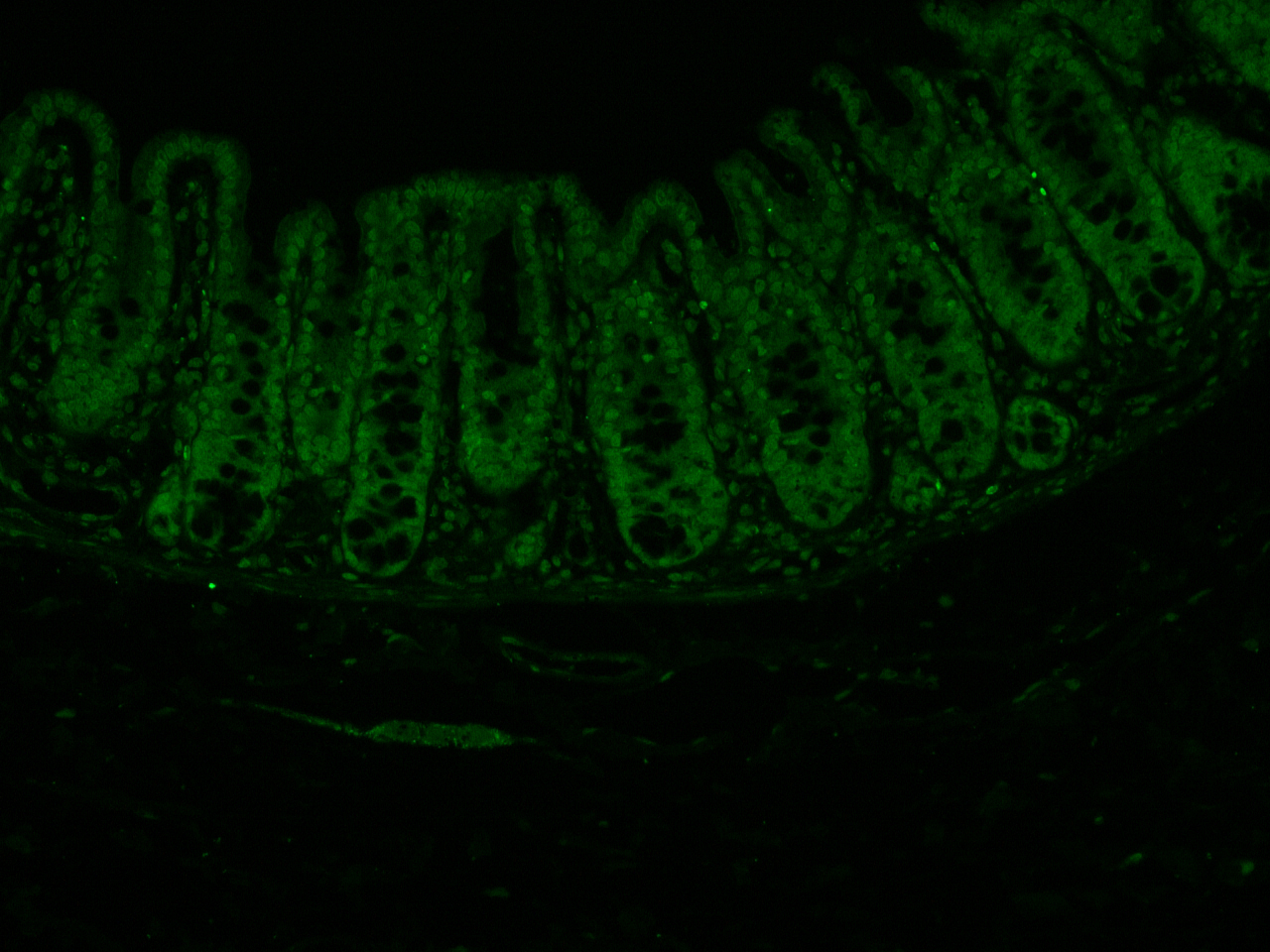

Supplement: Supplementary file 2 [file DataSheet4.ZIP › Supplementary_Material-original data2/FIGURE6/Figures 6J-K(Cecal-IF×200 )/Figure6-K-NLRP3-Caspase-1/Model/CM2-12 NLRP3(绿)+caspase1(红) 200-5.jpg]

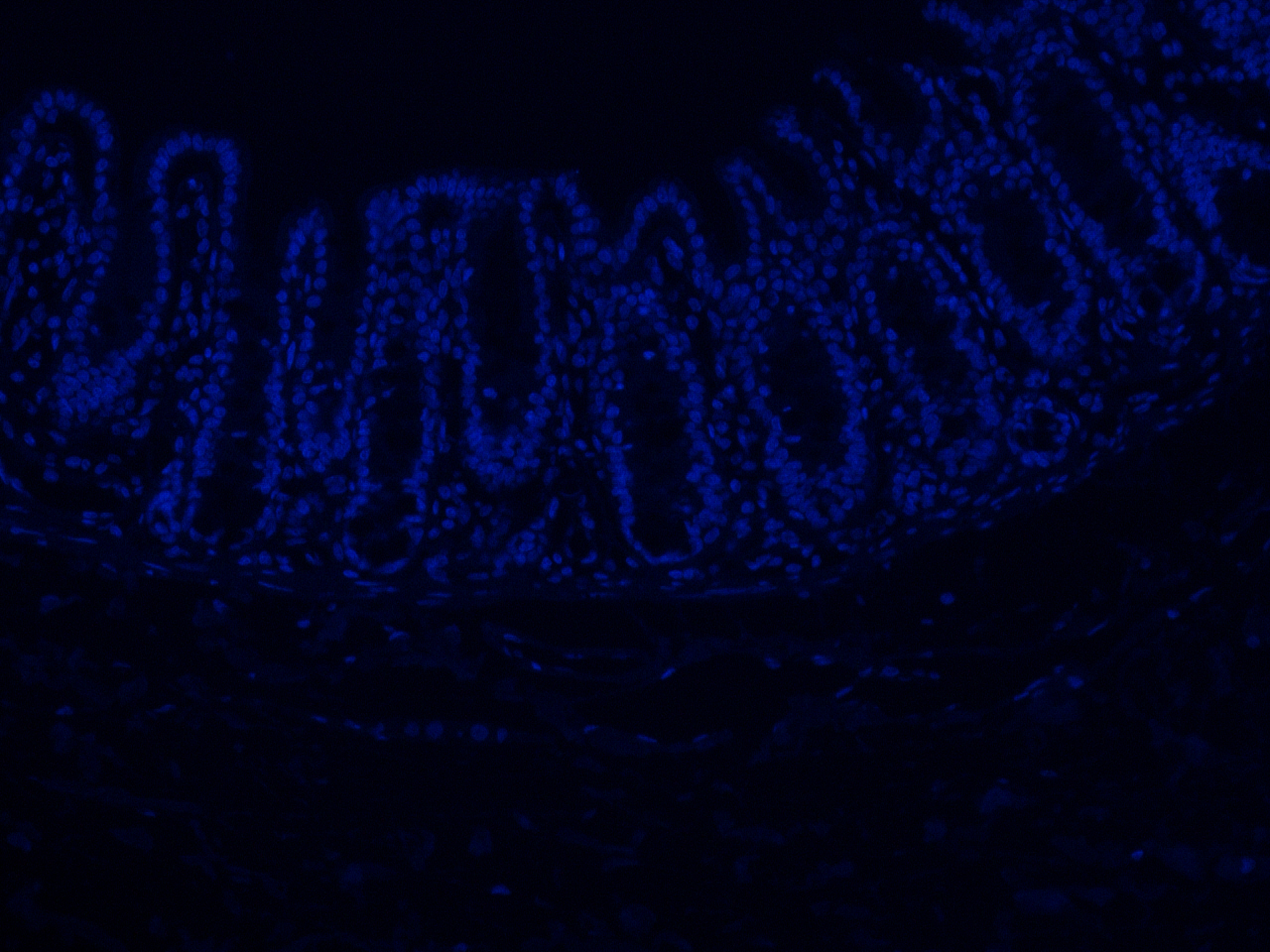

Supplement: Supplementary file 2 [file DataSheet4.ZIP › Supplementary_Material-original data2/FIGURE6/Figures 6J-K(Cecal-IF×200 )/Figure6-K-NLRP3-Caspase-1/Model/CM2-12 NLRP3(绿)+caspase1(红) 200-6.jpg]

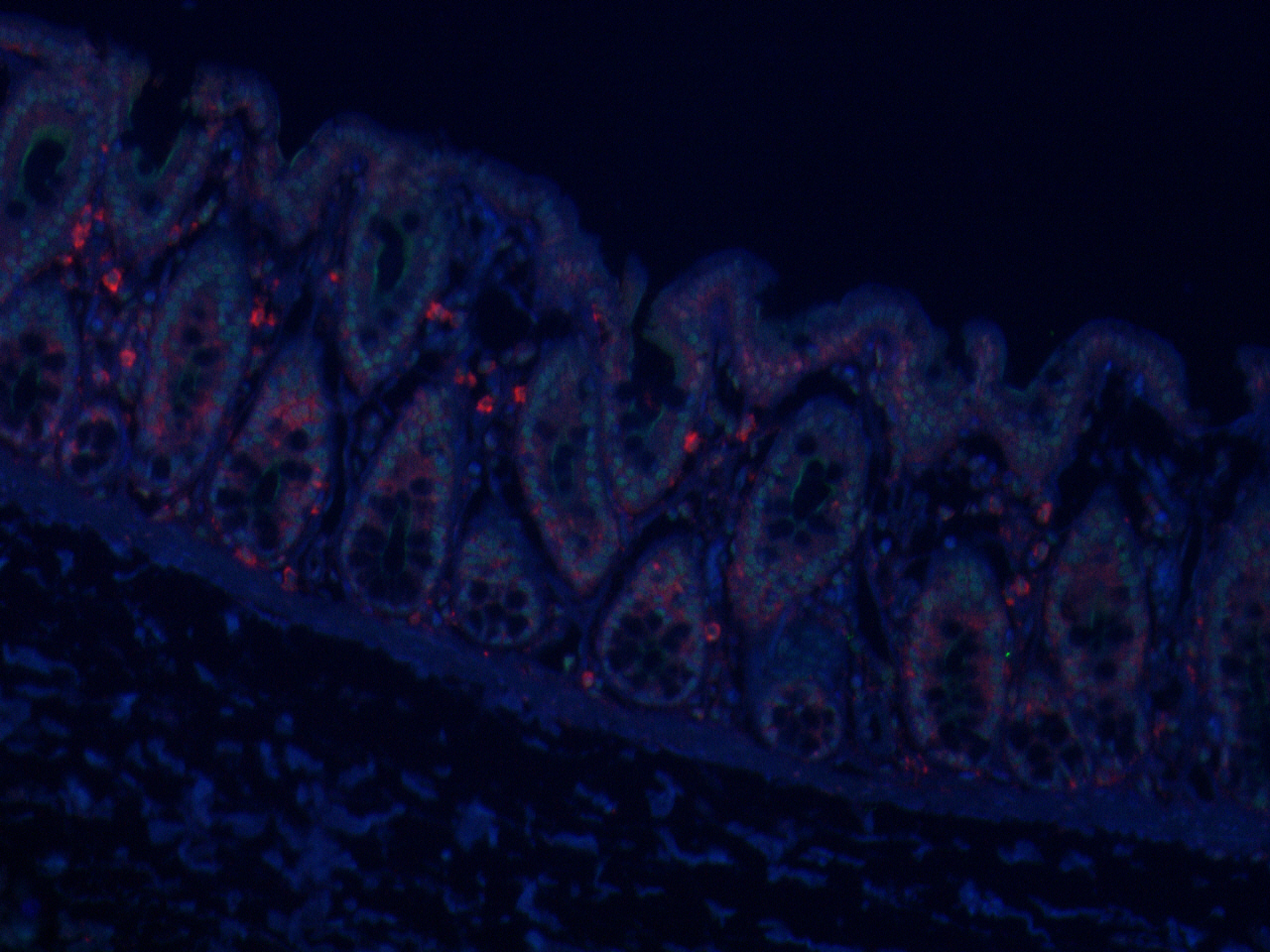

Supplement: Supplementary file 2 [file DataSheet4.ZIP › Supplementary_Material-original data2/FIGURE6/Figures 6J-K(Cecal-IF×200 )/Figure6-K-NLRP3-Caspase-1/SHD-H/CM4-11 NLRP3(绿)+caspase1(红) 200-4 5 6.jpg]

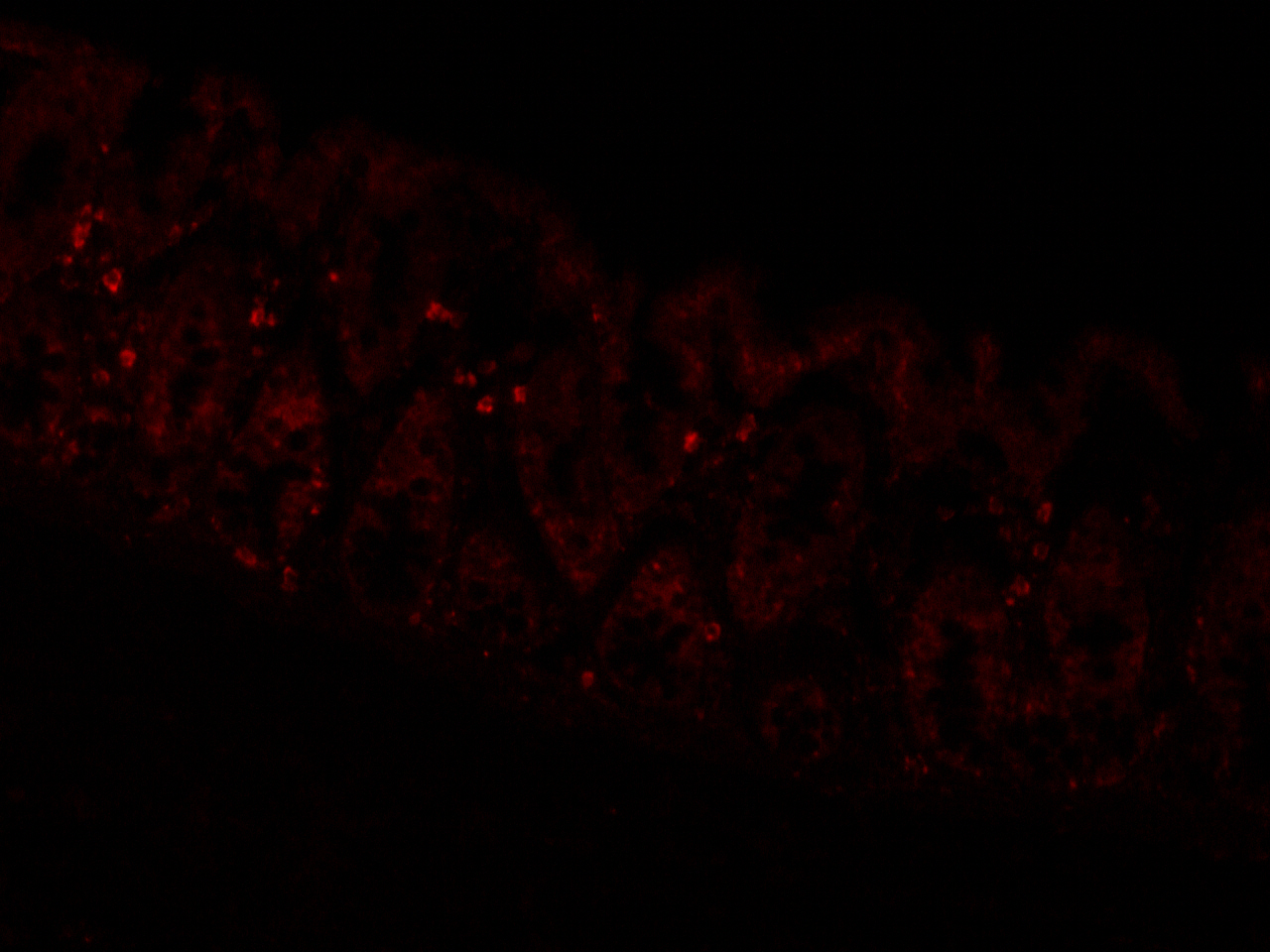

Supplement: Supplementary file 2 [file DataSheet4.ZIP › Supplementary_Material-original data2/FIGURE6/Figures 6J-K(Cecal-IF×200 )/Figure6-K-NLRP3-Caspase-1/SHD-H/CM4-11 NLRP3(绿)+caspase1(红) 200-4.jpg]

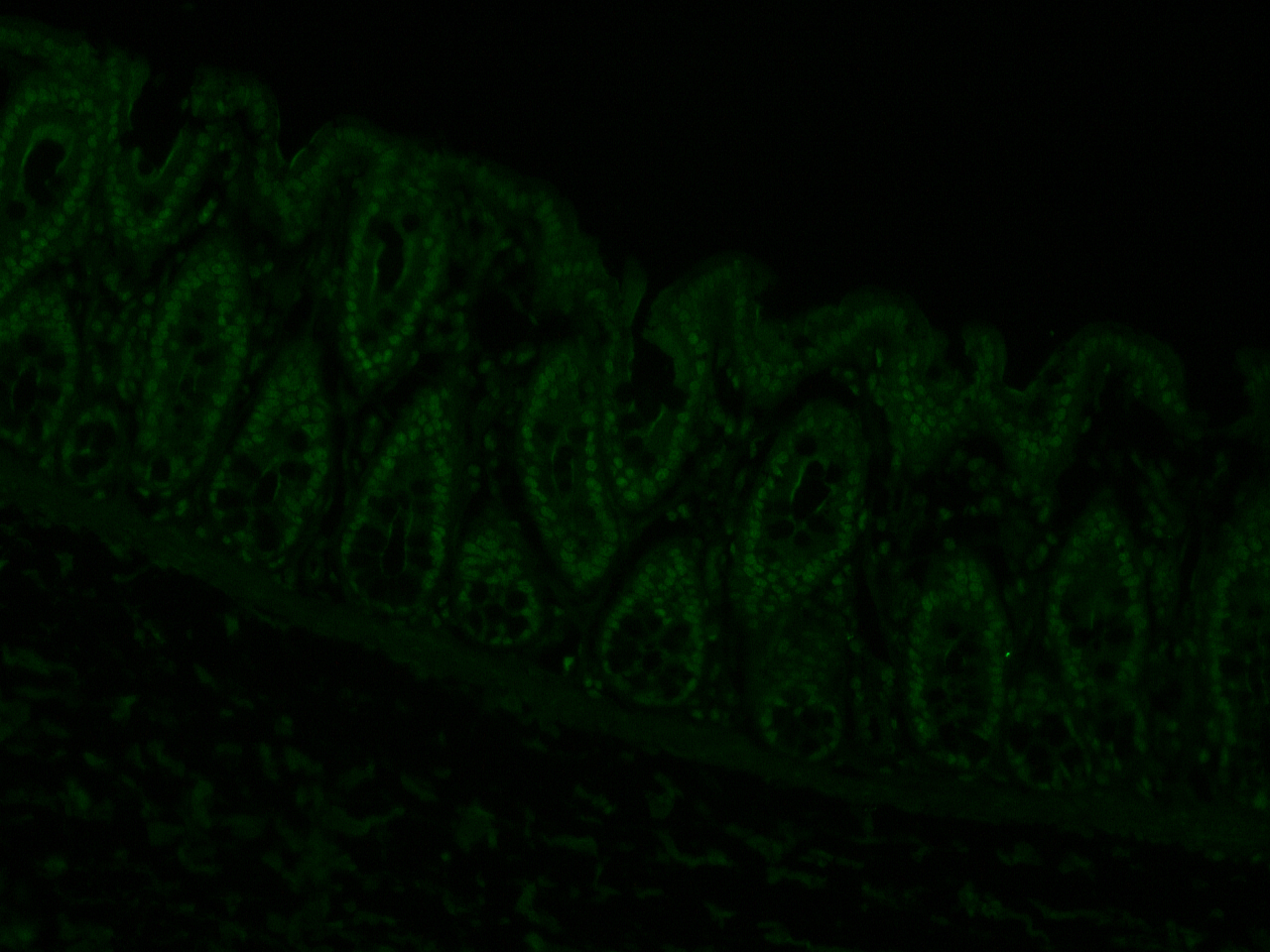

Supplement: Supplementary file 2 [file DataSheet4.ZIP › Supplementary_Material-original data2/FIGURE6/Figures 6J-K(Cecal-IF×200 )/Figure6-K-NLRP3-Caspase-1/SHD-H/CM4-11 NLRP3(绿)+caspase1(红) 200-5.jpg]

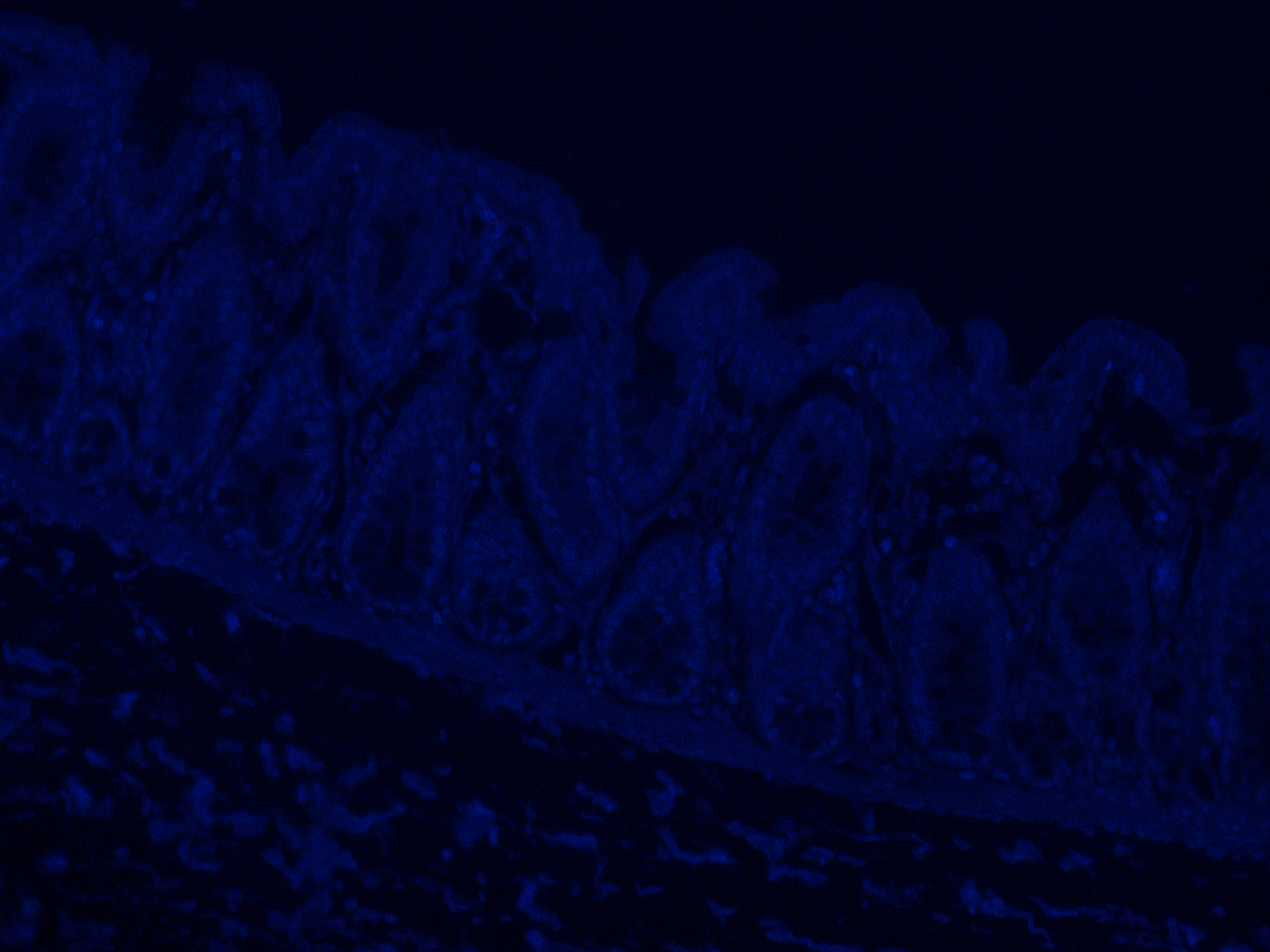

Supplement: Supplementary file 2 [file DataSheet4.ZIP › Supplementary_Material-original data2/FIGURE6/Figures 6J-K(Cecal-IF×200 )/Figure6-K-NLRP3-Caspase-1/SHD-H/CM4-11 NLRP3(绿)+caspase1(红) 200-6.jpg]

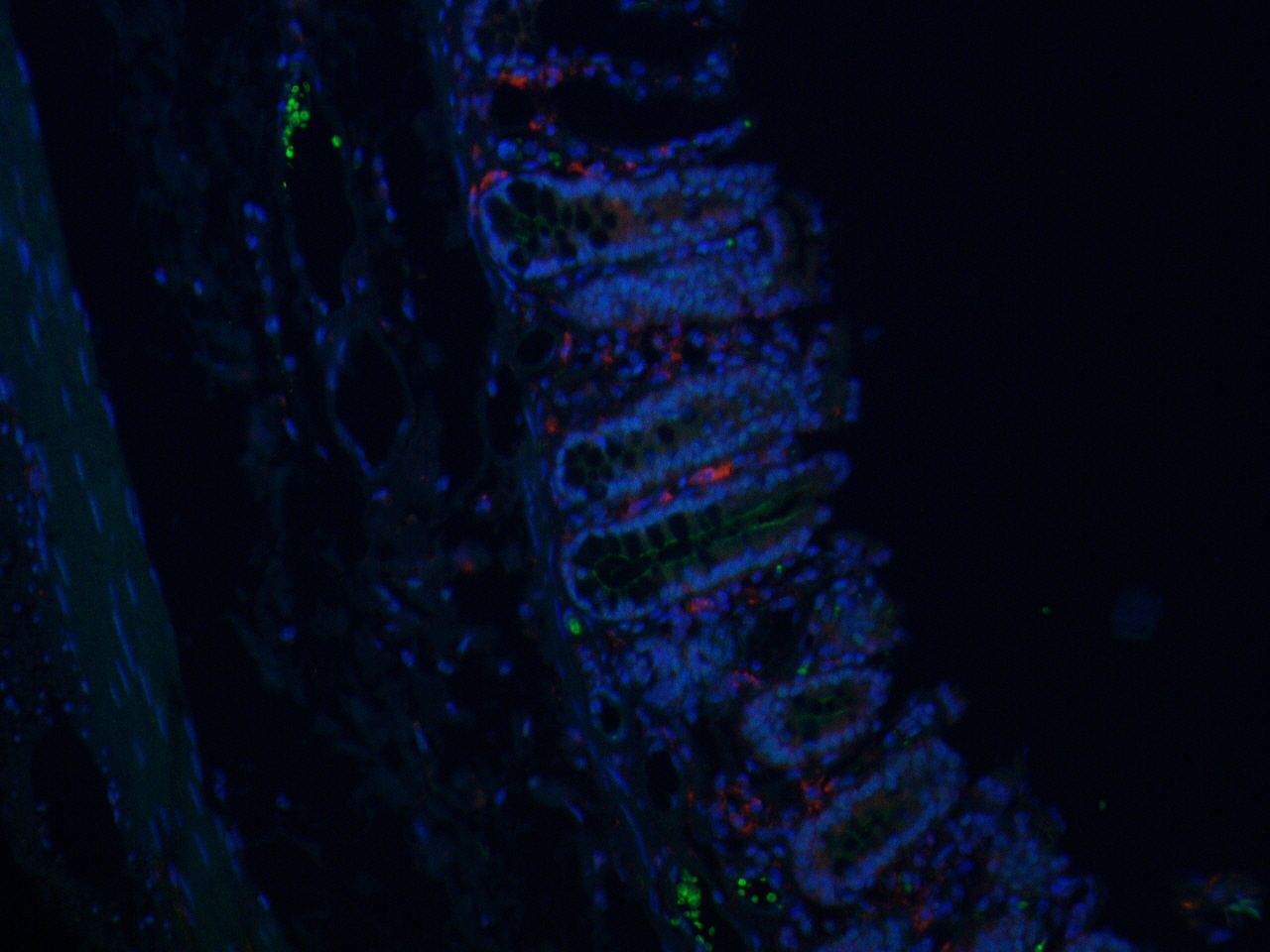

Supplement: Supplementary file 2 [file DataSheet4.ZIP › Supplementary_Material-original data2/FIGURE6/Figures 6J-K(Cecal-IF×200 )/Figure6-K-NLRP3-Caspase-1/SHD-L/CM3-9 NLRP3(绿)+caspase1(红) 200-4 5 6.jpg]

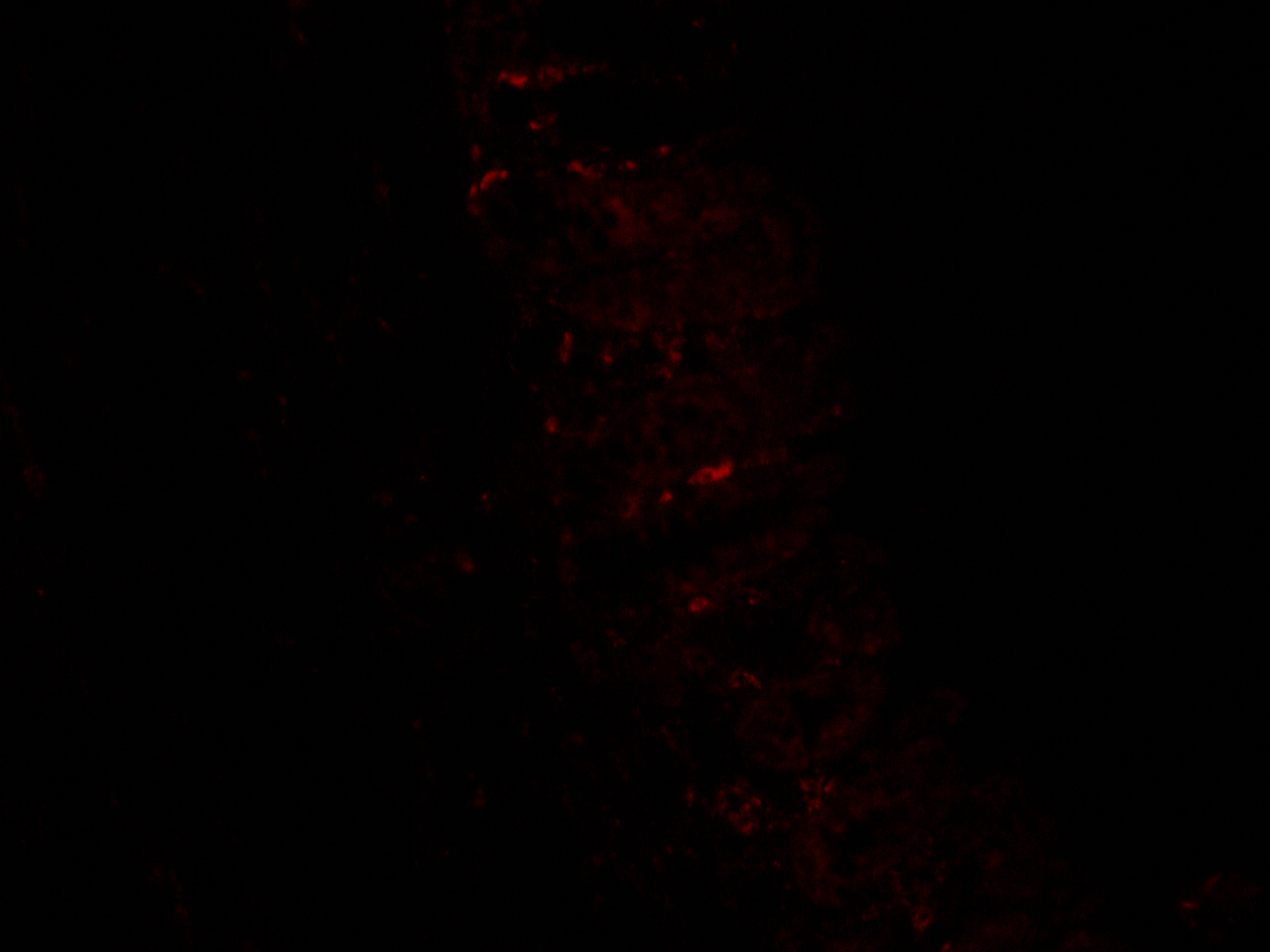

Supplement: Supplementary file 2 [file DataSheet4.ZIP › Supplementary_Material-original data2/FIGURE6/Figures 6J-K(Cecal-IF×200 )/Figure6-K-NLRP3-Caspase-1/SHD-L/CM3-9 NLRP3(绿)+caspase1(红) 200-4.jpg]

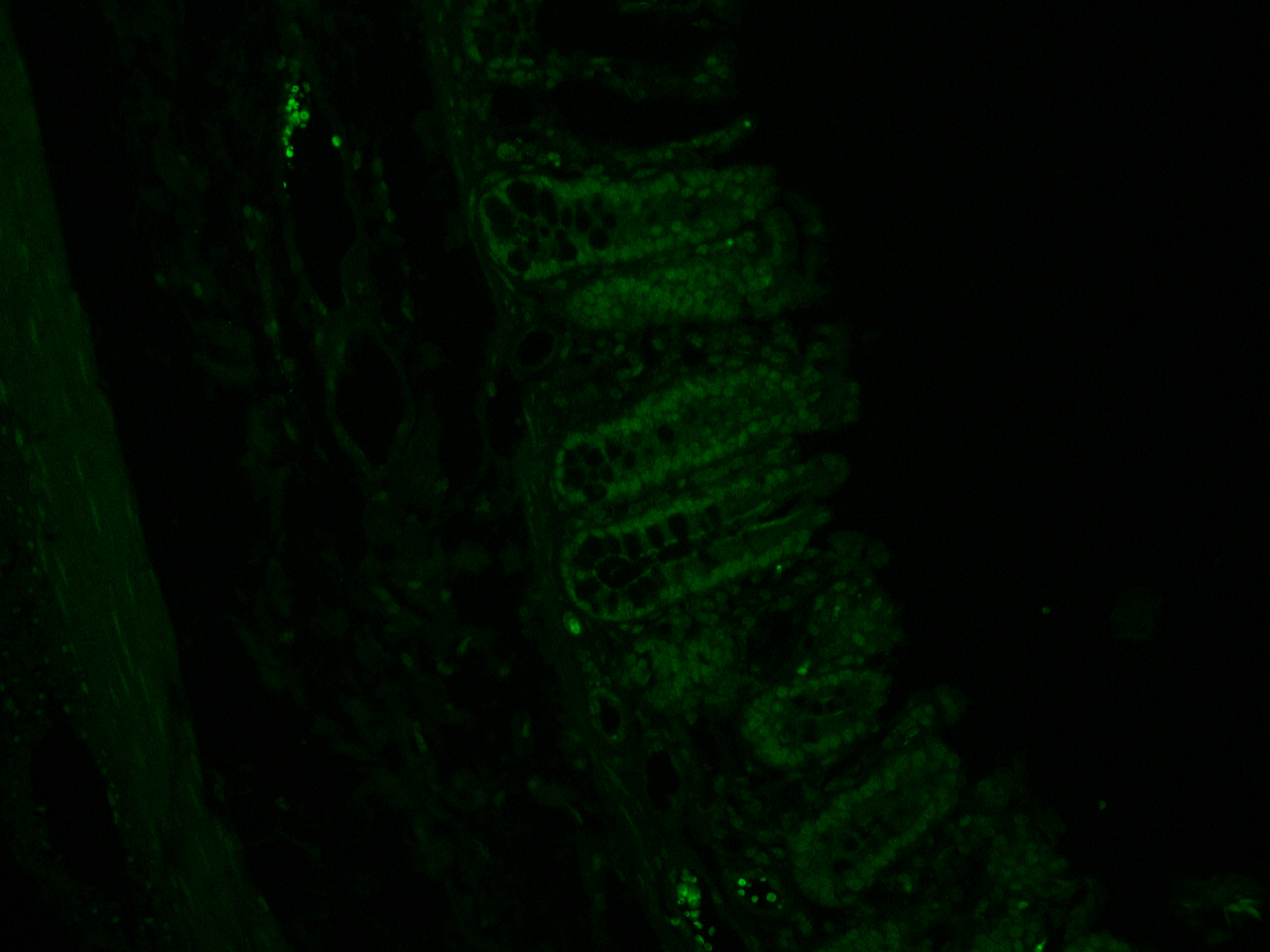

Supplement: Supplementary file 2 [file DataSheet4.ZIP › Supplementary_Material-original data2/FIGURE6/Figures 6J-K(Cecal-IF×200 )/Figure6-K-NLRP3-Caspase-1/SHD-L/CM3-9 NLRP3(绿)+caspase1(红) 200-5.jpg]

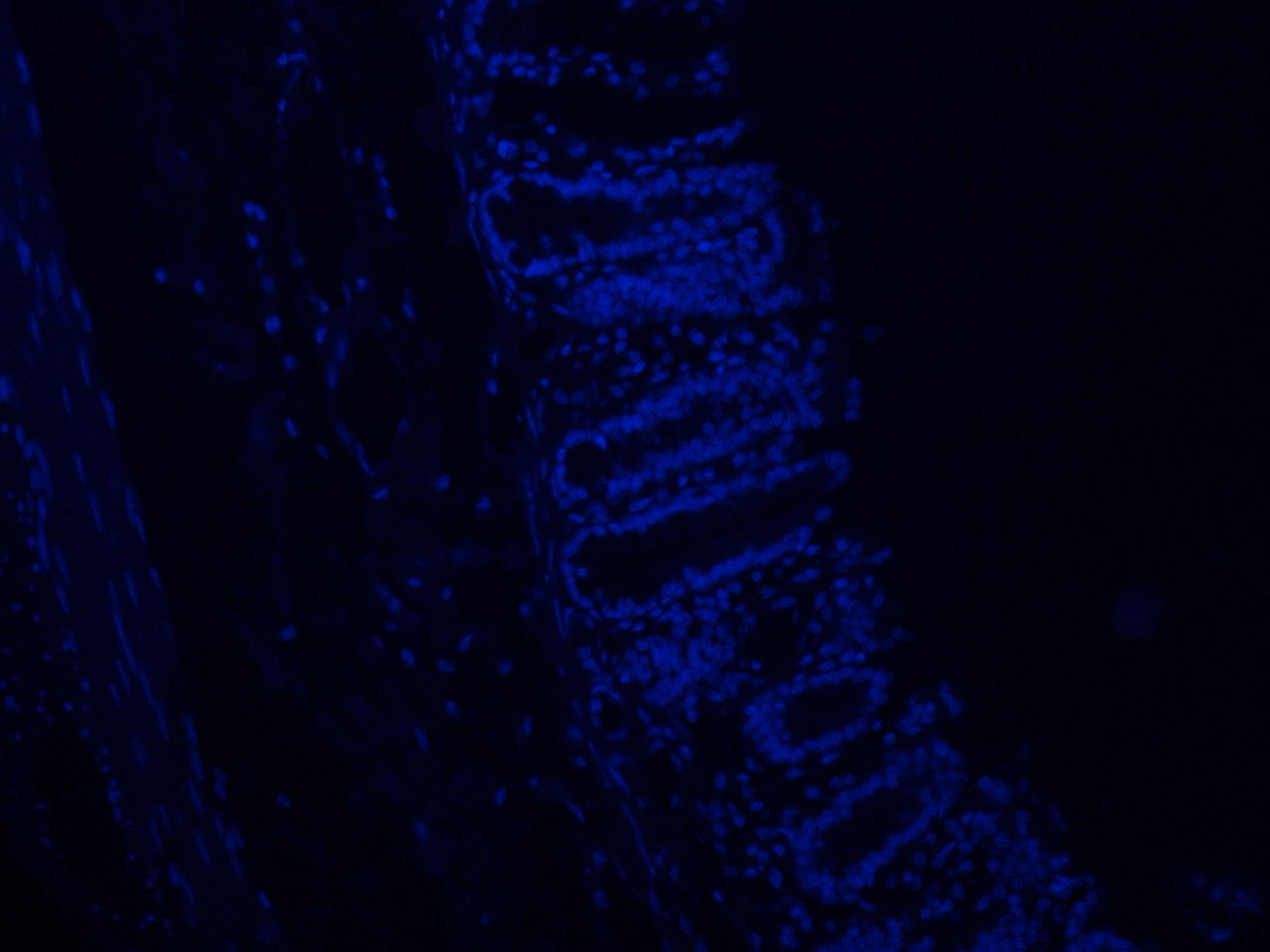

Supplement: Supplementary file 2 [file DataSheet4.ZIP › Supplementary_Material-original data2/FIGURE6/Figures 6J-K(Cecal-IF×200 )/Figure6-K-NLRP3-Caspase-1/SHD-L/CM3-9 NLRP3(绿)+caspase1(红) 200-6.jpg]

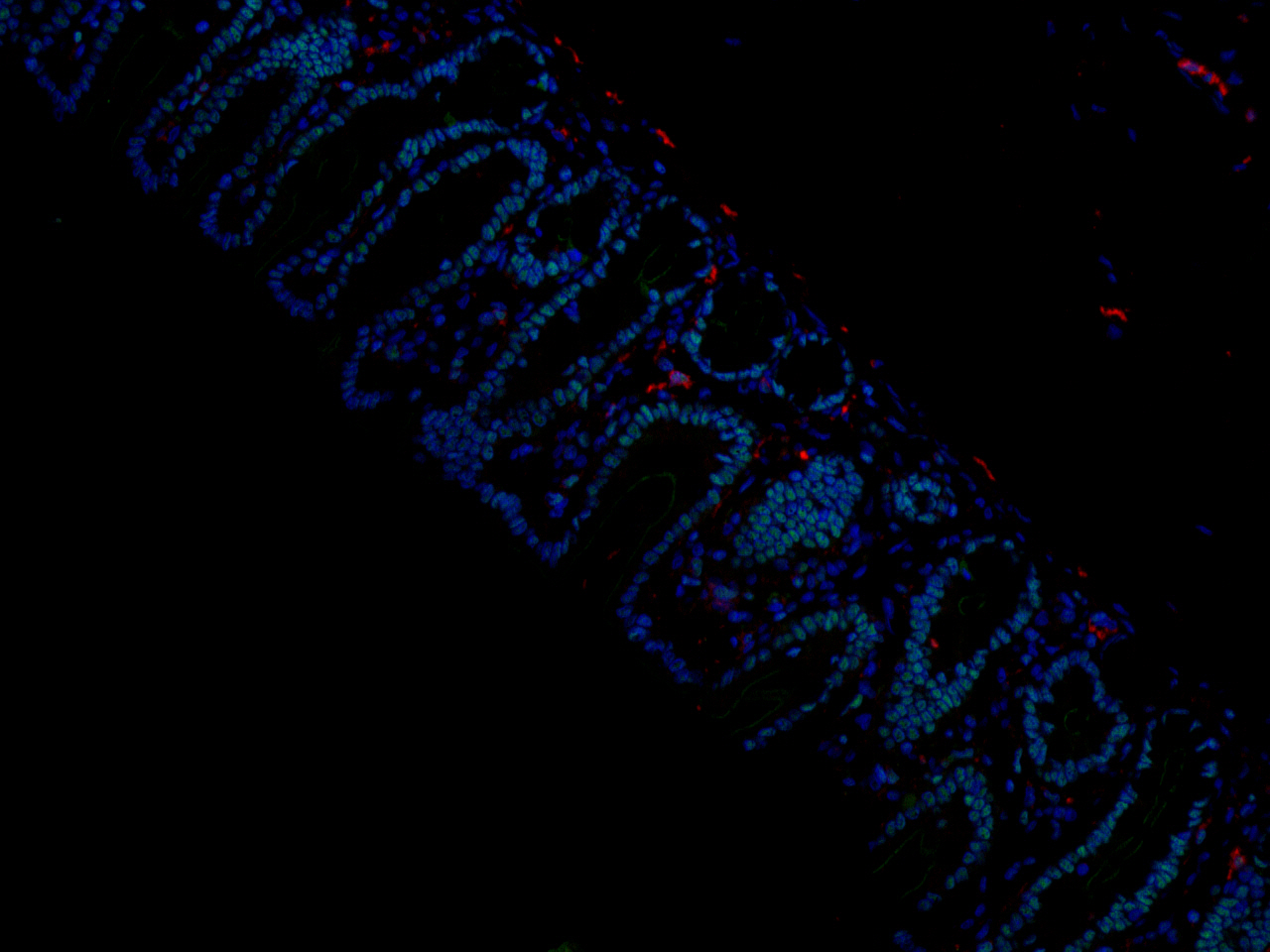

Supplement: Supplementary file 2 [file DataSheet4.ZIP › Supplementary_Material-original data2/FIGURE6/Figures 6J-K(Cecal-IF×200 )/Figure6-K-NLRP3-Caspase-1/SNS/盲 Y1-3 NLRP3(绿)+ASC(红) 200-7 8 9.jpg]

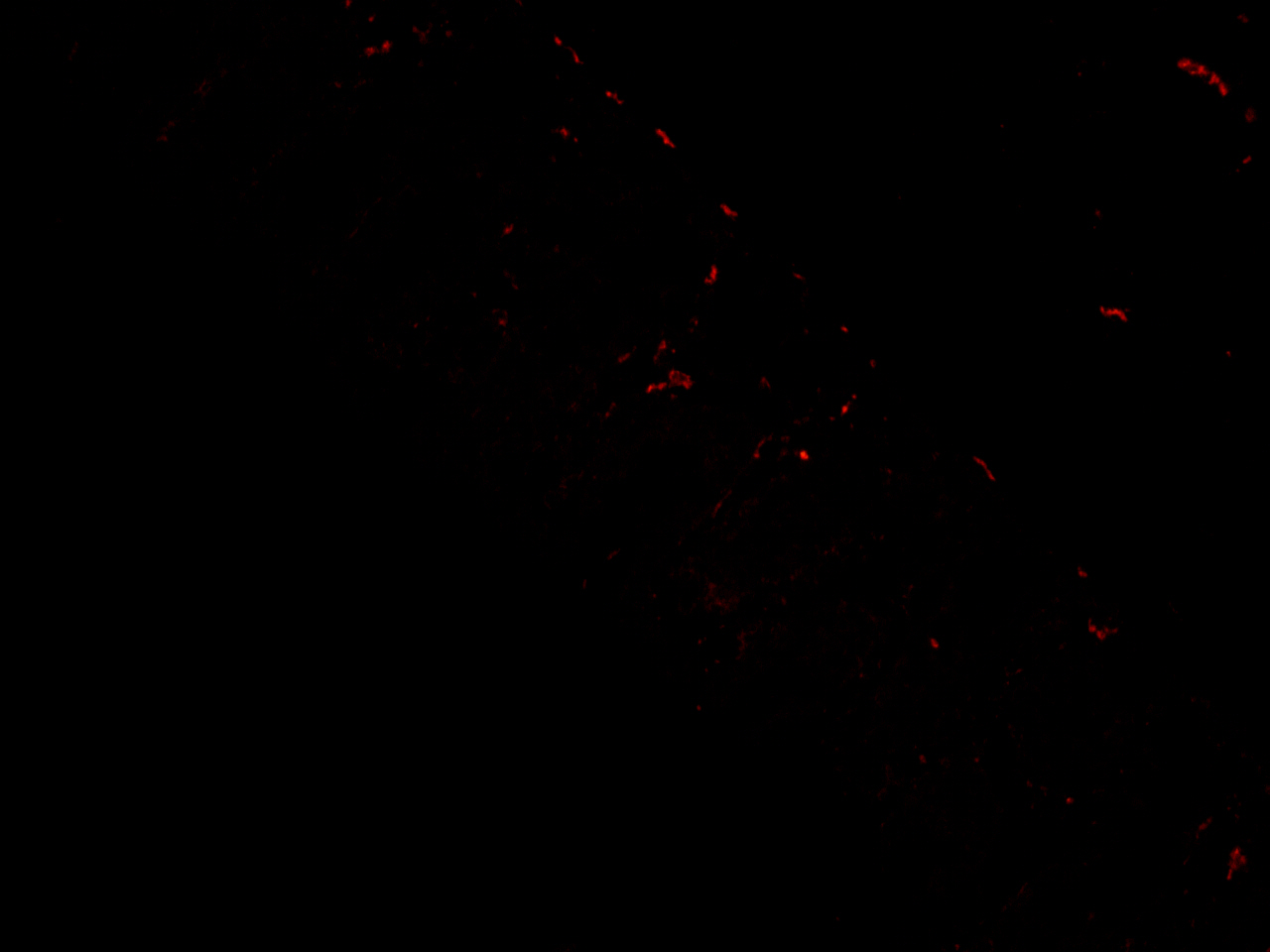

Supplement: Supplementary file 2 [file DataSheet4.ZIP › Supplementary_Material-original data2/FIGURE6/Figures 6J-K(Cecal-IF×200 )/Figure6-K-NLRP3-Caspase-1/SNS/盲 Y1-3 NLRP3(绿)+ASC(红) 200-7.jpg]

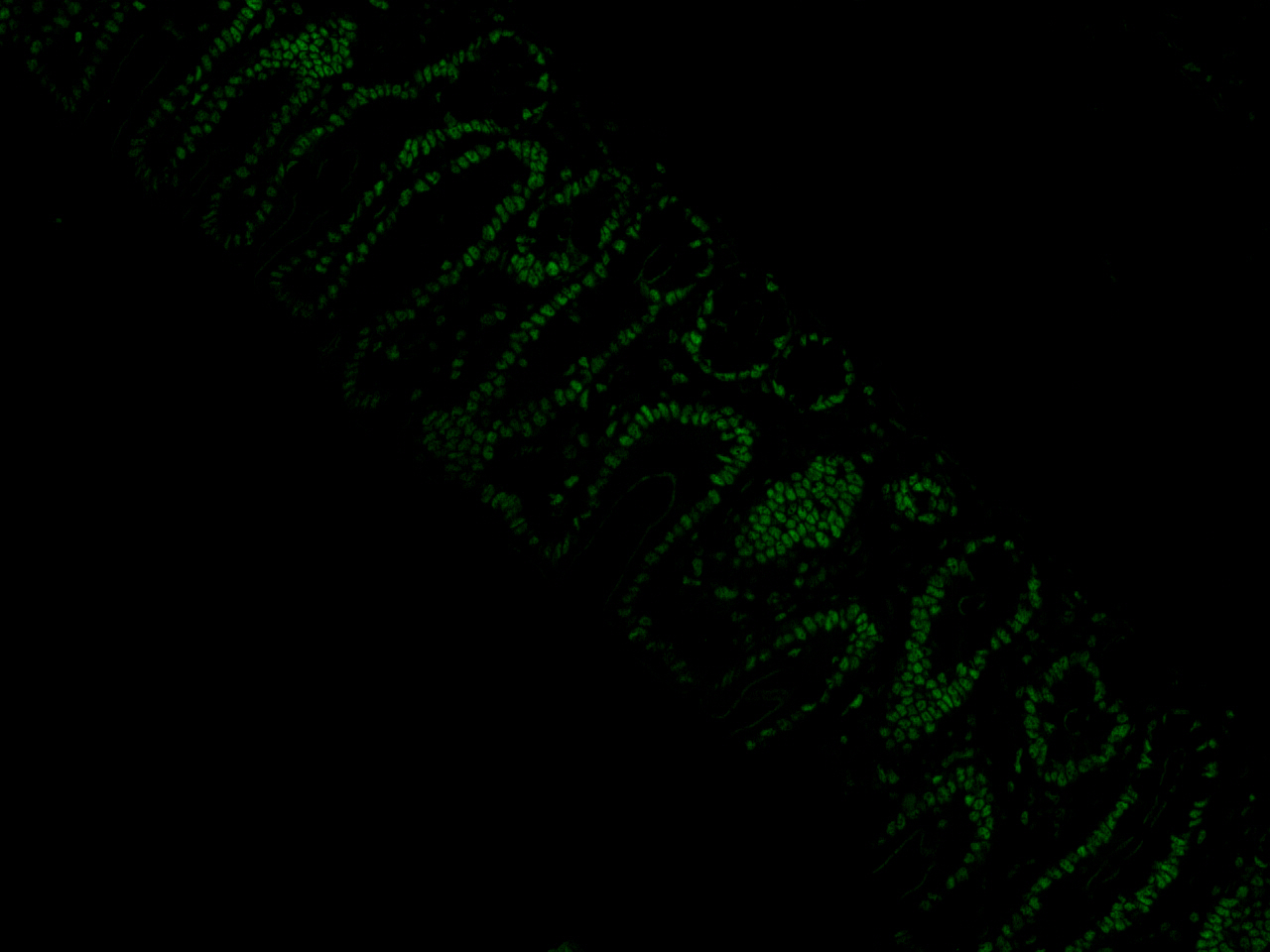

Supplement: Supplementary file 2 [file DataSheet4.ZIP › Supplementary_Material-original data2/FIGURE6/Figures 6J-K(Cecal-IF×200 )/Figure6-K-NLRP3-Caspase-1/SNS/盲 Y1-3 NLRP3(绿)+ASC(红) 200-8.jpg]

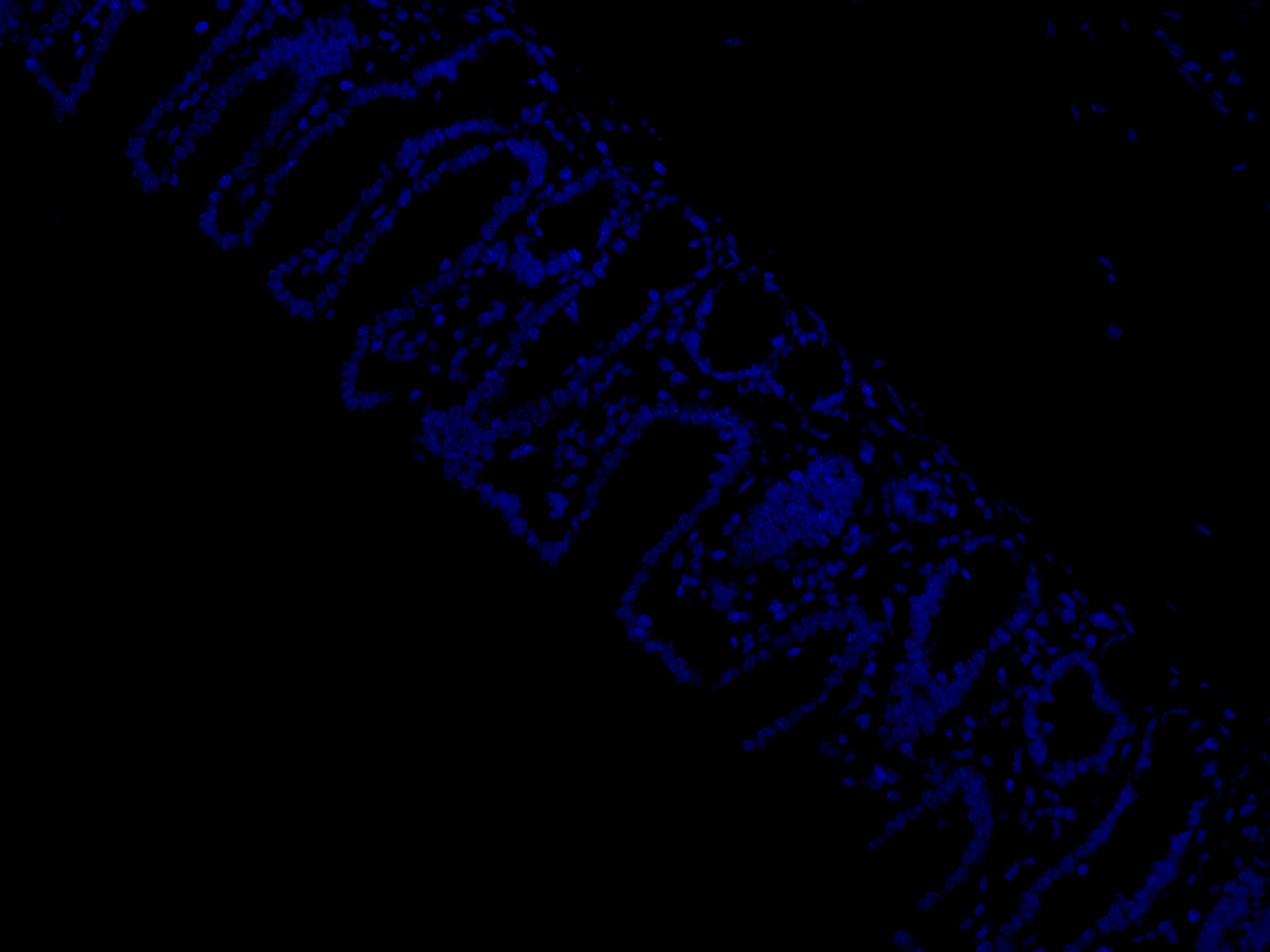

Supplement: Supplementary file 2 [file DataSheet4.ZIP › Supplementary_Material-original data2/FIGURE6/Figures 6J-K(Cecal-IF×200 )/Figure6-K-NLRP3-Caspase-1/SNS/盲 Y1-3 NLRP3(绿)+ASC(红) 200-9.jpg]

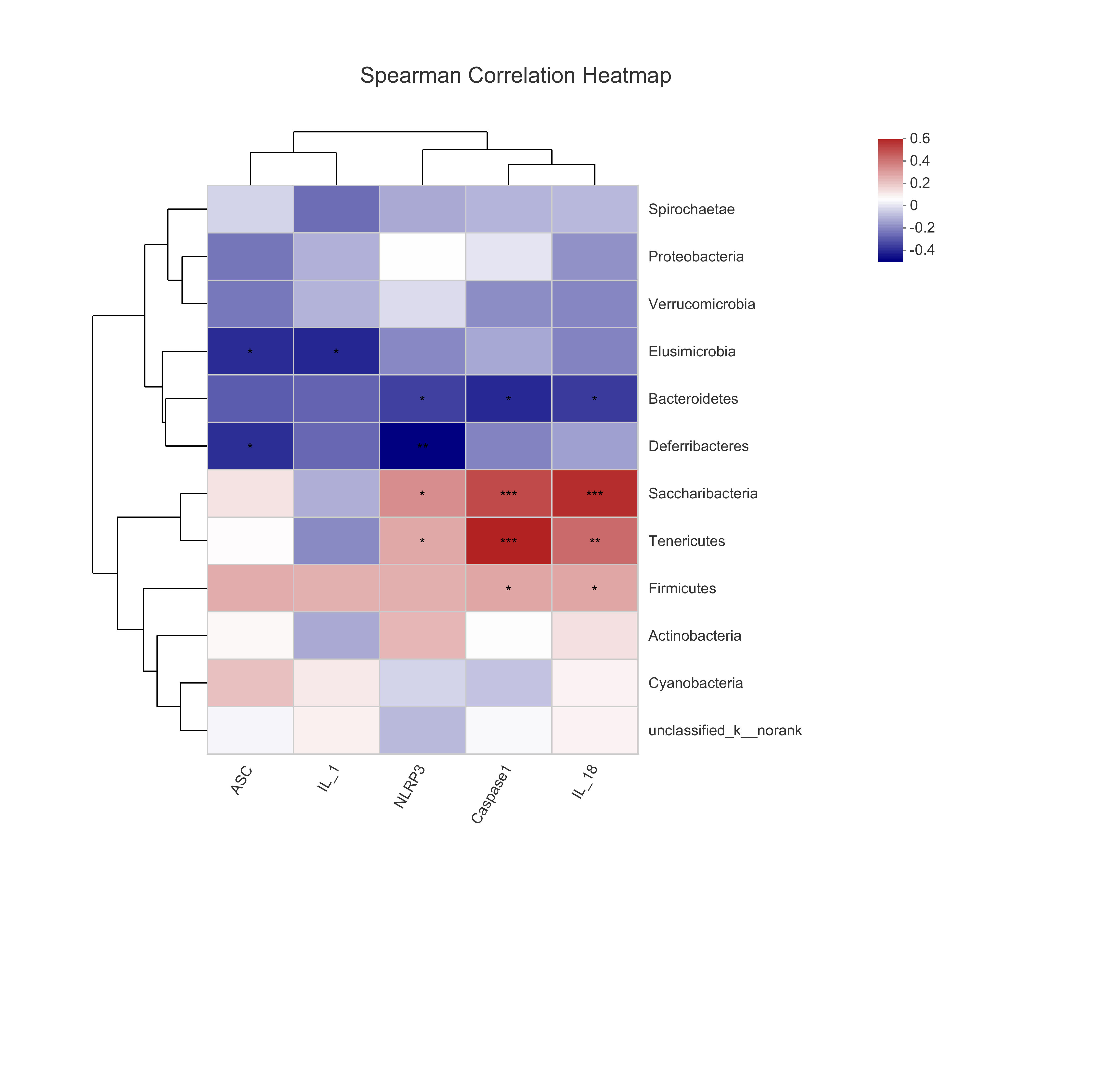

Supplement: Supplementary file 2 [file DataSheet4.ZIP › Supplementary_Material-original data2/FIGURE7/Figure 7A/Figure 6A.png]

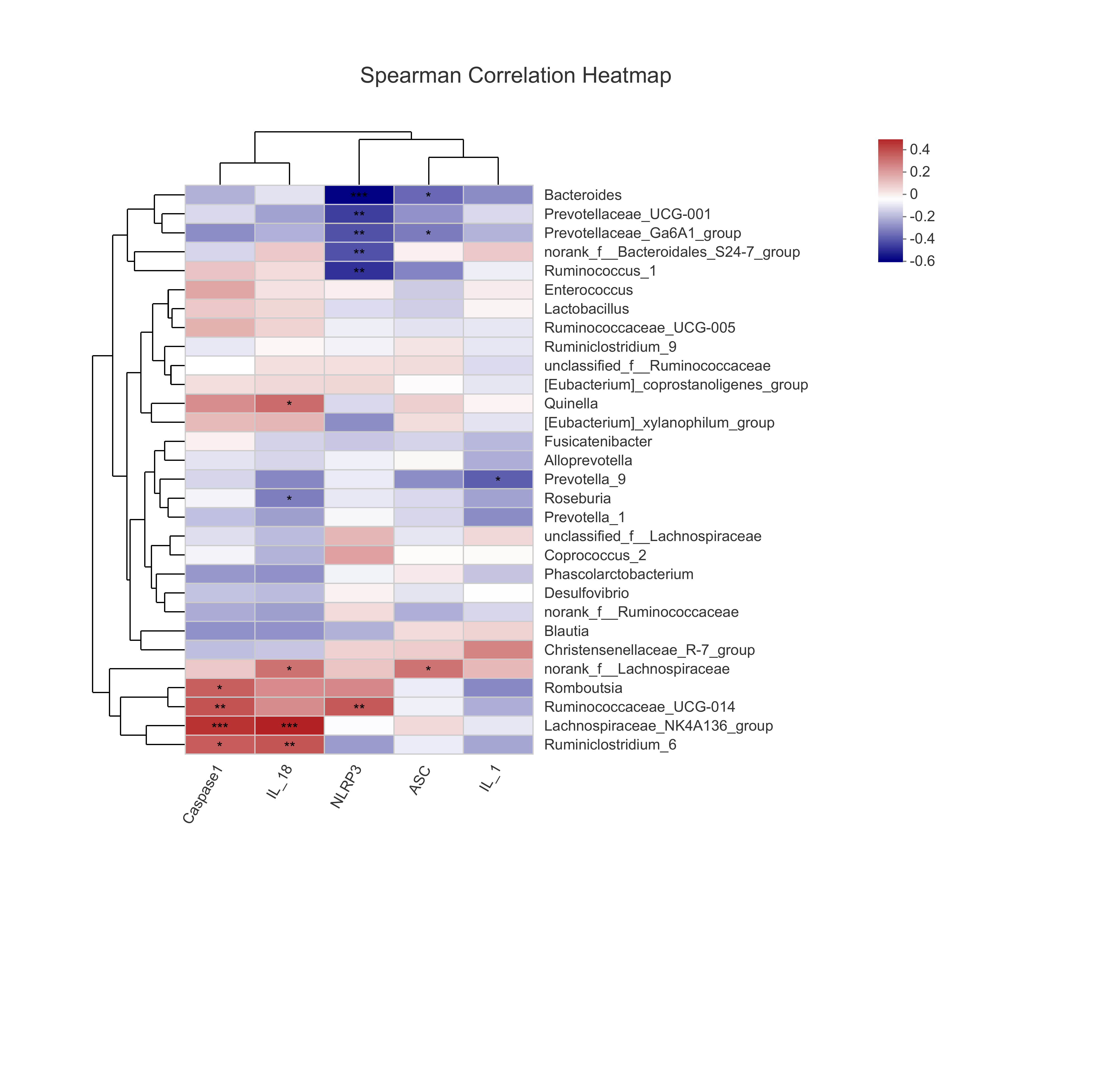

Supplement: Supplementary file 2 [file DataSheet4.ZIP › Supplementary_Material-original data2/FIGURE7/Figure 7B/Figure 6B.png]

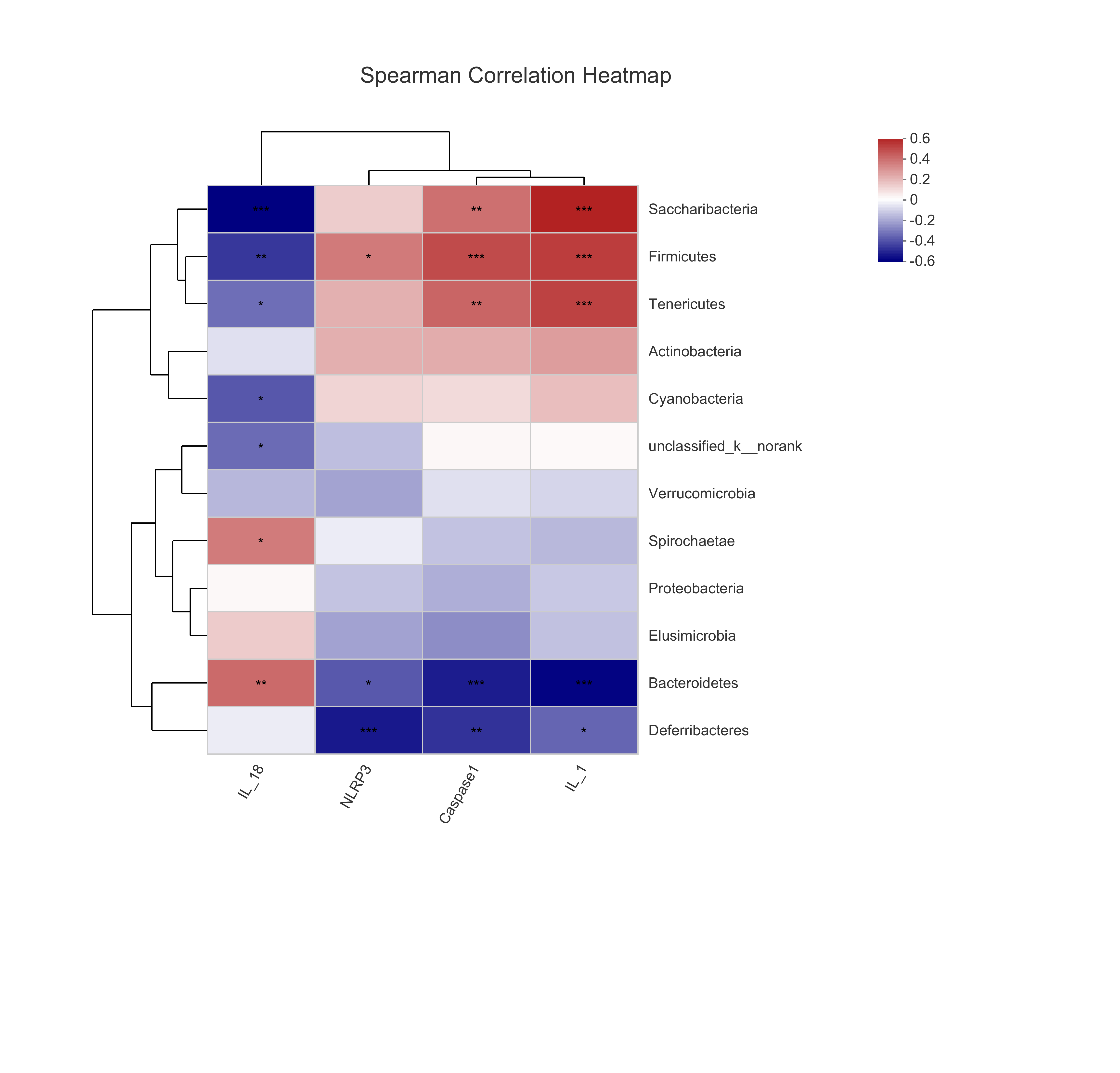

Supplement: Supplementary file 2 [file DataSheet4.ZIP › Supplementary_Material-original data2/FIGURE7/Figure 7C/Figure 6C.png]

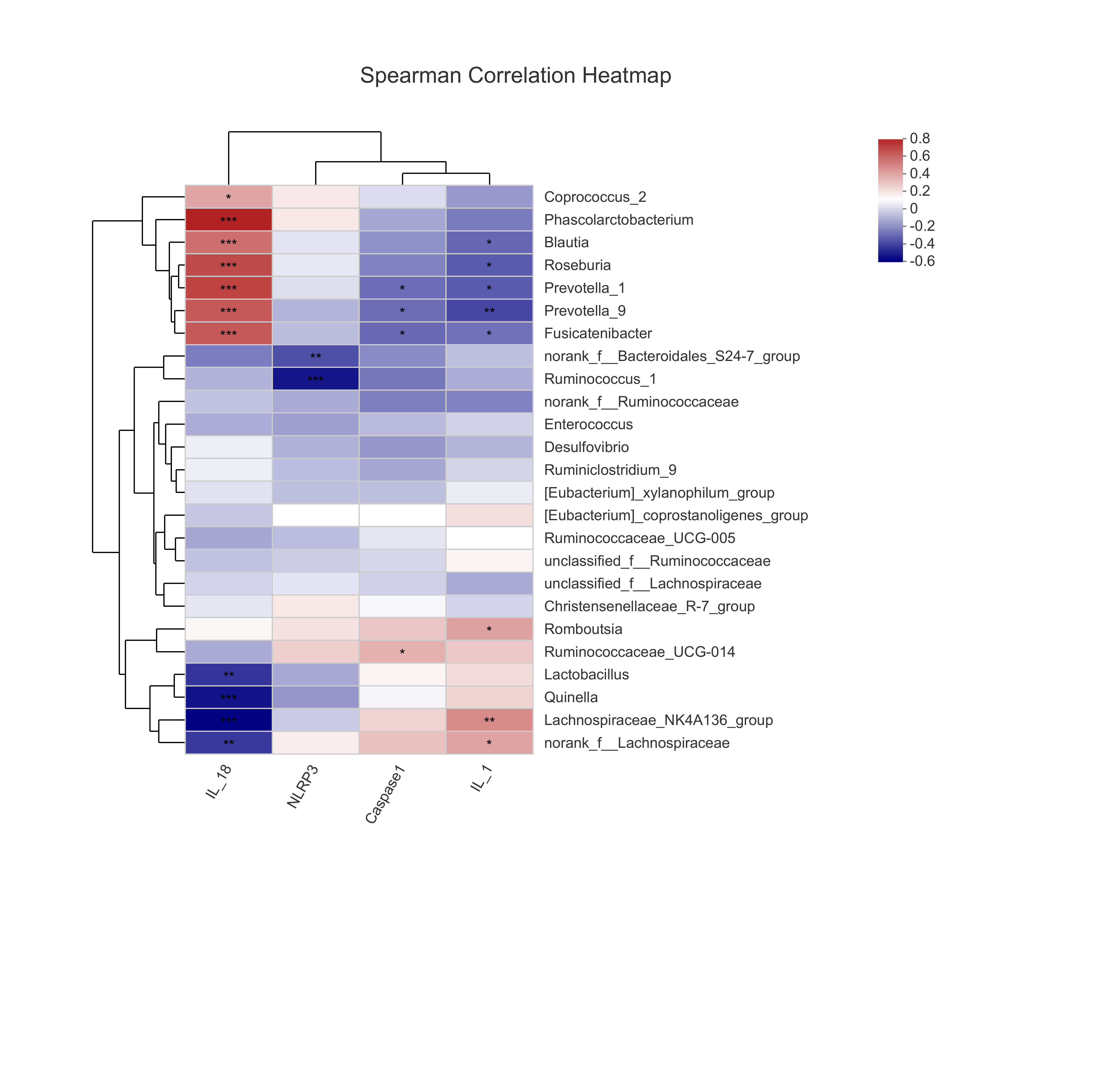

Supplement: Supplementary file 2 [file DataSheet4.ZIP › Supplementary_Material-original data2/FIGURE7/Figure 7D/Figure 6D.png]

Rank-Abundance curves

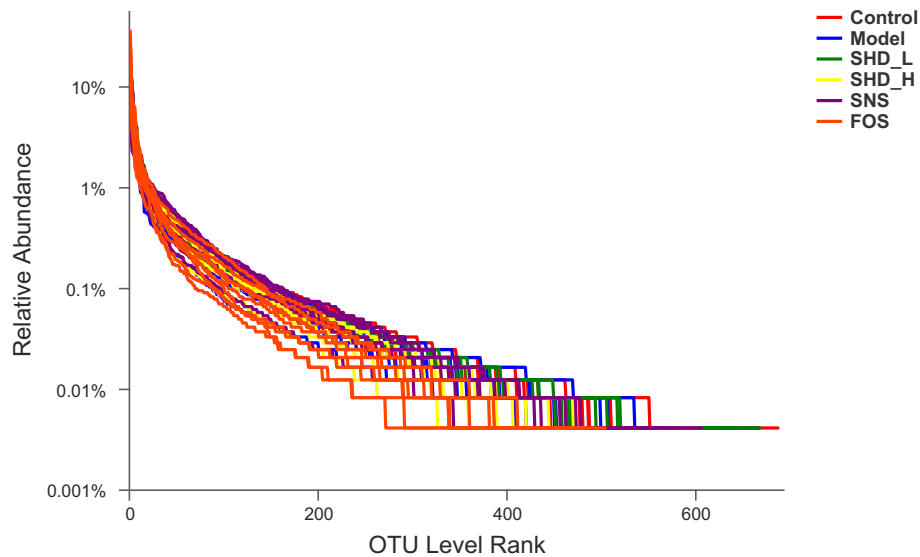

Supplement: Supplementary file 3 [file DataSheet2.ZIP › Supplementary Figures/Supplementary Figure 1-Rank-Abundance.pdf]

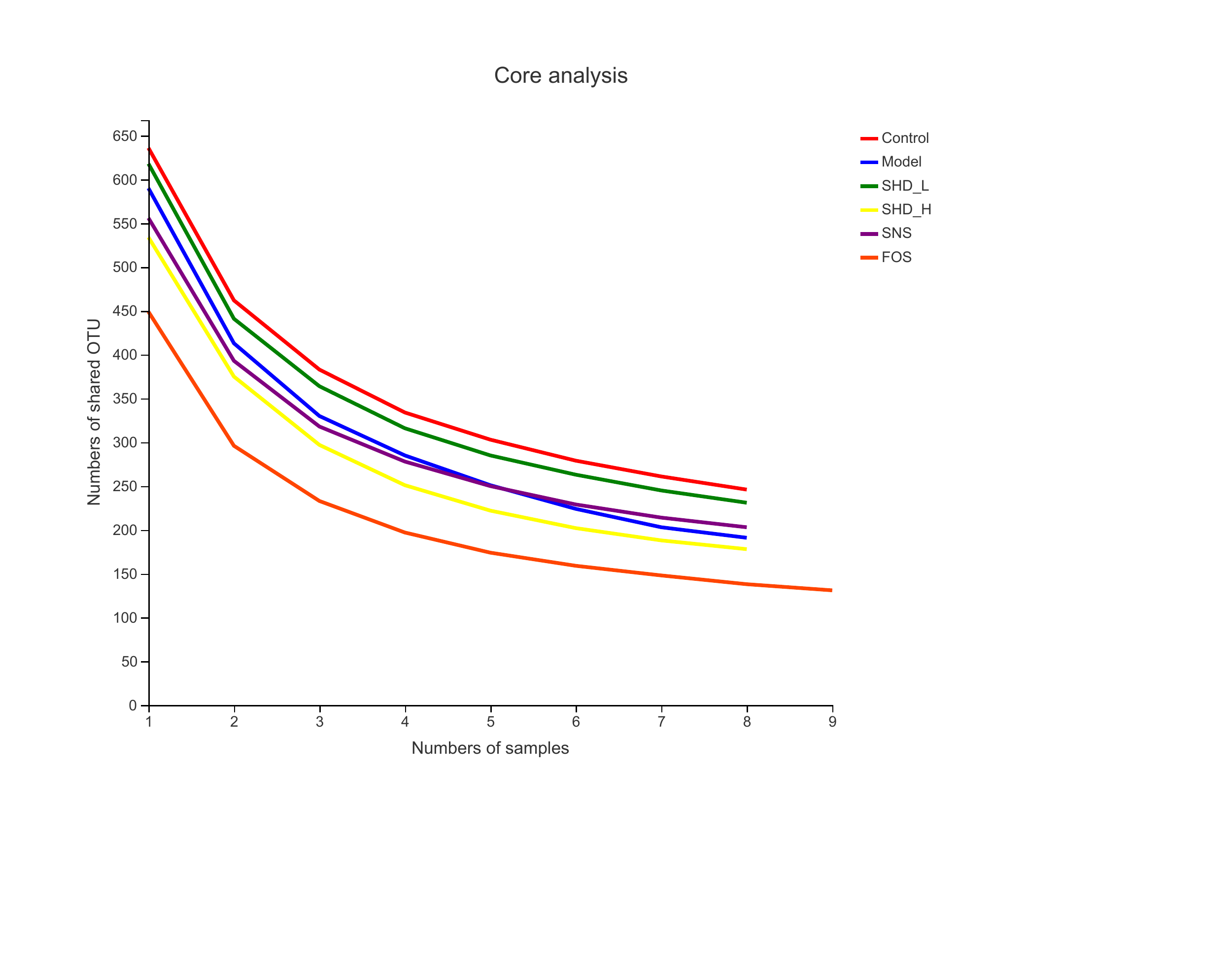

Supplement: Supplementary file 3 [file DataSheet2.ZIP › Supplementary Figures/Supplementary Figure 2-core.png]

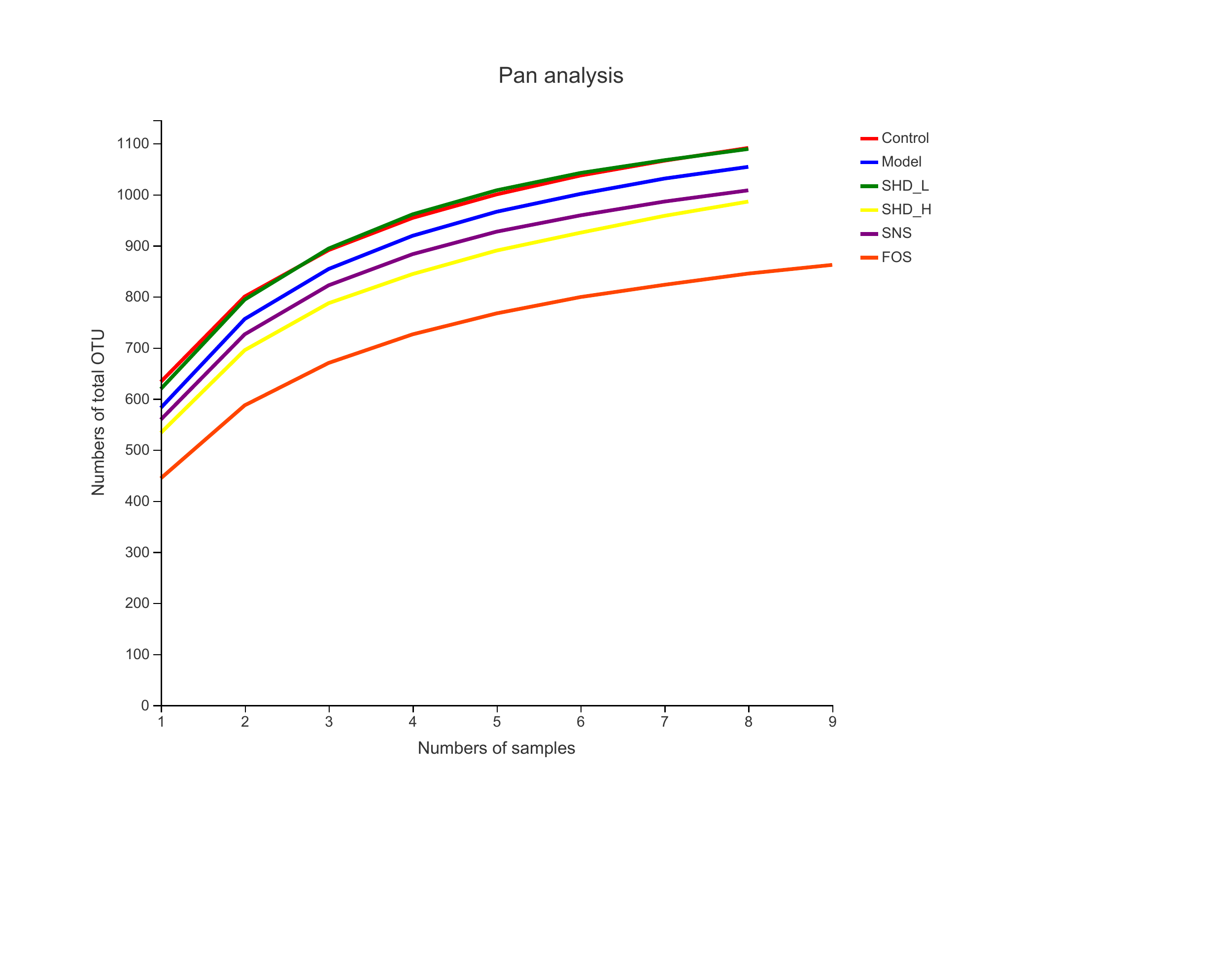

Supplement: Supplementary file 3 [file DataSheet2.ZIP › Supplementary Figures/Supplementary Figure 3-pan.png]

COG function classification

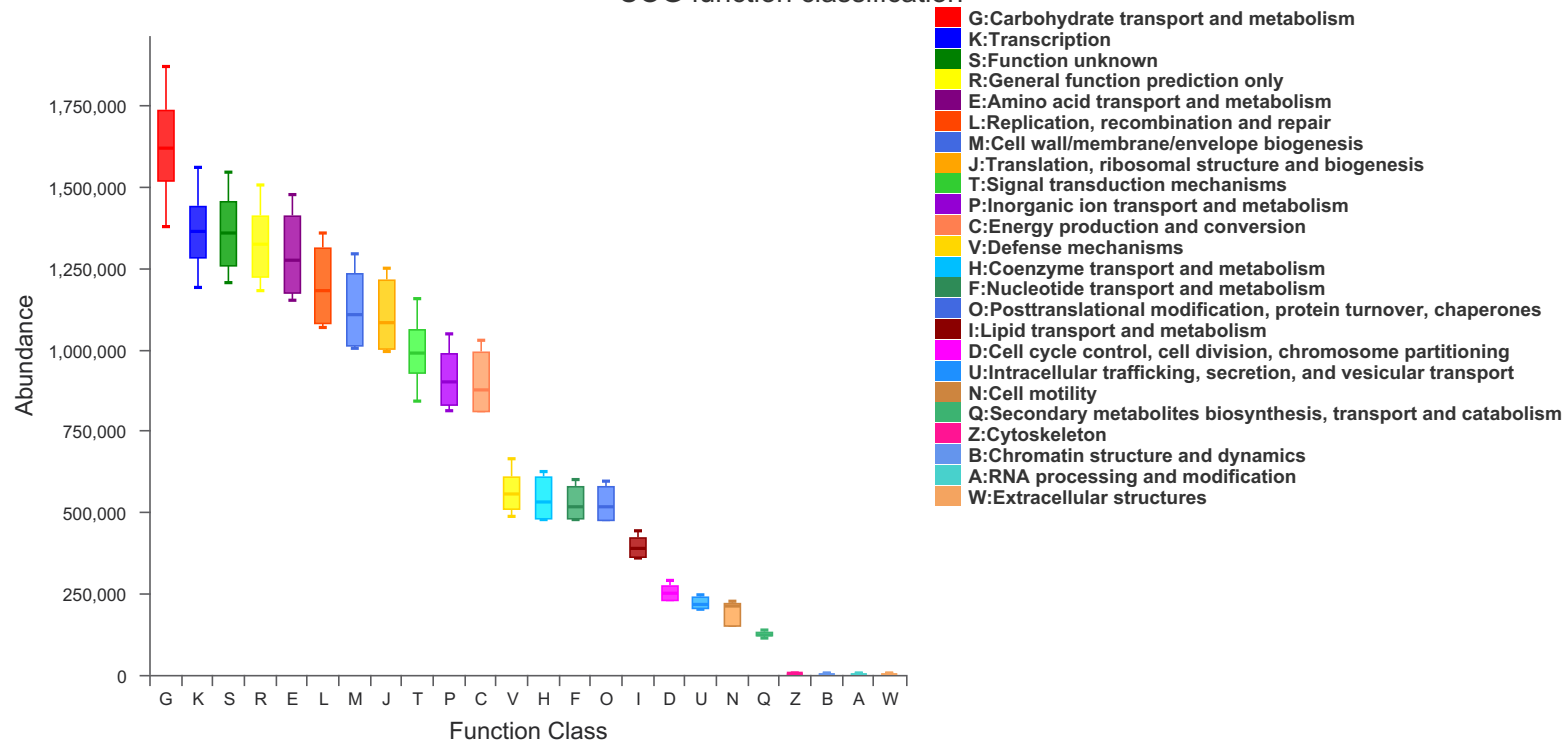

Supplement: Supplementary file 3 [file DataSheet2.ZIP › Supplementary Figures/Supplementary Figure 4-COG function classification-box.pdf]

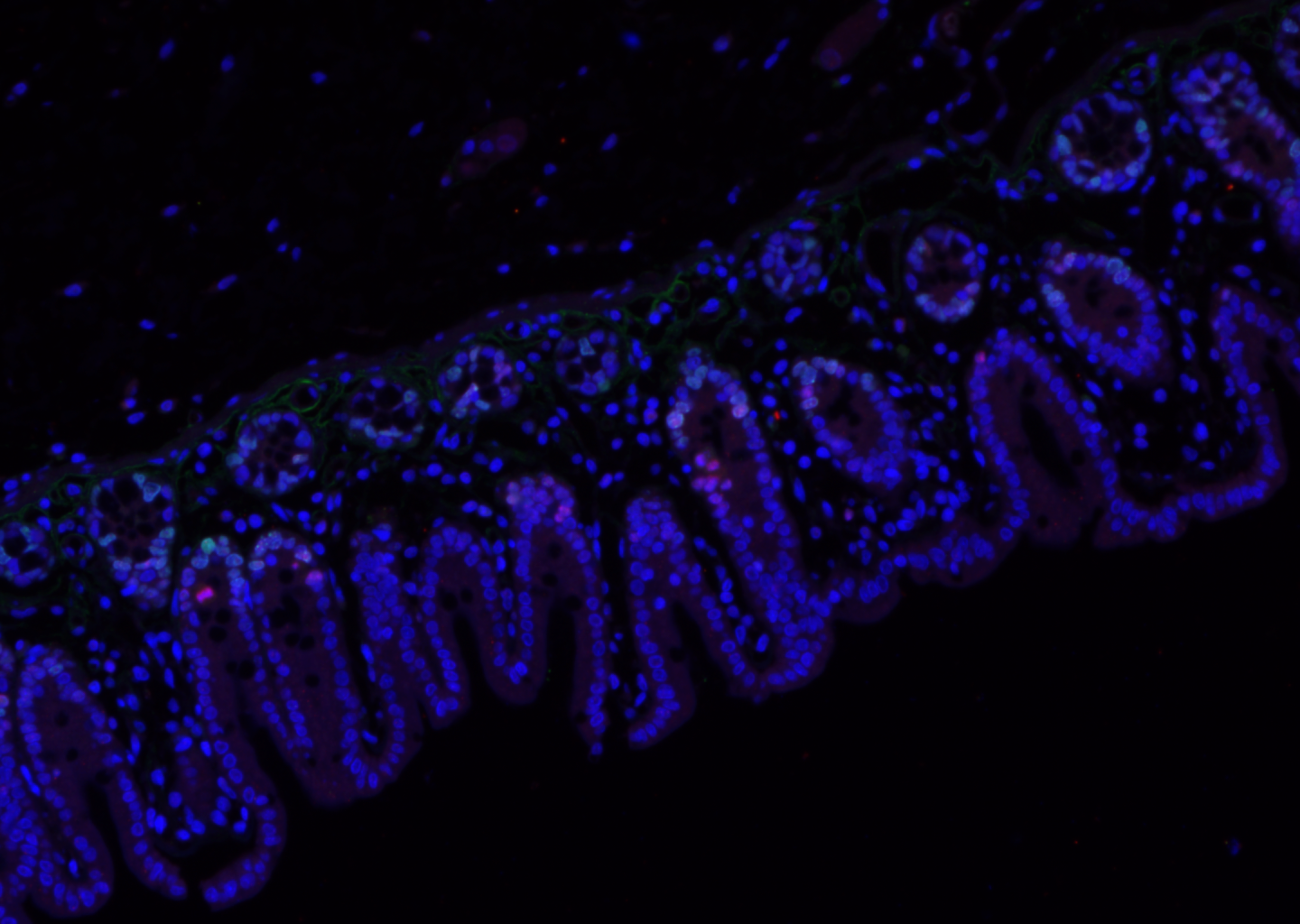

Supplement: Supplementary file 4 [file DataSheet5.ZIP › Supplementary_Material-original data3/FIGURE6/Figures 6J-K(Cecal-IF×200 )/Figure6-J-NLRP3-ASC/Control/CM1-13 NLRP3(绿光)+ASC(红光) 200-4 5 6.tif]

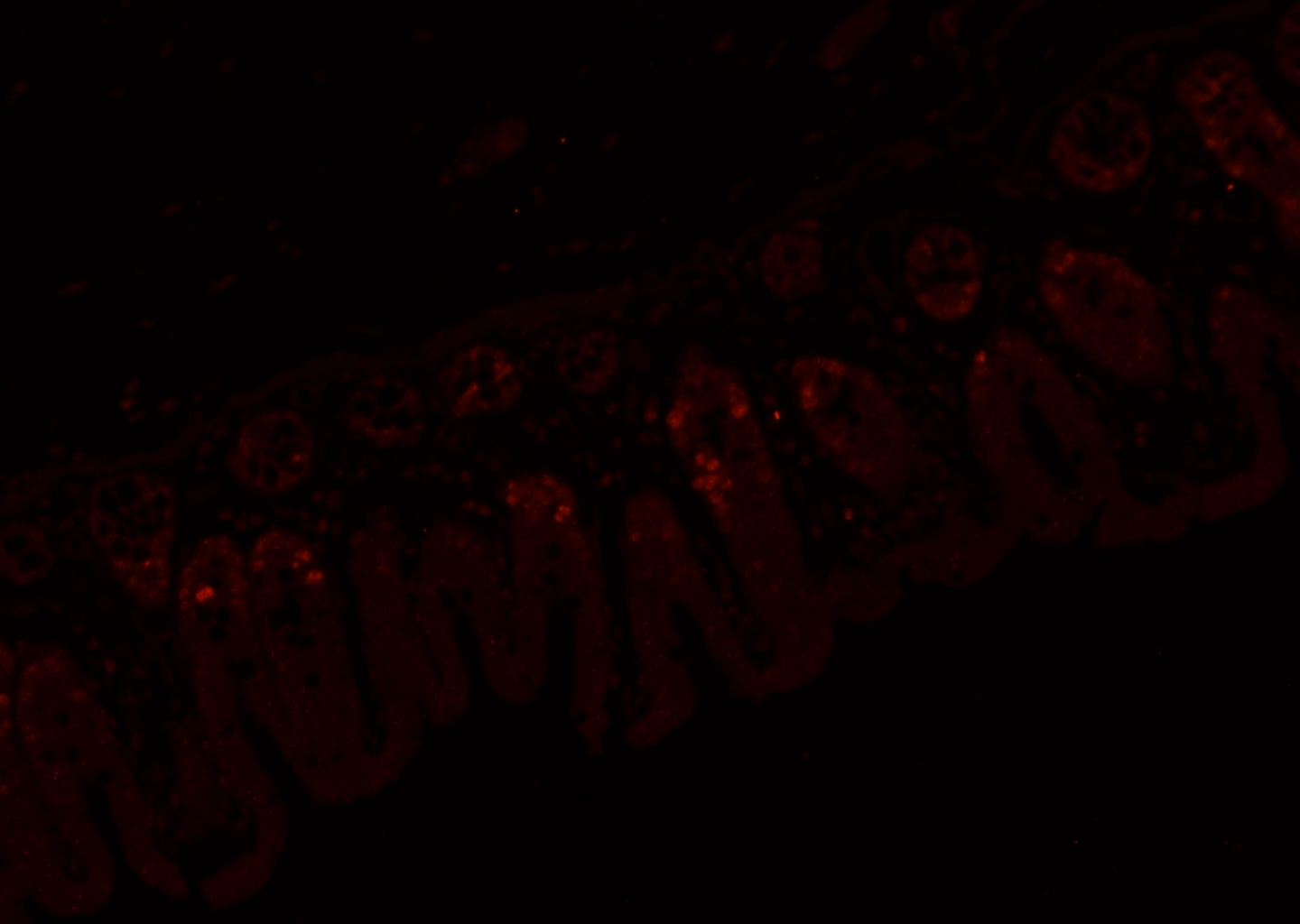

Supplement: Supplementary file 4 [file DataSheet5.ZIP › Supplementary_Material-original data3/FIGURE6/Figures 6J-K(Cecal-IF×200 )/Figure6-J-NLRP3-ASC/Control/CM1-13 NLRP3(绿光)+ASC(红光) 200-4.tif]

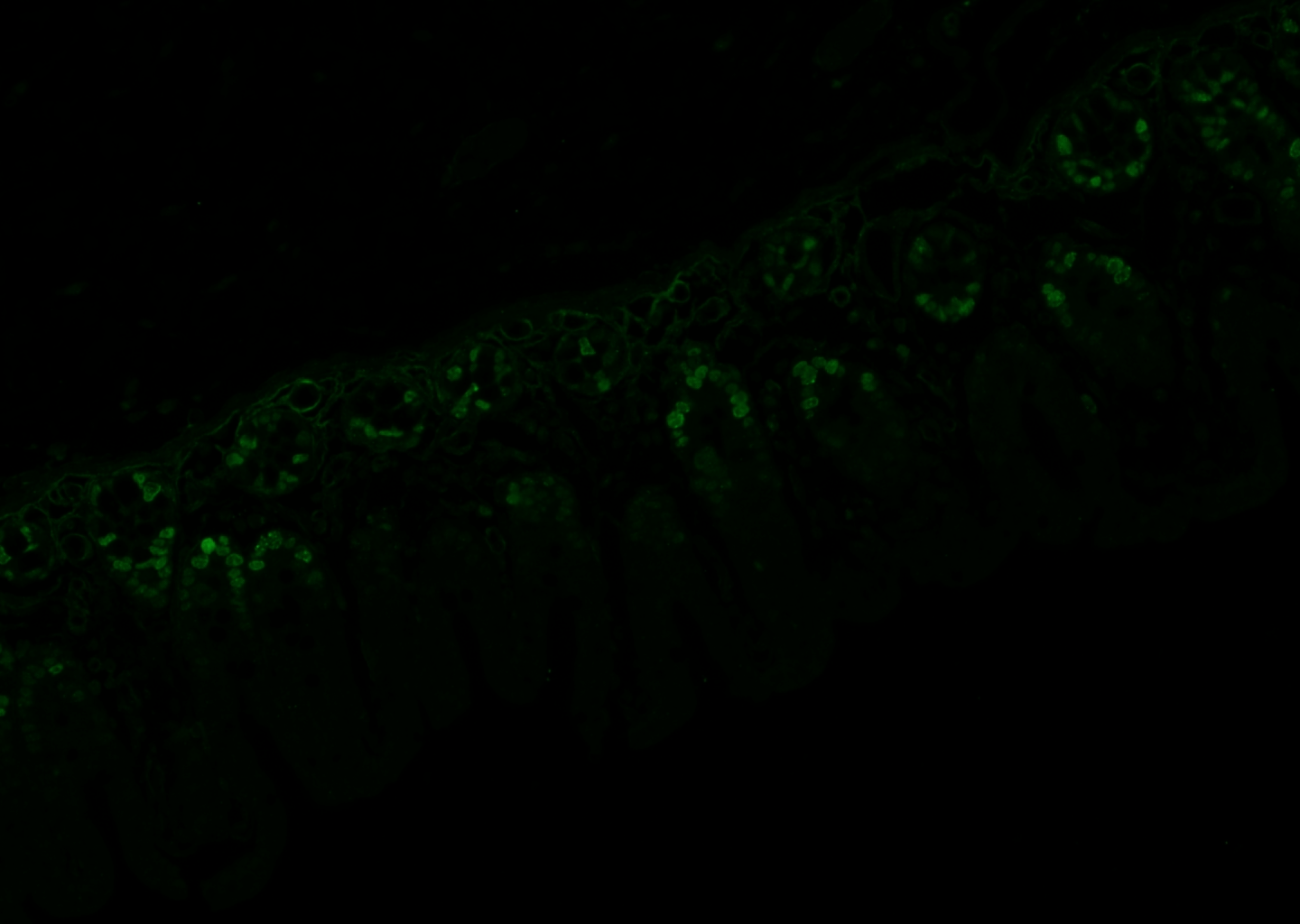

Supplement: Supplementary file 4 [file DataSheet5.ZIP › Supplementary_Material-original data3/FIGURE6/Figures 6J-K(Cecal-IF×200 )/Figure6-J-NLRP3-ASC/Control/CM1-13 NLRP3(绿光)+ASC(红光) 200-5.tif]

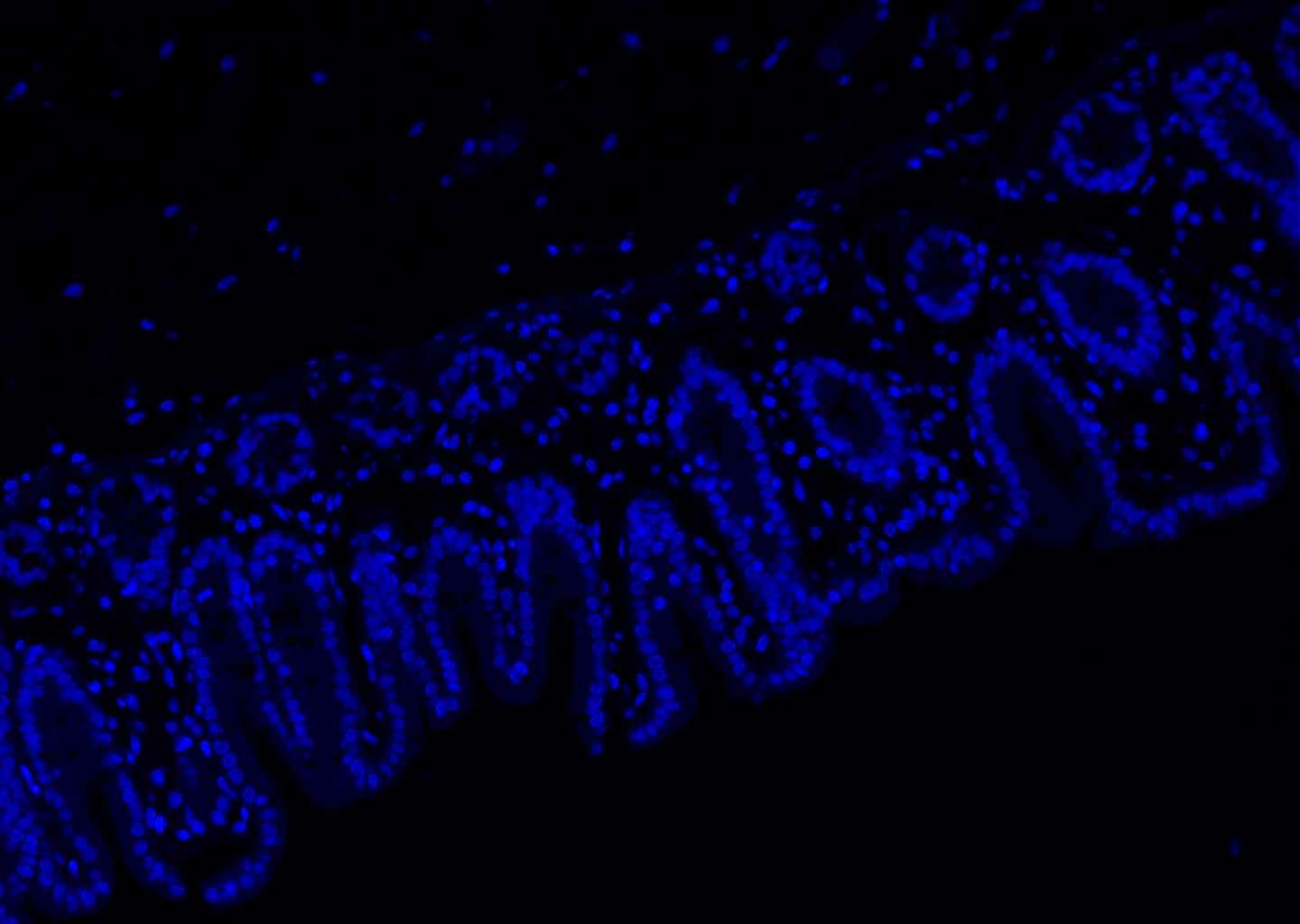

Supplement: Supplementary file 4 [file DataSheet5.ZIP › Supplementary_Material-original data3/FIGURE6/Figures 6J-K(Cecal-IF×200 )/Figure6-J-NLRP3-ASC/Control/CM1-13 NLRP3(绿光)+ASC(红光) 200-6.tif]

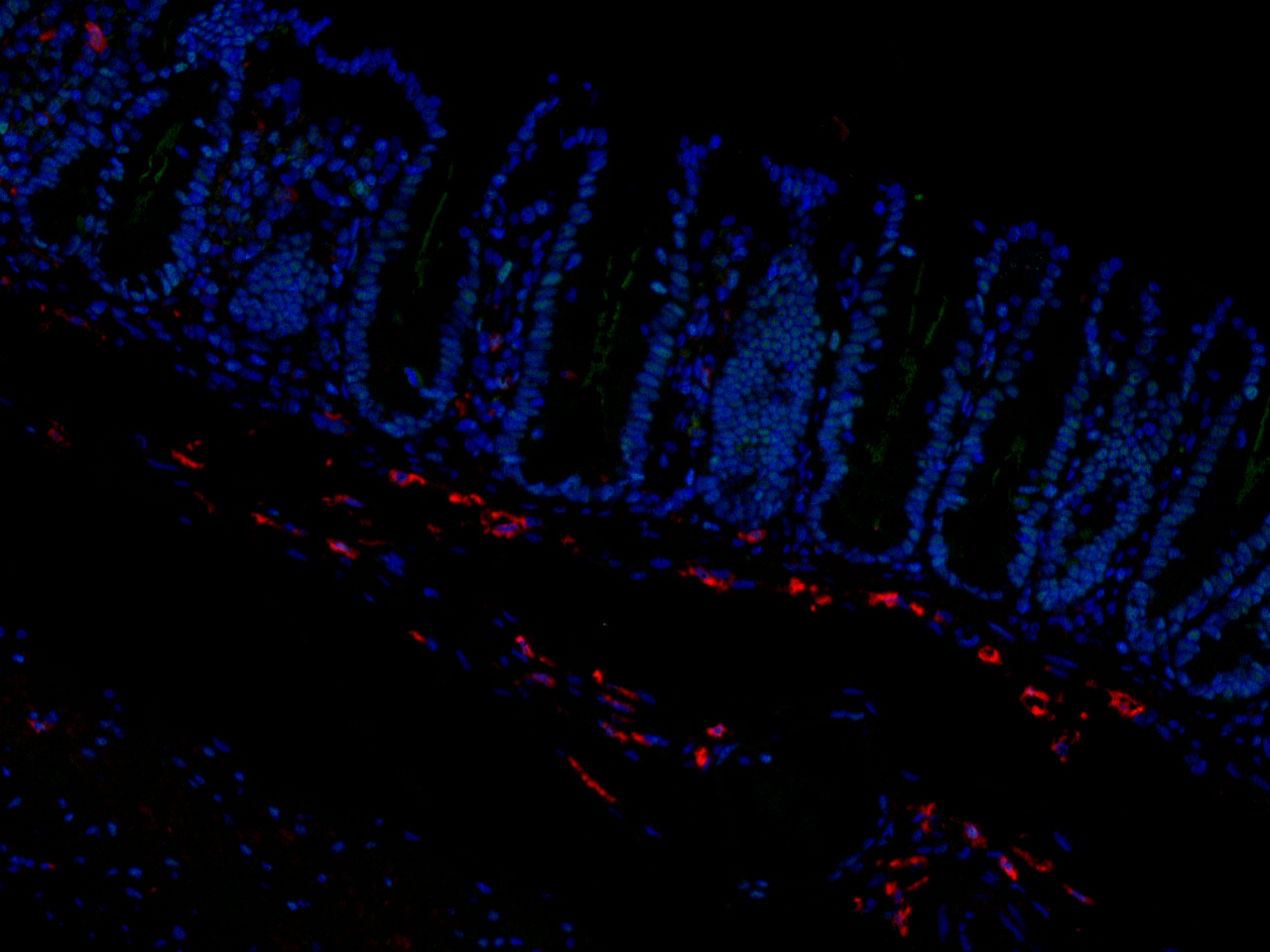

Supplement: Supplementary file 4 [file DataSheet5.ZIP › Supplementary_Material-original data3/FIGURE6/Figures 6J-K(Cecal-IF×200 )/Figure6-J-NLRP3-ASC/FOS/盲 Y2-12 NLRP3(绿)+ASC(红) 200-7 8 9.jpg]

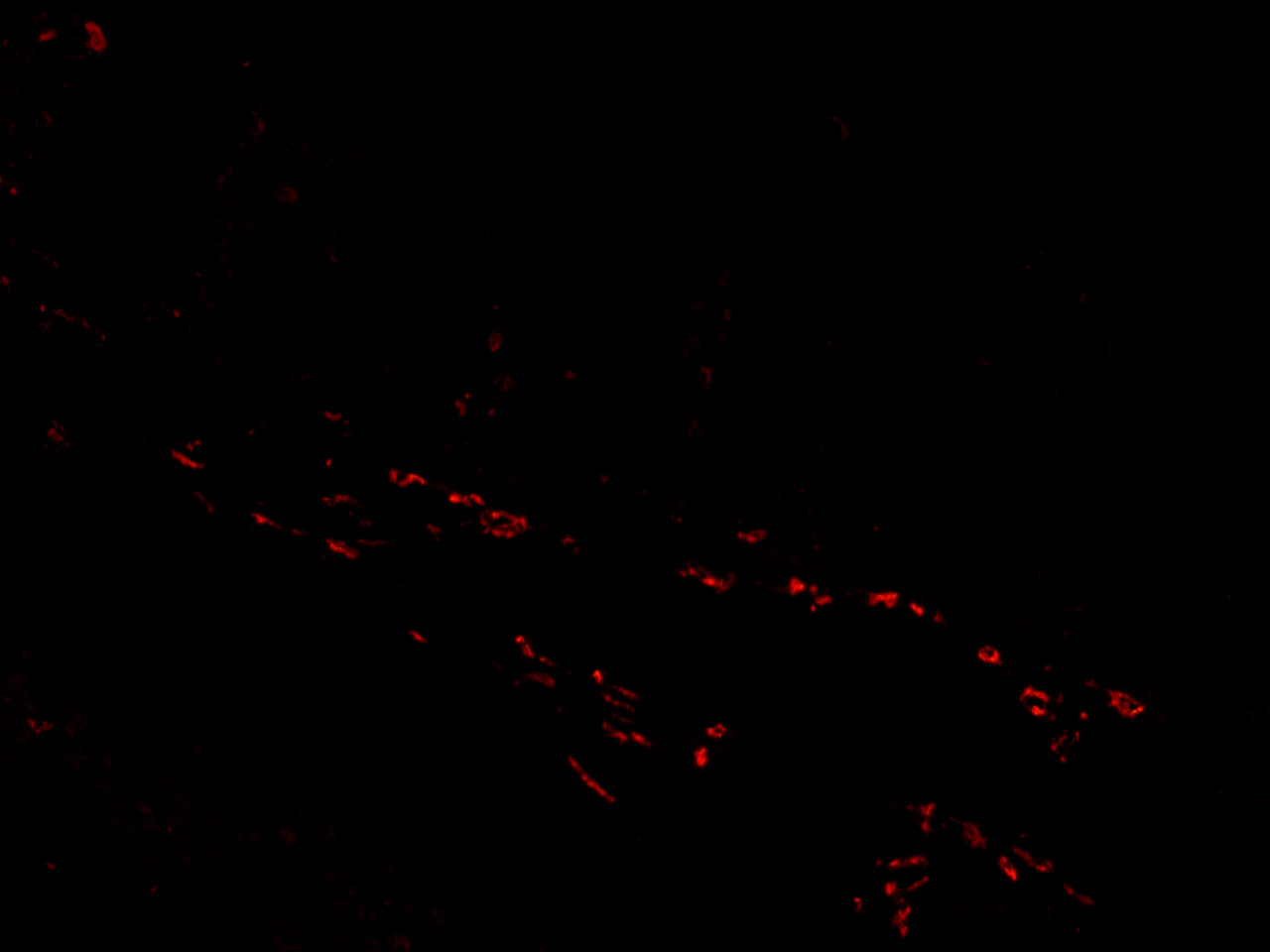

Supplement: Supplementary file 4 [file DataSheet5.ZIP › Supplementary_Material-original data3/FIGURE6/Figures 6J-K(Cecal-IF×200 )/Figure6-J-NLRP3-ASC/FOS/盲 Y2-12 NLRP3(绿)+ASC(红) 200-7.jpg]

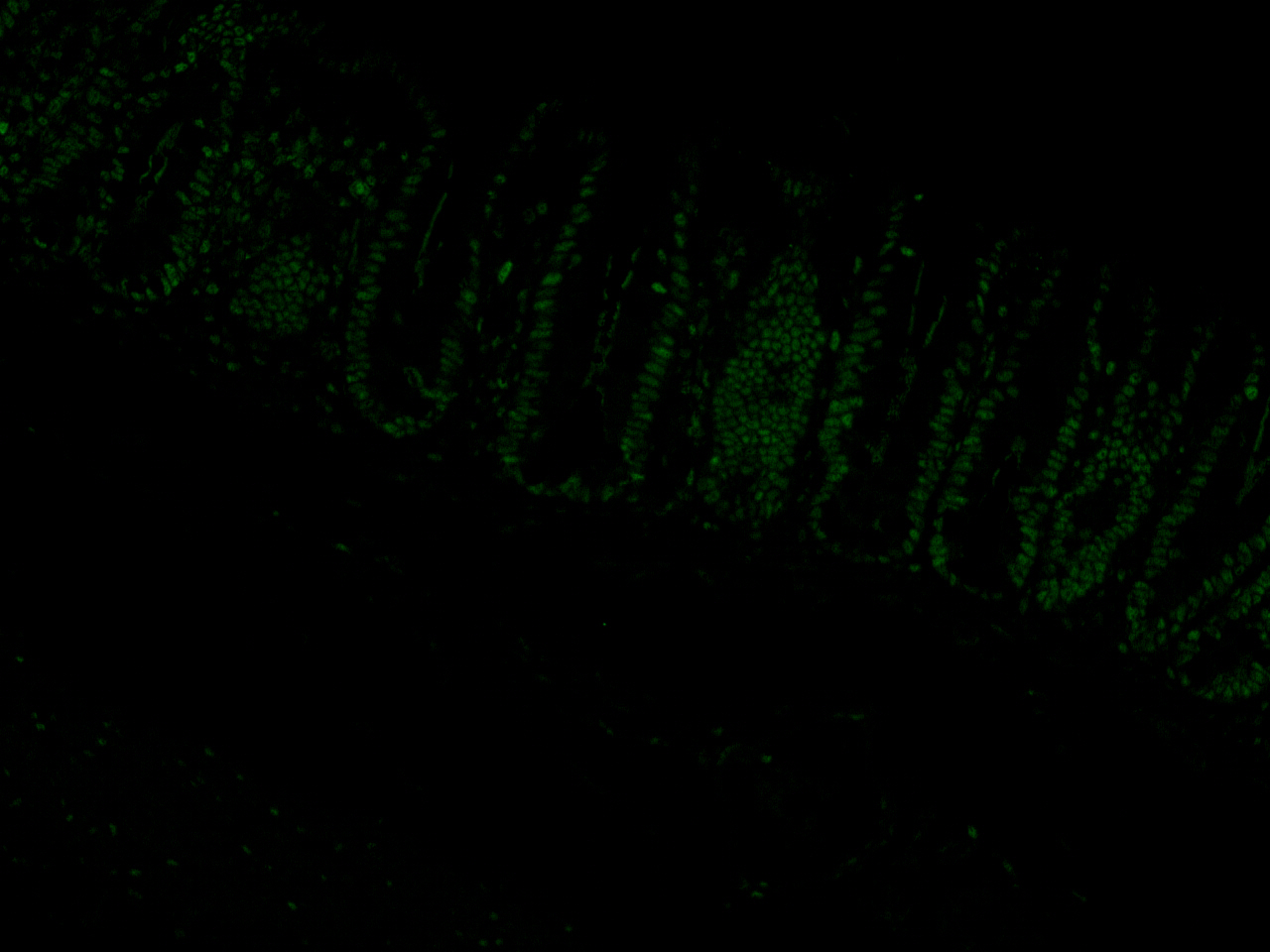

Supplement: Supplementary file 4 [file DataSheet5.ZIP › Supplementary_Material-original data3/FIGURE6/Figures 6J-K(Cecal-IF×200 )/Figure6-J-NLRP3-ASC/FOS/盲 Y2-12 NLRP3(绿)+ASC(红) 200-8.jpg]

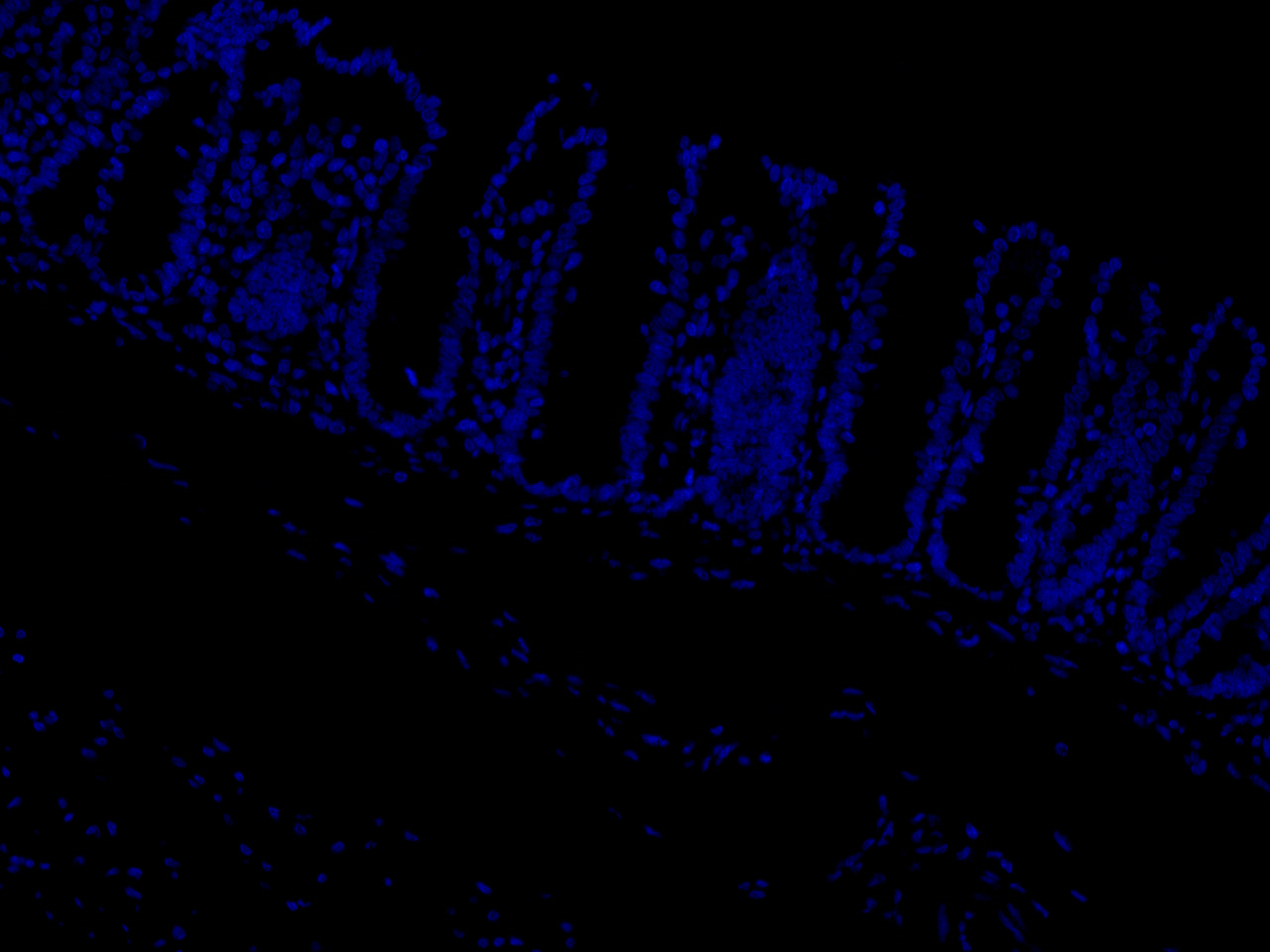

Supplement: Supplementary file 4 [file DataSheet5.ZIP › Supplementary_Material-original data3/FIGURE6/Figures 6J-K(Cecal-IF×200 )/Figure6-J-NLRP3-ASC/FOS/盲 Y2-12 NLRP3(绿)+ASC(红) 200-9.jpg]

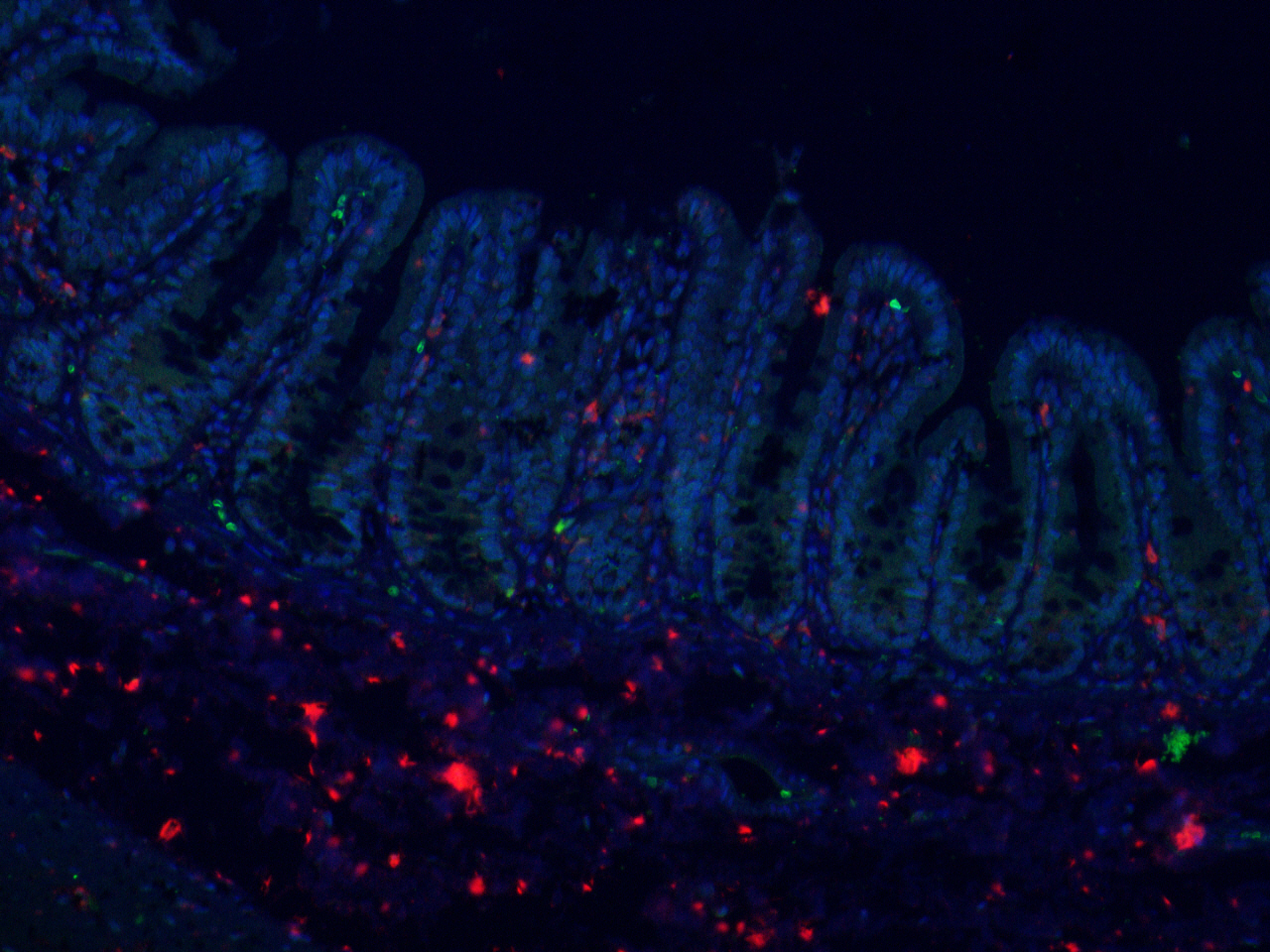

Supplement: Supplementary file 4 [file DataSheet5.ZIP › Supplementary_Material-original data3/FIGURE6/Figures 6J-K(Cecal-IF×200 )/Figure6-J-NLRP3-ASC/Model/CM2-11 NLRP3(绿)+ASC(红) 200-1 2 3.jpg]

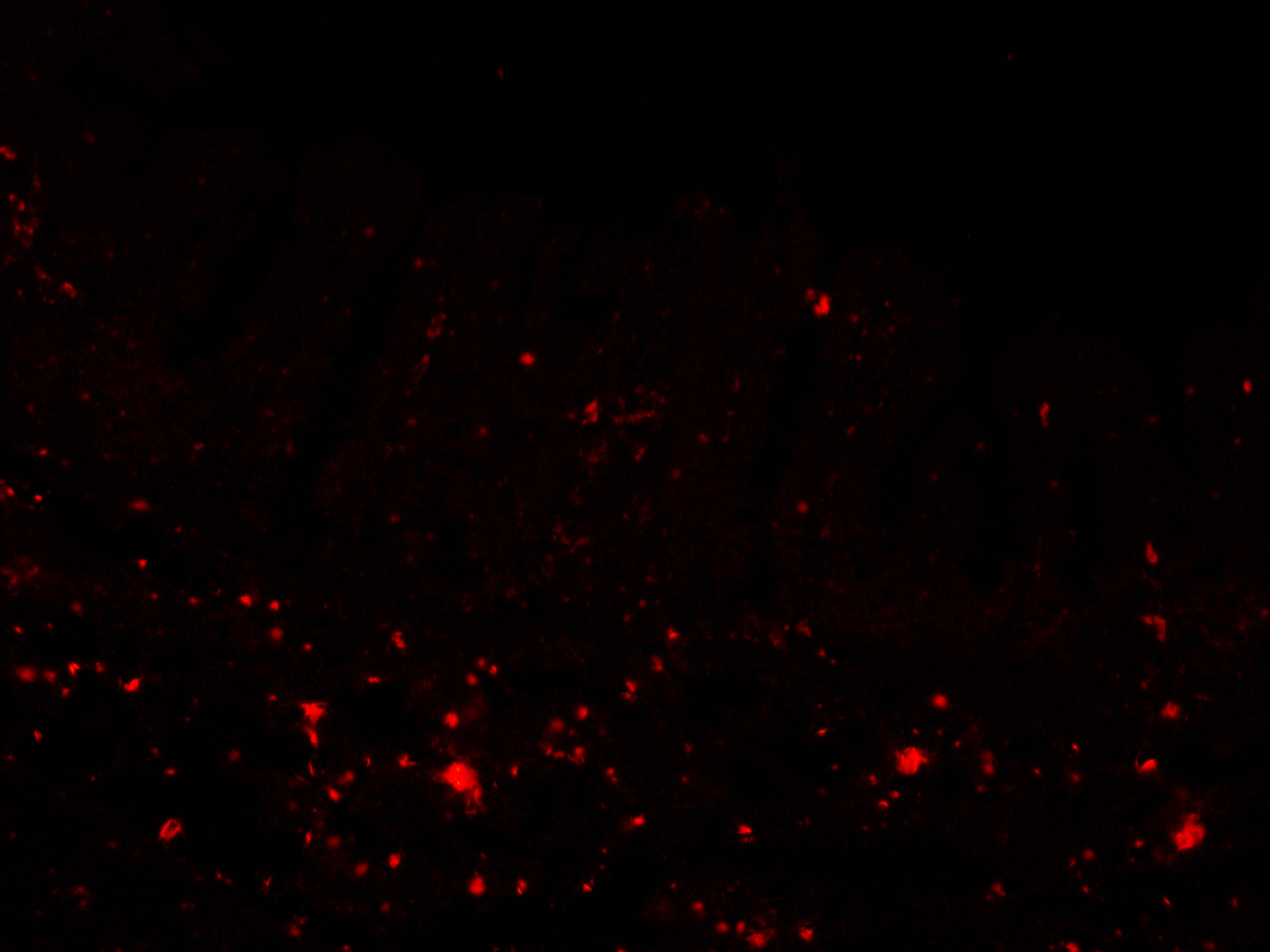

Supplement: Supplementary file 4 [file DataSheet5.ZIP › Supplementary_Material-original data3/FIGURE6/Figures 6J-K(Cecal-IF×200 )/Figure6-J-NLRP3-ASC/Model/CM2-11 NLRP3(绿)+ASC(红) 200-1.jpg]

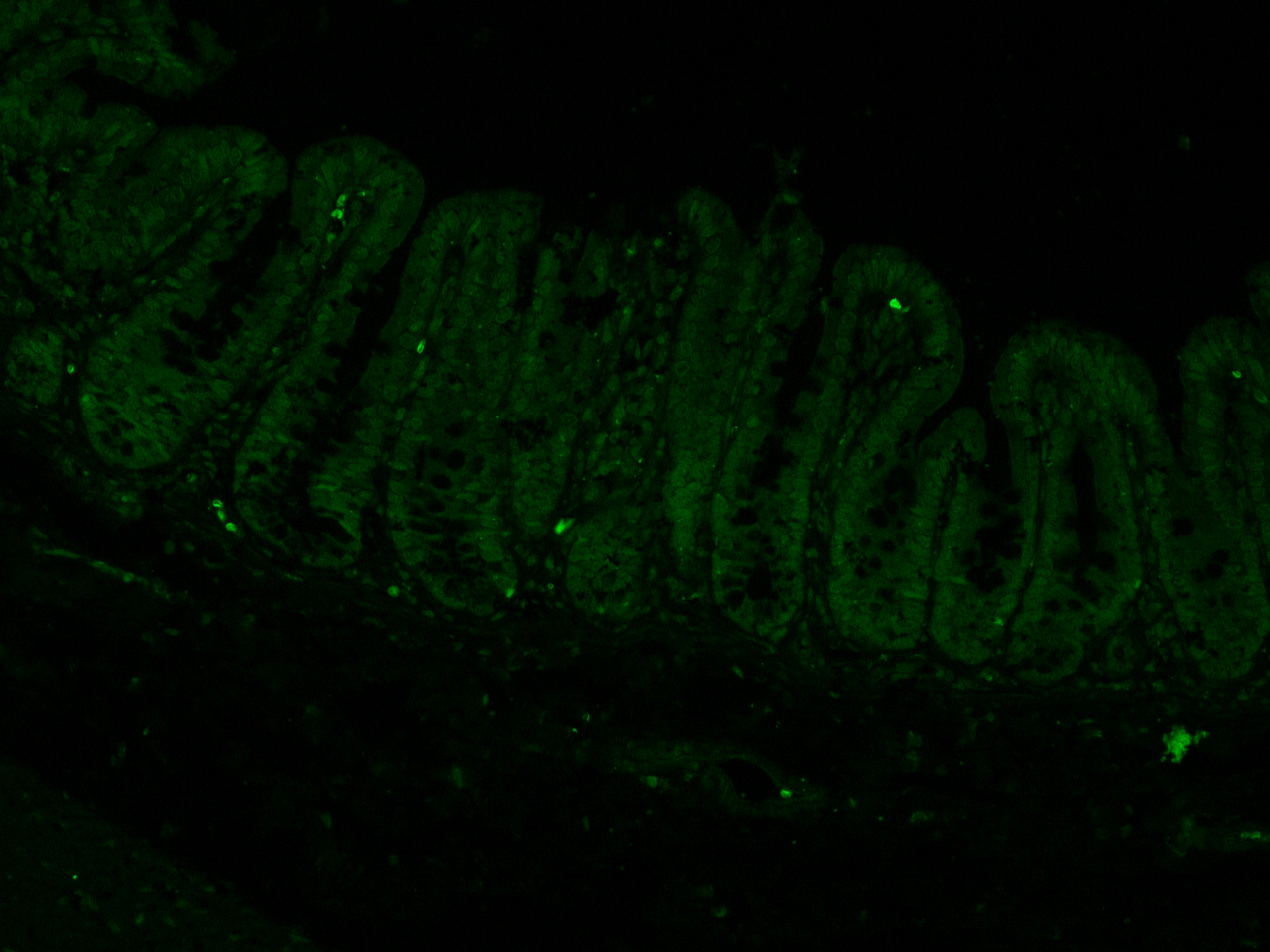

Supplement: Supplementary file 4 [file DataSheet5.ZIP › Supplementary_Material-original data3/FIGURE6/Figures 6J-K(Cecal-IF×200 )/Figure6-J-NLRP3-ASC/Model/CM2-11 NLRP3(绿)+ASC(红) 200-2.jpg]

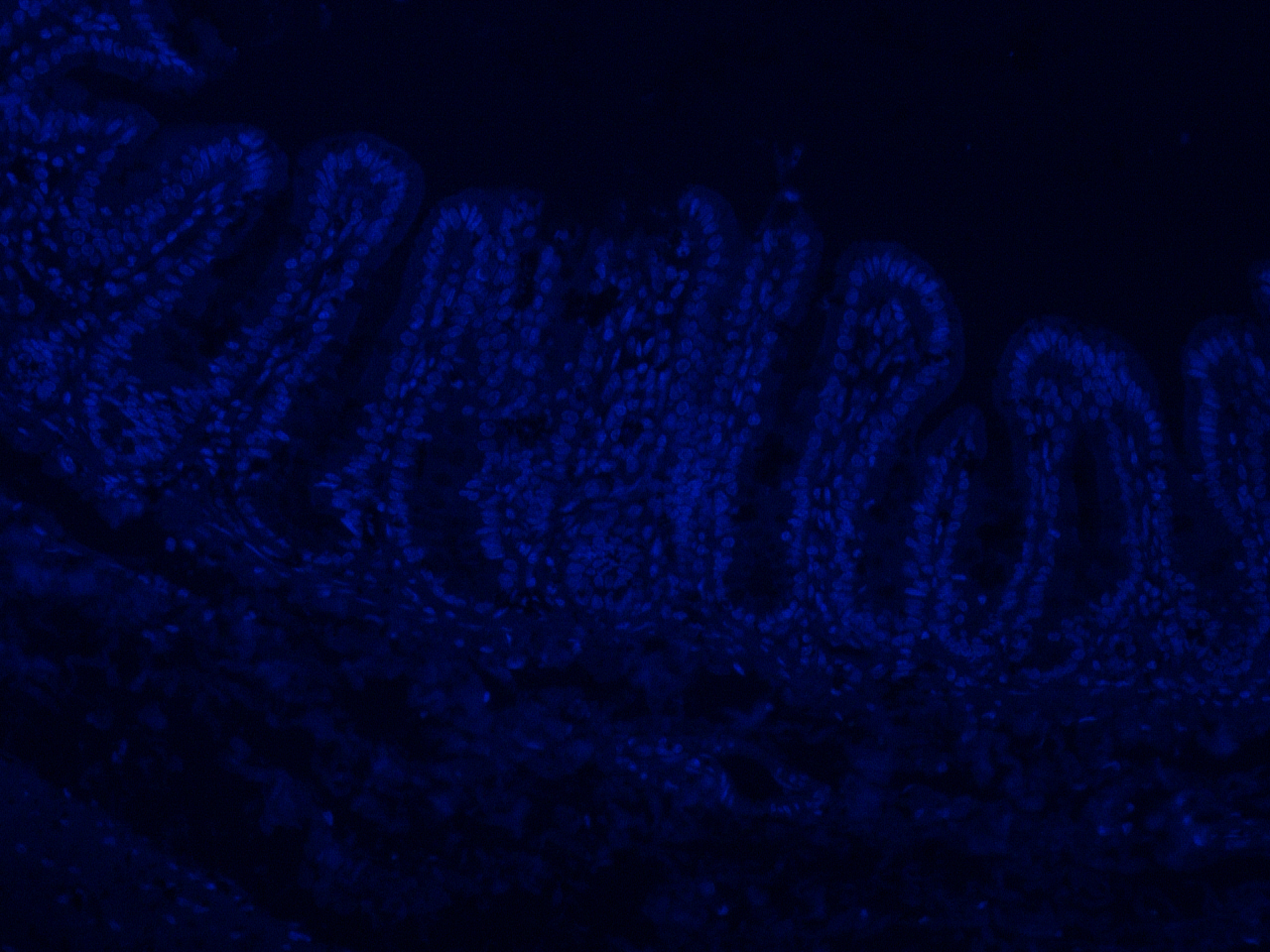

Supplement: Supplementary file 4 [file DataSheet5.ZIP › Supplementary_Material-original data3/FIGURE6/Figures 6J-K(Cecal-IF×200 )/Figure6-J-NLRP3-ASC/Model/CM2-11 NLRP3(绿)+ASC(红) 200-3.jpg]
