# Supplementary material for: Shugan Hewei Decoction Alleviates Cecum Mucosal Injury and Improves Depressive- and Anxiety-Like Behaviors in Chronic Stress Model Rats by Regulating Cecal Microbiota and Inhibiting NLRP3 Inflammasome
Source: Front Pharmacol. 2021 Dec 20;12:766474. doi: 10.3389/fphar.2021.766474 (PMC8721152; doi:10.3389/fphar.2021.766474)
Supplement: Supplementary file 2 [file DataSheet4.ZIP › Supplementary_Material-original data2/FIGURE2/Figure2A/Figure 2A.pptx]

## Slide 1
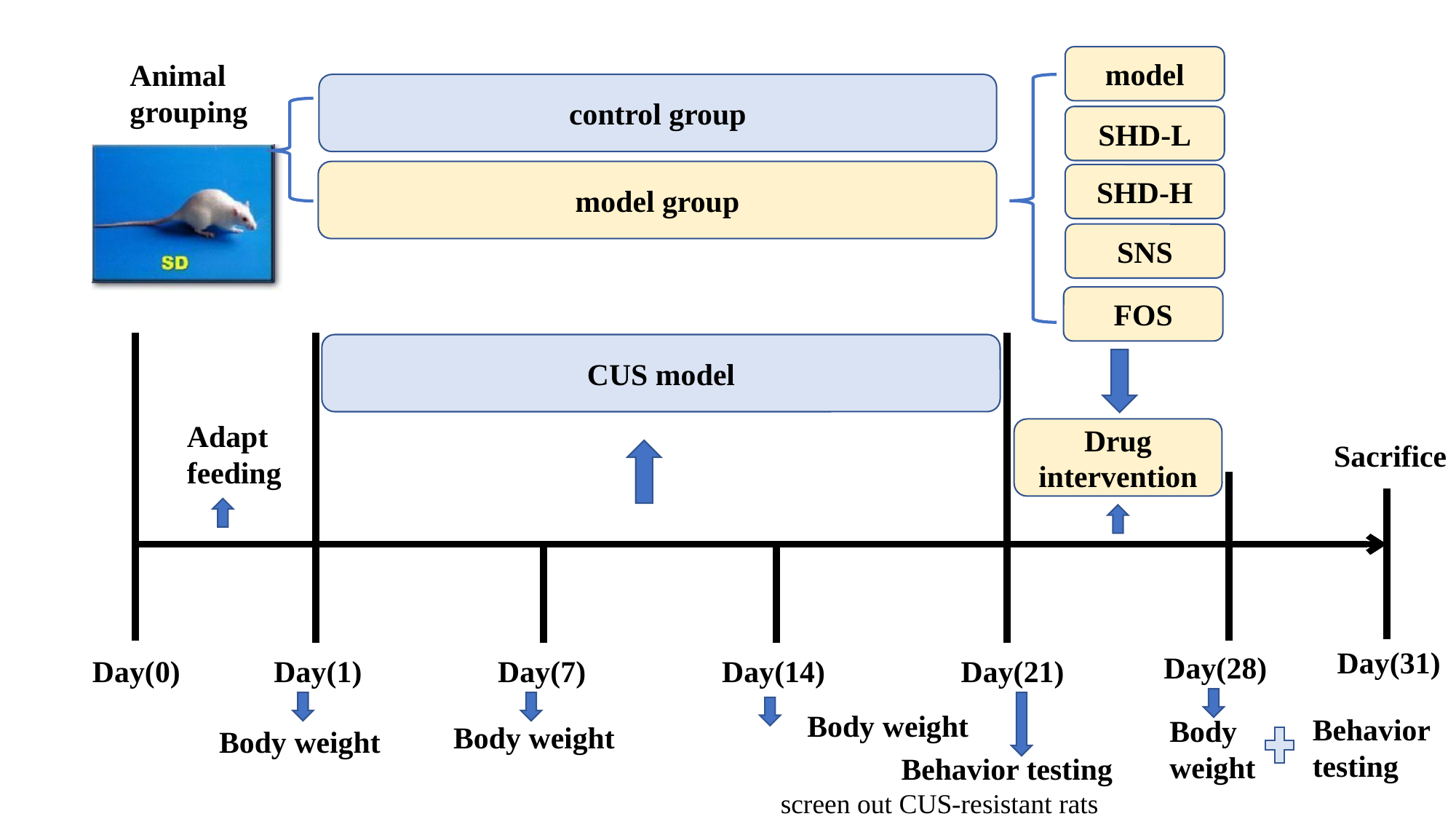

model
Animal grouping
control group
SHD-L
model group
SHD-H
SNS
FOS
CUS model
Adapt feeding
Drug intervention
Sacrifice
Day(31)
Day(28)
Day(0)
Day(1)
Day(7)
Day(14)
Day(21)
Body weight
Behavior testing
Body weight
Body weight
Body weight
Behavior testing
screen out CUS-resistant rats

## Slide 2
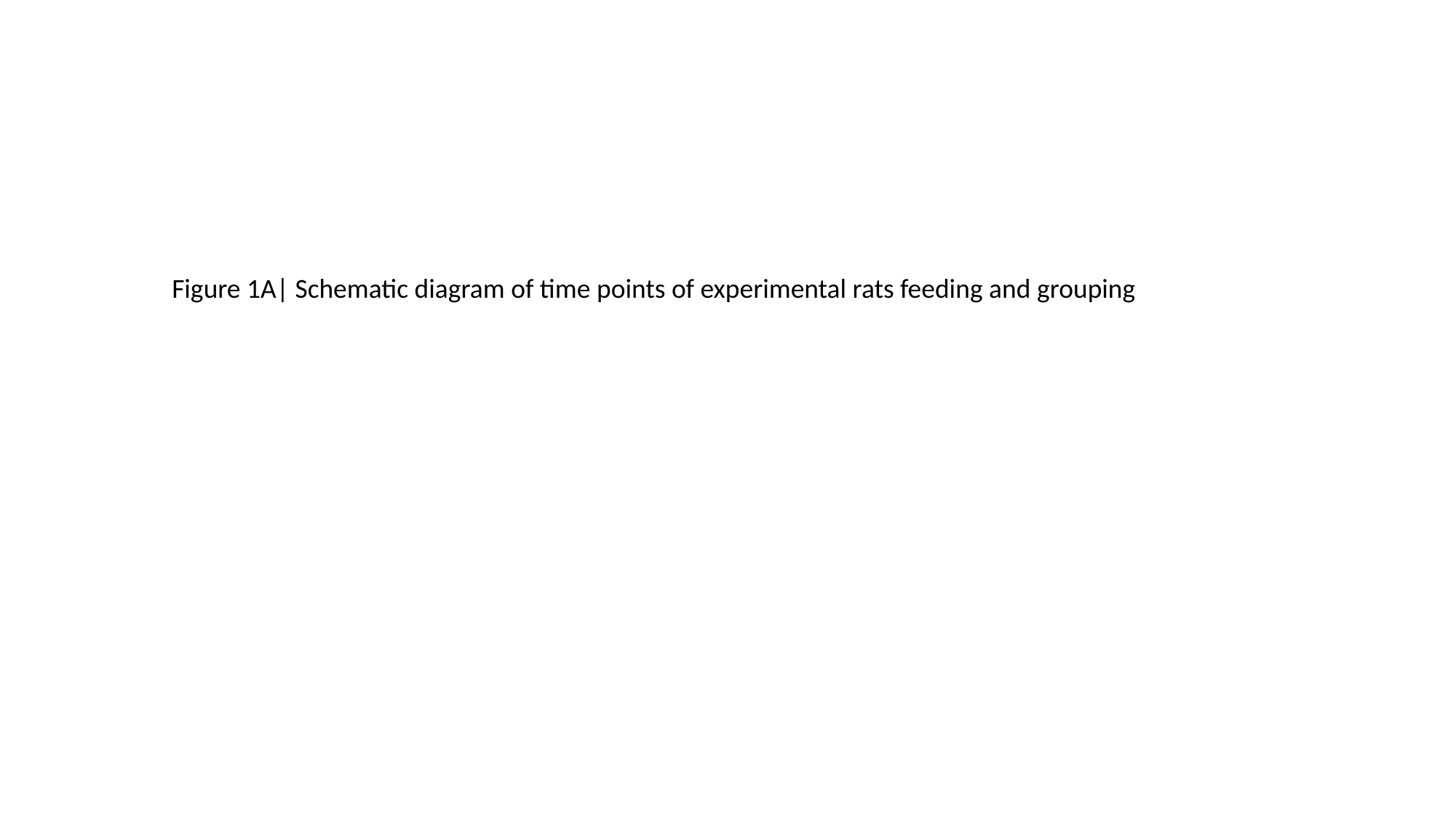

Figure 1A| Schematic diagram of time points of experimental rats feeding and grouping
