# Supplementary material for: Shugan Hewei Decoction Alleviates Cecum Mucosal Injury and Improves Depressive- and Anxiety-Like Behaviors in Chronic Stress Model Rats by Regulating Cecal Microbiota and Inhibiting NLRP3 Inflammasome
Source: Front Pharmacol. 2021 Dec 20;12:766474. doi: 10.3389/fphar.2021.766474 (PMC8721152; doi:10.3389/fphar.2021.766474)
Supplement: Supplementary file 2 [file DataSheet4.ZIP › Supplementary_Material-original data2/FIGURE4/Figures 4C,4D/Cecum Crypts Depth&Density-date.docx]

| Name | Cecum Crypts Depth（mm） | Cecum Crypts Numbers | Cecum Mucosal area（mm²） | Cecum Crypts Density（n/mm²） |
| --- | --- | --- | --- | --- |
| ①Control-10 100-3 | 0.1727983 | 28 | 0.23914 | 117.08623 |
| ①Control-11 100-1 | 0.2044043 | 32 | 0.30935 | 103.44308 |
| ①Control-12 100-2 | 0.1725903 | 29 | 0.27009 | 107.37162 |
| ①Control-13 100-2 | 0.1653042 | 30 | 0.27732 | 108.17829 |
| ①Control-9 100-3 | 0.2023136 | 31 | 0.29352 | 105.61461 |
| ②Model-10 100-2 | 0.2168565 | 23 | 0.28844 | 79.73929 |
| ②Model-11 100-3 | 0.2181969 | 25 | 0.38792 | 64.44628 |
| ②Model-12 100-1 | 0.2067866 | 21 | 0.28341 | 74.09661 |
| ②Model-13 100-3 | 0.179405 | 18 | 0.20754 | 86.72890 |
| ②Model-9 100-1 | 0.2153343 | 25 | 0.30081 | 83.10963 |
| ③SHD-L-10 100-1 | 0.1932501 | 31 | 0.25515 | 121.49599 |
| ③SHD-L-11 100-3 | 0.1803075 | 15 | 0.16731 | 89.65596 |
| ③SHD-L-12 100-2 | 0.1555726 | 30 | 0.31371 | 95.63026 |
| ③SHD-L-13 100-1 | 0.1996622 | 23 | 0.27317 | 84.19656 |
| ③SHD-L-9 100-3 | 0.1387452 | 13 | 0.14230 | 91.35553 |
| ④SHD-H-10 100-3 | 0.1709261 | 35 | 0.31989 | 109.41173 |
| ④SHD-H-11 100-2 | 0.2076185 | 40 | 0.31806 | 125.76338 |
| ④SHD-H-13 100-2 | 0.160615 | 36 | 0.25126 | 143.27788 |
| ④SHD-H-12 100-3 | 0.1527024 | 28 | 0.20533 | 136.36585 |
| ④SHD-H-9 100-2 | 0.1695254 | 19 | 0.19594 | 96.97081 |
| ⑤SNS-1 100-1 | 0.1480021 | 17 | 0.17383 | 97.79415 |
| ⑤SNS-11 100-1 | 0.1903293 | 35 | 0.29926 | 116.95607 |
| ⑤SNS-3 100-1 | 0.1510853 | 30 | 0.22891 | 131.05752 |
| ⑤SNS-7 100-2 | 0.1782242 | 39 | 0.33419 | 116.70001 |
| ⑤SNS-8 100-2 | 0.1741149 | 34 | 0.27427 | 123.96713 |
| ⑥FOS-1 100-3 | 0.1589339 | 19 | 0.21033 | 90.33400 |
| ⑥FOS-2 100-3 | 0.1537056 | 13 | 0.18549 | 70.08357 |
| ⑥FOS-10 100-1 | 0.1765643 | 16 | 0.23286 | 68.71102 |
| ⑥FOS-4 100-2 | 0.1537242 | 26 | 0.26172 | 99.34407 |
| ⑥FOS-5 100-3 | 0.1511138 | 16 | 0.19094 | 83.79458 |
