# Supplementary material for: Shugan Hewei Decoction Alleviates Cecum Mucosal Injury and Improves Depressive- and Anxiety-Like Behaviors in Chronic Stress Model Rats by Regulating Cecal Microbiota and Inhibiting NLRP3 Inflammasome
Source: Front Pharmacol. 2021 Dec 20;12:766474. doi: 10.3389/fphar.2021.766474 (PMC8721152; doi:10.3389/fphar.2021.766474)
Supplement: Supplementary file 2 [file DataSheet4.ZIP › Supplementary_Material-original data2/FIGURE5/Figure 5I.pdf]

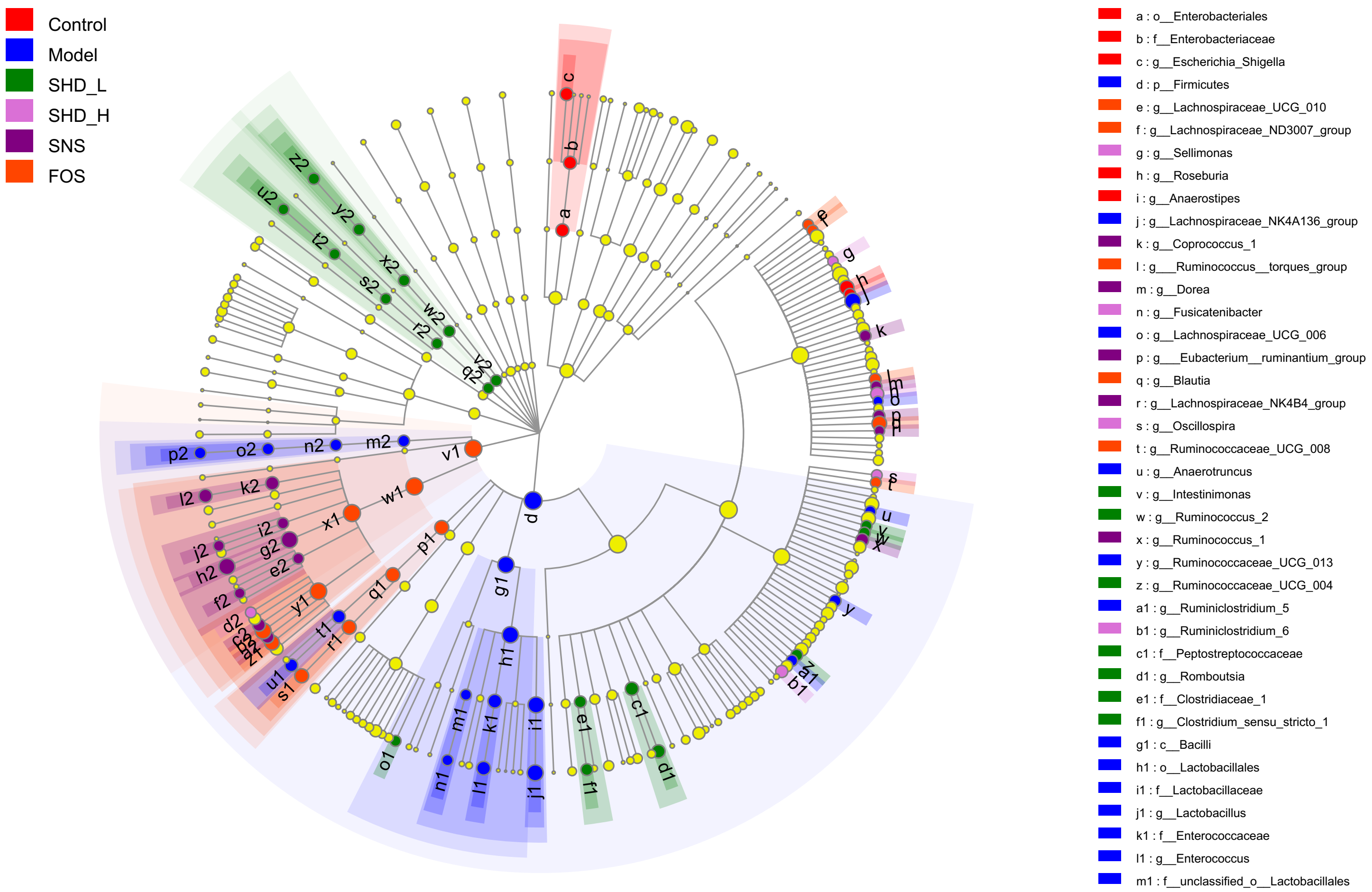
Control
Model
SHD_L
SHD_H
SNS
FOS
a : o__Enterobacteriales
b : f__Enterobacteriaceae
c : g__Escherichia_Shigella
d : p__Firmicutes
e : g__Lachnospiraceae_UCG_010
f : g__Lachnospiraceae_ND3007_group
g : g__Sellimonas
h : g__Roseburia
i : g__Anaerostipes
j : g__Lachnospiraceae_NK4A136_group
k : g__Coprococcus_1
l : g___Ruminococcus__torques_group
m : g__Dorea
n : g__Fusicatenibacter
o : g__Lachnospiraceae_UCG_006
p : g___Eubacterium__ruminantium_group
q : g__Blautia
r : g__Lachnospiraceae_NK4B4_group
s : g__Oscillospira
t : g__Ruminococcaceae_UCG_008
u : g__Anaerotruncus
v : g__Intestinimonas
w : g__Ruminococcus_2
x : g__Ruminococcus_1
y : g__Ruminococcaceae_UCG_013
z : g__Ruminococcaceae_UCG_004
a1 : g__Ruminiclostridium_5
b1 : g__Ruminiclostridium_6
c1 : f__Peptostreptococcaceae
d1 : g__Romboutsia
e1 : f__Clostridiaceae_1
f1 : g__Clostridium_sensu_stricto_1
g1 : c__Bacilli
h1 : o__Lactobacillales
i1 : f__Lactobacillaceae
j1 : g__Lactobacillus
k1 : f__Enterococcaceae
l1 : g__Enterococcus
m1 : f__unclassified_o__Lactobacillales
