# Supplementary material for: Shugan Hewei Decoction Alleviates Cecum Mucosal Injury and Improves Depressive- and Anxiety-Like Behaviors in Chronic Stress Model Rats by Regulating Cecal Microbiota and Inhibiting NLRP3 Inflammasome
Source: Front Pharmacol. 2021 Dec 20;12:766474. doi: 10.3389/fphar.2021.766474 (PMC8721152; doi:10.3389/fphar.2021.766474)
Supplement: Supplementary file 2 [file DataSheet4.ZIP › Supplementary_Material-original data2/FIGURE6/Figures 6E-I(Cecal-PCR )/RT-PCR-Report.docx]

**荧光定量PCR检测报告**

**客**

**目录**

样品及引物信息 4

实验仪器 5

试剂与耗材 5

实验步骤 6

结果展示 8

样品及引物信息

| **样本编号** | **样本来源** | **样本类型** | **样本量** |
| --- | --- | --- | --- |
| 膜1-3 | Rat | 组织 | 20mg |
| 膜1-5 | Rat | 组织 | 20mg |
| 膜1-6 | Rat | 组织 | 20mg |
| 膜1-7 | Rat | 组织 | 20mg |
| 膜1-8 | Rat | 组织 | 20mg |
| 膜2-1 | Rat | 组织 | 20mg |
| 膜2-2 | Rat | 组织 | 20mg |
| 膜2-3 | Rat | 组织 | 20mg |
| 膜2-4 | Rat | 组织 | 20mg |
| 膜2-5 | Rat | 组织 | 20mg |
| 膜3-1 | Rat | 组织 | 20mg |
| 膜3-2 | Rat | 组织 | 20mg |
| 膜3-3 | Rat | 组织 | 20mg |
| 膜3-4 | Rat | 组织 | 20mg |
| 膜3-5 | Rat | 组织 | 20mg |
| 膜4-1 | Rat | 组织 | 20mg |
| 膜4-2 | Rat | 组织 | 20mg |
| 膜4-3 | Rat | 组织 | 20mg |
| 膜4-4 | Rat | 组织 | 20mg |
| 膜4-5 | Rat | 组织 | 20mg |
| 膜5-1 | Rat | 组织 | 20mg |
| 膜5-2 | Rat | 组织 | 20mg |
| 膜5-3 | Rat | 组织 | 20mg |
| 膜5-4 | Rat | 组织 | 20mg |
| 膜5-5 | Rat | 组织 | 20mg |
| 膜6-1 | Rat | 组织 | 20mg |
| 膜6-2 | Rat | 组织 | 20mg |
| 膜6-3 | Rat | 组织 | 20mg |
| 膜6-4 | Rat | 组织 | 20mg |
| 膜6-5 | Rat | 组织 | 20mg |

| **引物名称** | **序列** |
| --- | --- |
| NLRP3-F | AGGAGTTCTTTGCGGCTAT |
| NLRP3-R | GACCTTCACGTCTCGGTTC |
| caspase-1-F | CAGGAGGGAATATGTGGG |
| caspase-1-R | TAACCTTGGGCTTGTCTT |
| IL-1β-F | GGATGAGGACCCAAGCAC |
| IL-1β-R | CAGACAGCACGAGGCATT |
| ASC-F | CCCCATAGACCTCACTGATAAA |
| ASC-R | CACAGCTCCAGACTCTTCCA |
| IL-18-F | TCAGACCACTTTGGCAGAC |
| IL-18-R | CACAGGCGGGTTTCTTTT |
| GAPDH -F | AAGTTCAACGGCACAGTCAA |
| GAPDH -R | TCTCGCTCCTGGAAGATGG |

实验仪器

| **仪器名称** | **品牌** | **型号** |
| --- | --- | --- |
| 研磨仪 | MP Bio | FastPreP-24 |
| 分光光度计 | Thermo Fisher | Nanodrop2000 |
| 冷冻离心机 | Beckman Coulter | Microfuge 20R |
| 凝胶成像仪 | Bio-Rad | Chemidoc XRS+ |
| 荧光定量PCR仪 | Roche | LightCycler96 |
| 采集及分析软件 | Roche | LightCycler® 96 SW 1.1 |

试剂与耗材

| **试剂名称** | **品牌** | **货号** |
| --- | --- | --- |
| Trizol | TAKARA | 9109 |
| MonScript^TM^5× RTⅣall-in-one Mix | Monad | RN05006M |
| MonAmp^TM^SYBR Green qPCR Mix | Monad | RN04005M |
| MonScript^TM^ RTase Ⅲ  RiboLock RNase Inhibitor  dNTP Mixture | Monad   Fermentas  TIANGEN | RN05002M  EO0381  CD117 |
| **耗材名称** | **品牌** | **货号** |
| 96孔板 | Monad | CN020202S |
| 96孔板膜 | Monad | CG010102M |

实验步骤

**1．样品反转录**

**Table 1 Reaction setup**

| **Component** | **Volume Per Reaction (μL)** |
| --- | --- |
| MonScript^TM^5× RTⅣall-in-one Mix | 4 |
| dsDNase | 1 |
| RNA template | 1μg |
| Nuclease-Free water | To 20 μL |

**Table 2 Reaction setup**

| **Component** | **Volume Per Reaction** |
| --- | --- |
| MonScript^TM^ RTase Ⅲ | 1 μL |
| MonScript^TM^ 5×M-MLV First Strand Buffer | 4 μL |
| 0.1M DTT | 1 μL |
| RiboLock RNase Inhibitor | 1 μL |
| dNTP Mixture | 1 μL |
| RNA template | 1 μg |
| Primers | 0.25μM |
| H_2_O | To 20 μL |

注意：Table 1为常规反转录体系，Table 2为microRNA反转录体系。

**2．荧光定量检测**

**Table3 Reaction setup**

| **Component** | **Volume Per Reaction** |
| --- | --- |
| MonAmp^TM^SYBR Green qPCR Mix2× | 5 μL |
| Forward and reverse primers | 0.4 μL |
| DNA template | 0.5 μL |
| H_2_O | To 10 μL |

**Table4 Reaction setup**

| **Temperature (^o^C)** | **Time** | **Cycles** |
| --- | --- | --- |
| 95 | 10 min |  |
| 95 | 10 sec | 40 |
| 60 | 60 sec. |  |
| 65 ^o^C -95 ^o^C | 0.5^o^C /5S |  |

**3．检测和数据分析**

将96孔板放入荧光定量PCR仪中，并在LightCycler96仪器上设定检测模式为SYBR，检测SYBR的荧光信号。仪器自动分析每个样品荧光信号，然后由LightCycler® 96 SW 1.1完成对数据的自动处理。

结果展示


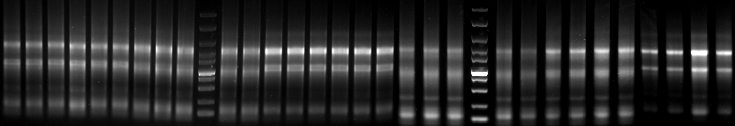


图1-2. 肠膜1-3到6-5 RNA完整性验证电泳图


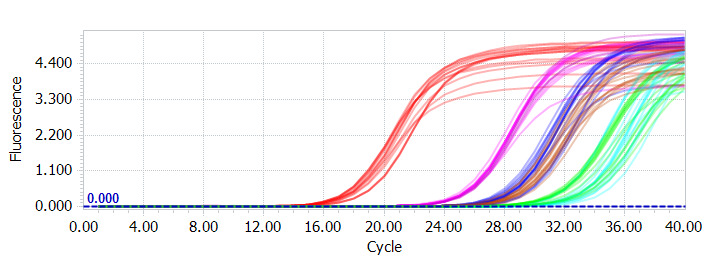


图2. 扩增曲线


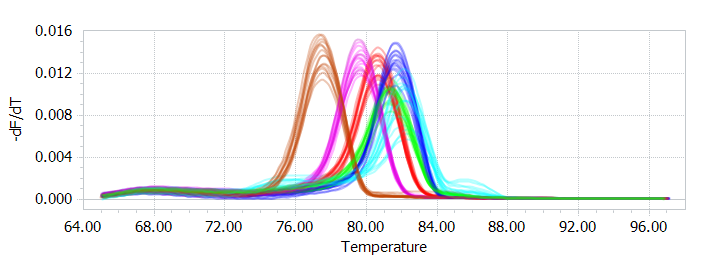


图3. 6对引物熔解曲线
