# Supplementary figures and images for: Shugan Hewei Decoction Alleviates Cecum Mucosal Injury and Improves Depressive- and Anxiety-Like Behaviors in Chronic Stress Model Rats by Regulating Cecal Microbiota and Inhibiting NLRP3 Inflammasome (part 2 of 2)
Source: Front Pharmacol. 2021 Dec 20;12:766474. doi: 10.3389/fphar.2021.766474 (PMC8721152; doi:10.3389/fphar.2021.766474)

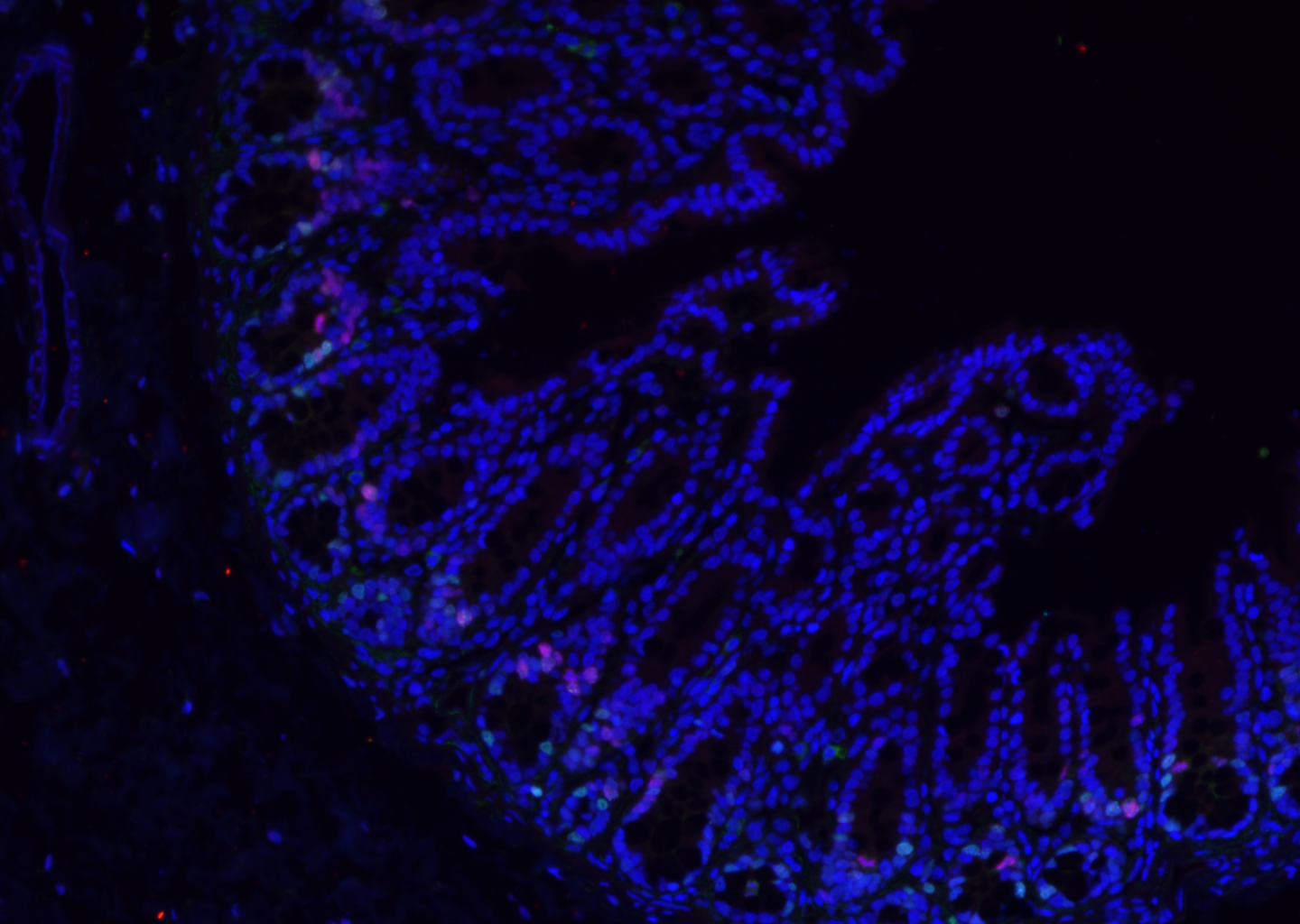

Supplement: Supplementary file 4 [file DataSheet5.ZIP › Supplementary_Material-original data3/FIGURE6/Figures 6J-K(Cecal-IF×200 )/Figure6-J-NLRP3-ASC/SHD-H/CM4-9 NLRP3(绿光)+ASC(红光) 200-1 2 3.tif]

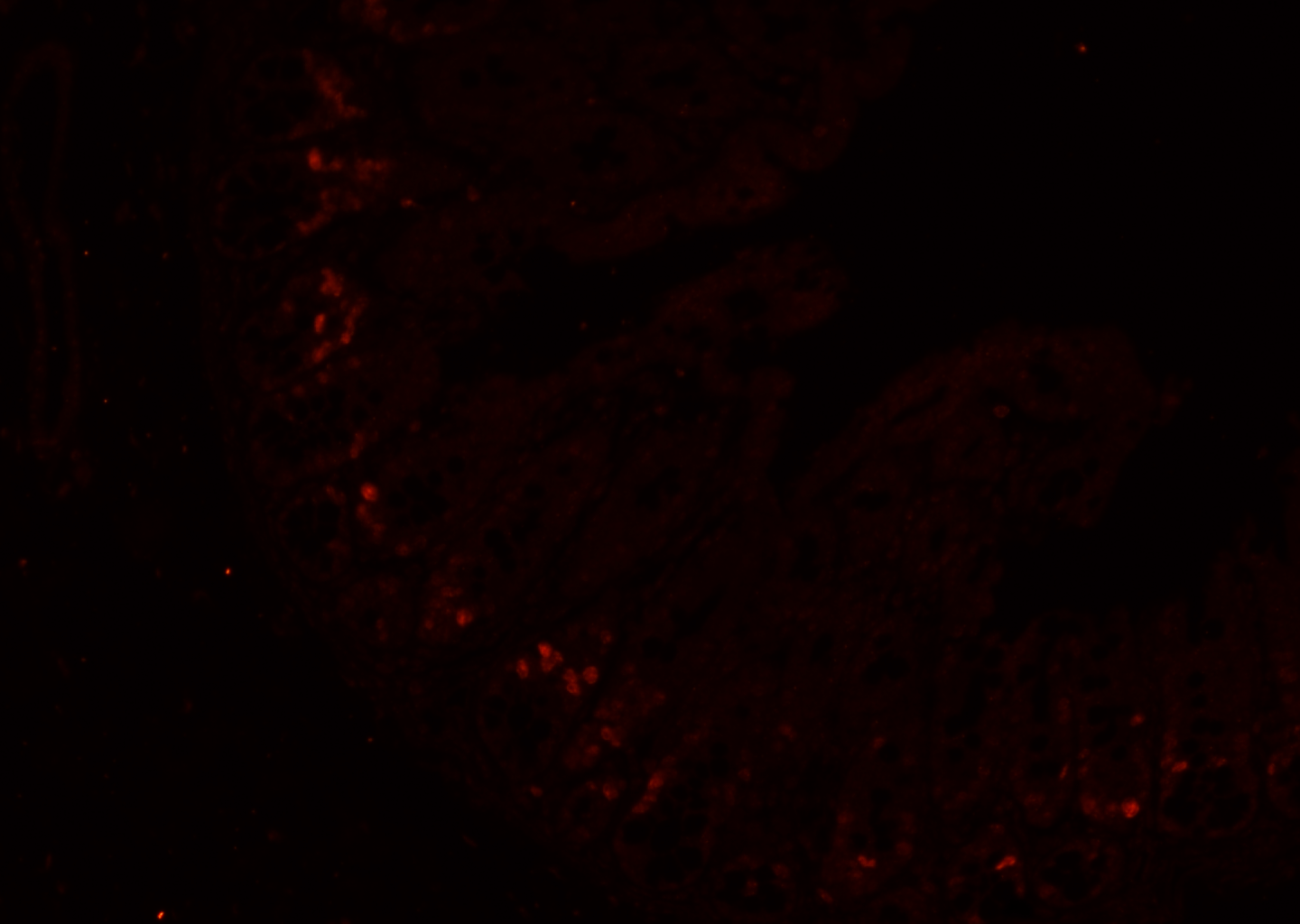

Supplement: Supplementary file 4 [file DataSheet5.ZIP › Supplementary_Material-original data3/FIGURE6/Figures 6J-K(Cecal-IF×200 )/Figure6-J-NLRP3-ASC/SHD-H/CM4-9 NLRP3(绿光)+ASC(红光) 200-1.tif]

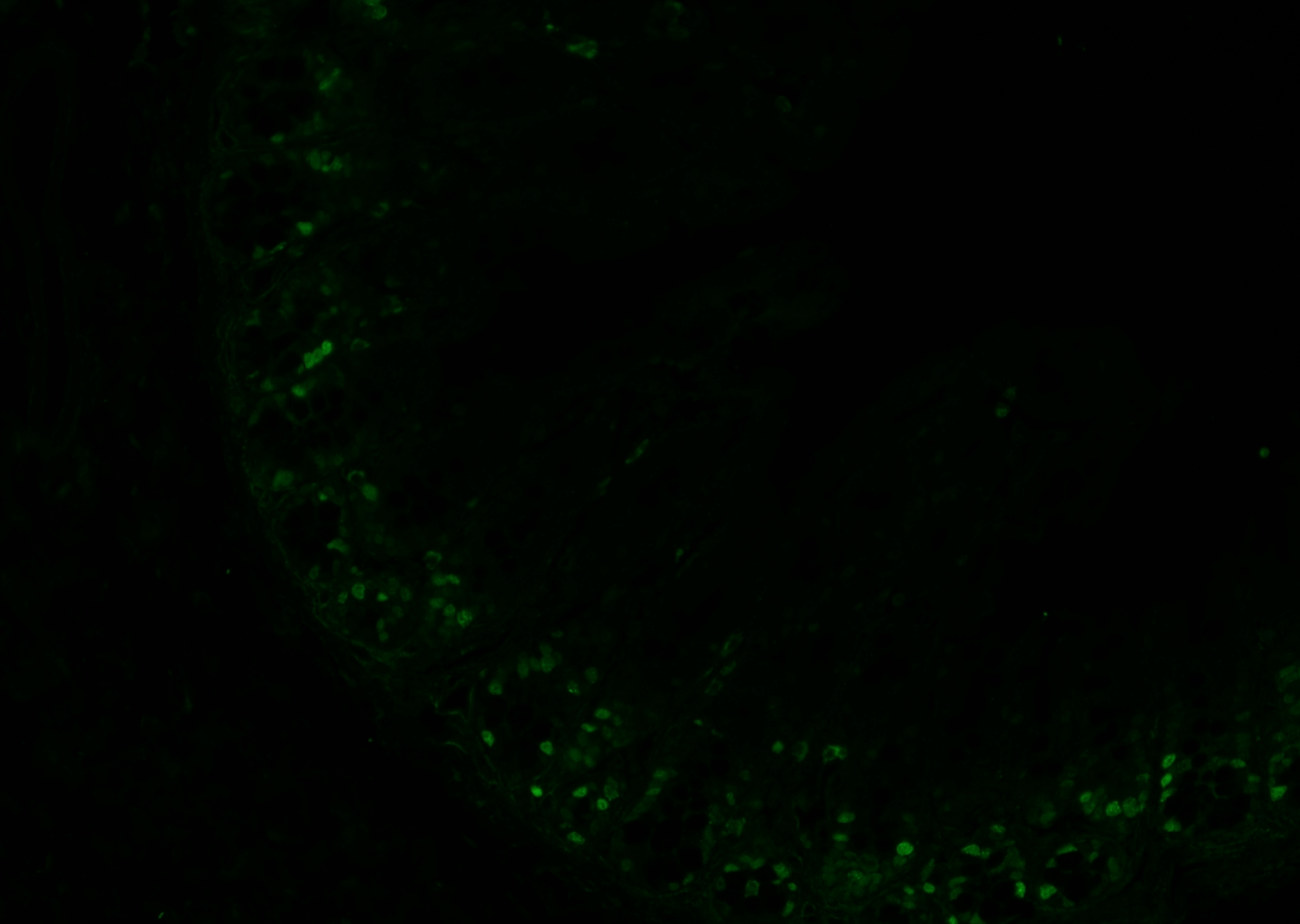

Supplement: Supplementary file 4 [file DataSheet5.ZIP › Supplementary_Material-original data3/FIGURE6/Figures 6J-K(Cecal-IF×200 )/Figure6-J-NLRP3-ASC/SHD-H/CM4-9 NLRP3(绿光)+ASC(红光) 200-2.tif]

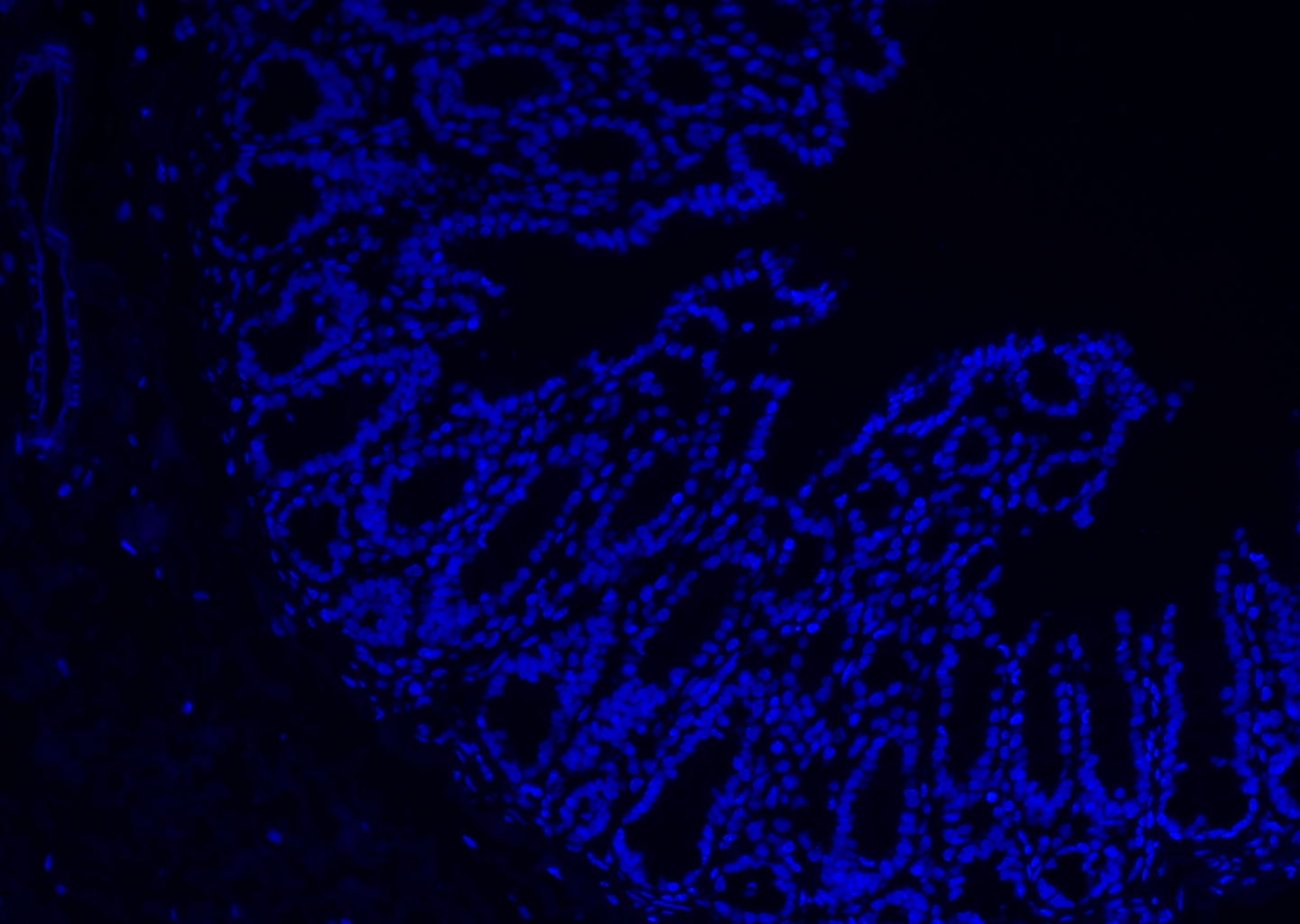

Supplement: Supplementary file 4 [file DataSheet5.ZIP › Supplementary_Material-original data3/FIGURE6/Figures 6J-K(Cecal-IF×200 )/Figure6-J-NLRP3-ASC/SHD-H/CM4-9 NLRP3(绿光)+ASC(红光) 200-3.tif]

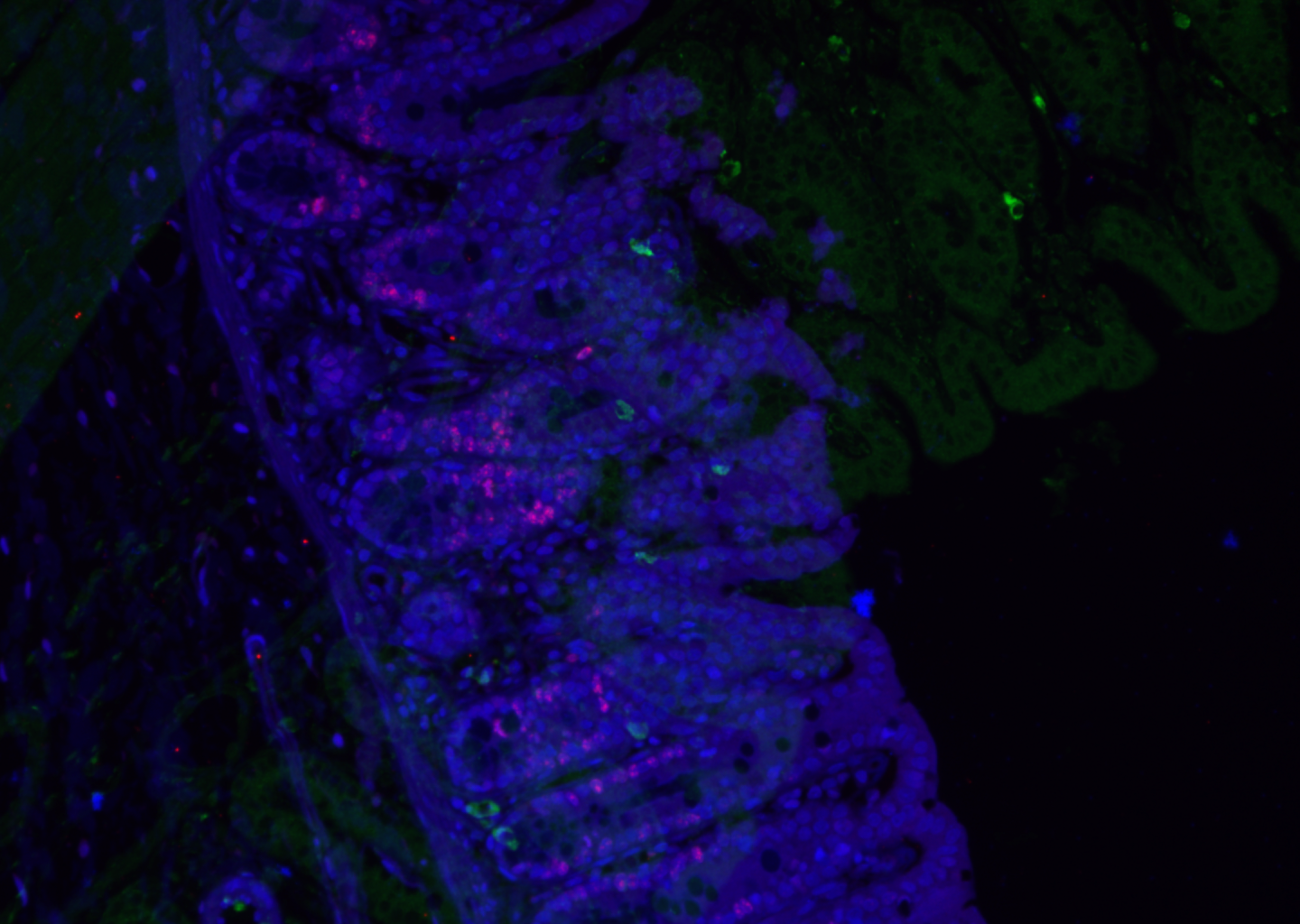

Supplement: Supplementary file 4 [file DataSheet5.ZIP › Supplementary_Material-original data3/FIGURE6/Figures 6J-K(Cecal-IF×200 )/Figure6-J-NLRP3-ASC/SHD-L/CM3-13 NLRP3(绿光)+ASC(红光) 200-4 5 6.tif]

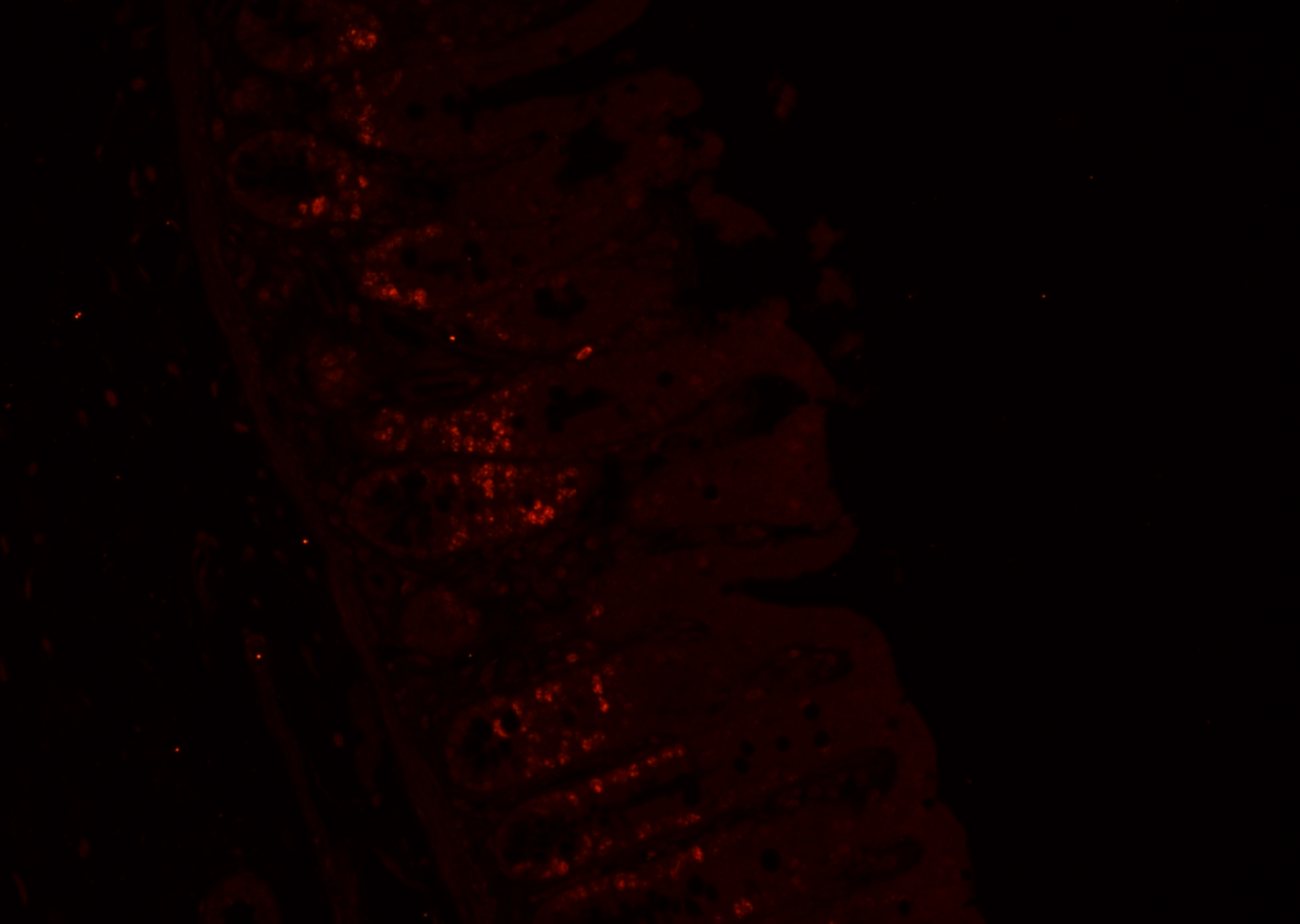

Supplement: Supplementary file 4 [file DataSheet5.ZIP › Supplementary_Material-original data3/FIGURE6/Figures 6J-K(Cecal-IF×200 )/Figure6-J-NLRP3-ASC/SHD-L/CM3-13 NLRP3(绿光)+ASC(红光) 200-4.tif]

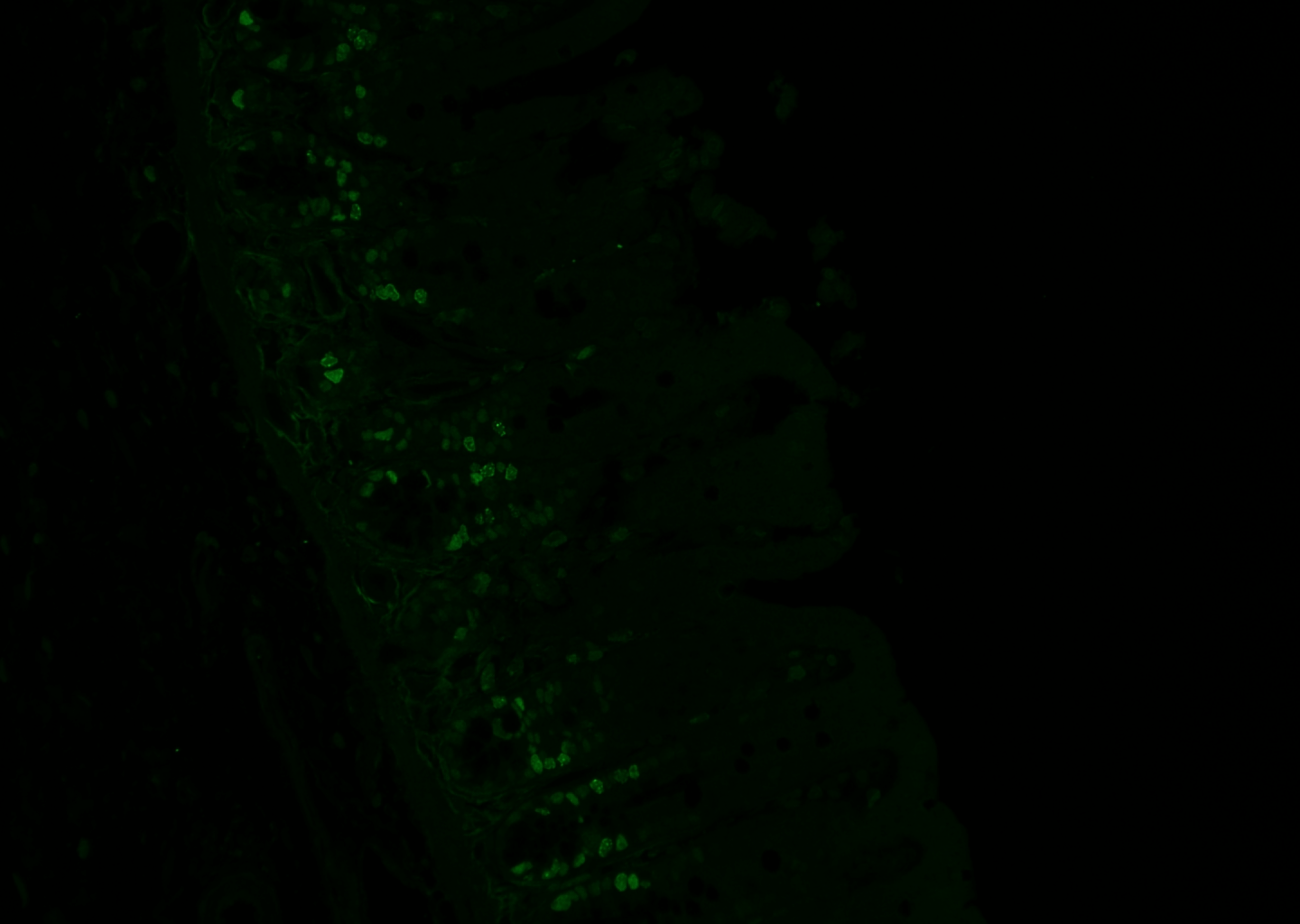

Supplement: Supplementary file 4 [file DataSheet5.ZIP › Supplementary_Material-original data3/FIGURE6/Figures 6J-K(Cecal-IF×200 )/Figure6-J-NLRP3-ASC/SHD-L/CM3-13 NLRP3(绿光)+ASC(红光) 200-5.tif]

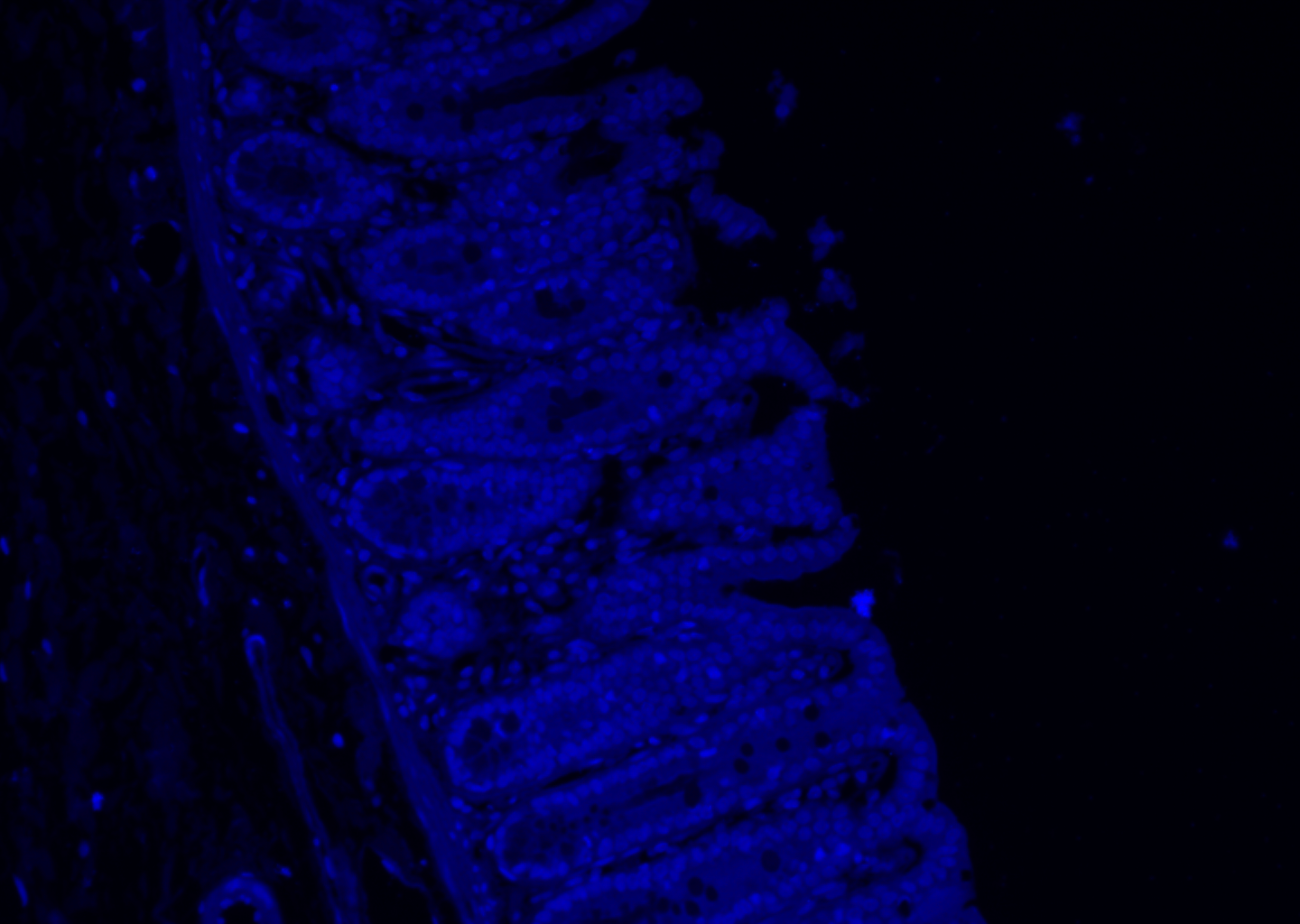

Supplement: Supplementary file 4 [file DataSheet5.ZIP › Supplementary_Material-original data3/FIGURE6/Figures 6J-K(Cecal-IF×200 )/Figure6-J-NLRP3-ASC/SHD-L/CM3-13 NLRP3(绿光)+ASC(红光) 200-6.tif]

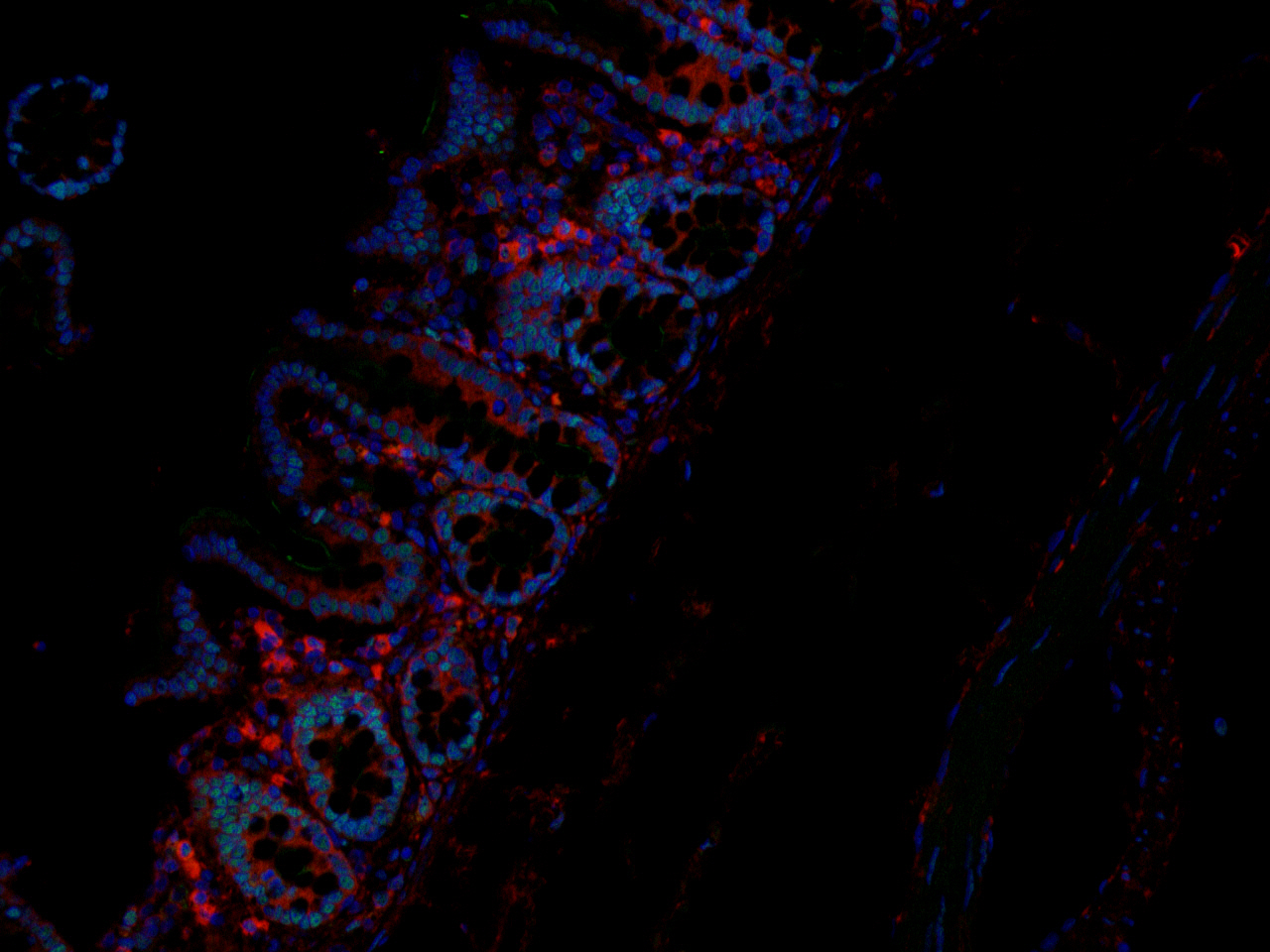

Supplement: Supplementary file 4 [file DataSheet5.ZIP › Supplementary_Material-original data3/FIGURE6/Figures 6J-K(Cecal-IF×200 )/Figure6-J-NLRP3-ASC/SNS/盲 Y1-8 NLRP3(绿)+ASC(红) 200-7 8 9.jpg]

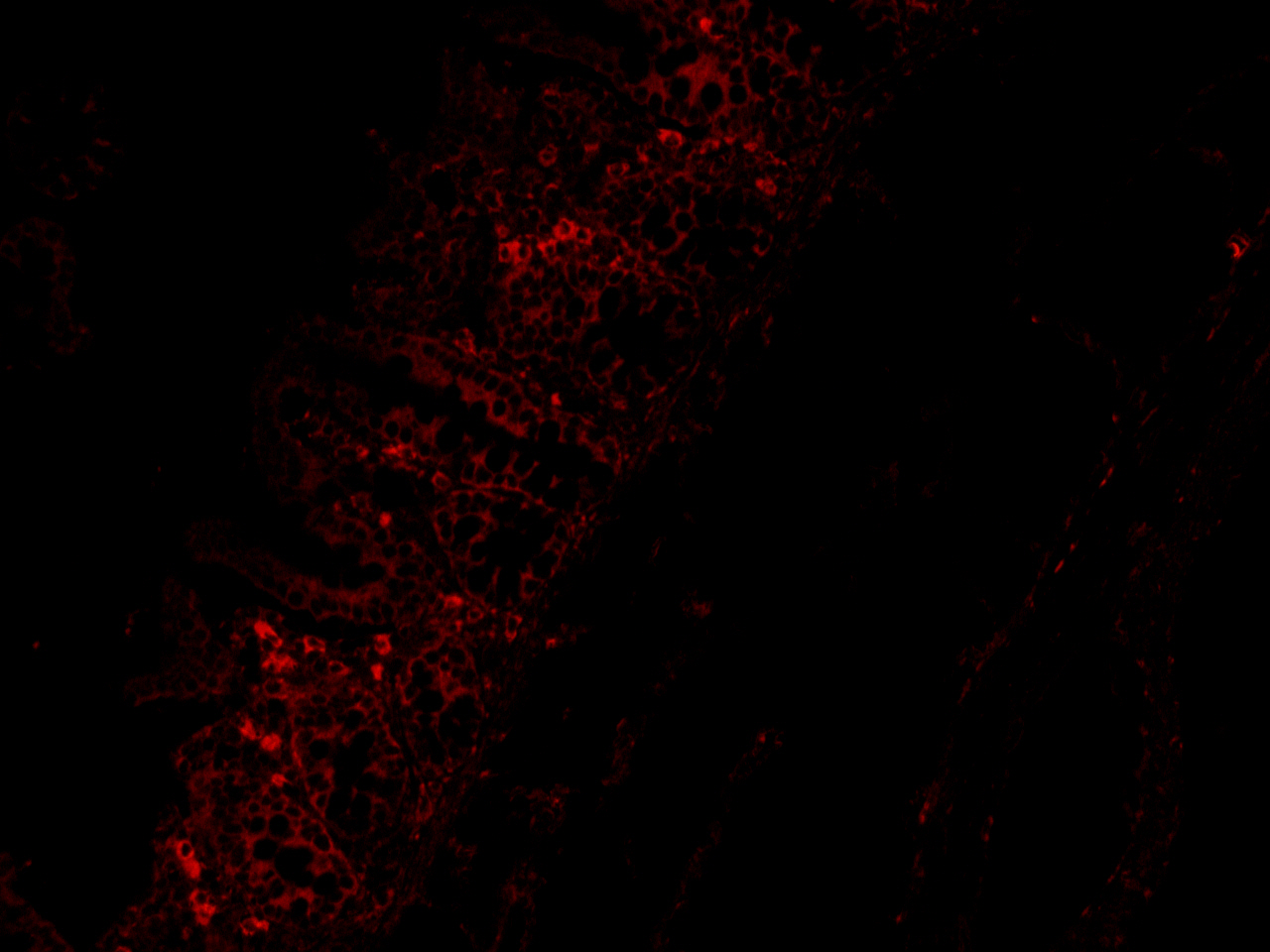

Supplement: Supplementary file 4 [file DataSheet5.ZIP › Supplementary_Material-original data3/FIGURE6/Figures 6J-K(Cecal-IF×200 )/Figure6-J-NLRP3-ASC/SNS/盲 Y1-8 NLRP3(绿)+ASC(红) 200-7.jpg]

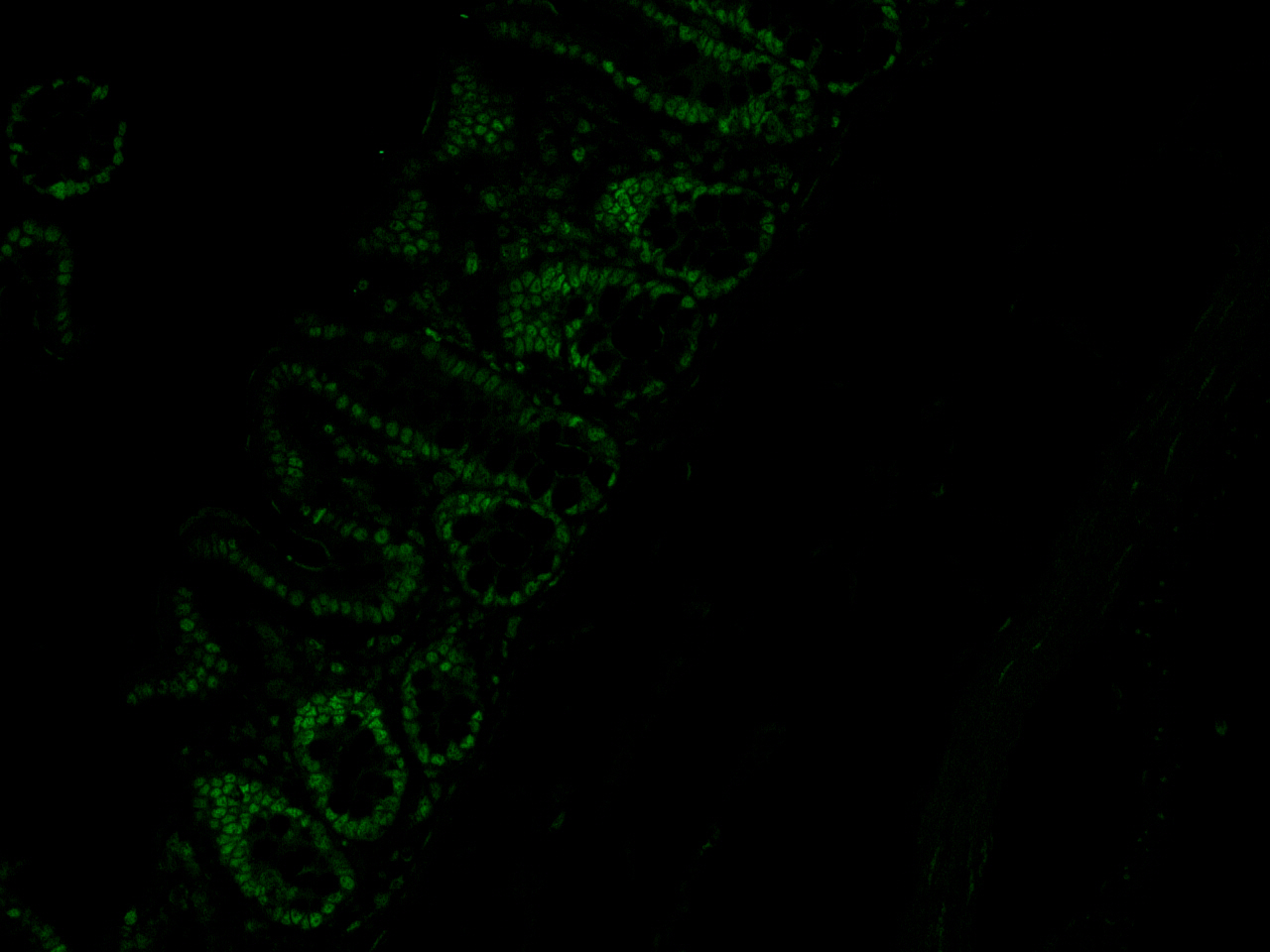

Supplement: Supplementary file 4 [file DataSheet5.ZIP › Supplementary_Material-original data3/FIGURE6/Figures 6J-K(Cecal-IF×200 )/Figure6-J-NLRP3-ASC/SNS/盲 Y1-8 NLRP3(绿)+ASC(红) 200-8.jpg]

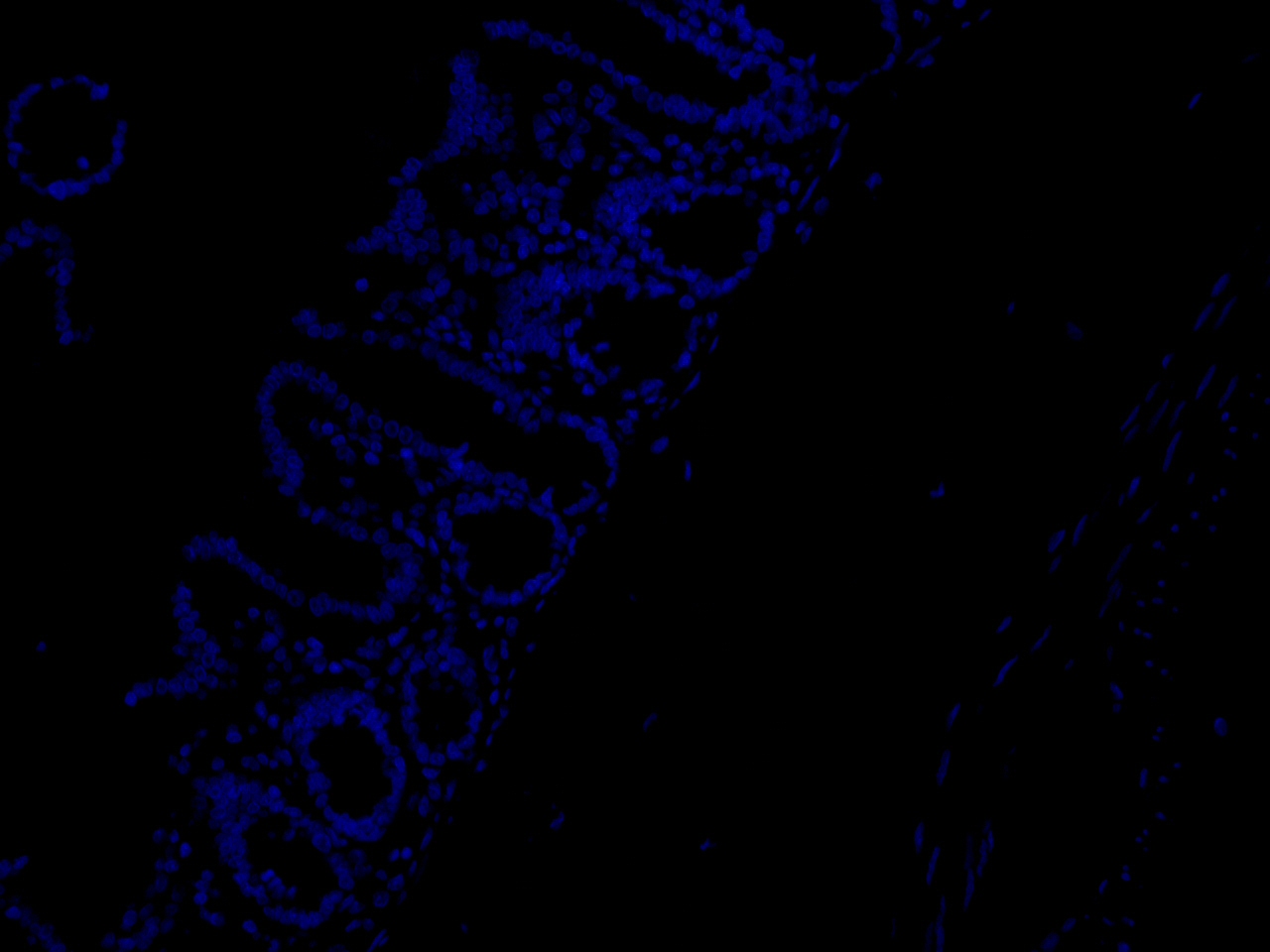

Supplement: Supplementary file 4 [file DataSheet5.ZIP › Supplementary_Material-original data3/FIGURE6/Figures 6J-K(Cecal-IF×200 )/Figure6-J-NLRP3-ASC/SNS/盲 Y1-8 NLRP3(绿)+ASC(红) 200-9.jpg]

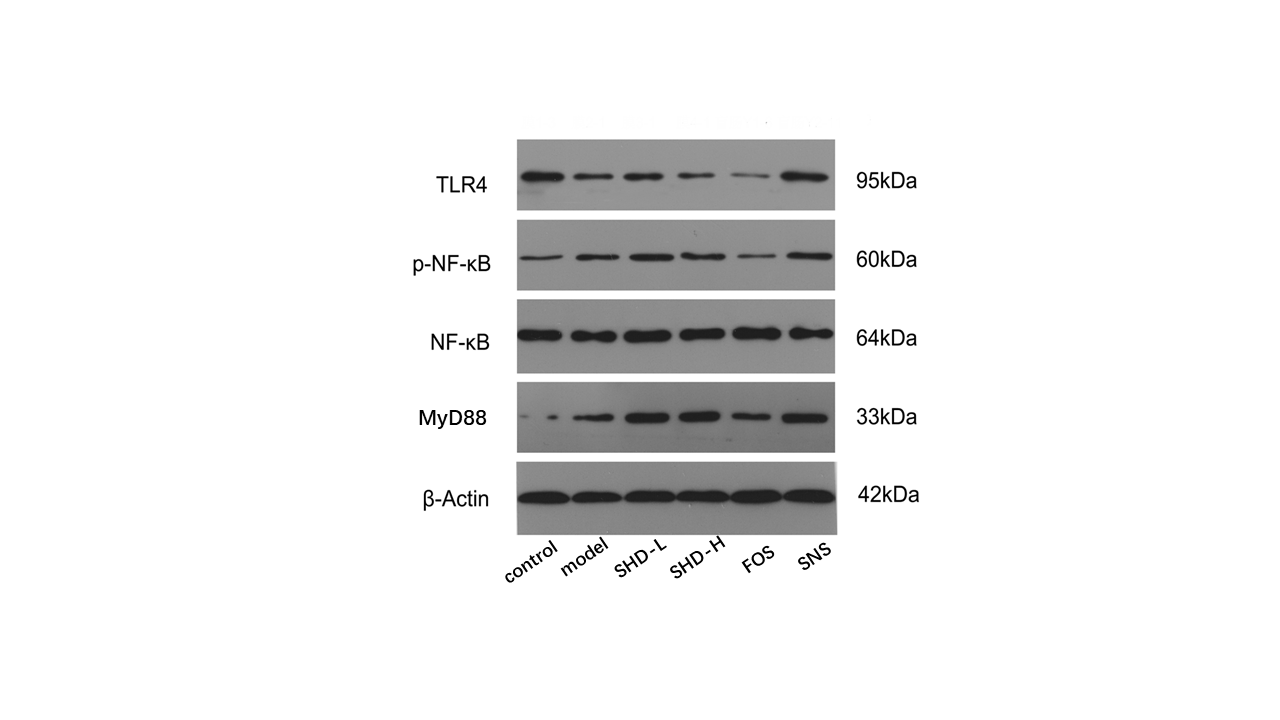

Supplement: Supplementary file 4 [file DataSheet5.ZIP › Supplementary_Material-original data3/FIGURE6/Figures 6L-O(Cecal-WB)/Figure 6L-Western blot analysis.tif]

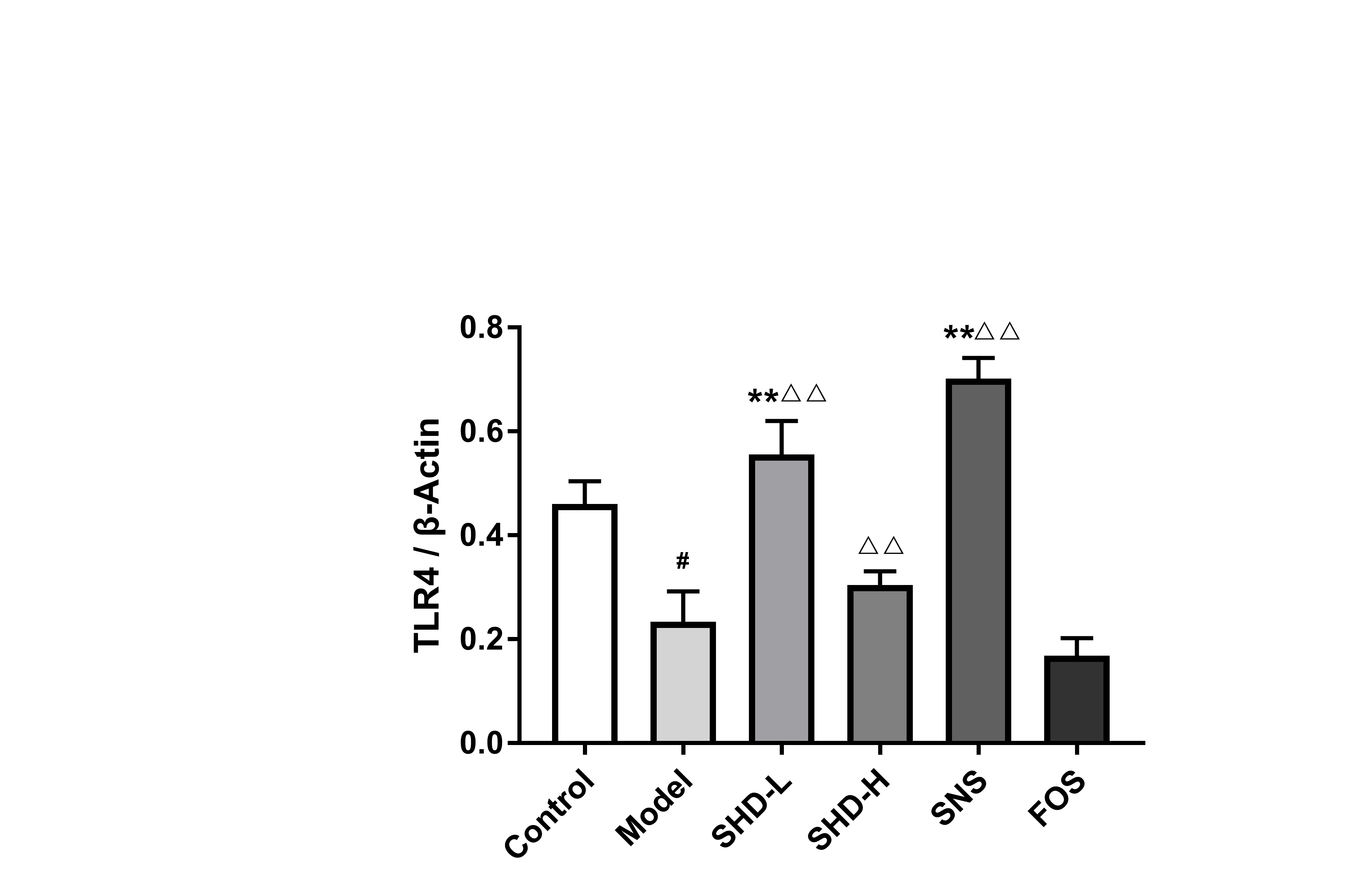

Supplement: Supplementary file 4 [file DataSheet5.ZIP › Supplementary_Material-original data3/FIGURE6/Figures 6L-O(Cecal-WB)/Figure 6M-Cecal-TLR4-WB.tif]

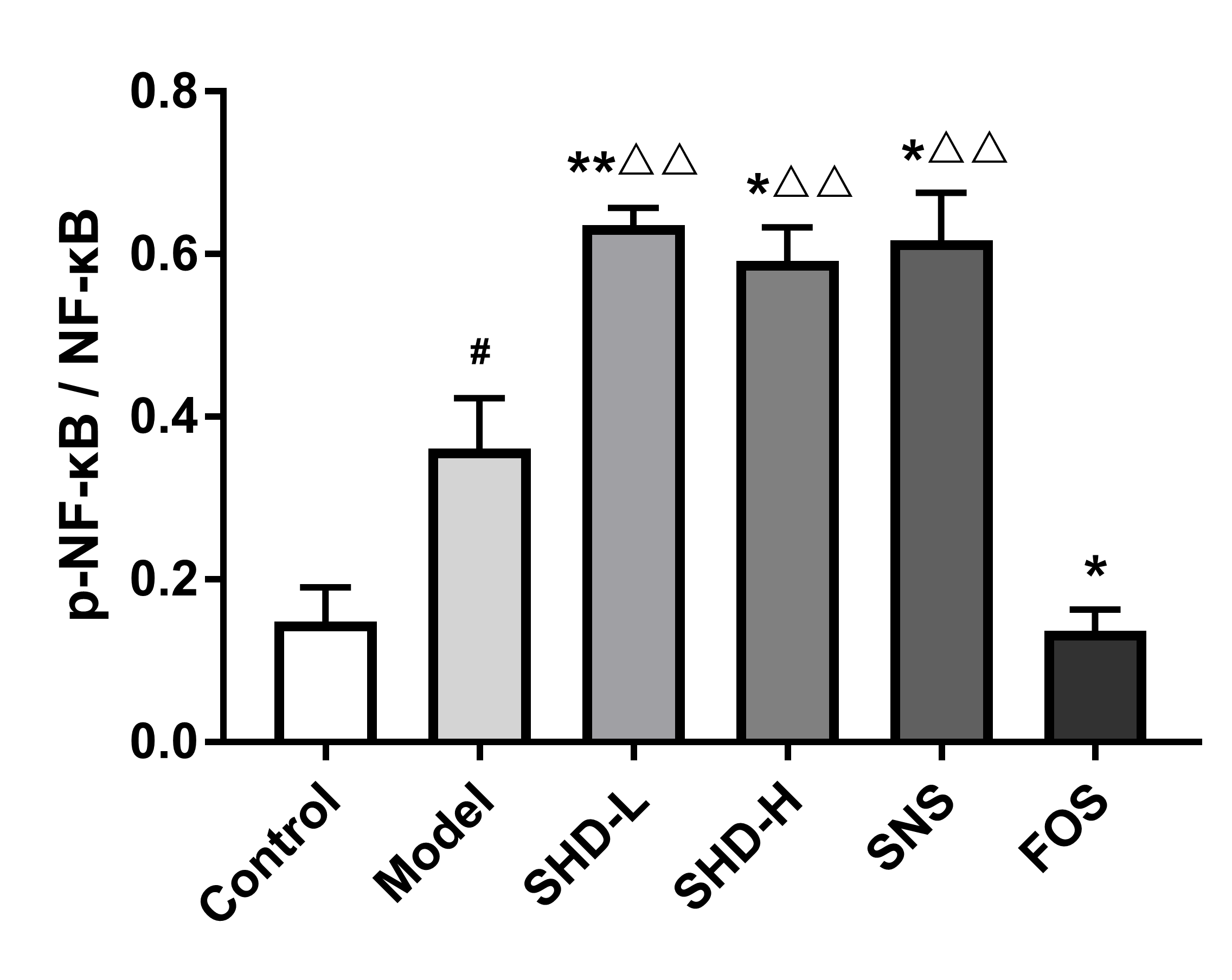

Supplement: Supplementary file 4 [file DataSheet5.ZIP › Supplementary_Material-original data3/FIGURE6/Figures 6L-O(Cecal-WB)/Figure 6N-Cecal-p-NF-κB_NF-Κb-WB.tif]

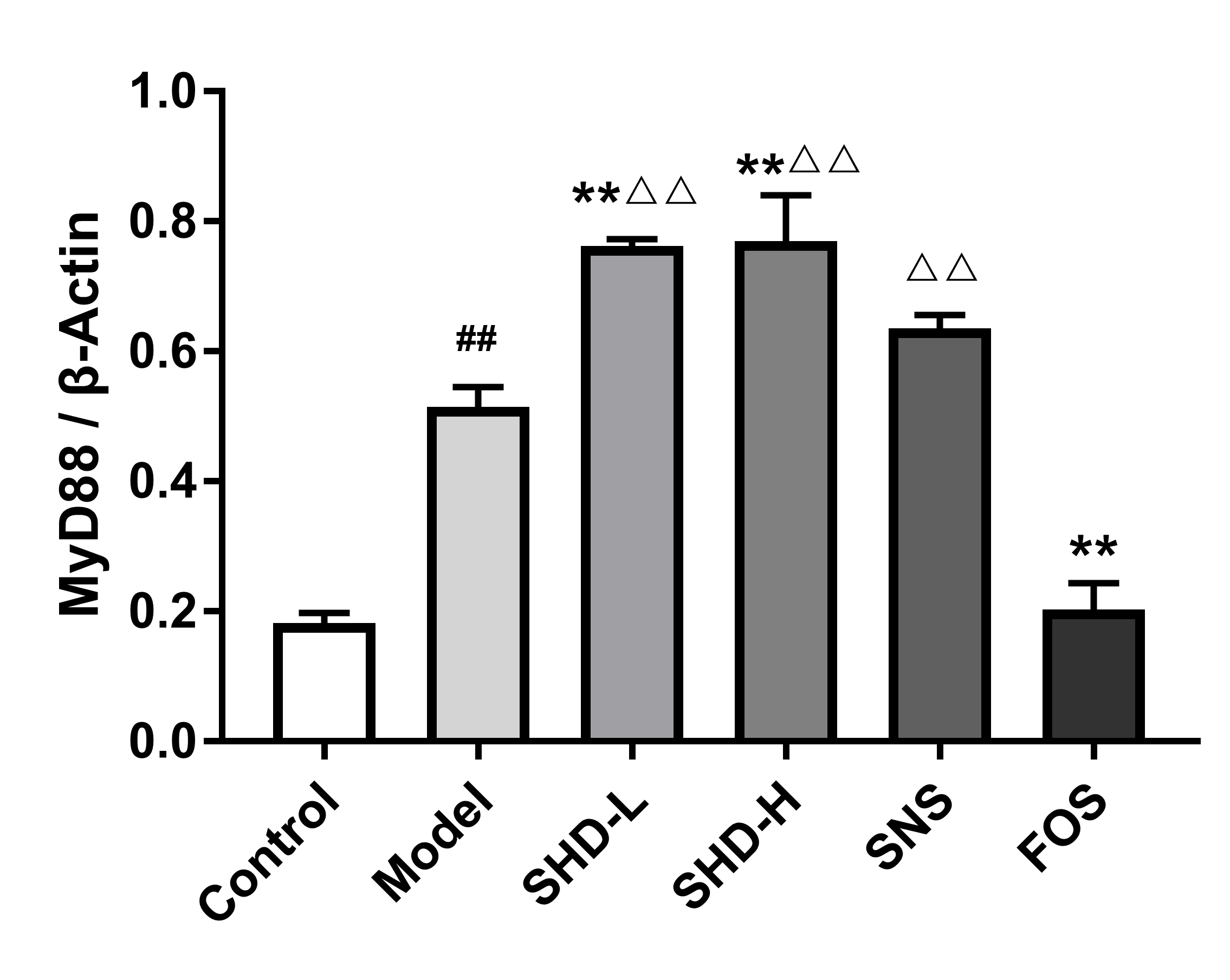

Supplement: Supplementary file 4 [file DataSheet5.ZIP › Supplementary_Material-original data3/FIGURE6/Figures 6L-O(Cecal-WB)/Figure6O-Cecal-MyD88_β-Actin-WB.tif]

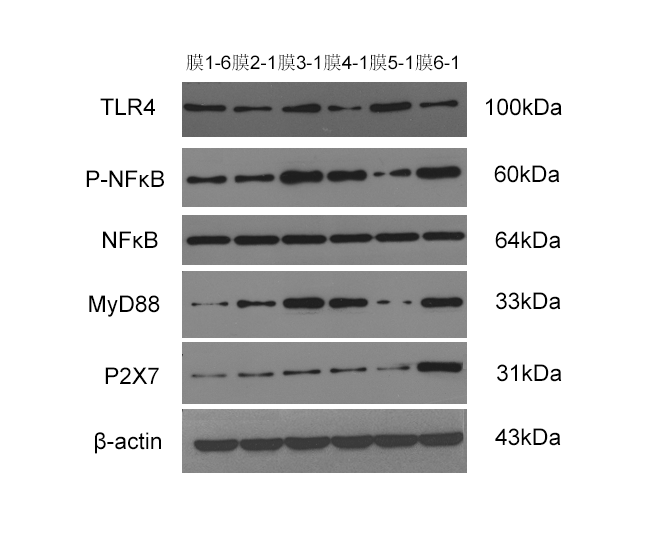

Supplement: Supplementary file 4 [file DataSheet5.ZIP › Supplementary_Material-original data3/FIGURE6/Figures 6L-O(Cecal-WB)/Western Blot Data Analysis and Gray value Pictures/western blot Gray value-Cecal1.tif]

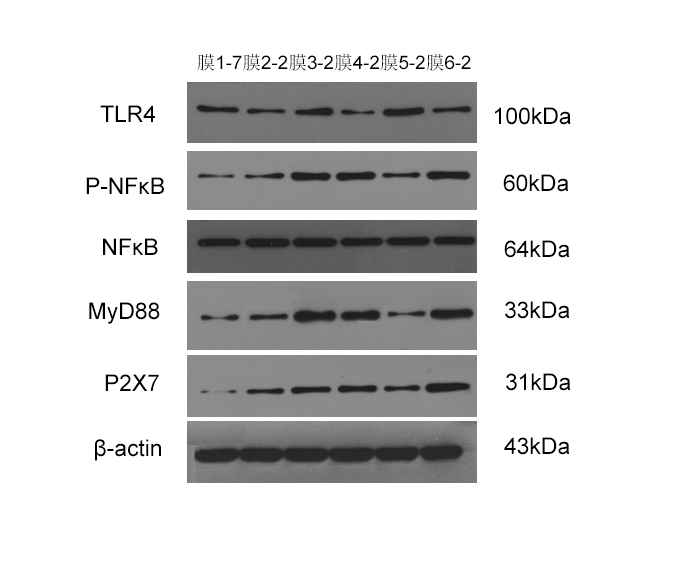

Supplement: Supplementary file 4 [file DataSheet5.ZIP › Supplementary_Material-original data3/FIGURE6/Figures 6L-O(Cecal-WB)/Western Blot Data Analysis and Gray value Pictures/western blot Gray value-Cecal2.tif]

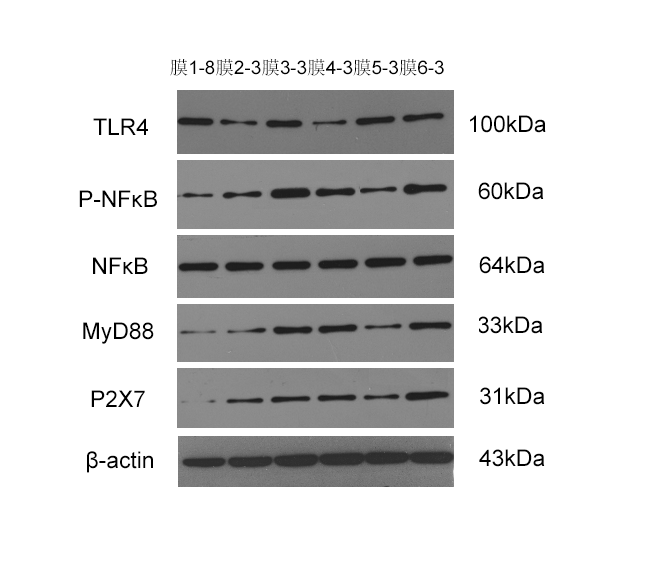

Supplement: Supplementary file 4 [file DataSheet5.ZIP › Supplementary_Material-original data3/FIGURE6/Figures 6L-O(Cecal-WB)/Western Blot Data Analysis and Gray value Pictures/western blot Gray value-Cecal3.tif]

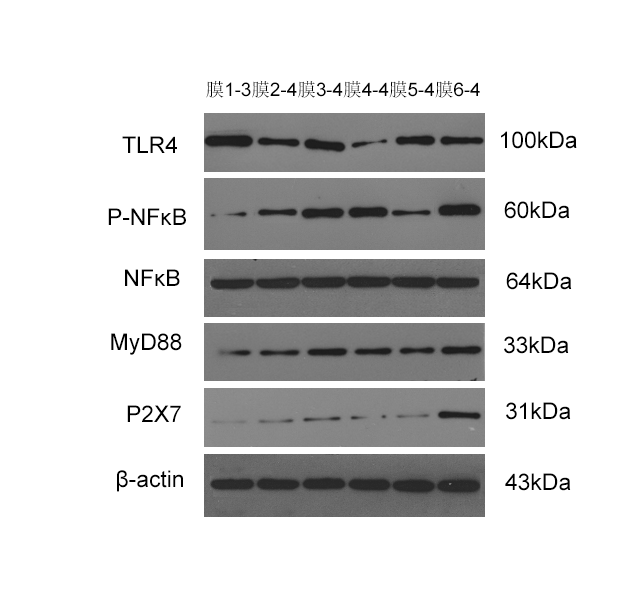

Supplement: Supplementary file 4 [file DataSheet5.ZIP › Supplementary_Material-original data3/FIGURE6/Figures 6L-O(Cecal-WB)/Western Blot Data Analysis and Gray value Pictures/western blot Gray value-Cecal4.tif]

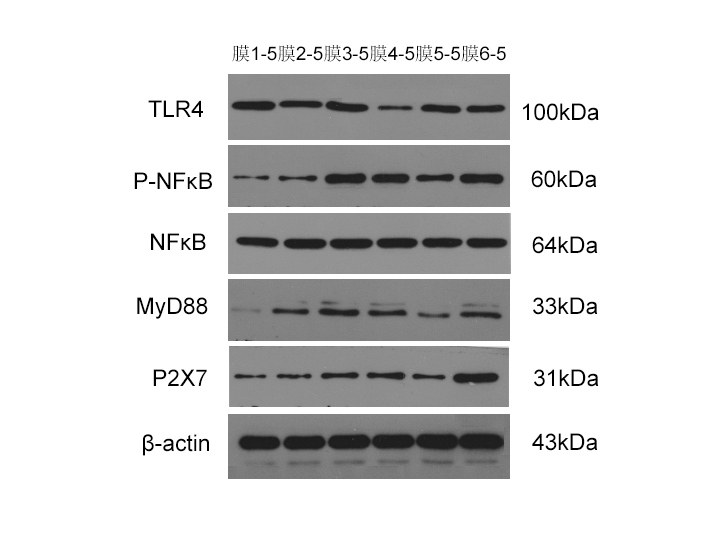

Supplement: Supplementary file 4 [file DataSheet5.ZIP › Supplementary_Material-original data3/FIGURE6/Figures 6L-O(Cecal-WB)/Western Blot Data Analysis and Gray value Pictures/western blot Gray value-Cecal5.tif]
