# Supplementary material for: Shugan Hewei Decoction Alleviates Cecum Mucosal Injury and Improves Depressive- and Anxiety-Like Behaviors in Chronic Stress Model Rats by Regulating Cecal Microbiota and Inhibiting NLRP3 Inflammasome
Source: Front Pharmacol. 2021 Dec 20;12:766474. doi: 10.3389/fphar.2021.766474 (PMC8721152; doi:10.3389/fphar.2021.766474)
Supplement: Supplementary file 5 [file DataSheet1.docx]

Supplementary Material

# Supplementary Tables

**Supplementary Table S1.** The composition of Shugan Hewei Decoction (SHD)

| TCM materials  (pinyin) | Latin name | Species name | Herbal medicine name | Dry weight (g) of daily  dose in clinic | |
| --- | --- | --- | --- | --- | --- |
| Chaihu | *Bupleurum chinense* DC. | Apiaceae | Bupleuri Radix | | 10 |
| Baishao | *Paeonia lactiflora* Pall. | Paeoniaceae | Paeoniae Radix Alba | | 10 |
| Zhishi | *Citrus × aurantium* L. | Rutaceae | Aurantii Fructus Immaturus | | 10 |
| Yujin | *Curcuma aromatica* Salisb. | Zingiberaceae | Curcumae Radix | | 10 |
| Sharen | *Wurfbainia villosa* (Lour.) Skornick. & A.D.Poulsen | Zingiberaceae | Amomi Fructus | | 10 |
| Baizhu | *Atractylodes macrocephala* Koidz. | Asteraceae | Atractylodis Macrocephalae Rhizoma | | 15 |
| Muxiang | *Aucklandia costus* Falc. | Asteraceae | Aucklandiae Radix | | 10 |
| Huanglian | *Coptis chinensis* Franch. | Ranunculaceae | Coptidis Rhizoma | | 6 |
| Wuzhuyu | *Tetradium ruticarpum* (A.Juss.) T.G.Hartley | Rutaceae | Euodiae Fructus | | 6 |
| Gancao | *Glycyrrhiza uralensis* Fisch. ex DC. | Fabaceae | Glycyrrhizae Radix et Rhizoma | | 6 |
| Total |  |  |  | | 93 |

**Supplementary Table S2**. Schedule of CUS procedures

| Stressors | Day of CUS procedures | | |
| --- | --- | --- | --- |
| food deprivation 24 h | 4.11 | 4.18 | 4.29 |
| water deprivation 24 h | 4.12 | 4.19 | 4.30 |
| squeezing tail 1 min | 4.16 | 4.24 | 4.28 |
| swimming in 4℃water 5 min | 4.14 | 4.23 | 4.27 |
| shaking 1 time /s for 5 min | 4.17 | 4.22 | 4.26 |
| reversal of the light/dark | 4.13 | 4.20 | 5.1 |
| shocking on the plantar | 4.15 | 4.21 | 4.25 |

**Supplementary Table S3.** Bristol stool form scale

| Type | Stool form | Score |
| --- | --- | --- |
| Type1 | Separate hard lumps, like nuts. | Type 1, 2 ,3 were constipations, for 1 score for each type |
| Type2 | Sausage-shaped but lumpy. |  |
| Type3 | Like a sausage or snake but with cracks on its surface. |  |
| Type4 | Like a sausage or snake, smooth and soft. | Types 4 and 5 are normal fecal shape, with 2 scores for each type |
| Type5 | Soft blobs with clear-cut edges. |  |
| Type6 | Fluffy pieces with ragged edges, a mushy stool. | Type 6 and 7 were diarrhea fecalshape, with 3 scores for each  type |
| Type7 | Watery, no solid pieces. |  |

**Supplementary Table S4.** Effect of SHD on the weight growth, the Bristol score and fecal moisture content（‾x±SEM，n=5~10）

| Group | Weight gain（g） | Bristol score | Fecal moisture content（%） |
| --- | --- | --- | --- |
| Control | 140.63±9.33  101.25±3.39^##^  128.13±3.53^*^  134.38±7.93^**^  90.63±6.34 | 2.10±0.10 | 11.42±0.42 |
| Model |  | 1.10±0.10^##^ | 9.72±0.94 |
| SHD-L |  | 1.50±0.16 | 15.11±1.03^*^ |
| SHD-H |  | 2.30±0.15^**^ | 18.91±1.58^**ΔΔ^ |
| SNS |  | 1.80±0.25^*^ | 13.35±1.07 |
| FOS | 108.12±6.45 | 1.80±0.20^*^ | 11.64±0.36 |

The data are expressed as the mean±SEM. ^#^*P* ＜0.05，^##^*P*＜0.01 vs. the control group. **P*＜0.05，***P*＜0.01 vs. the model group. **^Δ^***P* ＜0.05，**^ΔΔ^***P* ＜0.01 vs. the FOS group.

**Supplementary Table S5.** Effect of SHD on the Sucrose Preference test and Forced Swimming test（‾x±SEM，n=8）

| Group | Sucrose preference（%） | Immobility time（s） |
| --- | --- | --- |
| Control | 93.37±1.22 | 10.70±1.27 |
| Model | 55.12±5.91^##^ | 16.42±2.40^#^ |
| SHD-L | 86.50±3.85^**^ | 10.42±1.55^*ΔΔ^ |
| SHD-H | 89.25±1.76^**^ | 11.29±1.17^ΔΔ^ |
| SNS | 84.75±2.48^**^ | 17.01±1.97 |
| FOS | 79.25±5.06^**^ | 20.85±2.65 |

**Supplementary Table S6.** Effect of SHD on the Open-field test（‾x±SEM，n=8）


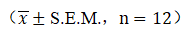

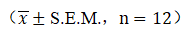


| Group | total movement distance（cm） | stationary time（s） | central area crossing lattice |
| --- | --- | --- | --- |
| Control | 489.00±53.00 | 91.76±8.14 | 13.13±0.74 |
| Model | 310.85±34.86^#^ | 121.34±5.85^#^ | 7.13±0.54^##^ |
| SHD-L | 501.47±56.61^*^ | 91.20±6.38^*Δ^ | 7.63±0.50 |
| SHD-H | 512.18±51.49^*^ | 90.79±7.24^*Δ^ | 10.13±0.87^*ΔΔ^ |
| SNS | 382.14±29.57 | 99.78±3.92 | 7.50±0.53 |
| FOS | 414.70±52.68 | 117.64±6.23 | 7.13±0.41 |

**Supplementary Table S7** Optimized sequence information

| Amplified Region | Samples | Sequences | Bases(bp) | Average Length |
| --- | --- | --- | --- | --- |
| V3-V4 | 49 | 2888146 | 1199415504 | 415.289083031 |

**Supplementary Table S8** Effect of SHD on Alpha diversity of cecal contents

of rats in each group（‾x±SD，n=7～8）

| Group | simpson | chao |
| --- | --- | --- |
| Control | 0.029±0.004 | 784.45±26.95 |
| Model | 0.053±0.019^##^ | 669.99±57.06^##^ |
| SHD-L | 0.034±0.011^*^ | 701.83±28.84^ΔΔ^ |
| SHD-H | 0.029±0.003^*Δ^ | 678.98±22.31^ΔΔ^ |
| SNS | 0.033±0.023 | 681.29±42.71^ΔΔ^ |
| FOS | 0.056±0.036 | 537.12±65.02^*^ |

The data are expressed as the mean±SD. ^#^*P* ＜0.05，^##^*P*＜0.01 vs. the Control group. **P*＜0.05，***P*＜0.01 vs. the Model group. **^Δ^***P* ＜0.05，**^ΔΔ^***P* ＜0.01 vs. the FOS group.

**Supplementary Table S9** Effect of SHD on NLRP3、Caspase-1、IL-1β、IL-18 at the serum

of rats in each group（‾x±SEM，n=5）

| Group | NLRP3 | Caspase-1 | IL-1β | IL-18 |
| --- | --- | --- | --- | --- |
| Control | 8.71±1.19 | 22.77±4.14 | 209.34±42.07 | 2.24±0.10 |
| Model | 17.23±0.73^##^ | 68.22±3.58^##^ | 502.24±21.75^##^ | 0.86±0.17^##^ |
| SHD-L | 12.94±0.52^**^**^ΔΔ^** | 51.22±2.71^**^**^Δ^** | 387.20±14.35^**^ | 0.98±0.02**^ΔΔ^** |
| SHD-H | 12.15±0.46^**^**^ΔΔ^** | 42.32±0.81^**^**^ΔΔ^** | 326.60±5.61^**^**^ΔΔ^** | 1.62±0.08^**^**^ΔΔ^** |
| SNS | 14.10±0.64^**^**^ΔΔ^** | 52.23±1.84^**^**^Δ^** | 401.28±13.36^**^ | 1.33±0.06^**^ |
| FOS | 13.30±0.64^**^ | 44.56±1.48^**^ | 354.93±9.25^**^ | 1.47±0.06^**^ |

**Supplementary Table S10** Effect of SHD on NLRP3, ASC, Caspase-1, IL-1β and IL-18 mRNA

at the cecum tissue of rats in each group（‾x±SEM，n=4～5）

| Group | NLRP3 mRNA | ASC mRNA | Caspase-1 mRNA | IL-1β mRNA | IL-18 mRNA |
| --- | --- | --- | --- | --- | --- |
| Control | 1.205±0.236 | 0.035±0.022 | 0.898±0.079 | 0.278±0.182 | 0.323±0.034 |
| Model | 2.642±0.416^#^ | 0.398±0.106^#^ | 2.043±0.479^#^ | 1.848±0.745^##^ | 1.778±0.250^##^ |
| SHD-L | 2.553±0.472 | 0.415±0.083 | 0.742±0.078^**^ | 0.626±0.365 | 1.395±0.405 |
| SHD-H | 0.712±0.098^**^ | 0.082±0.046 | 0.880±0.281^*^ | 0.250±0.077^*Δ^ | 0.695±0.137^*^ |
| SNS | 0.558±0.293^**^ | 0.132±0.039 | 0.896±0.081^*^ | 0.330±0.037^*ΔΔ^ | 0.673±0.112^*^ |
| FOS | 1.740±0.321 | 0.386±0.136 | 1.108±0.264 | 1.585±0.439 | 1.245±0.365 |

**Supplementary Table S11** Effect of SHD on NLRP3, ASC positive area at the cecal tissue of rats in each group, (%)（‾x±SEM，n=4～6）

| Group | NLRP3 | ASC |
| --- | --- | --- |
| Control | 17.840±3.603 | 0.7603±0.375 |
| Model | 24.640±2.956^#^ | 1.285±0.418 |
| SHD-L | 16.120±2.486 | 1.832±0.418 |
| SHD-H | 13.500±3.857*****^Δ^** | 3.006±0.445*** |
| SNS | 6.526±0.624**** | 9.839±1.576**** |
| FOS | 6.277±0.718 | 10.8±1.867 |

**Supplementary Table S12** Effect of SHD on NLRP3, Caspase-1 positive area at the cecal tissue of rats in each group, (%)（‾x±SEM，n=4～6）

| Group | NLRP3 | Caspase-1 |
| --- | --- | --- |
| Control | 16.580±4.508 | 1.348±0.389 |
| Model | 25.180±1.077^#^ | 2.227±0.917^#^ |
| SHD-L | 11.430±3.632^*Δ^ | 2.66±0.251 |
| SHD-H | 15.970±0.553*****^Δ^** | 1.336±0.019***^Δ^ |
| SNS | 26.580±4.429 | 15.96±1.828**** |
| FOS | 24.430±3.036 | 12.64±2.91**** |

**Supplementary Table S13** Correlations of intestinal microbiota (the top 9 of total abundance in phylum level) and NLRP3 inflammasomes mRNA in cecum tissue

| Phylum | NLRP3 mRNA | | ASC mRNA | | Caspase-1mRNA | | IL-1β mRNA | | IL-18 mRNA | |
| --- | --- | --- | --- | --- | --- | --- | --- | --- | --- | --- |
|  | R | P | R | P | R | P | R | P | R | P |
| Firmicutes | 0.270 | 0.061 | 0.275 | 0.056 | 0.289 | 0.044 | 0.266 | 0.064 | 0.288 | 0.045 |
| Bacteroidetes | -0.287 | 0.046 | -0.233 | 0.108 | -0.333 | 0.019 | -0.218 | 0.133 | -0.299 | 0.037 |
| Proteobacteria | 0.090 | 0.538 | -0.183 | 0.208 | 0.035 | 0.811 | -0.067 | 0.649 | -0.128 | 0.381 |
| Saccharibacteria | 0.346 | 0.015 | 0.152 | 0.297 | 0.496 | 0.000 | -0.071 | 0.627 | 0.562 | 0.000 |
| Actinobacteria | 0.252 | 0.081 | 0.103 | 0.483 | 0.083 | 0.569 | -0.081 | 0.582 | 0.158 | 0.279 |
| Tenericutes | 0.285 | 0.047 | 0.094 | 0.520 | 0.587 | 0.000 | -0.144 | 0.323 | 0.424 | 0.002 |
| Cyanobacteria | 0.001 | 0.993 | 0.230 | 0.113 | -0.030 | 0.839 | 0.137 | 0.348 | 0.115 | 0.432 |
| Spirochaetae | -0.082 | 0.575 | 0.001 | 0.997 | -0.059 | 0.689 | -0.199 | 0.171 | -0.052 | 0.724 |
| Deferribacteres | -0.412 | 0.003 | -0.324 | 0.023 | -0.155 | 0.288 | -0.210 | 0.148 | -0.099 | 0.500 |

The whole data set consisted of the pooled data of the intervention groups and the model, control groups. R value, the closer this value is to 1, the more positive the correlation, and the closer it is to -1, the more negative. *P* ≤ 0.001 indicates an extremely significant correlation, 0.001 ＜ *P* ≤ 0.01 indicates a very significant correlation, 0.01 ＜*P* ≤0.05 indicates a significant correlation.

**Supplementary Table S14** Correlations of cecal microbiota (the top 9 of total abundance in genus level) and NLRP3 inflammasomes mRNA in cecum tissue

| Genus | NLRP3 mRNA | | ASC mRNA | | Caspase-1m RNA | | IL-1β mRNA | | IL-18 mRNA | |
| --- | --- | --- | --- | --- | --- | --- | --- | --- | --- | --- |
|  | R | P | R | P | R | P | R | P | R | P |
| norank_f__Bacteroidales_S24-7_group | -0.384 | 0.006 | 0.010 | 0.948 | -0.114 | 0.434 | 0.104 | 0.476 | 0.104 | 0.478 |
| Lactobacillus | -0.097 | 0.507 | -0.126 | 0.388 | 0.104 | 0.478 | 0.000 | 0.999 | 0.070 | 0.634 |
| Prevotella_9 | -0.068 | 0.645 | -0.261 | 0.070 | -0.111 | 0.447 | -0.355 | 0.012 | -0.272 | 0.058 |
| Lachnospiraceae_NK4A136_group | -0.032 | 0.829 | 0.064 | 0.660 | 0.456 | 0.001 | -0.076 | 0.605 | 0.494 | 0.000 |
| unclassified_f__Lachnospiraceae | 0.145 | 0.322 | -0.079 | 0.590 | -0.095 | 0.517 | 0.068 | 0.643 | -0.168 | 0.249 |
| Roseburia | -0.073 | 0.618 | -0.105 | 0.474 | -0.051 | 0.730 | -0.217 | 0.135 | -0.289 | 0.044 |
| Blautia | -0.189 | 0.195 | 0.061 | 0.675 | -0.252 | 0.080 | 0.080 | 0.584 | -0.248 | 0.086 |
| Ruminococcaceae_UCG-005 | -0.062 | 0.673 | -0.086 | 0.556 | 0.157 | 0.280 | -0.075 | 0.608 | 0.080 | 0.584 |
| Prevotella_1 | -0.044 | 0.765 | -0.108 | 0.460 | -0.158 | 0.278 | -0.265 | 0.066 | -0.225 | 0.121 |

**Supplementary Table S15** Correlations of cecal microbiota (the top 9 of total abundance in phylum level) and NLRP3 inflammasomes in serum

| Phylum | NLRP3 | | | | Caspase-1 | | | | IL-1β | | | | | IL-18 | | | | |
| --- | --- | --- | --- | --- | --- | --- | --- | --- | --- | --- | --- | --- | --- | --- | --- | --- | --- | --- |
|  | R | P | | R | | | | P | | R | | | P | | R | | P | |
| Firmicutes | 0.353 | | 0.013 | | | 0.471 | 0.001 | | | | -0.408 | 0.000 | | | | 0.505 | | 0.004 |
| Bacteroidetes | -0.337 | | 0.018 | | | -0.465 | 0.001 | | | | 0.393 | 0.000 | | | | -0.519 | | 0.005 |
| Proteobacteria | -0.105 | | 0.472 | | | -0.151 | 0.299 | | | | 0.042 | 0.516 | | | | -0.095 | | 0.775 |
| Saccharibacteria | 0.149 | | 0.305 | | | 0.380 | 0.007 | | | | -0.526 | 1.684 | | | | 0.573 | | 0.000 |
| Actinobacteria | 0.223 | | 0.124 | | | 0.230 | 0.111 | | | | -0.043 | 0.063 | | | | 0.267 | | 0.768 |
| Tenericutes | 0.220 | | 0.129 | | | 0.406 | 0.004 | | | | -0.289 | 0.000 | | | | 0.493 | | 0.044 |
| Cyanobacteria | 0.131 | | 0.368 | | | 0.116 | 0.427 | | | | -0.338 | 0.206 | | | | 0.183 | | 0.017 |
| Spirochaetae | -0.017 | | 0.908 | | | -0.107 | 0.462 | | | | 0.351 | 0.372 | | | | -0.130 | | 0.013 |
| Deferribacteres | -0.474 | | 0.001 | | | -0.418 | 0.003 | | | | -0.018 | 0.031 | | | | -0.309 | | 0.902 |

**Supplementary Table S16** Correlations of cecal microbiota (the top 9 of total abundance in genus level) and NLRP3 inflammasomes in serum

| Genus | NLRP3 | | Caspase-1 | | IL-1β | | IL-18 | |
| --- | --- | --- | --- | --- | --- | --- | --- | --- |
|  | R | P | R | P | R | P | R | P |
| norank_f__Bacteroidales_S24-7_group | -0.371 | 0.008 | -0.219 | 0.131 | -0.089 | 0.543 | -0.254 | 0.078 |
| Lactobacillus | -0.143 | 0.326 | 0.102 | 0.487 | 0.178 | 0.221 | -0.436 | 0.002 |
| Prevotella_9 | -0.114 | 0.434 | -0.293 | 0.041 | -0.384 | 0.006 | 0.553 | 0.000 |
| Lachnospiraceae_NK4A136_group | -0.063 | 0.669 | 0.198 | 0.173 | 0.412 | 0.003 | -0.565 | 0.000 |
| unclassified_f__Lachnospiraceae | 0.005 | 0.968 | -0.046 | 0.756 | -0.143 | 0.327 | -0.0364 | 0.819 |
| Roseburia | 0.015 | 0.918 | -0.240 | 0.096 | -0.341 | 0.016 | 0.595 | 0.000 |
| Blautia | 0.004 | 0.976 | -0.204 | 0.160 | -0.308 | 0.032 | 0.489 | 0.000 |
| Ruminococcaceae_UCG-005 | -0.097 | 0.509 | 0.012 | 0.937 | 0.073 | 0.617 | -0.149 | 0.307 |
| Prevotella_1 | -0.011 | 0.940 | -0.296 | 0.039 | -0.341 | 0.0167 | 0.612 | 0.000 |

# Supplementary Figures
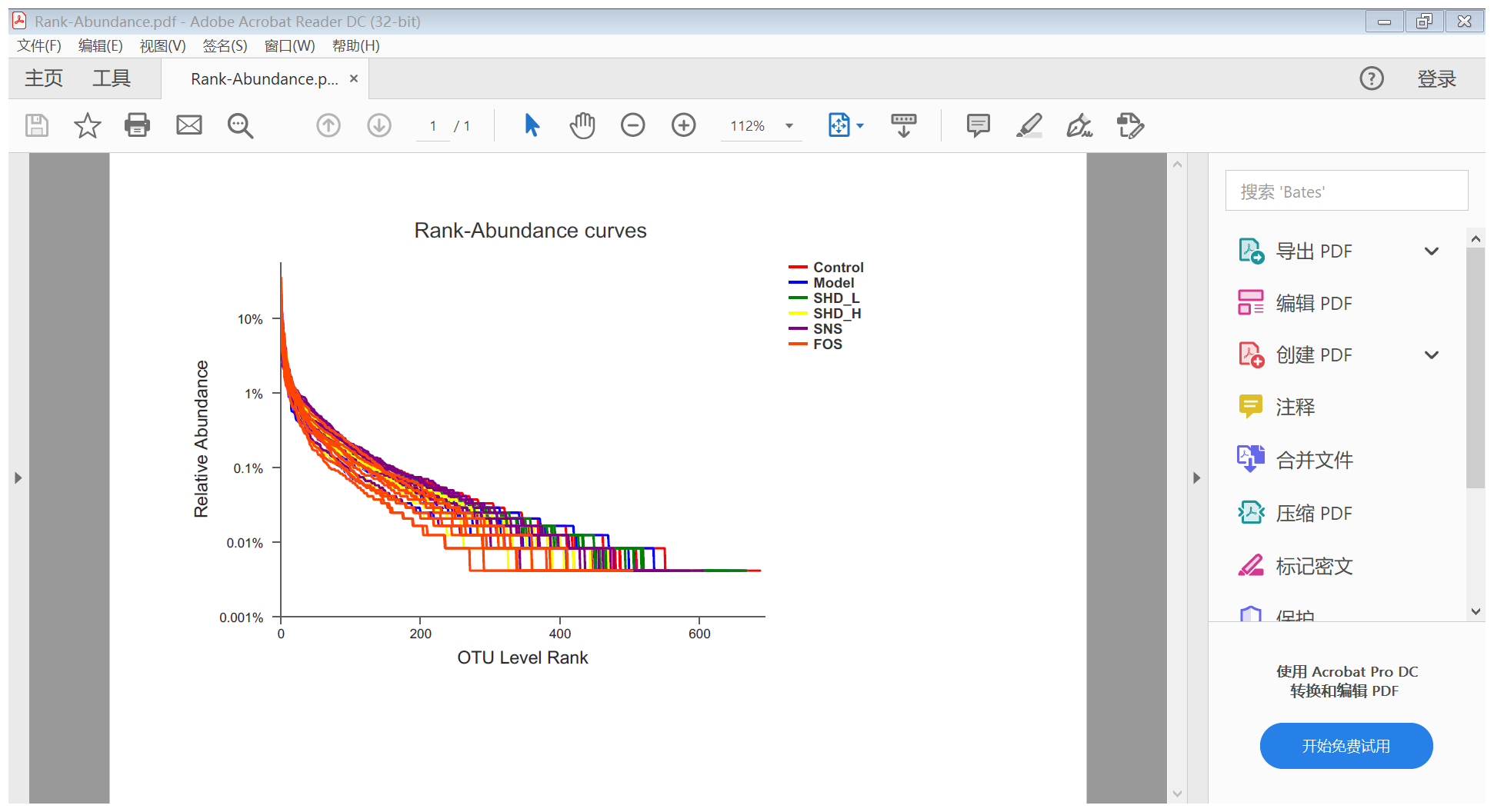


**Supplementary Figure 1.** Rank-abundance curve of OTU classification level of cecal contents of rats in each group.


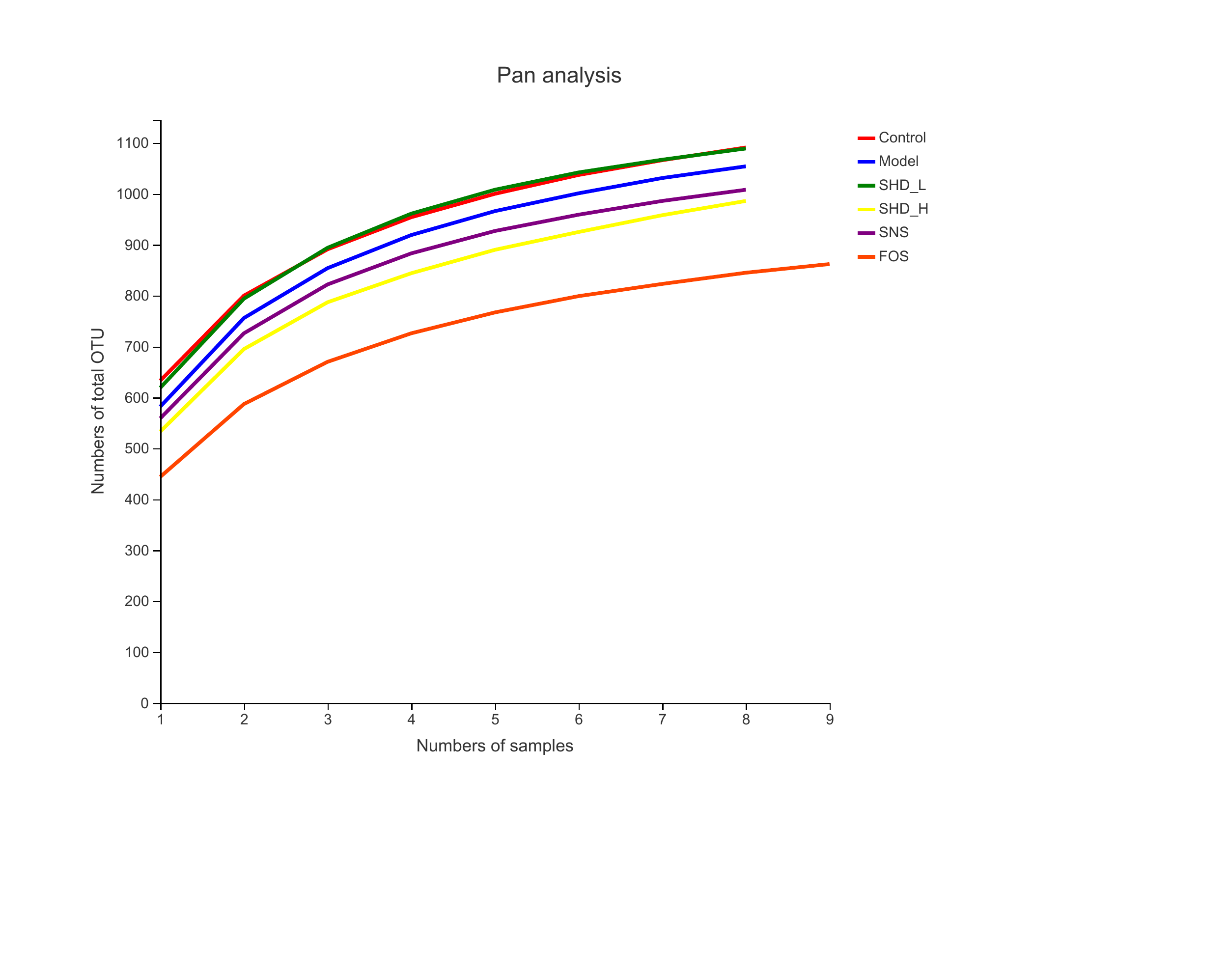


**Supplementary Figure 2.** Pan curve of OTU classification level of cecal contents of rats in each group.


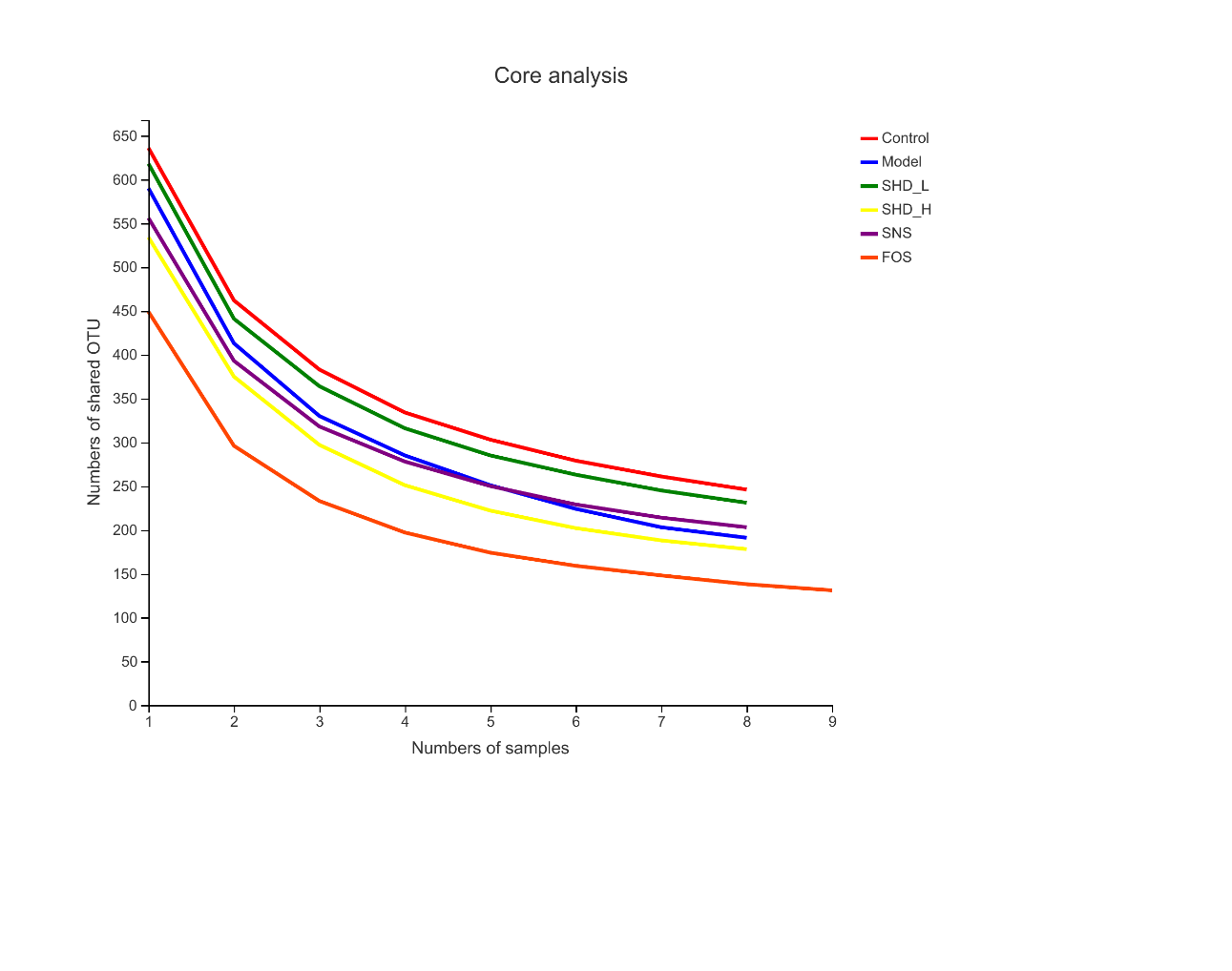


**Supplementary Figure 3.** Core cure of OTU classification level of cecal contents of rats in each group.

**
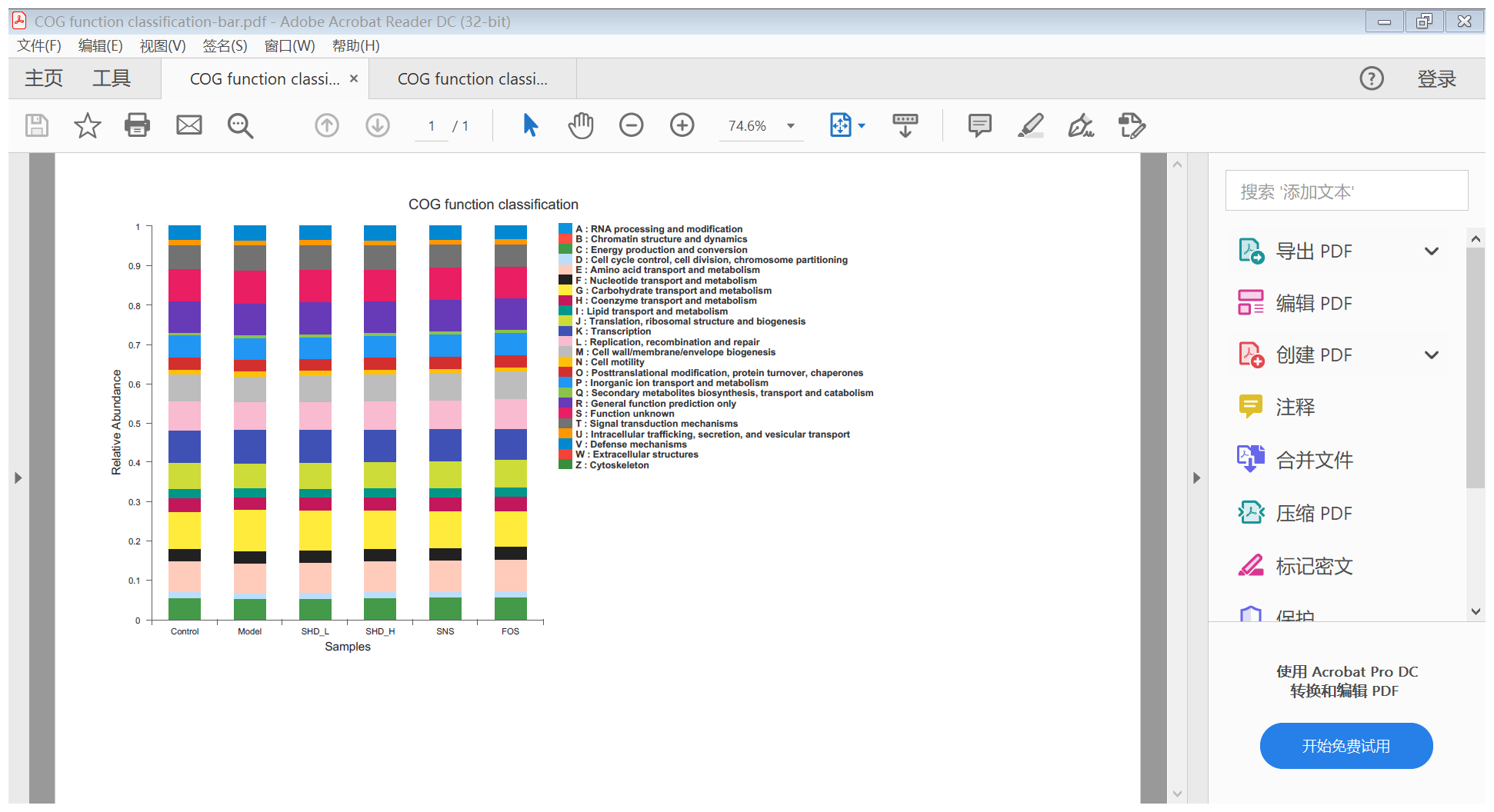
**

**Supplementary Figure 4**. Bar figure about COG functional classification of cecal microbiota of rats in each group


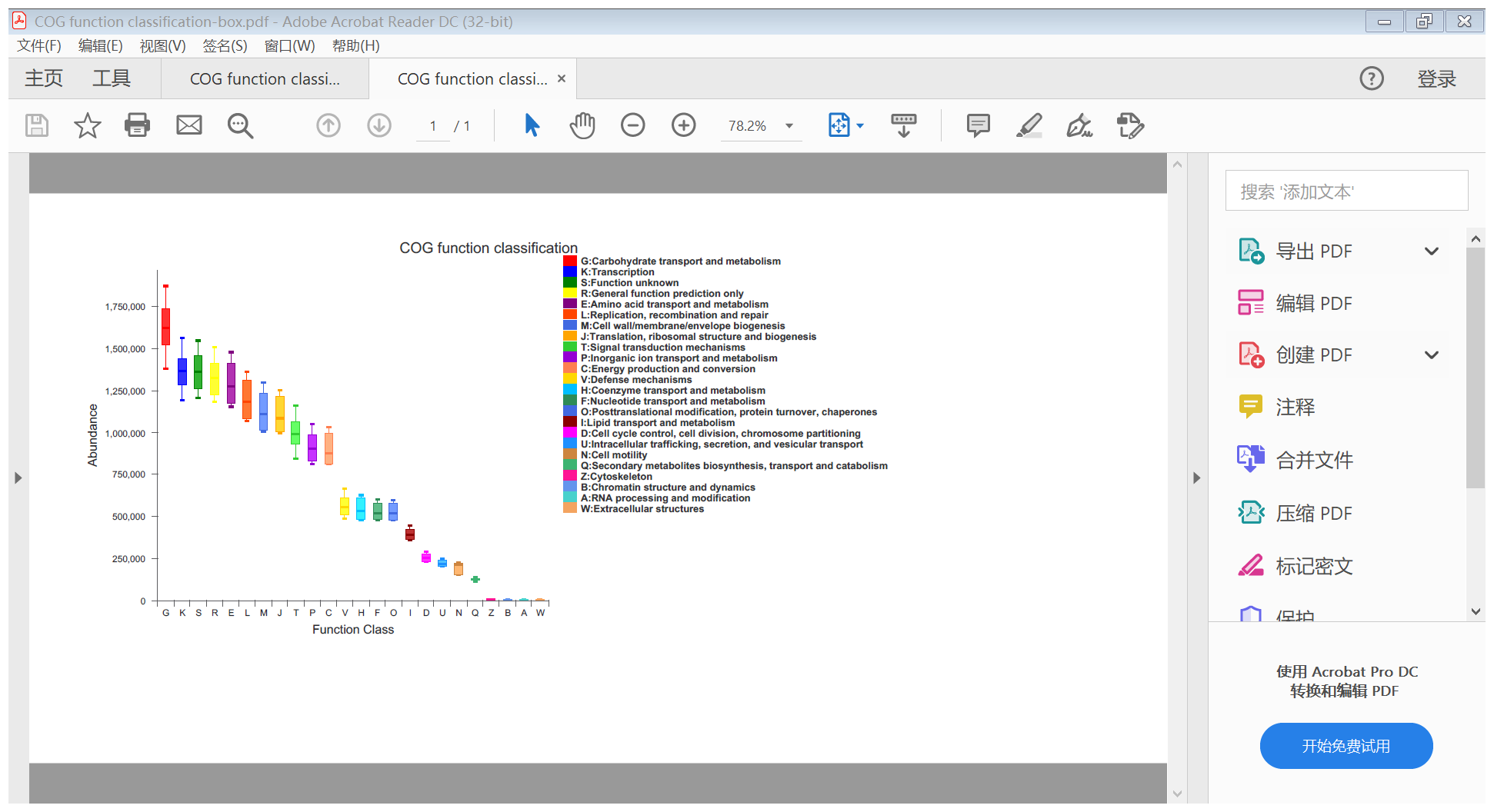


**Supplementary Figure 5.** Box figure about COG functional classification of cecal microbiota of rats in each group.
